# Gene Expression Signatures in AML-12 Hepatocyte Cells upon *Dengue virus* Infection and Acetaminophen Treatment

**DOI:** 10.3390/v12111284

**Published:** 2020-11-10

**Authors:** Jorge G. G. Ferreira, Sandra G. Gava, Eneida S. Oliveira, Izabella C. A. Batista, Gabriel da R. Fernandes, Marina M. Mourão, Carlos E. Calzavara-Silva

**Affiliations:** 1 Imunologia Celular e Molecular, Instituto René Rachou, Belo Horizonte, MG 30190-002, Brazil; jorgekorps@yahoo.com.br (J.G.G.F.); izabella.batista@fiocruz.br (I.C.A.B.); 2 Helmintologia e Malacologia Médica, Instituto René Rachou, Belo Horizonte, MG 30190-002, Brazil; sandra.grossi@fiocruz.br (S.G.G.); marina.mourao@fiocruz.br (M.M.M.); 3 Secretaria Municipal de Saúde, Prefeitura de Belo Horizonte, Belo Horizonte, MG 30550-340, Brazil; eneida.oliveira@pbh.gov.br; 4 Informática de Biossistemas, Instituto René Rachou, Belo Horizonte, MG 30190-002, Brazil; gabriel.fernandes@fiocruz.br

**Keywords:** Dengue, DENV, acetaminophen, hepatocytes, transcriptome, RNA sequencing (RNAseq)

## Abstract

Dengue is an acute viral disease caused by *Dengue virus* (DENV) and is considered to be the most common arbovirus worldwide. The clinical characteristics of dengue may vary from asymptomatic to severe complications and severe organ impairment, particularly affecting the liver. Dengue treatment is palliative with acetaminophen (APAP), usually known as Paracetamol, being the most used drug aiming to relieve the mild symptoms of dengue. APAP is a safe and effective drug but, like dengue, can trigger the development of liver disorders. Given this scenario, it is necessary to investigate the effects of combining these two factors on hepatocyte homeostasis. Therefore, this study aimed to evaluate the molecular changes in hepatocytes resulting from the association between DENV infection and treatment with sub-toxic APAP concentrations. Using an in vitro experimental model of DENV-2 infected hepatocytes (AML-12 cells) treated with APAP, we evaluated the influence of the virus and drug association on the transcriptome of these hepatocytes by RNA sequencing (RNAseq). The virus–drug association was able to induce changes in the gene expression profile of AML-12 cells and here we highlight and explore these changes and its putative influence on biological processes for cellular homeostasis.

## 1. Introduction

Dengue is a re-emerging mosquito-borne viral disease transmitted by the bites of mosquitoes mainly from the genus *Aedes*. The disease is caused by *Dengue virus* (DENV)*,* an enveloped, positive-sense single-stranded RNA virus with four distinct serotypes (DENV-1 to -4). Approximately 3.9 billion people from 128 countries live at risk of infection by DENV [1].

Dengue is an acute febrile illness that has a large clinical spectrum ranging from inapparent infection, flu-like mild undifferentiated fever to severe and possibly lethal vasculopathy [2]. Liver dysfunction is a well-documented dengue complication; thus, altered levels of liver injury biochemical indicators are commonly found in all stages of the disease. In dengue, acute liver failure has been associated with complications such as encephalopathy, severe bleeding, renal failure, metabolic acidosis and consequently high mortality rates [1,3,4,5].

Currently, there are no specific drugs against the DENV infection; thus, dengue treatment is palliative, aiming at alleviating its symptoms and avoiding patient dehydration [1]. The clinical management of dengue is performed by the use of non-steroidal analgesics and anti-inflammatory drugs without acetylsalicylic acid in its composition due to the increased risk of hemorrhagic complications. Approximately 90% of dengue cases are treated with acetaminophen (APAP) [6]. APAP, commercially known as Paracetamol, is a non-opioid and non-steroidal drug, which is largely consumed worldwide and mainly used for fever reduction and temporary relief of mild to moderate pain symptoms [7,8].

APAP is a safe and effective drug but, if used constantly or in high doses, it can induce severe intoxication due to liver damage resulting in progressive hepatic failure and death [9]. According to Pandejpong et al. [6], patients with dengue usually require higher doses of APAP to reduce symptoms, reaching more than 20 g throughout the viremia period. Thus, even though APAP is safe when used following the administration recommendations, some conditions have a direct influence over the APAP metabolism by the human liver and consequently may induce hepatic dysfunction [10].

As DENV disturbs the hepatic homeostasis [11] and there have been case reports in which patients with hepatic impairment developed severe dengue infections [12,13], a few studies investigated the interaction between DENV infection and APAP metabolism into the liver and its contribution to hepatic dysfunction [6,14,15,16,17,18]. However, these studies are limited to describe clinical observations. Here we aim to explore the changes in the molecular pathways of hepatocyte induced by the association between the DENV infection and APAP treatment and hepatocyte homeostasis.

## 2. Materials and Methods

### 2.1. Cell Culture

BHK-21, C6/36 and AML-12 cell lines (Banco de Células do Rio de Janeiro (BCRJ), Brazil) were cultured according to the protocol described by the supplier in their specific media supplemented with 5% heat-inactivated fetal bovine serum (FBS) (Cultilab, Campinas, SP, Brazil) and 1% Antibiotic-Antimycotic Solution (100×) (Thermo Fisher Scientific, Walthan, MA, USA). BHK-21 was cultured in Dulbecco’s modified Eagle’s medium (DMEM) (Thermo Fisher Scientific, Walthan, MA, USA) and incubated at 37 °C in a 5% CO_2_ atmosphere. C6/36 cells were cultured in a Leibowitz 15 (L-15) medium (Cultilab, Campinas, SP, Brazil) and incubated at 28 °C. AML-12 cells were cultured in DMEM plus Ham’s F12 (1:1) (Sigma-Aldrich, St. Louis, MO, USA) supplemented with 0.005 mg/mL insulin, 0.005 mg/mL transferrin, 5 ng/mL selenium (Sigma-Aldrich, St. Louis, MO, USA) and 40 ng/mL dexamethasone (Sigma-Aldrich, St. Louis, MO, USA) and incubated at 37 °C in a 5% CO_2_ atmosphere.

### 2.2. Dengue Virus

The samples of DENV-2, provided from the culture supernatant, were kindly donated by Dr. Erna Geessien Kroon from the Universidade Federal de Minas Gerais (UFMG, Belo Horizonte, MG, Brazil). The DENV-2 was multiplied in C6/36 cells. The cells were infected at a multiplicity of infection of 0.1 (MOI = 0.1) and maintained at 28 °C for 7 days. After this period, the supernatant was collected and clarified by centrifugation at 1800× *g*, 4 °C for 10 min and then posteriorly stored at −80 °C until use. The titration was performed in BHK-21 cells by plaque assays according to the previously described protocol [19].

### 2.3. DENV-2 Infection and Acetaminophen (APAP) Treatment of AML-12 Cells

A total of 10^5^ AML-12 cells/well were plated and cultured for 24 h in 24-well culture plates containing DMEM plus Ham F12 (1:1), supplemented as described above. The culture medium was then removed and cells were split into four groups: (1) cells incubated with DENV-2 (MOI = 1) for 48 h (named DENV infected cells), (2) cells treated with 1 mM of APAP for 24 h after an additional 24 h of culture (named APAP treated cells), (3) cells incubated with DENV-2 (MOI = 1) for 24 h and treated with 1 mM of APAP for an additional 24 h (named DENV infected/APAP treated cells) and (4) untreated cells cultured for 48 h (named untreated cells). The culture supernatant of the three biological replicates from each group was then removed and AML-12 cells were stored in TRIzol^®^ Reagent (Life Technologies, Carlsbad, CA, USA). The sub-toxic concentration of APAP used to treat the AML-12 cells was determined by MTT assay using concentrations ranging from 0 to 60 mM and also based on its estimated concentration found in the plasma of individuals who had developed acute hepatic failure due to the intake of APAP [20]. Cells treated with APAP at 1 mM kept their mitochondrial activity unaltered when compared with the untreated cells after 24 h. Thus, 1 mM APAP was established to be used in further assays (data not shown). The permissiveness of AML-12 cells to DENV-2 infection was evaluated by immunocytochemistry as well as by RT-PCR. DENV-2 infected AML-12 cells were positively labeled when an anti-flavivirus recombinant monoclonal antibody was used over a period of 48 h (Supplementary Figure S1). The viral infection was confirmed by the amplification of the viral cDNA by RT-PCR (Supplementary Figure S2). Moreover, the viability of AML-12 cells after treatment with APAP and the cellular damage caused by DENV-2 infection or DENV-2 infection followed by APAP treatment was estimated by measuring the levels of Alanine aminotransferase (ALT) in the AML-12 culture supernatants. ALT was not detected after treatments with APAP (1 mM), DENV-2 (MOI = 1), DENV-2 (MOI = 1) and APAP (1 mM) as well as untreated (data not shown).

### 2.4. RNA Extraction, Library Construction and mRNA Sequencing

The total RNA extraction of AML-12 cells was performed using a TRIzol™ Reagent (Thermo Fisher Scientific, Waltham, MA, USA), following the protocol described by the manufacturer. The concentration was analyzed using a Qubit™ RNA HS Assay Kit (Thermo Fisher Scientific, Waltham, MA, USA) in a Qubit Fluorometer (Thermo Fisher Scientific, Waltham, MA, USA). The purity and integrity of the RNA samples were determined in a Bioanalyzer 2100 system (Agilent Technologies, Santa Clara, CA, USA) using an Agilent RNA 6000 Pico Kit (Agilent Technologies, Santa Clara, CA, USA).

Libraries were generated using a TruSeq Stranded mRNA Library Preparation Kit (Illumina, San Diego, CA, USA). Libraries were quantified using real-time quantitative PCR in addition to the high sensitivity DNA kit and the Agilent 2100 Bioanalyzer System (Agilent Technologies, Santa Clara, CA, USA). Barcoded libraries had their cDNA concentrations adjusted for 4 nM. Sample sequencing was performed using a NextSeq^®^ 500/550 High Output Kit v2 (75 cycles) (Illumina, San Diego, CA, USA) in a NextSeq^®^ 550 Sequencing System (Illumina, San Diego, CA, USA) according to the manufacturer’s protocol. The data were pre-processed using the standard Illumina processing pipeline to segregate multiplexed reads.

### 2.5. Differential Gene Expression Analysis

The initial bioinformatics processing was performed using a Trimmomatic tool [21] to remove low-quality regions from raw reads, specifying a SLIDINGWINDOW 4:15 parameter to remove regions with a quality average below a Phred Quality Score of 15 (Q ≥ 15) at four-base intervals. Reads smaller than 50 bases were excluded. The filtered reads were mapped to the genome of *Mus musculus* version GRCm38.p5, available from Ensembl [22], using the STAR program [23]. The mapping of reads to genes was counted using the multiBamCov function of the BEDTools utilities (v2.28.0). Reads mapped to more than one locus were excluded. Raw reads are available in the SRA database under accession number PRJNA558685.

Differential gene expression was performed using the quasi-likelihood F tests method of the edgeR package (v3.24.3) [24] implemented in R (v3.5.1) [25]. Data were filtered using a HSTFilter package (v1.22.1) [26] and differentially expressed genes (DEGs) were identified using the following thresholds: *p*-value < 0.01 and |logFC| > log_2_(1.5). A multidimensional scaling plot of distances between gene expression profiles was performed using the plotMDS function of the limma package (v3.38.3) [27]. The heatmap was generated using the pheatmap package (v1.0.12) [28].

### 2.6. Gene Ontology (GO) and Pathway Enrichment Analyses

To identify enriched gene ontology (GO) categories [29,30], the functional classification of DEGs was assessed using the enrichGO function from the clusterProfiler package [31] with a Benjamini–Hochberg (BH) p-value adjustment (p.adjust; cutoff < 0.05). The ontologies obtained with the enrichGO were then included in the REVIGO tool to produce non-hierarchical summaries of the enriched GO categories [32]. Dot plots were generated using the ggplot2 package (3.2.0) [33]. The chord plot of enriched GO terms was generated using the GOchord function from the GOplot package (v1.0.2) [34]. MetaCore software (version 20.3 build 70200) from Clarivate Analytics (GeneGo, St. Joseph, MN, USA) was used to identify pathways associated with the gene expression profiles.

## 3. Results

To evaluate the transcriptional changes resulting from the association between the DENV infection and the treatment with sub-toxic APAP concentrations in AML-12 cells, RNAseq data were generated for four experimental groups in three biological replicates involving two variables, DENV infection and APAP treatment, and their respective control groups. These groups generated 12 paired-end libraries yielding a total of 56–70 million paired-end reads per sample. The sequence mapping in the *Mus musculus* reference genome (version GRCm38.p5) resulted in uniquely mapped reads ranging from 80.53% to 84.24%. A wider analysis of the results was performed through a multidimensional scaling plot designed to indicate sample relationship similarities. The multidimensional scaling analysis demonstrated that the infection by DENV-2 was determinant to separate this group of samples from the untreated and APAP treated cells (Supplementary Figure S3).

The treatment of AML-12 cells with APAP 1 mM for a 24-h period compared with the untreated control induced significant changes in the expression levels of 86 genes. Of those, 72 genes were downregulated and 14 genes were upregulated. The infection of AML-12 cells by DENV-2 for a 48-h period, when compared with the untreated control, induced significant changes in the expression levels of 1536 genes, resulting in 466 downregulated and 1070 upregulated genes (Supplementary Table S1).

The data obtained in the differential gene expression analysis were analyzed using GO enrichment analysis, which focused on the biological process categories that analyzed up and downregulated genes separately (Supplementary Table S2).

**Figure 1:**
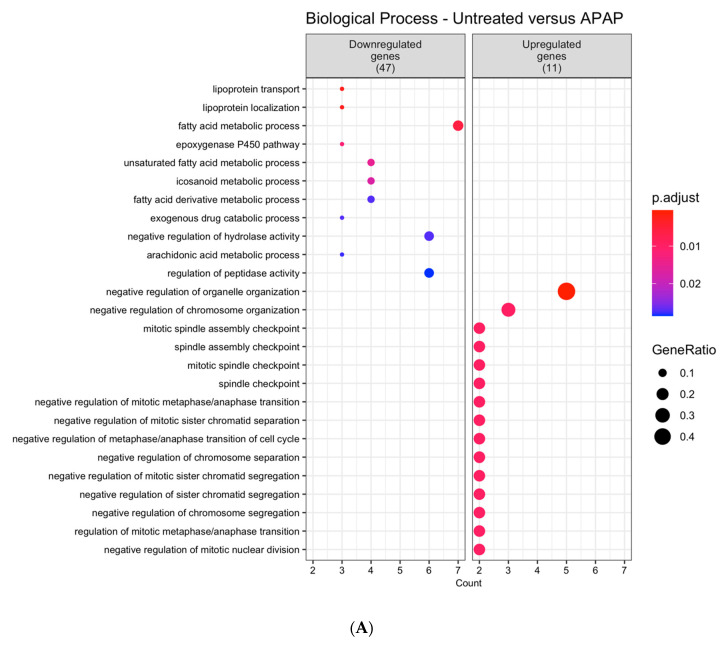

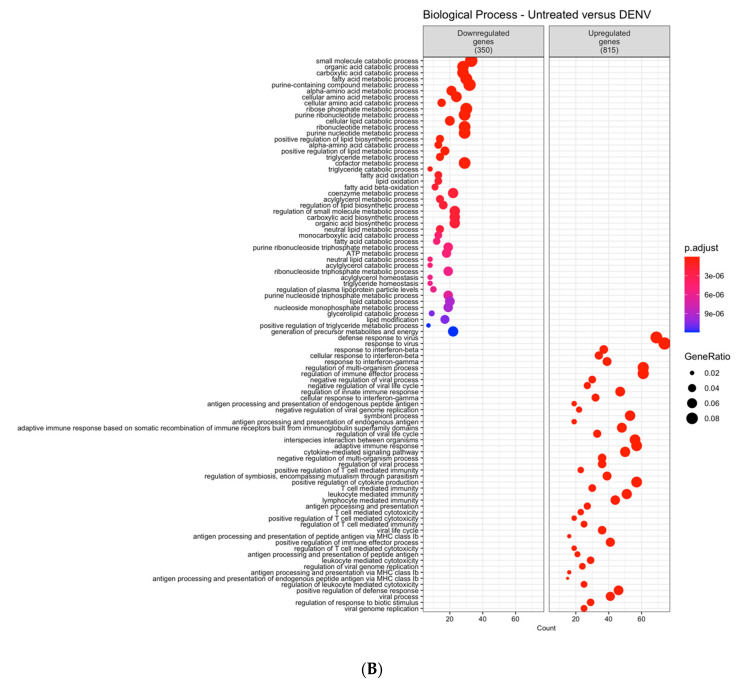
Dot plot of hierarchical summaries of gene ontology (GO) terms enriched in differentially expressed genes in AML-12 hepatocytes treatment with (**A**) APAP or (**B**) infection by DENV-2. Data were obtained from the analysis of the GO terms that were significantly enriched within the biological process categories (p.adjust < 0.05) among differentially expressed genes (DEGs) from each comparison. Dots represent the enriched GO terms after summarization with the REVIGO tool. The color of the dots represents the p-adjust values following Benjamini–Hochberg (BH) significance testing. The position of the dots in the x-axis (Count) is related to the amount of DEGs associated with the GO term. The size of the dots (GeneRatio) represents the number of DEGs related to the number of genes associated with a GO term in the *Mus musculus* genome.

After summarizing the enriched GO terms, the resulting non-redundant terms were ordered according to the uniqueness value. Cells treated with APAP presented seven non-redundant GO terms significantly enriched among the downregulated genes and 17 GO terms among the upregulated genes (Figure 1A). Additionally, for the dataset accounting for the DENV-2 infected cells profile, 46 GO terms among the downregulated genes and 29 GO terms among the upregulated genes were significantly enriched (Figure 1B).

**Figure 2:**
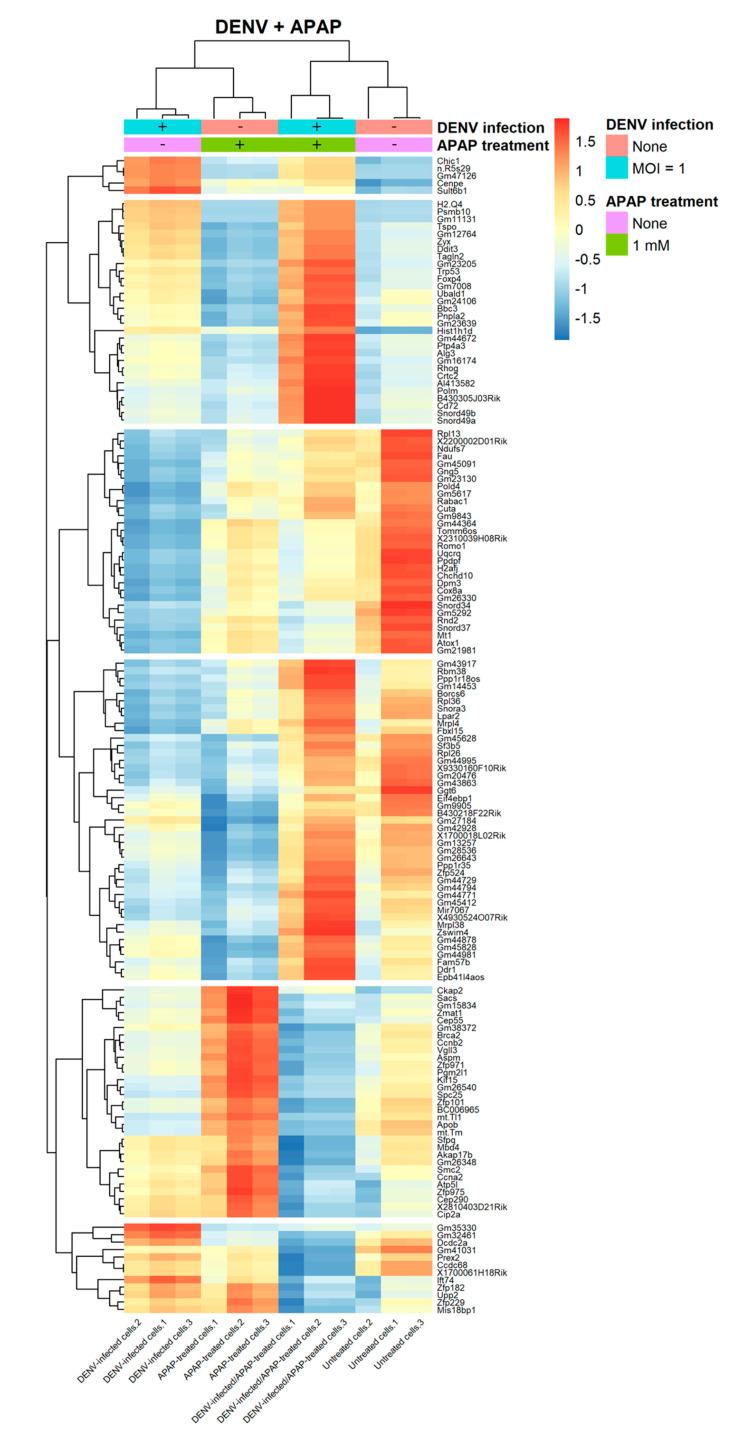
Gene expression patterns of significant DEGs in AML-12 hepatocytes both DENV infected and APAP treated. Hierarchical clustering of the significant 151 genes differentially expressed in AML-12 cells, DENV infected and/or APAP treated AML-12 cells with a *p*-value < 0.01 and fold-change cutoff of log_2_(1.5) (color range from blue to red). Transcripts upregulated are colored red while downregulated are colored blue. Rows represent gene names and columns represent samples. Row sidebar colors indicate the experimental conditions: non-infected cells (orange), DENV infected cells (blue), untreated cells (purple) and APAP treated cells (green).

To visualize the pattern on the transcriptional alterations in response to the interaction of the tested variables, DENV infection and APAP treatment, hierarchical clustering of the DEGs was performed. We found 151 DEGs, of which 49 had downregulated and 102 had upregulated genes. DEGs with similar expression patterns based on the average gene expression values were clustered in six groups of DEGs (Figure 2).

The first cluster consisted of five genes, upregulated when cells were infected with DENV-2. The upregulation induced by DENV-2 infection was reduced when cells infected with DENV-2 were also treated with APAP. The second cluster called attention to the increased expression levels of 30 genes in cells infected with DENV and treated with APAP. In untreated cells and APAP treated cells, these genes were downregulated whereas infection by DENV alone also promoted the positive regulation of these genes. Genes in this cluster were enriched in GO terms associated with 15 biological processes among which we have highlighted the autophagy, regulation of mitochondrion organization, toxin transport, calcium ion transport, positive regulation of intrinsic apoptotic signaling pathway and response to endoplasmic reticulum stress (Supplementary Figure S4A and Supplementary Table S2).

In the third cluster, 30 genes were downregulated in cells infected with DENV. However, in cells infected with DENV and treated with APAP, the presence of the drug appeared to slightly reverse the action of the DENV infection in the expression of these genes. The drug-virus association shifted the expression pattern to that observed in hepatocytes treated with APAP only although the expression still was not the same as that observed in untreated cells.

The fourth cluster contained 43 genes. It was noted that hepatocytes infected with DENV and treated with APAP separately caused a reduction in the expression levels of these genes. However, when DENV and APAP were in association, they induced gene expression levels similar to those presented by untreated cells. This cluster presented genes enriched in GO terms associated with the signal transduction involved in a mitotic DNA damage checkpoint, cytoplasmic translation and signal transduction by a p53 class mediator (Supplementary Figure S4B and Supplementary Table S2).

In the fifth cluster, 31 genes were upregulated in cells treated with APAP. However, when hepatocytes were infected with DENV and treated with APAP, these genes were downregulated. Similar expression patterns in cells infected with DENV and treated with APAP were observed in the sixth cluster, which consisted of 12 genes. However, there was a pronounced upregulation of those genes in cells infected by DENV only. In this cluster, APAP treated and untreated cells presented similar expression patterns. Genes in cluster 5 were enriched in GO terms associated with mitotic cytokinesis and chromosome segregation (Supplementary Figure S4C and Supplementary Table S2).

**Figure 3:**
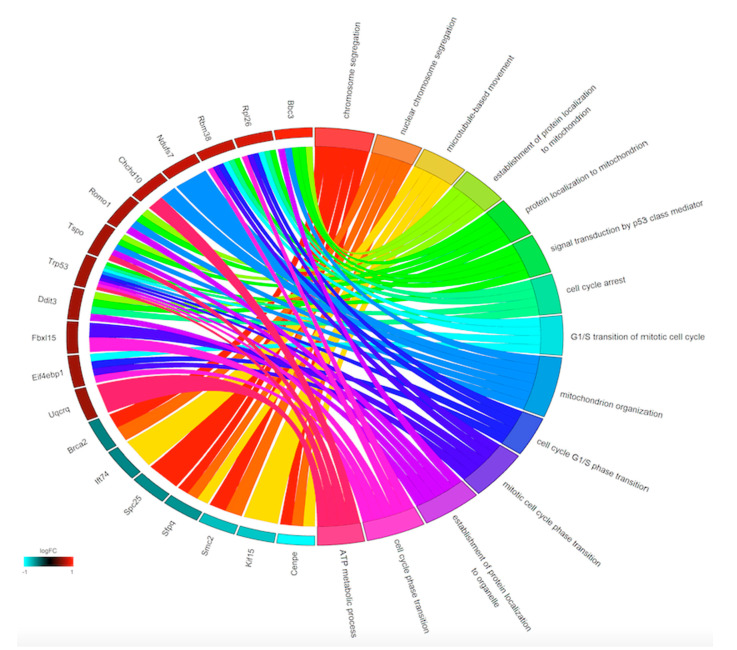
Enriched GO biological process terms identified after DENV infection and APAP treatment. Enriched GO biological processes are shown on the right and differentially expressed genes are shown on the left. On the left side, each gene is represented by a rectangle and the color transition from green to red indicates the log fold change of the expression levels. Upregulated genes are displayed in red whereas downregulated genes are displayed in blue. On the right side, each GO term is represented by a colored rectangle. Chords connect genes (Ensembl IDs) with the biological process (GO terms).

The analyses of the GO terms related to these genes revealed six GO terms significantly enriched by the downregulated genes and 10 GO terms significantly enriched by the upregulated genes (Figure 3).

**Figure 4:**
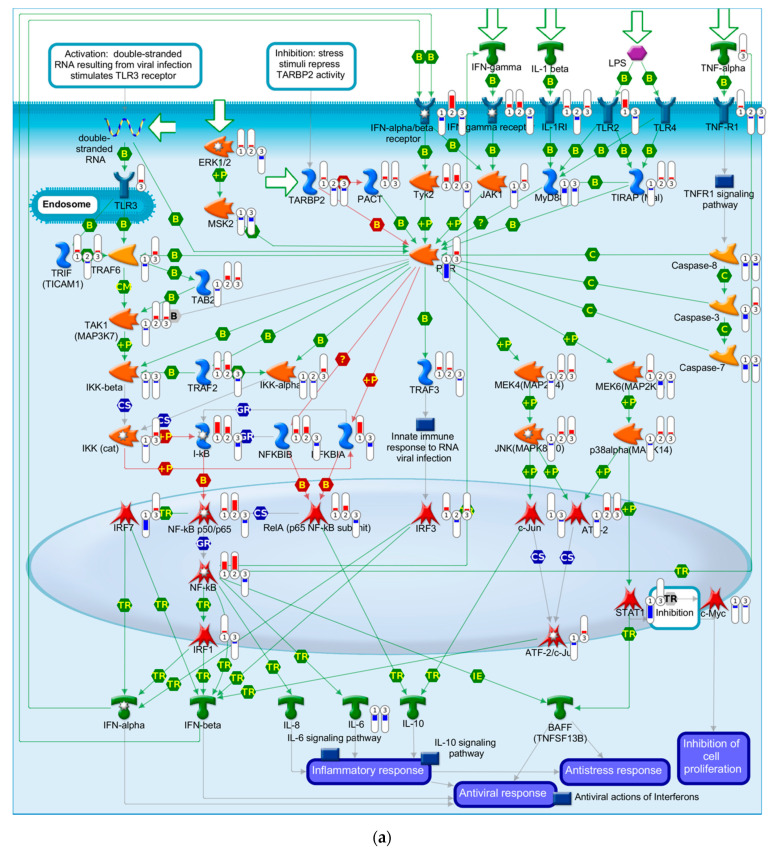

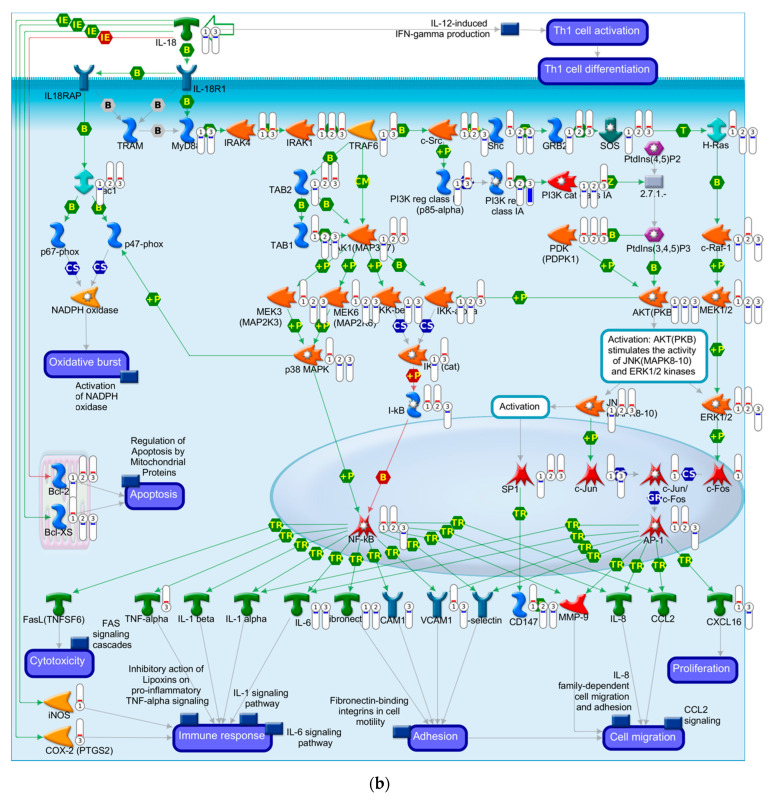
Schematically illustrated MetaCore pathway associated with dengue and liver failure. Differentially expressed genes (DEGs) from cells after DENV-infection and/or APAP-treatment were mapped to the MetaCore pathways: (**a**) “Immune response—Role of Protein Kinase regulated by double-stranded RNA (PKR) in stress-induced antiviral cell response” that is the main canonical pathway associated with dengue; (**b**) “Immune response—Interleukin-18 (IL-18) signaling” that is the main canonical pathway associated with liver failure. The thermometers in the figure indicate the DEGs in cells after DENV-infection and APAP-treatment (1), DENV-infection (2), and APAP-treatment (3). The red columns indicate upregulation and the blue columns indicate downregulation. Further details can be accessed at https://portal.genego.com/help/MC_legend.pdf.

A MetaCore pathway analysis was performed using all DEGs comparing the APAP treatment, DENV infection or DENV infection followed by APAP treatment to the untreated cells. Here, we presented the main pathway associated with Dengue disease (immune response—role of protein kinase regulated by double-stranded RNA (PKR) in stress-induced antiviral cell response (Figure 4a)) and with liver failure (immune response—Interleukin-18 (IL-18) signaling (Figure 4b)).

## 4. Discussion

DENV infection and APAP treatment are capable of triggering severe liver disorders [10,11,35]. APAP is the most commonly used drug to treat dengue symptoms [6] and there are studies showing that dengue patients who use APAP are at increased risk of developing liver failure [14]. Many studies have evaluated the liver damage resulting from the association between DENV and APAP [6,15,36,37] but, despite evidence suggesting that this association may lead to severe hepatic insufficiency, no studies have yet been performed to evaluate cellular and molecular mechanisms involved in hepatocyte response to the association between DENV and APAP. Thus, this study evaluated the transcriptional changes in hepatocytes resulting from the association between DENV infection and APAP treatment and the possible effects of these changes on the molecular and cellular mechanisms that influence cellular homeostasis.

Studies that have elucidated the mechanism of APAP induced hepatotoxicity have significantly contributed to the development of diagnostic and treatment methods for this acute liver failure [38]. However, most of these studies have used APAP doses above the acceptable hepatotoxic dose for an adult human over a 24-h period [39]. Thus, transcriptomic analysis of cells exposed to APAP overdose uniquely elucidates the processes involved in the mechanism of overdose toxicity not taking into account the altered molecular mechanisms at sub-toxic drug concentrations. A study using a mice model demonstrated that the molecular pathways related to liver damage from an APAP overdose are different from those activated in APAP induced idiosyncratic pathogenesis [40]. APAP induced liver injury may be associated with patient-related risk factors (age, chronic diseases, genetics, immune system, metabolism, etc.) and the environment (diet, habits, infections, etc.) [41]. Understanding which of the molecular mechanisms are altered in sub-toxic doses of APAP is of utmost importance for understanding these specific risk factors including the DENV infection.

In this context, the analysis of transcriptomic changes in AML-12 cells resulting from treatment with sub-toxic APAP concentration was performed. The APAP induced liver injury model in mice is suitable for studying metabolic functions or immunological reactions after an APAP overdose and is a directly transferable model for the pathogenesis of human hepatotoxicity [42]. Thus, AML-12 cells were elected as a model to verify the combined effects between treatment with APAP and DENV infection because these cells are transgenic hepatocytes that overexpress the human transforming growth factor alpha (TGFα), capable of metabolizing APAP in a similar way to that observed in human hepatocytes and also susceptible to DENV infection [43,44]. We found genes downregulated that participated in biological processes involved with lipid metabolism. It has been shown that liver damage induced by APAP has important implications for the homeostasis of lipid metabolism and the eicosanoid signaling pathway. A study demonstrated significant changes in the levels of serum transaminases and the profile of free liver fatty acids as well as changes in the gene expression levels of cyclooxygenase, elongase, lipogenesis and lipolysis; important for hepatic lipid metabolism [45]. These data corroborate our findings and suggest that important molecular mechanisms for lipid metabolism in hepatocytes undergo changes induced by APAP.

It was also observed that sub-toxic APAP doses upregulated genes mainly related to the mechanisms of the cell’s multiplication. It is known that, at lower APAP doses, acute liver damage is followed by compensatory liver regeneration. Viable hepatocytes near the injured area undergo mitosis and replace dead cells, promoting tissue recovery [46]. The overexpression of genes that participate in the mitosis process is possibly related to the induction of a mechanism of tissue regeneration.

DENV can infect different cell types causing altered clinical and pathological effects [18]. Among the organs infected by DENV, the liver is commonly the most affected with the hepatocytes and Kupffer cells primary targets of DENV infection [11]. Dengue is usually associated with different degrees of liver damage, with altered levels of biochemical markers, mainly aspartate aminotransferase (AST) and alanine aminotransferase (ALT), being observed at all stages of the disease and even in asymptomatic individuals [35]. The liver damage in dengue patients can be promoted directly by viral toxicity or indirectly by a dysregulated immune response to the virus [11]. A few studies have already assessed hepatic impairment in dengue evaluating the effects of viral infection on the host’s transcriptome [47,48,49].

The changes in the transcriptome in response to DENV can vary depending on different DENV strains and stages of the virus life cycle [47]. In this work, the transcriptional response of AML-12 cells to DENV-2 infection was evaluated, indicating that biological processes enriched by downregulated genes are mostly involved in lipid metabolism and mitochondrial organization. Studies have shown that DENV infection induces the processing of lipid bodies and triglycerides, manipulating lipid metabolism to increase viral production. Autophagy induction is the mechanism by which DENV causes these changes in cellular lipid metabolism, stimulating β-oxidation, which is necessary for viral replication [50,51]. In the autophagy process, membrane structures called phagophores surround the targets and form autophagosomes, which mature and consume their content [52].The process of regulating cellular lipid metabolism through autophagy occurs with the modification of lipid bodies, which act as cellular reserves of triglycerides and cholesterol esters [53]. Triglycerides are hydrolyzed by lipases releasing fatty acids, which are imported into the mitochondria where they undergo β-oxidation for ATP production [54]. DENV infected cells have shown a reduction in the lipid body area and triglyceride levels and increased β-oxidation levels [50,51]. Thus, the negative regulation of genes involved in lipid metabolism in hepatocytes is probably a DENV strategy to reduce the consumption of intracellular lipids by hepatocytes and to provide subsidies to increase viral replication. Accordingly, changes in lipoprotein concentrations in individuals infected with DENV are already described as indicative of liver dysfunction [55].

The downregulation in genes involved in mitochondrial organization corroborates with studies that showed that DENV infection induces mitochondrial and consequently bioenergetic dysfunction, contributing to viral replication [56]. During DENV infection, the NS4B viral protein promotes mitochondrial elongation and the contact of the mitochondrial membrane with the contorted membranes. This process compromises the integrity of the membranes associated with mitochondria, an important site for innate immune signaling, reducing the RIG-I dependent response to DENV [57,58].

In DENV infected AML-12 hepatocytes, upregulated genes are mainly related to antiviral immune responses. The innate immune response against DENV is mainly induced by RIG-I and MDA5 receptors, which recognize dsRNA, detect viral replication in the cytoplasm and constitute an essential part of the innate immune response against the virus [59]. This process triggers the activation of innate immune pathways, which include mainly the synthesis of type I interferon, activate the complement system, apoptosis and autophagy [60].

An epitome of results obtained allows the description that in AML-12 hepatocytes treated with sub-toxic APAP doses, genes involved in the mitosis process were upregulated while in cells infected with DENV, genes involved with the antiviral cellular response were upregulated. However, it is noteworthy that both in DENV infection and in APAP treatment there is a reduction in the expression levels of the genes involved with lipid metabolism. Lipid metabolism is an extremely important mechanism in several processes that guarantee cellular homeostasis [61]. It has been shown that the disturbance of lipid metabolism is directly related to APAP induced liver injury [45] and also with the multiplication of viral particles in DENV infection [50,51].

Lipids are stored mainly as triacylglycerols, cholesterol esters and retinyl esters in organelles composed of a phospholipid monolayer known as lipid droplets. These lipids can be accessed mainly through the lipolysis process, which involves the decomposition of triacylglycerols and esters by cytosolic lipases. Another important process for accessing cellular lipid content is known as lipophagy, a specific subset of selective autophagy that targets lipid droplets, catabolizing their components into free fatty acids and glycerol. Lipophagy is very important to lipid metabolism and the regulation of cellular homeostasis [62], presenting significant pathophysiological implications for the liver. For example, in fatty liver diseases, the positive regulation of free fatty acid levels and lipogenesis induces the accumulation of triglycerides in hepatocytes. In hepatic fibrosis, however, the release of the extracellular content of lipid droplets contributes to the fibrosis process in the liver tissue [63]. As both DENV infection and APAP treatment influence the expression levels of genes involved with lipid metabolism, it is possible that the association between DENV and APAP influences the hepatocytes lipid metabolism and consequently the clinical outcome of dengue patients using APAP.

To verify the effects of the association between the virus and the drug on the transcriptome of AML-12 cells infected with DENV and treated with APAP, the transcriptome of these cells was compared with all other conditions (infected with DENV, treatment with APAP and the untreated cells). Downregulated genes are involved mainly in the mitotic cell cycle and upregulated genes are related to cellular component organization and intracellular signal transduction. Thus, when combined, DENV and APAP seemed to modify pathways that were not individually altered by the virus or the drug. DENV and APAP also acted synergistically, reinforcing the expression patterns of some genes and antagonistically, reversing the expression patterns of other genes. There was a reduction in the expression levels of genes involved with cell division and an increase in the expression of genes related to the apoptosis process, which is important for mitochondrial morphology and cellular bioenergetic mechanisms.

The “Role of PKR in stress-induced antiviral cell response” pathway is the main canonical MetaCore pathway associated with Dengue disease culminating in processes of inflammatory, antiviral and antistress responses besides the inhibition of cell proliferation. The association between DENV and APAP decreased or reversed the expression patterns of several genes in relation to those of DENV infected cells, indicating a possible influence of this association on the responses triggered by the virus in the hepatocytes. Regarding the main MetaCore pathway associated with liver failure, the “IL-18 signaling” pathway is related to Th1 cell activation and differentiation, oxidative burst, apoptosis, cytotoxicity, immune responses, adhesion, cell migration and proliferation. IL-18 mediated signaling is essential for the host’s defense, inflammation and tissue regeneration processes, being related to the mechanisms of liver injury in vivo [64]. The combination of DENV and APAP resulted mainly in an antagonistic regulation of genes involved in this pathway, which could indicate a beneficial outcome for cellular damage in the hepatocytes. It is worth mentioning that our study was limited to a single time point and no independent experiment was performed to confirm the observed expression levels. Therefore, a study contemplating the dynamics of DENV infection may bring a better understanding of the APAP treatment influence on DENV infected hepatocytes.

## 5. Conclusions

Taken together, our results provide an overview of the effects of DENV infection, APAP treatment and the interaction between DENV infection and APAP treatment in the transcriptome profile of AML-12 hepatocytes. Both DENV infection and APAP treatment impact the expression levels of genes involved to lipid metabolism in hepatocytes, a mechanism extremely important for cell homeostasis that should be the target of further studies. It was also observed that the association between DENV and APAP interfered in the expression profile of genes that were molded only by DENV or only by APAP; thus, the joint presence of the virus and the drug led to the alteration of the expression of genes in a way that restored the profiles of expression at levels similar to those of the control (clusters 3 and 4). However, for some genes, the joint action of DENV and APAP made these profiles opposite to those observed in the control (clusters 1, 2, 5 and 6), indicating genes whose expression could act to disrupt the normal cellular metabolism. Investigating the influence of the expression levels of these genes on the cell phenotype may contribute to the understanding of the mechanisms that could induce liver injury during dengue treatment using APAP. Therefore, it may contribute significantly to the safer and efficient management of dengue patients.

## Supplementary Materials

The following are available online at https://www.mdpi.com/1999-4915/12/11/1284/s1, Figure S1: Detection of DENV-2 in AML-12 cell cultures, Figure S2: Evaluation of DENV infection in AML-12 cells by RT-PCR, Figure S3: Multidimensional scaling plot of sequencing libraries of AML-12 cells infected by DENV-2 and/or treated with APAP, Figure S4: Dot plot of hierarchical summaries of GO terms enriched in differentially expressed genes AML-12 hepatocytes after infection by DENV-2 and treatment with APAP in gene clusters 2 (A), 4 (B) and 5 (C), Table S1: Differentially expressed genes identified in AML-12 hepatocytes cells after infection by DENV-2 and/or treatment with APAP, Table S2: Gene ontology/biological process categories significantly enriched for the lists of differentially expressed genes (DEGs) in AML-12 hepatocytes after infection by DENV-2 or treatment with APAP and in gene clusters (2, 4 and 5) in AML-12 hepatocytes after infection by DENV-2 and treatment with APAP. References [65,66] are cited in the supplementary materials.
