# Supplementary material for: Gene Expression Signatures in AML-12 Hepatocyte Cells upon Dengue virus Infection and Acetaminophen Treatment
Source: Viruses. 2020 Nov 10;12(11):1284. doi: 10.3390/v12111284 (PMC7697769; doi:10.3390/v12111284)
Supplement: Supplementary file 1 [file viruses-12-01284-s001.pdf]

viruses

MDPI

Supplementary Materials: Gene Expression Signatures in AML-12 Hepatocyte Cells Upon *Dengue virus* Infection and Acetaminophen Treatment

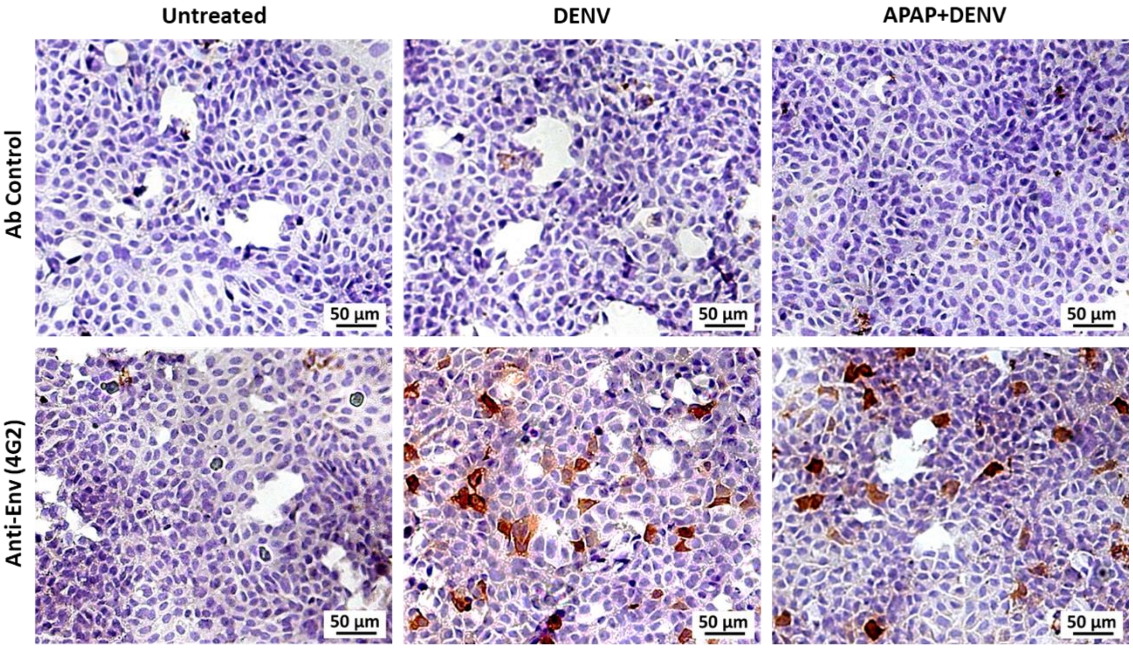

Figure S1. Detection of DENV−2 in AML−12 cell cultures. AML−12 cells were grown in DMEM/Ham's F12. The presence of DENV2 into the cells was performed by immunocytochemistry, using a mouse anti−flavivirus envelope protein antibody (4G2), diluted 1:10, and Novolink™ Polymer Detection Systems. Images are shown at 100X magnification. The brown staining indicates the presence of DENV−2 in the cell cultures. *Ab control:* cells labeled with peroxidase−conjugated antibody. *Anti−Env (4G2):* cells labeled with mouse anti−Flavivirus envelope protein antibody (4G2) and peroxidase−conjugated antibody. *Untreated* = cells not incubated with DENV−2 and untreated with APAP (Negative controls). *DENV* = cells incubated with DENV−2 at a MOI = 1. *APAP + DENV* = cells incubated with DENV−2 at a MOI = 1 (48 h) and 1 mM APAP (24 h).

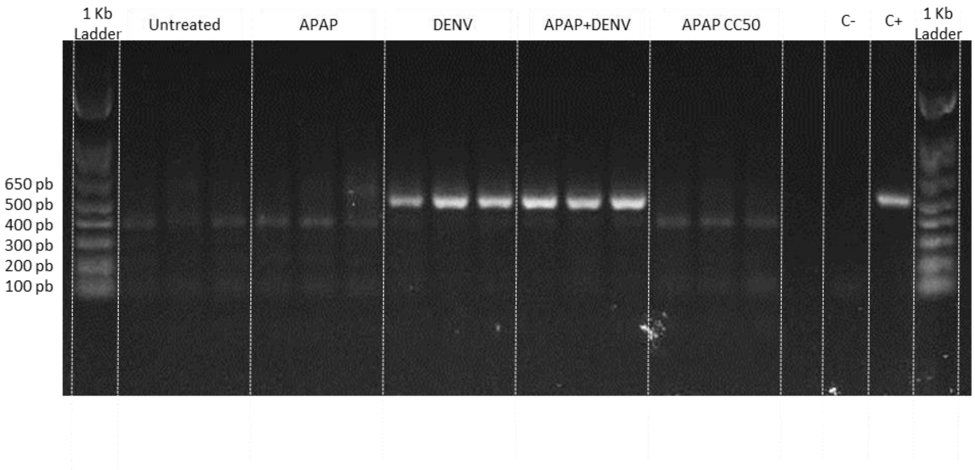

Figure S2. Evaluation of DENV infection in AML−12 cells by RT−PCR. Total RNA extracted from AML−12 cells was used to run RT−PCR. The cDNA was submitted to PCR assays according to [65] modified by [66]. The PCR products were run at 1% agarose/TAE (Tris−acetate, 0,5 mM EDTA) and stained using SYBR Safe—DNA Gel Stain (Thermo Fisher Scientific). The positive control (C+) pUC−

cloning vector, containing a sequence designed for annealing with primers. *(C−)* PCR reaction control (no cDNA). *Untreated* = cultured cells (not incubated with DENV−2 and untreated with APAP. *APAP* = cells treated with APAP 1 mM (24 h). *DENV* = cells incubated with DENV−2 at a MOI = 1 (24 h). *APAP+DENV* = cells incubated with DENV−2 at a MOI = 1 (48 h) and treated with 1 mM APAP (24 h). *APAP CC50*= cells treated with APAP 20 mM (24 h).

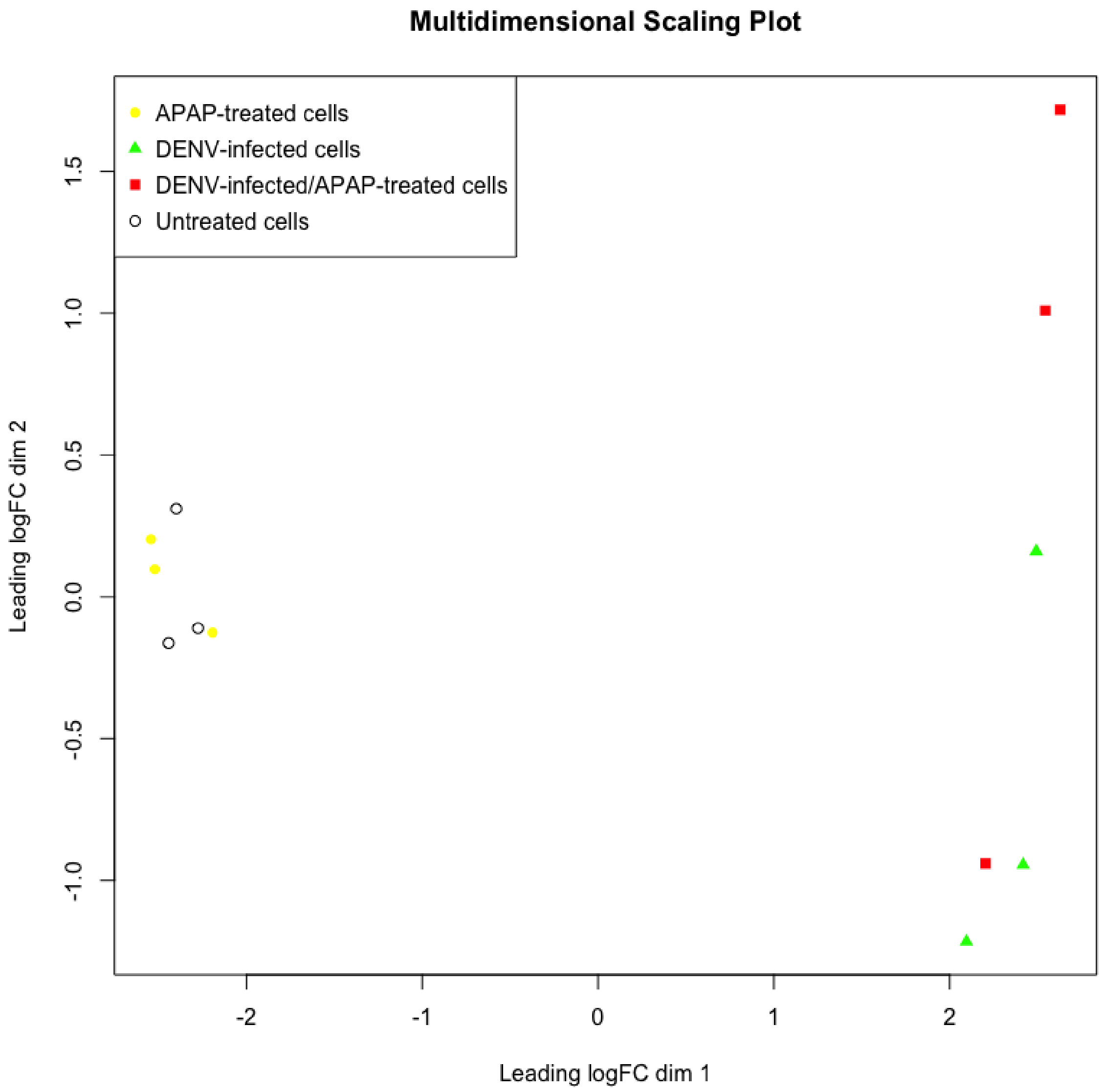

Figure S3. Multidimensional scaling plot of sequencing libraries of AML−12 cells infected by DENV−2 and/or treated with APAP. The multidimensional scaling plot based on pairwise gene expression profiling distances among AML−12 cells infected by DENV−2 and/or treated with APAP. On the x−axis, it is possible to observe the separation of DENV2−infected cells (DENV−infected and DENV−infected/APAP−treated cells) of the non−infected groups (Untreated and APAP−treated cells). DENV−infected cells: cells incubated for 48 hours with DENV−2 (MOI=1); APAP−treated cells: cells treated with 1mM APAP for 24 hours after additional 24 hours of culture; DENV−infected/APAP−treated cells: cells incubated for 24 hours with DENV−2 (MOI=1) and further treated with 1mM APAP for 24 hours; Untreated cells: untreated cells cultured for 48 hours.

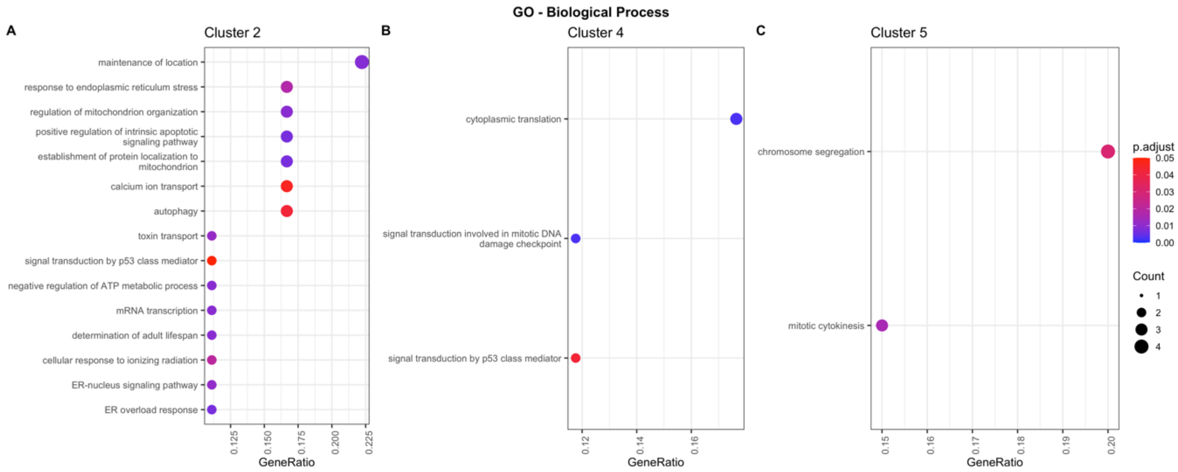

Figure S4. Dot plot of hierarchical summaries of GO terms enriched in differentially expressed genes AML−12 hepatocytes after infection by DENV−2 and treatment with APAP in gene clusters 2 (**A**), 4 (**B**), and 5 (**C**). Data were obtained from the analysis of the GO terms that were significantly enriched within the Biological Process categories (p.adjust < 0.05) among DEGs from each cluster presented in Figure 2. Dots represent the enriched GO terms after summarization with the REVIGO tool. The color of the dots represents the p−adjust values following Benjamini−Hochberg (BH) significance testing. The position of the dots in the x−axis (Count) is related to the amount of DEGs associated with the GO term. The size of the dots (GeneRatio) represents the number of DEGs related to the number of genes associated with a GO term in the Mus musculus genome.

Table S1. Differentially expressed genes identified in AML^{-12} hepatocytes cells after infection by DENV−2 and/or treatment with APAP.

Differentially Expressed Genes in APAP−Treated Cells Compared to Untreated Cells

Gene	logFC	logCPM	F	*p* Value	FDR
Serpinb9b	−0.81	5.00	102.13	2.89×10^{-15}	1.95×10^{-11}
Akr1b7	−0.74	7.09	96.32	9.65×10^{-15}	4.35×10^{-11}
Gm13857	−0.72	6.17	91.88	2.49×10^{-14}	8.44×10^{-11}
Azgp1	−0.66	4.62	72.99	1.97×10^{-12}	5.33×10^{-09}
Eno3	−0.87	5.09	102.17	8.23×10^{-12}	1.86×10^{-08}
Arhgap36	−0.66	4.39	65.53	1.31×10^{-11}	2.52×10^{-08}
Crip1	−0.80	6.01	90.22	5.10×10^{-11}	7.67×10^{-08}
Serpina7	−1.35	1.41	57.90	1.01×10^{-10}	1.37×10^{-07}
Cyp2c68	−0.70	3.67	55.16	2.18×10^{-10}	2.46×10^{-07}
Cyp2c55	−0.76	3.80	59.18	1.68×10^{-09}	1.62×10^{-06}
Gm48702	−0.63	4.78	56.36	2.40×10^{-09}	2.17×10^{-06}
Lamc2	−0.61	3.87	44.17	5.71×10^{-09}	4.16×10^{-06}
Ttc36	−0.62	4.81	53.72	7.92×10^{-09}	5.36×10^{-06}
Spink4	−0.96	2.04	42.81	8.74×10^{-09}	5.63×10^{-06}
Gm36099	−0.88	2.50	40.98	1.57×10^{-08}	8.49×10^{-06}
Wnk2	−1.12	1.40	40.17	2.03×10^{-08}	1.02×10^{-05}
Pax8	−0.60	3.54	39.18	2.81×10^{-08}	1.36×10^{-05}
Fabp5	−0.59	3.65	38.58	3.42×10^{-08}	1.60×10^{-05}
Apol7a	−0.79	3.63	44.62	9.38×10^{-08}	3.73×10^{-05}
Asgr2	−0.67	2.97	35.08	1.10×10^{-07}	3.83×10^{-05}
Apobec1	−0.64	3.72	33.59	1.84×10^{-07}	5.40×10^{-05}
Hsf2bp	−0.59	2.95	29.47	7.90×10^{-07}	1.77×10^{-04}
X2010204K13Rik	−0.93	1.34	29.44	7.97×10^{-07}	1.77×10^{-04}
Ifi27l2b	−1.01	1.81	27.21	1.80×10^{-06}	3.21×10^{-04}
Tekt4	−0.64	2.77	27.16	1.84×10^{-06}	3.23×10^{-04}
Gm42603	−0.64	2.79	26.95	1.99×10^{-06}	3.31×10^{-04}
Orm1	−0.64	2.89	26.91	2.15×10^{-06}	3.51×10^{-04}
Gm6093	−0.98	0.97	26.61	2.26×10^{-06}	3.60×10^{-04}
B230322F03Rik	−0.85	1.87	26.99	2.42×10^{-06}	3.76×10^{-04}
Sorbs3	−0.60	2.72	25.98	2.86×10^{-06}	4.30×10^{-04}
Apof	−1.20	1.94	28.08	3.79×10^{-06}	5.13×10^{-04}
Dbn1	−0.80	1.48	24.21	5.62×10^{-06}	6.93×10^{-04}
Ltbp1	−0.63	2.51	23.44	7.56×10^{-06}	8.05×10^{-04}
Ltbp4	−0.69	3.09	28.57	1.05×10^{-05}	9.60×10^{-04}
Gm27184	−0.81	1.38	22.11	1.27×10^{-05}	1.12×10^{-03}
Gm13257	−0.64	2.44	20.64	2.46×10^{-05}	1.76×10^{-03}
Gm9905	−0.71	2.01	20.50	2.58×10^{-05}	1.82×10^{-03}
Plxnb3	−0.64	2.27	20.20	2.72×10^{-05}	1.88×10^{-03}
BC064078	−0.68	2.11	20.15	2.78×10^{-05}	1.90×10^{-03}
Muc5b	−0.65	2.99	24.69	2.80×10^{-05}	1.91×10^{-03}
Fbp2	−0.59	2.34	19.76	3.26×10^{-05}	2.11×10^{-03}
Hrct1	−0.62	2.22	19.57	3.51×10^{-05}	2.22×10^{-03}
Pparg	−0.68	1.76	19.52	3.59×10^{-05}	2.26×10^{-03}
Cym	−0.84	1.13	19.44	3.72×10^{-05}	2.31×10^{-03}
Apoc2	−0.59	5.09	30.85	4.47×10^{-05}	2.64×10^{-03}
X2310040G24Rik	−0.62	2.59	20.17	4.85×10^{-05}	2.81×10^{-03}
Gm8130	−0.69	2.30	20.20	5.59×10^{-05}	3.06×10^{-03}
A730090N16Rik	−0.65	1.83	18.43	5.61×10^{-05}	3.06×10^{-03}
Gm47577	−0.75	1.38	18.38	5.73×10^{-05}	3.10×10^{-03}
Atp6v0a4	−0.65	1.71	18.13	6.36×10^{-05}	3.33×10^{-03}
Orm3	−0.73	1.71	18.03	6.64×10^{-05}	3.42×10^{-03}
Cbln3	−0.60	4.83	29.63	6.92×10^{-05}	3.45×10^{-03}
Tmprss6	−0.66	4.52	31.54	7.04×10^{-05}	3.47×10^{-03}
Cyp2c65	−0.75	1.27	17.59	7.99×10^{-05}	3.77×10^{-03}
X9130230N09Rik	−0.71	1.56	17.34	8.84×10^{-05}	3.97×10^{-03}
Lmcd1	−0.65	1.88	16.64	1.19×10^{-04}	4.74×10^{-03}
Crb2	−0.73	1.28	16.47	1.28×10^{-04}	4.95×10^{-03}
Ifi204	−0.61	8.23	25.04	1.33×10^{-04}	5.09×10^{-03}
B430218F22Rik	−0.84	1.78	19.46	1.42×10^{-04}	5.33×10^{-03}
Gm47528	−0.61	1.92	15.78	1.72×10^{-04}	6.09×10^{-03}

X4930471E19Rik	−0.67	1.49	15.53	1.92×10^{-04}	6.53×10^{-03}
Tmem236	−0.77	1.39	15.89	2.00×10^{-04}	6.75×10^{-03}
Gm28536	−0.64	2.05	15.98	2.06×10^{-04}	6.88×10^{-03}
Gm36033	−0.74	1.10	15.32	2.09×10^{-04}	6.95×10^{-03}
Gjb4	−0.73	1.81	16.86	2.10×10^{-04}	6.95×10^{-03}
Acnat2	−0.68	1.49	15.06	2.35×10^{-04}	7.49×10^{-03}
Pnmt	−0.62	1.61	15.00	2.41×10^{-04}	7.61×10^{-03}
Dctd	−0.68	1.32	14.80	2.63×10^{-04}	8.14×10^{-03}
Gm35853	−0.64	1.43	14.45	3.07×10^{-04}	9.12×10^{-03}
X1200007C13Rik	−0.90	1.17	16.14	3.14×10^{-04}	9.28×10^{-03}
Hpx	−0.90	7.37	33.48	3.21×10^{-04}	9.39×10^{-03}
Gm19935	−0.64	1.37	14.31	3.26×10^{-04}	9.44×10^{-03}
Hist1h1d	1.54	2.64	131.63	1.13×10^{-17}	1.53×10^{-13}
Mphosph9	0.59	4.27	48.07	2.94×10^{-09}	2.48×10^{-06}
Ckap2	1.10	1.93	45.74	5.84×10^{-09}	4.16×10^{-06}
Casc4	0.66	3.09	36.11	7.78×10^{-08}	3.19×10^{-05}
Cenpe	0.85	2.47	35.07	2.42×10^{-07}	6.85×10^{-05}
Pfn2	0.72	2.39	30.20	6.07×10^{-07}	1.42×10^{-04}
Gm15834	0.62	2.91	27.86	1.42×10^{-06}	2.78×10^{-04}
Gen1	0.93	1.17	26.68	2.20×10^{-06}	3.54×10^{-04}
Nanos1	0.75	2.04	26.35	2.49×10^{-06}	3.83×10^{-04}
Kif15	0.64	2.08	20.03	2.92×10^{-05}	1.95×10^{-03}
Gm47572	0.64	1.71	17.26	9.17×10^{-05}	4.03×10^{-03}
Mybl1	0.63	2.13	17.10	9.80×10^{-05}	4.14×10^{-03}
Gm16042	0.78	1.02	15.76	1.74×10^{-04}	6.10×10^{-03}
Egr1	1.69	6.12	36.88	2.74×10^{-04}	8.35×10^{-03}
**Differentially expressed genes in DENV–infected cells compared to Untreated cells**					
**Gene**	**logFC**	**logCPM**	**F**	**PValue**	**FDR**
Hsd17b4	−0.99	7.29	182.34	4.42×10^{-21}	1.75×10^{-19}
Gnmt	−1.12	4.24	174.07	1.41×10^{-20}	5.49×10^{-19}
Pccb	−0.96	5.56	171.55	2.04×10^{-20}	7.80×10^{-19}
Acot12	−1.42	3.35	180.86	2.30×10^{-20}	8.75×10^{-19}
Kifc2	−1.15	4.20	172.42	2.44×10^{-20}	9.22×10^{-19}
Acad11	−0.92	6.67	158.18	1.49×10^{-19}	5.43×10^{-18}
Uroc1	−1.45	2.97	155.33	2.31×10^{-19}	8.28×10^{-18}
Acaa1b	−1.73	4.83	360.28	5.75×10^{-19}	2.00×10^{-17}
Mturn	−1.30	6.76	251.67	1.07×10^{-18}	3.69×10^{-17}
Ackr4	−0.89	5.60	142.94	1.67×10^{-18}	5.66×10^{-17}
Aldh1a7	−1.08	8.45	179.39	2.56×10^{-18}	8.59×10^{-17}
Tpmt	−1.17	4.45	184.21	1.17×10^{-17}	3.82×10^{-16}
mt.Tl1	−1.03	4.71	155.91	1.21×10^{-17}	3.95×10^{-16}
S100a13	−0.92	4.21	130.99	1.26×10^{-17}	4.08×10^{-16}
Epas1	−1.05	3.87	129.66	1.59×10^{-17}	5.01×10^{-16}
Hist2h2be	−1.10	3.41	128.47	1.96×10^{-17}	6.11×10^{-16}
X4930486I03Rik	−0.90	4.57	126.20	2.94×10^{-17}	9.09×10^{-16}
Cyp2c55	−1.40	3.80	194.10	3.33×10^{-17}	1.03×10^{-15}
Ces2a	−0.96	4.22	126.24	3.70×10^{-17}	1.13×10^{-15}
Dbi	−0.83	8.95	123.37	4.88×10^{-17}	1.48×10^{-15}
Mt1	−0.86	10.05	119.23	1.04×10^{-16}	3.09×10^{-15}
Uqcr11	−0.77	6.09	116.65	1.69×10^{-16}	4.89×10^{-15}
Eif4b	−0.81	9.67	115.51	2.09×10^{-16}	6.00×10^{-15}
Ndufs2	−0.76	7.35	115.30	2.18×10^{-16}	6.24×10^{-15}
Mrpl34	−1.03	3.43	113.95	2.81×10^{-16}	7.96×10^{-15}
Tkfc	−0.76	5.77	112.81	3.49×10^{-16}	9.78×10^{-15}
Klhl13	−0.86	4.35	112.39	3.79×10^{-16}	1.06×10^{-14}
Vat1	−0.73	5.53	110.59	5.37×10^{-16}	1.49×10^{-14}

Matn2	−0.81	4.91	110.49	5.47×10^{-16}	1.51×10^{-14}
Gcdh	−0.74	5.67	110.03	5.98×10^{-16}	1.65×10^{-14}
Cth	−0.82	4.92	109.79	6.27×10^{-16}	1.72×10^{-14}
Sord	−0.82	4.59	109.58	6.53×10^{-16}	1.79×10^{-14}
Cisd3	−0.80	4.60	109.37	6.81×10^{-16}	1.86×10^{-14}
Nop10	−0.78	4.89	109.32	6.87×10^{-16}	1.87×10^{-14}
Abcc6	−1.08	3.19	106.65	1.16×10^{-15}	3.12×10^{-14}
Snord37	−0.79	4.92	106.49	1.20×10^{-15}	3.21×10^{-14}
Apob	−0.81	9.96	105.31	1.52×10^{-15}	4.03×10^{-14}
Fras1	−0.98	3.52	105.29	1.52×10^{-15}	4.04×10^{-14}
Atox1	−0.80	5.94	109.54	1.91×10^{-15}	5.04×10^{-14}
Cmc2	−1.18	2.80	103.41	2.23×10^{-15}	5.82×10^{-14}
Ndufb11	−0.73	7.86	101.70	3.15×10^{-15}	8.17×10^{-14}
Pcca	−0.74	7.61	101.16	3.52×10^{-15}	9.05×10^{-14}
Ttc38	−0.74	6.94	100.86	3.75×10^{-15}	9.62×10^{-14}
Otc	−1.50	3.14	155.20	3.82×10^{-15}	9.78×10^{-14}
Eif3l	−0.72	7.26	100.66	3.91×10^{-15}	9.99×10^{-14}
Cnih1	−0.71	5.51	100.48	4.05×10^{-15}	1.03×10^{-13}
Dhrs1	−0.72	5.25	100.22	4.28×10^{-15}	1.09×10^{-13}
Defb1	−1.38	2.34	100.06	4.42×10^{-15}	1.12×10^{-13}
Sdr42e1	−0.82	4.12	99.45	5.01×10^{-15}	1.26×10^{-13}
Pkhd1	−0.79	8.09	103.34	5.53×10^{-15}	1.38×10^{-13}
Magix	−1.77	1.28	97.24	7.95×10^{-15}	1.96×10^{-13}
Cmbl	−0.71	6.82	97.20	8.01×10^{-15}	1.97×10^{-13}
Npnt	−1.57	5.01	271.29	1.05×10^{-14}	2.55×10^{-13}
Rbp1	−0.74	7.51	95.40	1.17×10^{-14}	2.83×10^{-13}
Abca5	−0.81	4.37	95.35	1.18×10^{-14}	2.85×10^{-13}
Pabpc4	−0.72	4.78	94.28	1.49×10^{-14}	3.53×10^{-13}
L2hgdh	−0.74	4.37	94.28	1.49×10^{-14}	3.53×10^{-13}
Rrm2	−0.74	6.65	95.28	1.54×10^{-14}	3.65×10^{-13}
Entpd5	−0.74	4.98	94.03	1.57×10^{-14}	3.70×10^{-13}
Cox7a2l	−0.70	6.18	93.49	1.76×10^{-14}	4.16×10^{-13}
Nnt	−0.68	6.66	93.19	1.88×10^{-14}	4.41×10^{-13}
Nnt.1	−0.68	6.66	93.19	1.88×10^{-14}	4.41×10^{-13}
Fat1	−0.70	7.12	92.58	2.15×10^{-14}	4.98×10^{-13}
Pgap1	−1.01	3.23	91.96	2.46×10^{-14}	5.68×10^{-13}
Gm42109	−0.75	4.32	91.75	2.57×10^{-14}	5.92×10^{-13}
Slc25a10	−0.82	5.37	106.60	2.77×10^{-14}	6.36×10^{-13}
Ss18l2	−0.83	3.76	90.86	3.12×10^{-14}	7.12×10^{-13}
Ddt	−0.70	5.59	89.86	3.88×10^{-14}	8.82×10^{-13}
Kyat1	−0.76	4.64	89.58	4.13×10^{-14}	9.36×10^{-13}
Micos13	−0.68	6.08	89.06	4.63×10^{-14}	1.05×10^{-12}
Rnd2	−1.04	3.04	88.63	5.10×10^{-14}	1.15×10^{-12}
Cisd1	−0.68	5.56	86.75	7.75×10^{-14}	1.73×10^{-12}
Elovl6	−0.77	4.25	86.20	8.77×10^{-14}	1.95×10^{-12}
Nipsnap1	−0.68	7.13	85.99	9.19×10^{-14}	2.03×10^{-12}
Mien1	−0.69	5.58	85.30	1.07×10^{-13}	2.36×10^{-12}
Ggact	−0.67	5.76	84.36	1.33×10^{-13}	2.91×10^{-12}
Atp5k	−0.67	5.27	84.34	1.34×10^{-13}	2.92×10^{-12}
Scd2	−0.73	10.94	83.89	1.48×10^{-13}	3.23×10^{-12}
Acy1	−0.71	5.09	83.20	1.73×10^{-13}	3.76×10^{-12}
Rogdi	−0.78	4.08	82.79	1.91×10^{-13}	4.13×10^{-12}
Ndrg3	−0.66	5.54	82.76	1.92×10^{-13}	4.14×10^{-12}
Gm42477	−0.78	4.08	82.62	1.99×10^{-13}	4.28×10^{-12}
Afm	−0.78	7.58	96.69	2.15×10^{-13}	4.61×10^{-12}
Gm36738	−0.69	4.55	82.11	2.23×10^{-13}	4.76×10^{-12}

Gcat.1	−0.79	3.95	82.05	2.26×10^{-13}	4.82×10^{-12}
Spp2	−1.40	3.66	158.78	2.35×10^{-13}	4.98×10^{-12}
Pbld2	−0.81	3.86	81.77	2.41×10^{-13}	5.12×10^{-12}
Tmie	−0.89	3.69	84.84	2.52×10^{-13}	5.34×10^{-12}
Acsl1	−0.68	6.83	81.52	2.56×10^{-13}	5.41×10^{-12}
Ppp1r3c	−0.70	4.44	81.47	2.59×10^{-13}	5.47×10^{-12}
Acat2	−0.69	4.54	81.22	2.74×10^{-13}	5.78×10^{-12}
Gcat	−0.78	3.93	80.64	3.14×10^{-13}	6.57×10^{-12}
X2310039H08Rik	−0.73	4.33	80.37	3.35×10^{-13}	6.98×10^{-12}
Ctse	−0.76	5.02	89.71	3.92×10^{-13}	8.11×10^{-12}
Tmem256	−0.70	4.89	79.65	3.96×10^{-13}	8.19×10^{-12}
Gcsh	−0.67	4.80	79.52	4.08×10^{-13}	8.40×10^{-12}
Fmo5	−0.79	7.38	98.42	4.79×10^{-13}	9.82×10^{-12}
Gm12999	−0.65	5.76	78.83	4.81×10^{-13}	9.84×10^{-12}
Sardhos	−0.84	3.97	83.60	5.17×10^{-13}	1.05×10^{-11}
Hebp1	−0.64	5.31	76.78	7.84×10^{-13}	1.58×10^{-11}
Smox	−0.65	5.42	76.46	8.45×10^{-13}	1.69×10^{-11}
Hmgcs2	−0.67	9.44	76.46	8.47×10^{-13}	1.69×10^{-11}
Hyal1	−0.72	4.22	76.36	8.68×10^{-13}	1.73×10^{-11}
Myzap	−0.87	3.67	80.54	8.78×10^{-13}	1.74×10^{-11}
Pcyt2	−0.70	6.71	82.91	8.91×10^{-13}	1.77×10^{-11}
Padi2	−0.91	3.28	76.12	9.18×10^{-13}	1.82×10^{-11}
Polr3g	−0.86	3.39	75.94	9.59×10^{-13}	1.89×10^{-11}
Cox7a1	−0.79	3.91	75.90	9.69×10^{-13}	1.90×10^{-11}
Nudt8	−0.69	4.26	75.41	1.09×10^{-12}	2.13×10^{-11}
Dclk3	−0.85	3.78	79.74	1.18×10^{-12}	2.30×10^{-11}
Atp5g1	−0.67	5.17	75.01	1.20×10^{-12}	2.33×10^{-11}
Ivd	−0.64	6.77	74.46	1.37×10^{-12}	2.66×10^{-11}
Coq8a	−0.69	5.33	78.96	1.64×10^{-12}	3.17×10^{-11}
Tm2d2	−0.64	5.78	73.53	1.72×10^{-12}	3.31×10^{-11}
Phyhd1	−0.61	6.51	73.51	1.73×10^{-12}	3.33×10^{-11}
Tm6sf2	−1.09	2.66	73.44	1.77×10^{-12}	3.38×10^{-11}
Ndufc1	−0.67	5.05	74.17	1.95×10^{-12}	3.72×10^{-11}
C2cd2l	−0.69	4.35	72.87	2.03×10^{-12}	3.86×10^{-11}
Ildr2	−0.65	6.55	74.19	2.28×10^{-12}	4.30×10^{-11}
Sh2b2	−0.96	3.26	78.30	2.45×10^{-12}	4.60×10^{-11}
X0610005C13Rik	−1.40	1.78	72.02	2.51×10^{-12}	4.71×10^{-11}
H2afj	−0.67	5.45	74.74	2.92×10^{-12}	5.44×10^{-11}
Ndufa1	−0.60	5.81	71.09	3.16×10^{-12}	5.86×10^{-11}
Acaa2	−0.63	8.38	70.97	3.25×10^{-12}	6.02×10^{-11}
X4932702P03Rik	−0.79	3.70	70.90	3.31×10^{-12}	6.12×10^{-11}
Gm15788	−0.63	4.66	70.68	3.50×10^{-12}	6.46×10^{-11}
Dqx1	−0.82	3.43	70.42	3.74×10^{-12}	6.89×10^{-11}
Apoe	−0.63	7.88	70.35	3.80×10^{-12}	7.00×10^{-11}
Aldh5a1	−0.74	4.01	69.92	4.24×10^{-12}	7.77×10^{-11}
Aadac	−1.28	1.82	69.86	4.30×10^{-12}	7.86×10^{-11}
Mgat3	−0.82	3.25	69.60	4.59×10^{-12}	8.36×10^{-11}
X2810408A11Rik	−0.94	2.84	69.38	4.86×10^{-12}	8.83×10^{-11}
Hook1	−0.61	5.46	69.36	4.88×10^{-12}	8.86×10^{-11}
Gm21974	−0.65	5.16	69.23	5.05×10^{-12}	9.15×10^{-11}
Mir324	−0.89	3.15	69.14	5.16×10^{-12}	9.33×10^{-11}
Gamt	−0.95	3.62	86.03	5.20×10^{-12}	9.40×10^{-11}
Pcx	−0.65	5.70	71.58	6.31×10^{-12}	1.13×10^{-10}
Mettl26	−0.61	6.19	67.97	6.96×10^{-12}	1.24×10^{-10}
Gpr155	−0.92	2.95	67.52	7.81×10^{-12}	1.39×10^{-10}
Atpif1	−0.59	6.77	67.50	7.84×10^{-12}	1.39×10^{-10}

Syce2	−0.64	4.61	67.06	8.78×10^{-12}	1.55×10^{-10}
Srgap3	−0.79	3.46	66.08	1.13×10^{-11}	1.97×10^{-10}
Acadm	−0.62	8.80	66.05	1.14×10^{-11}	1.99×10^{-10}
Gm16343	−0.63	5.07	65.90	1.19×10^{-11}	2.06×10^{-10}
Vkorc1	−0.63	5.06	65.81	1.21×10^{-11}	2.11×10^{-10}
Adra1a	−1.35	1.47	65.79	1.22×10^{-11}	2.12×10^{-10}
X9430015G10Rik	−0.64	4.21	65.77	1.23×10^{-11}	2.12×10^{-10}
Anxa13	−0.59	7.21	65.77	1.23×10^{-11}	2.12×10^{-10}
Rps17	−0.61	7.90	65.60	1.28×10^{-11}	2.21×10^{-10}
Dcxr	−0.62	4.61	65.50	1.32×10^{-11}	2.26×10^{-10}
Psat1	−0.62	10.64	65.36	1.37×10^{-11}	2.35×10^{-10}
Slc17a5	−0.59	4.88	65.33	1.38×10^{-11}	2.36×10^{-10}
Gm44364	−0.65	4.41	64.68	1.63×10^{-11}	2.77×10^{-10}
Arhgap4	−0.60	4.99	64.54	1.69×10^{-11}	2.87×10^{-10}
Nudt21	−0.64	4.60	64.44	1.73×10^{-11}	2.93×10^{-10}
Naa10	−0.60	4.94	64.15	1.88×10^{-11}	3.15×10^{-10}
Timm17b	−0.68	4.14	63.95	1.97×10^{-11}	3.31×10^{-10}
Agl	−0.60	8.21	63.93	1.99×10^{-11}	3.33×10^{-10}
Tmem184c	−0.63	4.45	63.69	2.12×10^{-11}	3.53×10^{-10}
Rab4a	−0.71	4.20	66.48	2.13×10^{-11}	3.55×10^{-10}
Rbp2	−1.02	8.70	123.79	2.51×10^{-11}	4.14×10^{-10}
Atp5mpl	−0.64	5.46	67.04	3.17×10^{-11}	5.19×10^{-10}
AV356131	−1.42	1.45	62.09	3.24×10^{-11}	5.30×10^{-10}
Nr1i2	−0.62	4.83	62.01	3.31×10^{-11}	5.41×10^{-10}
Entpd8	−0.90	2.89	61.98	3.33×10^{-11}	5.44×10^{-10}
Glud1	−0.59	8.73	61.95	3.36×10^{-11}	5.48×10^{-10}
Bzw2	−0.59	4.96	61.63	3.66×10^{-11}	5.94×10^{-10}
Bcat2	−1.35	1.46	61.15	4.17×10^{-11}	6.74×10^{-10}
Eno3	−0.81	5.09	89.64	4.35×10^{-11}	7.01×10^{-10}
Gm18807	−1.33	1.65	60.98	4.37×10^{-11}	7.02×10^{-10}
Apoa4	−0.93	4.21	94.20	4.40×10^{-11}	7.06×10^{-10}
X9530053A07Rik	−0.82	3.07	60.70	4.71×10^{-11}	7.50×10^{-10}
Sec14l2	−1.21	1.82	59.50	6.52×10^{-11}	1.02×10^{-09}
Reep6	−0.98	4.74	110.08	7.07×10^{-11}	1.10×10^{-09}
P3h4	−0.84	3.09	59.12	7.24×10^{-11}	1.13×10^{-09}
Fbxo2	−0.88	2.95	59.08	7.32×10^{-11}	1.14×10^{-09}
Mttp	−0.69	3.87	59.07	7.35×10^{-11}	1.14×10^{-09}
Shpk	−0.60	4.28	57.85	1.03×10^{-10}	1.57×10^{-09}
Abcb6	−0.63	4.09	57.67	1.08×10^{-10}	1.64×10^{-09}
Lrp1	−0.59	5.06	57.45	1.15×10^{-10}	1.74×10^{-09}
Hacl1	−0.69	3.70	57.38	1.17×10^{-10}	1.77×10^{-09}
Gm49387	−0.69	3.69	56.67	1.43×10^{-10}	2.12×10^{-09}
Il12rb2	−0.97	2.50	56.63	1.44×10^{-10}	2.14×10^{-09}
Fam83b	−0.72	3.45	56.18	1.64×10^{-10}	2.41×10^{-09}
Sardh	−0.80	4.81	82.57	1.89×10^{-10}	2.76×10^{-09}
Dlg2	−1.08	2.11	55.52	1.97×10^{-10}	2.87×10^{-09}
Paxx	−0.71	3.55	55.43	2.02×10^{-10}	2.94×10^{-09}
Cox20	−0.79	3.11	55.13	2.20×10^{-10}	3.19×10^{-09}
Gm10680	−0.92	3.41	71.08	2.34×10^{-10}	3.37×10^{-09}
Alb	−0.87	11.74	86.70	3.83×10^{-10}	5.42×10^{-09}
Pipox	−0.67	6.56	68.52	4.40×10^{-10}	6.17×10^{-09}
Naa80	−0.60	4.75	54.64	4.59×10^{-10}	6.42×10^{-09}
Col7a1	−0.66	3.68	52.41	4.80×10^{-10}	6.71×10^{-09}
Gm49260	−0.89	2.55	52.10	5.24×10^{-10}	7.31×10^{-09}
Spink4	−1.07	2.04	51.99	5.42×10^{-10}	7.53×10^{-09}
X2510046G10Rik	−1.03	2.19	51.57	6.13×10^{-10}	8.43×10^{-09}

Snord83b	−0.64	5.09	61.14	6.16×10^{-10}	8.46×10^{-09}
Soat2	−0.59	4.34	51.30	6.63×10^{-10}	9.06×10^{-09}
Gm44430	−0.83	3.65	64.26	7.34×10^{-10}	9.95×10^{-09}
Slc9a3r2	−0.65	5.11	61.32	9.30×10^{-10}	1.23×10^{-08}
Crat	−0.60	5.62	57.03	1.03×10^{-09}	1.35×10^{-08}
Slc10a5	−1.30	1.08	49.55	1.11×10^{-09}	1.45×10^{-08}
Snapc5	−0.72	3.16	49.32	1.19×10^{-09}	1.55×10^{-08}
Cenpx	−0.64	3.63	48.97	1.32×10^{-09}	1.71×10^{-08}
Chrnb1	−0.64	3.79	48.57	1.49×10^{-09}	1.90×10^{-08}
Gm47528	−1.09	1.92	48.44	1.55×10^{-09}	1.97×10^{-08}
Aqp1	−1.57	3.79	146.12	1.67×10^{-09}	2.11×10^{-08}
Slc25a23	−0.65	6.16	62.66	1.71×10^{-09}	2.15×10^{-08}
Shisa4	−0.68	3.31	48.09	1.72×10^{-09}	2.16×10^{-08}
Ftcd	−0.93	2.49	47.85	1.84×10^{-09}	2.31×10^{-08}
X2810410L24Rik	−0.81	2.79	47.74	1.91×10^{-09}	2.38×10^{-08}
Gfra1	−0.69	3.16	47.35	2.15×10^{-09}	2.66×10^{-08}
Grb14	−0.68	3.29	46.58	2.71×10^{-09}	3.31×10^{-08}
Dlec1	−0.96	2.19	46.32	2.93×10^{-09}	3.55×10^{-08}
Gm13031	−0.86	2.65	46.26	2.99×10^{-09}	3.61×10^{-08}
Nt5dc2	−1.07	4.69	105.73	3.64×10^{-09}	4.35×10^{-08}
Cd36	−1.19	1.57	46.27	3.81×10^{-09}	4.53×10^{-08}
Nme3	−0.68	3.25	45.04	4.35×10^{-09}	5.14×10^{-08}
Cyp2d10	−0.99	2.11	44.74	4.78×10^{-09}	5.58×10^{-08}
Fn3k	−0.65	3.36	44.67	4.88×10^{-09}	5.68×10^{-08}
Sec16b	−0.97	2.06	43.97	6.06×10^{-09}	6.97×10^{-08}
Pank1	−0.71	5.53	66.64	6.09×10^{-09}	6.99×10^{-08}
Gm49259	−0.92	2.26	43.51	7.01×10^{-09}	7.96×10^{-08}
Gmnn	−0.62	3.50	43.01	8.21×10^{-09}	9.20×10^{-08}
Gm14764	−0.68	3.26	42.59	9.36×10^{-09}	1.04×10^{-07}
Snord34	−0.62	5.66	55.68	1.10×10^{-08}	1.21×10^{-07}
Romo1	−0.73	5.20	64.96	1.35×10^{-08}	1.45×10^{-07}
Gm12352	−0.80	4.26	64.88	1.37×10^{-08}	1.48×10^{-07}
Mapt	−0.61	3.76	41.91	1.46×10^{-08}	1.56×10^{-07}
Gm28402	−1.12	1.29	40.61	1.76×10^{-08}	1.87×10^{-07}
Enpp3	−0.96	1.76	40.15	2.05×10^{-08}	2.14×10^{-07}
C030014I23Rik	−0.74	2.72	39.81	2.28×10^{-08}	2.38×10^{-07}
Ugt2b35	−0.77	2.70	39.67	2.39×10^{-08}	2.47×10^{-07}
Cpm	−0.73	2.91	39.61	2.44×10^{-08}	2.52×10^{-07}
Ctnnal1	−0.94	1.89	38.93	3.04×10^{-08}	3.11×10^{-07}
Atp1b1	−0.60	6.88	51.74	3.15×10^{-08}	3.21×10^{-07}
Rdh9	−0.98	1.96	38.76	3.22×10^{-08}	3.27×10^{-07}
Nectin1	−0.62	3.36	38.76	3.22×10^{-08}	3.28×10^{-07}
Myorg	−0.84	2.45	38.65	3.33×10^{-08}	3.38×10^{-07}
Izumo4	−0.72	2.81	38.29	3.76×10^{-08}	3.76×10^{-07}
Dpm3	−0.59	3.59	38.19	3.88×10^{-08}	3.87×10^{-07}
Dab2	−0.79	2.64	38.12	3.97×10^{-08}	3.96×10^{-07}
Apoc1	−0.68	3.72	45.65	4.02×10^{-08}	4.00×10^{-07}
mt.Tm	−0.86	2.38	37.93	4.24×10^{-08}	4.20×10^{-07}
Snrnp25	−0.62	3.15	37.89	4.29×10^{-08}	4.24×10^{-07}
Sucnr1	−2.01	1.28	70.56	4.60×10^{-08}	4.53×10^{-07}
Snora41	−0.60	6.68	50.95	4.72×10^{-08}	4.63×10^{-07}
Gal3st2b	−0.79	2.99	42.49	4.79×10^{-08}	4.69×10^{-07}
Taf13	−0.61	3.29	37.43	5.00×10^{-08}	4.89×10^{-07}
Scrn2	−0.63	3.32	37.42	5.01×10^{-08}	4.90×10^{-07}
Fndc10	−0.74	2.73	37.18	5.42×10^{-08}	5.27×10^{-07}
Cyp17a1	−0.77	2.57	37.16	5.46×10^{-08}	5.30×10^{-07}

Apoc3	−1.08	3.86	79.02	5.56 × 10−08	5.38 × 10−07
Chn2	−0.62	3.13	36.89	5.99 × 10−08	5.76 × 10−07
Gm47204	−0.82	2.29	36.65	6.48 × 10−08	6.21 × 10−07
Hyal2	−0.91	4.32	71.36	6.55 × 10−08	6.27 × 10−07
Aldob	−0.60	9.11	48.08	7.15 × 10−08	6.81 × 10−07
Fbp2	−0.81	2.34	36.11	7.79 × 10−08	7.38 × 10−07
Nrp1	−0.89	1.99	36.07	7.88 × 10−08	7.45 × 10−07
Gm22574	−0.70	3.56	43.38	8.36 × 10−08	7.88 × 10−07
Tmem125	−0.93	1.86	35.88	8.41 × 10−08	7.92 × 10−07
Cpn2	−0.93	1.84	35.80	8.64 × 10−08	8.10 × 10−07
Clic6	−1.15	2.16	46.46	8.81 × 10−08	8.25 × 10−07
Gm5292	−1.16	1.09	35.16	1.07 × 10−07	9.86 × 10−07
B230217O12Rik	−0.82	2.21	35.09	1.10 × 10−07	1.01 × 10−06
Apoc2	−0.96	5.09	80.54	1.28 × 10−07	1.17 × 10−06
Gm26372	−0.67	2.96	34.56	1.31 × 10−07	1.19 × 10−06
Gm10501	−0.78	2.49	34.29	1.44 × 10−07	1.30 × 10−06
Plin5	−1.05	4.69	85.75	1.45 × 10−07	1.30 × 10−06
Dbn1	−0.96	1.48	34.28	1.45 × 10−07	1.30 × 10−06
Stmn1	−0.84	2.32	34.24	1.47 × 10−07	1.32 × 10−06
Serpina1a	−0.82	2.37	34.14	1.52 × 10−07	1.36 × 10−06
Cyp4f15	−0.82	2.42	34.23	1.59 × 10−07	1.42 × 10−06
Gm17251	−0.79	2.33	33.73	1.75 × 10−07	1.55 × 10−06
Grk4	−0.90	1.79	33.55	1.87 × 10−07	1.64 × 10−06
Gm43189	−0.68	3.09	35.23	2.14 × 10−07	1.86 × 10−06
Gm4032	−0.92	1.77	33.12	2.16 × 10−07	1.88 × 10−06
Gm44805	−0.91	5.30	74.49	2.26 × 10−07	1.95 × 10−06
Cadm4	−0.86	1.95	32.86	2.37 × 10−07	2.04 × 10−06
D830044D21Rik	−0.85	1.86	32.75	2.46 × 10−07	2.11 × 10−06
BC028777	−0.63	2.89	32.11	3.09 × 10−07	2.58 × 10−06
Rdh16f1	−0.83	2.22	32.02	3.17 × 10−07	2.66 × 10−06
Col27a1	−0.61	8.25	45.97	3.22 × 10−07	2.70 × 10−06
Plekhg6	−0.87	2.09	31.82	3.41 × 10−07	2.84 × 10−06
Dnajc28	−0.62	3.35	33.90	3.42 × 10−07	2.84 × 10−06
Ctxn1	−0.80	2.33	31.68	3.58 × 10−07	2.96 × 10−06
Pcsk4	−0.74	2.49	31.63	3.65 × 10−07	3.02 × 10−06
Serpinf1	−0.90	1.82	31.55	3.75 × 10−07	3.09 × 10−06
X5330438I03Rik	−0.70	2.69	31.45	3.88 × 10−07	3.19 × 10−06
Tedc2	−0.62	2.87	31.37	4.00 × 10−07	3.27 × 10−06
X1700003F12Rik	−0.93	1.50	31.31	4.08 × 10−07	3.33 × 10−06
Gm21981	−0.69	4.92	50.64	4.12 × 10−07	3.36 × 10−06
Cyp2d26	−0.75	2.31	31.05	4.48 × 10−07	3.63 × 10−06
Slc22a30	−1.01	1.31	30.82	4.87 × 10−07	3.91 × 10−06
Slc9a9	−0.60	3.25	31.16	5.05 × 10−07	4.05 × 10−06
Ropn1l	−0.68	2.64	30.65	5.17 × 10−07	4.13 × 10−06
Cyp2c67	−0.84	1.69	30.15	6.18 × 10−07	4.87 × 10−06
Sort1	−0.81	1.95	30.01	6.49 × 10−07	5.10 × 10−06
Gm15775	−0.60	2.98	29.94	6.66 × 10−07	5.22 × 10−06
Fancm	−0.60	2.95	29.93	6.68 × 10−07	5.23 × 10−06
Akr7a5	−0.92	5.03	67.39	7.53 × 10−07	5.83 × 10−06
Dmpk	−1.00	4.18	66.17	7.64 × 10−07	5.91 × 10−06
Pcyox1l	−0.62	3.31	32.51	7.77 × 10−07	6.00 × 10−06
Mir7046	−0.76	2.29	29.33	8.31 × 10−07	6.39 × 10−06
Gm10039	−0.67	3.99	40.62	8.38 × 10−07	6.43 × 10−06
X2500004C02Rik	−0.77	2.37	29.23	8.60 × 10−07	6.58 × 10−06
Mir6397	−0.71	2.46	28.72	1.04 × 10−06	7.79 × 10−06
Gm49188	−0.66	2.61	28.53	1.11 × 10−06	8.26 × 10−06

Mrps21	−0.62	4.54	41.22	1.12 × 10^{-06}	8.32 × 10^{-06}
X9330102E08Rik	−0.72	2.36	28.43	1.15 × 10^{-06}	8.54 × 10^{-06}
Tmem38a	−0.74	2.33	28.21	1.25 × 10^{-06}	9.16 × 10^{-06}
X4930480K23Rik	−0.75	2.16	28.17	1.27 × 10^{-06}	9.29 × 10^{-06}
Cbln3	−0.85	4.83	59.22	1.43 × 10^{-06}	1.03 × 10^{-05}
X9130019P16Rik	−0.62	2.70	27.62	1.55 × 10^{-06}	1.11 × 10^{-05}
Gm44816	−0.80	2.09	27.52	1.61 × 10^{-06}	1.16 × 10^{-05}
Ihh	−0.72	2.38	27.46	1.65 × 10^{-06}	1.18 × 10^{-05}
Necab3	−0.82	1.70	27.39	1.69 × 10^{-06}	1.21 × 10^{-05}
Gm37113	−1.40	4.88	99.77	1.76 × 10^{-06}	1.26 × 10^{-05}
Kank1	−0.71	2.99	32.19	1.82 × 10^{-06}	1.29 × 10^{-05}
Gm43691	−0.81	1.76	27.12	1.87 × 10^{-06}	1.32 × 10^{-05}
Fam81a	−0.78	2.04	26.82	2.09 × 10^{-06}	1.47 × 10^{-05}
Rab26os	−0.83	1.57	26.52	2.34 × 10^{-06}	1.62 × 10^{-05}
Gpt	−0.94	5.68	65.91	2.36 × 10^{-06}	1.64 × 10^{-05}
Tomm6os	−0.79	3.83	43.36	2.64 × 10^{-06}	1.81 × 10^{-05}
Sds	−0.85	1.51	26.15	2.69 × 10^{-06}	1.84 × 10^{-05}
mt.Cytb	−1.96	12.12	122.84	2.69 × 10^{-06}	1.84 × 10^{-05}
Suox	−0.74	3.27	35.75	2.79 × 10^{-06}	1.91 × 10^{-05}
Rpusd3	−0.62	2.62	25.89	2.96 × 10^{-06}	2.01 × 10^{-05}
Cda	−0.94	1.17	25.82	3.04 × 10^{-06}	2.06 × 10^{-05}
Camsap3	−0.59	3.00	25.92	3.14 × 10^{-06}	2.12 × 10^{-05}
Gm9512	−0.87	1.40	25.61	3.29 × 10^{-06}	2.21 × 10^{-05}
Cops9	−0.73	5.46	48.60	3.42 × 10^{-06}	2.29 × 10^{-05}
Itga7	−0.85	1.57	25.42	3.54 × 10^{-06}	2.36 × 10^{-05}
Scnn1a	−0.61	3.24	28.87	3.73 × 10^{-06}	2.47 × 10^{-05}
Nradd	−0.76	2.26	25.84	3.86 × 10^{-06}	2.55 × 10^{-05}
Tomm6	−0.68	4.85	43.17	3.92 × 10^{-06}	2.58 × 10^{-05}
Ndrg2	−0.59	6.28	38.92	4.14 × 10^{-06}	2.72 × 10^{-05}
Chchd10	−0.67	3.77	34.97	4.37 × 10^{-06}	2.86 × 10^{-05}
Ddit4l	−0.74	2.11	24.58	4.87 × 10^{-06}	3.15 × 10^{-05}
Gm10010	−0.75	1.82	24.48	5.06 × 10^{-06}	3.26 × 10^{-05}
Pbld1	−0.74	2.02	24.44	5.13 × 10^{-06}	3.30 × 10^{-05}
Fgf11	−0.81	1.85	24.50	5.15 × 10^{-06}	3.32 × 10^{-05}
Fgf1	−0.60	6.68	38.71	5.29 × 10^{-06}	3.39 × 10^{-05}
Gm8130	−0.81	2.30	27.14	5.68 × 10^{-06}	3.63 × 10^{-05}
Ass1	−0.80	5.22	49.76	6.30 × 10^{-06}	3.99 × 10^{-05}
Gm18609	−0.73	1.86	23.65	6.97 × 10^{-06}	4.38 × 10^{-05}
Gm25788	−0.74	1.87	23.58	7.14 × 10^{-06}	4.48 × 10^{-05}
Wdhd1	−0.59	2.71	23.55	7.23 × 10^{-06}	4.53 × 10^{-05}
Gm10644	−0.59	2.78	23.53	7.28 × 10^{-06}	4.56 × 10^{-05}
Tmem45a	−0.65	2.36	23.25	8.12 × 10^{-06}	5.03 × 10^{-05}
E130208F15Rik	−0.63	3.16	27.37	8.17 × 10^{-06}	5.05 × 10^{-05}
Pnmt	−0.77	1.61	23.23	8.19 × 10^{-06}	5.06 × 10^{-05}
Ankrd9	−0.69	2.27	23.12	8.56 × 10^{-06}	5.27 × 10^{-05}
Hspa1l	−0.82	1.50	22.79	9.73 × 10^{-06}	5.90 × 10^{-05}
A730063M14Rik	−0.65	2.38	22.68	1.01 × 10^{-05}	6.13 × 10^{-05}
Cd63.ps	−0.66	2.34	22.57	1.06 × 10^{-05}	6.39 × 10^{-05}
Gm14372	−0.65	2.42	22.56	1.06 × 10^{-05}	6.40 × 10^{-05}
Endou	−0.76	1.73	22.52	1.08 × 10^{-05}	6.49 × 10^{-05}
Hykk	−0.84	1.37	22.50	1.09 × 10^{-05}	6.52 × 10^{-05}
Cracr2b	−0.69	1.97	22.44	1.12 × 10^{-05}	6.68 × 10^{-05}
Hnf4aos	−0.82	1.55	22.40	1.18 × 10^{-05}	7.00 × 10^{-05}
Itih3	−0.68	2.10	22.23	1.21 × 10^{-05}	7.15 × 10^{-05}
Ptrh1	−0.61	2.47	22.16	1.25 × 10^{-05}	7.33 × 10^{-05}
Gm47163	−0.60	2.59	22.14	1.26 × 10^{-05}	7.40 × 10^{-05}

Fabp1	−1.76	5.10	86.12	1.26 × 10^{-05}	7.43 × 10^{-05}
Dbndd1	−0.64	2.45	21.73	1.48 × 10^{-05}	8.59 × 10^{-05}
mt.Nd4	−1.35	10.42	73.29	1.50 × 10^{-05}	8.74 × 10^{-05}
Gm2366	−0.66	2.14	21.57	1.57 × 10^{-05}	9.08 × 10^{-05}
Rap1gapos	−0.67	2.23	21.50	1.62 × 10^{-05}	9.34 × 10^{-05}
Tmc4	−0.71	1.78	21.49	1.63 × 10^{-05}	9.37 × 10^{-05}
Slc25a34	−1.07	2.58	35.63	1.70 × 10^{-05}	9.75 × 10^{-05}
Adam12	−0.66	2.06	21.32	1.74 × 10^{-05}	9.93 × 10^{-05}
Tmprss11f	−0.63	2.36	21.16	1.85 × 10^{-05}	0.0001
Cyp3a11	−0.79	1.47	20.89	2.06 × 10^{-05}	0.0001
Apoc4	−0.63	2.47	21.09	2.10 × 10^{-05}	0.0001
Gm45091	−0.84	1.19	20.74	2.19 × 10^{-05}	0.0001
Prodh2	−1.08	6.38	60.55	2.20 × 10^{-05}	0.0001
Rab26	−0.70	1.76	20.67	2.25 × 10^{-05}	0.0001
Rfc4	−0.59	2.41	20.37	2.55 × 10^{-05}	0.0001
Mcmdc2	−0.69	1.78	20.31	2.61 × 10^{-05}	0.0001
Rab3il1	−0.85	2.29	25.21	2.62 × 10^{-05}	0.0001
Gm44280	−0.63	2.10	20.10	2.83 × 10^{-05}	0.0002
X1810044D09Rik	−0.76	1.48	19.99	2.97 × 10^{-05}	0.0002
Mir1960	−0.64	2.12	19.87	3.12 × 10^{-05}	0.0002
Krt10	−0.71	1.62	19.83	3.17 × 10^{-05}	0.0002
mt.Tt	−1.42	2.31	39.86	3.21 × 10^{-05}	0.0002
Gm11266	−0.67	2.05	19.79	3.22 × 10^{-05}	0.0002
Fndc4	−0.64	2.49	20.69	3.24 × 10^{-05}	0.0002
Gm44704	−0.67	1.81	19.67	3.38 × 10^{-05}	0.0002
Vash2	−0.66	1.98	19.58	3.51 × 10^{-05}	0.0002
Gm35853	−0.75	1.43	19.55	3.55 × 10^{-05}	0.0002
Pfn2	−0.59	2.39	19.26	3.99 × 10^{-05}	0.0002
Pxmp2	−0.90	4.48	44.58	4.00 × 10^{-05}	0.0002
Metrn	−0.77	2.02	21.19	4.28 × 10^{-05}	0.0002
Cyp3a16	−0.72	1.49	19.07	4.32 × 10^{-05}	0.0002
Lbh	−0.69	1.89	19.06	4.34 × 10^{-05}	0.0002
Gm14767	−0.73	1.41	18.87	4.68 × 10^{-05}	0.0002
Tnfaip8l1	−0.64	1.93	18.84	4.74 × 10^{-05}	0.0002
Apoa1	−0.79	10.94	38.84	5.04 × 10^{-05}	0.0003
X1700042O10Rik	−0.67	2.03	18.74	5.08 × 10^{-05}	0.0003
Gm23547	−0.59	2.44	18.61	5.21 × 10^{-05}	0.0003
Sdhaf1	−0.60	3.85	25.96	5.54 × 10^{-05}	0.0003
Mospd3	−0.59	2.45	18.34	5.91 × 10^{-05}	0.0003
Plcb1	−0.69	1.77	18.30	5.93 × 10^{-05}	0.0003
Ajm1	−0.68	1.79	18.08	6.51 × 10^{-05}	0.0003
Mir6900	−0.62	2.01	17.93	6.91 × 10^{-05}	0.0003
G0s2	−0.70	4.52	33.38	6.93 × 10^{-05}	0.0003
Extl1	−0.64	1.85	17.52	8.23 × 10^{-05}	0.0004
Cyp4a10	−0.67	1.61	17.42	8.55 × 10^{-05}	0.0004
Gm26702	−0.67	1.79	17.30	9.02 × 10^{-05}	0.0004
Gm12043	−0.62	2.16	17.17	9.51 × 10^{-05}	0.0005
Sept1	−0.72	1.28	17.13	9.67 × 10^{-05}	0.0005
Gm17168	−0.64	5.30	30.29	0.0001	0.0005
A330069K06Rik	−0.70	1.94	17.79	0.0001	0.0006
Cand2	−0.74	5.69	34.16	0.0001	0.0006
Tmem25	−0.64	3.21	22.16	0.0001	0.0006
Zfp457	−0.63	1.86	16.30	0.0001	0.0006
Pde4d	−0.61	1.85	16.28	0.0001	0.0006
C630043F03Rik	−0.68	1.39	16.20	0.0001	0.0007
Efcab11	−0.59	2.00	16.09	0.0002	0.0007

Gm28151	−0.68	1.43	15.86	0.0002	0.0008
Ppp2r2b	−0.73	1.31	15.83	0.0002	0.0008
Csdc2	−0.66	2.48	18.16	0.0002	0.0008
Dancr	−0.63	1.74	15.64	0.0002	0.0008
Gckr	−0.61	1.60	15.34	0.0002	0.0009
Ddc	−0.66	3.88	24.72	0.0002	0.0009
Car1	−0.98	1.70	20.70	0.0002	0.0010
Gm6598	−0.68	1.29	15.14	0.0002	0.0010
Gm14226	−0.63	1.56	15.13	0.0002	0.0010
Gm6297	−0.60	1.71	15.12	0.0002	0.0010
X9630028H03Rik	−0.68	1.27	14.97	0.0002	0.0011
Rgn	−0.98	3.64	31.27	0.0003	0.0012
Postn	−0.69	5.61	28.52	0.0003	0.0013
Gm42984	−0.59	1.84	14.33	0.0003	0.0013
Milr1	−0.60	1.52	14.18	0.0003	0.0014
Fzd4	−0.63	5.85	26.03	0.0004	0.0015
Rps10.ps2	−0.83	1.44	16.39	0.0004	0.0015
Serpinf2	−0.74	5.88	27.32	0.0005	0.0021
Cyp2c65	−0.65	1.27	13.21	0.0005	0.0021
Hnf4a	−0.62	7.76	23.62	0.0006	0.0022
X1500026H17Rik	−0.63	1.19	12.68	0.0007	0.0026
Slc38a3	−0.70	6.66	24.76	0.0007	0.0028
Gm45767	−0.61	1.29	12.39	0.0008	0.0029
Foxo6	−0.60	1.92	12.95	0.0008	0.0029
Scarf1	−0.70	1.68	13.77	0.0008	0.0031
D030055H07Rik	−0.86	4.05	23.98	0.0010	0.0035
X1810019D21Rik	−1.23	3.15	24.94	0.0010	0.0036
D930028M14Rik	−0.74	2.62	16.62	0.0010	0.0037
S100b	−0.82	1.25	13.65	0.0012	0.0042
Glyctk	−0.87	4.04	22.61	0.0012	0.0043
Gm3222	−0.64	1.03	11.38	0.0012	0.0043
Rpl7a.ps12	−0.71	3.74	19.42	0.0012	0.0044
Pou2f2	−0.73	2.66	15.90	0.0013	0.0045
Gm6472	−0.71	3.74	19.02	0.0014	0.0048
Gm12258	−0.59	3.05	14.81	0.0014	0.0049
Gm22980	−0.63	6.34	20.18	0.0015	0.0050
Nrep	−1.89	2.54	21.84	0.0015	0.0052
Mir5114	−0.79	5.65	20.84	0.0017	0.0058
Tm4sf20	−0.60	2.97	14.17	0.0018	0.0060
Brca1	−0.69	4.23	18.55	0.0020	0.0065
Gm25745	−0.61	2.24	11.82	0.0022	0.0072
Hr	−0.62	2.04	11.23	0.0026	0.0083
mt.Nd6	−0.96	10.11	17.95	0.0027	0.0085
mt.Nd5	−1.09	11.62	17.91	0.0027	0.0086
Gm48914	−0.60	0.90	9.64	0.0028	0.0086
Gm47467	−0.67	1.25	10.42	0.0028	0.0087
Gm12185	8.36	8.29	6214.98	1.50×10^{-69}	1.58×10^{-65}
Tgtp1	8.50	8.24	6116.57	2.59×10^{-69}	1.58×10^{-65}
Olfr56	8.19	8.34	6062.34	3.51×10^{-69}	1.58×10^{-65}
Oas3	8.25	8.42	5857.89	1.14×10^{-68}	3.85×10^{-65}
Mx2	7.32	7.92	5465.99	1.22×10^{-68}	2.83×10^{-64}
Irf7	7.92	8.95	5460.69	1.26×10^{-68}	2.83×10^{-64}
Usp18	7.31	8.50	5294.58	3.62×10^{-68}	6.99×10^{-64}
Gm44148	7.27	8.27	5032.83	2.05 × 10−66	3.46×10^{-63}
Psmb8	6.31	6.81	4664.74	2.74×10^{-65}	4.12×10^{-62}
Stat1	6.17	9.78	4614.69	3.96×10^{-65}	5.35×10^{-62}

Gm28177	6.26	9.50	4523.51	7.82×10^{-65}	9.62×10^{-62}
Ifitm3	8.38	7.29	5568.54	1.30×10^{-64}	1.44×10^{-61}
Gm20496	6.12	6.69	4448.25	1.39×10^{-64}	1.44×10^{-61}
Dhx58	6.29	6.93	4343.29	3.13×10^{-64}	3.02×10^{-61}
Ifi211	6.33	5.99	4249.76	6.57×10^{-64}	5.93×10^{-61}
Ifi202b	6.44	9.50	4204.76	9.45×10^{-64}	7.99×10^{-61}
Gm43802	8.29	4.65	4100.53	2.22×10^{-63}	1.77×10^{-60}
Irgm2	5.62	9.22	3545.20	3.14×10^{-61}	2.36×10^{-58}
Tor3a	5.04	7.89	3278.56	4.47×10^{-60}	3.18×10^{-57}
Ifih1	4.84	7.27	3158.70	1.58×10^{-59}	1.07×10^{-56}
Parp12	4.70	7.50	3110.58	2.66×10^{-59}	1.71×10^{-56}
Parp14	4.99	7.97	3069.20	4.19×10^{-59}	2.58×10^{-56}
Psmb9	4.58	5.66	2907.41	2.62×10^{-58}	1.54×10^{-55}
Dtx3l	4.30	7.69	2805.22	8.79×10^{-58}	4.96×10^{-55}
X9930111J21Rik2	7.89	3.64	2798.88	9.49×10^{-58}	5.14×10^{-55}
Tap1	4.69	5.94	2777.08	1.24×10^{-57}	6.43×10^{-55}
Bst2	4.57	8.39	2771.48	1.32×10^{-57}	6.63×10^{-55}
Gm45193	4.56	7.49	2761.10	1.50×10^{-57}	7.26×10^{-55}
Tap2	4.64	6.76	2734.16	2.09×10^{-57}	9.76×10^{-55}
Dhx58os	6.22	5.45	3607.56	3.42×10^{-57}	1.54×10^{-54}
Gm5431	7.72	3.56	2603.20	1.10×10^{-56}	4.79×10^{-54}
Parp9	4.07	7.69	2579.87	1.49×10^{-56}	6.29×10^{-54}
H2.Q4	4.56	6.23	2611.98	3.10×10^{-56}	1.27×10^{-53}
Trim34a	4.47	4.43	2243.92	1.63×10^{-54}	6.50×10^{-52}
Stat2	3.89	7.60	2219.31	2.37×10^{-54}	9.15×10^{-52}
A930037H05Rik	3.93	9.63	2123.46	1.04×10^{-53}	3.92×10^{-51}
Znfx1	3.74	8.49	2054.56	3.15×10^{-53}	1.15×10^{-50}
Uba7	3.67	6.73	2040.23	3.98×10^{-53}	1.42×10^{-50}
Samhd1	3.62	7.68	1975.76	1.17×10^{-52}	4.05×10^{-50}
Gm26797	4.71	4.03	1930.72	2.53×10^{-52}	8.55×10^{-50}
H2.K1	3.62	9.49	1898.35	4.45×10^{-52}	1.47×10^{-49}
Igtp	5.98	8.24	3671.93	7.17×10^{-52}	2.31×10^{-49}
H2.D1	3.54	9.23	1822.07	1.75×10^{-51}	5.51×10^{-49}
Trafd1	3.43	7.28	1814.18	2.02×10^{-51}	6.22×10^{-49}
Apol9b	9.46	6.05	5164.80	2.20×10^{-51}	6.62×10^{-49}
Parp11	3.40	6.11	1791.55	3.08×10^{-51}	9.05×10^{-49}
Pcdh17	5.05	4.51	2447.18	3.74×10^{-51}	1.08×10^{-48}
Ddx58	3.19	7.55	1775.70	4.14×10^{-51}	1.17×10^{-48}
Mlkl	3.40	6.02	1762.19	5.34×10^{-51}	1.47×10^{-48}
Spats2l	3.17	7.35	1705.67	1.58×10^{-50}	4.28×10^{-48}
Eif2ak2	3.18	7.82	1692.26	2.06×10^{-50}	5.46×10^{-48}
Oas1b	6.34	6.59	3861.25	3.45×10^{-50}	8.99×10^{-48}
Daxx	3.16	6.16	1662.99	3.68×10^{-50}	9.39×10^{-48}
Gm43197	3.81	5.17	1850.36	8.98×10^{-50}	2.25×10^{-47}
Trim21	3.15	6.00	1609.82	1.08×10^{-49}	2.67×10^{-47}
Mir6381	6.99	3.50	2060.29	2.49×10^{-49}	6.01×10^{-47}
Gm19244	6.86	2.82	1553.39	3.54×10^{-49}	8.41×10^{-47}
Xdh	3.33	9.62	1528.07	6.11×10^{-49}	1.43×10^{-46}
Ifi27	3.76	4.09	1504.77	1.02×10^{-48}	2.33×10^{-46}
A930015D03Rik	3.14	5.50	1456.69	2.98×10^{-48}	6.72×10^{-46}
Nmi	2.98	5.36	1452.27	3.30×10^{-48}	7.31×10^{-46}
Trim34b	4.23	3.64	1390.98	1.37×10^{-47}	2.99×10^{-45}
Irf9	2.80	6.01	1383.26	1.65×10^{-47}	3.54×10^{-45}
Slfn2	4.08	3.64	1376.62	1.93×10^{-47}	4.02×10^{-45}
Gm20234	4.08	3.64	1376.62	1.93×10^{-47}	4.02×10^{-45}
H2.T.ps	3.80	3.92	1372.55	2.13×10^{-47}	4.36×10^{-45}

Tapbp	2.86	8.14	1337.12	5.04×10^{-47}	1.02×10^{-44}
Rbm43	2.70	5.52	1281.19	2.06×10^{-46}	4.09×10^{-44}
Lgals3bp	2.95	9.86	1273.48	2.51×10^{-46}	4.92×10^{-44}
Shisa5	2.82	7.60	1261.49	3.42×10^{-46}	6.62×10^{-44}
Ifi35	2.67	7.05	1234.83	6.91×10^{-46}	1.32×10^{-43}
Adar	2.70	7.17	1177.84	3.25×10^{-45}	6.11×10^{-43}
Parp10	2.60	6.65	1174.14	3.60×10^{-45}	6.68×10^{-43}
Rtp4	3.94	5.97	1998.68	1.24×10^{-44}	2.26×10^{-42}
Psmb10	2.62	5.89	1121.72	1.60×10^{-44}	2.89×10^{-42}
Nt5c3	2.58	7.45	1107.92	2.40×10^{-44}	4.28×10^{-42}
Rasa4	5.40	3.38	1542.50	2.96×10^{-44}	5.19×10^{-42}
Tdrd7	2.43	6.65	1097.86	3.23×10^{-44}	5.61×10^{-42}
Gm11626	2.69	4.93	1079.79	5.55×10^{-44}	9.51×10^{-42}
Gm38316	2.46	6.91	1071.28	7.19×10^{-44}	1.22×10^{-41}
Pnpt1	2.52	7.38	1064.06	8.96×10^{-44}	1.50×10^{-41}
Gm49342	2.49	7.60	1056.97	1.11×10^{-43}	1.84×10^{-41}
Pnp	2.49	7.64	1052.84	1.27×10^{-43}	2.06×10^{-41}
Mov10	2.63	5.36	1037.29	2.05×10^{-43}	3.30×10^{-41}
Apobec1	3.35	3.72	1033.28	2.33×10^{-43}	3.70×10^{-41}
Gm20661	3.67	3.84	1173.10	4.06×10^{-43}	6.39×10^{-41}
Pml	2.51	6.90	1011.30	4.68×10^{-43}	7.27×10^{-41}
Helz2	2.90	8.18	1213.07	5.23×10^{-43}	8.03×10^{-41}
Gm38050	5.85	2.46	1007.22	5.33×10^{-43}	8.11×10^{-41}
B2m	2.45	10.50	993.05	8.44×10^{-43}	1.27×10^{-40}
Patl2	2.45	10.51	991.93	8.76×10^{-43}	1.30×10^{-40}
Zc3hav1	2.31	7.54	990.15	9.28×10^{-43}	1.36×10^{-40}
C2	2.40	8.62	989.85	9.37×10^{-43}	1.36×10^{-40}
Gm45418	12.00	1.89	1023.82	1.99×10^{-42}	2.87×10^{-40}
Ifnb1	12.44	2.43	1294.57	2.22×10^{-42}	3.17×10^{-40}
Gm43198	3.71	3.21	933.76	6.18×10^{-42}	8.72×10^{-40}
Chrnb2	2.33	6.14	918.08	1.07×10^{-41}	1.49×10^{-39}
Btc	3.09	3.76	916.16	1.14×10^{-41}	1.58×10^{-39}
Trim25	2.35	8.00	911.83	1.33×10^{-41}	1.82×10^{-39}
Serpinb6b	2.39	7.20	911.03	1.37×10^{-41}	1.85×10^{-39}
Arel1	2.21	6.65	898.38	2.15×10^{-41}	2.88×10^{-39}
Rnf135	2.30	5.98	891.99	2.70×10^{-41}	3.58×10^{-39}
Gm19412	2.96	5.46	1231.13	2.86×10^{-41}	3.75×10^{-39}
Gm7030	2.58	6.05	1021.28	8.93×10^{-41}	1.16×10^{-38}
Aida	2.21	7.09	833.96	2.34×10^{-40}	3.02×10^{-38}
Mitd1	2.05	6.64	791.98	1.22×10^{-39}	1.56×10^{-37}
X9330175E14Rik	6.87	1.83	786.52	1.52×10^{-39}	1.92×10^{-37}
Tmem140	2.08	6.28	749.10	7.17×10^{-39}	8.98×10^{-37}
Lgals9	2.09	9.34	740.54	1.03×10^{-38}	1.28×10^{-36}
Gm20559	3.88	7.34	1866.07	1.36×10^{-38}	1.68×10^{-36}
Gm49730	4.95	2.13	731.81	1.50×10^{-38}	1.83×10^{-36}
H2.T23	3.62	4.92	1526.75	1.72×10^{-38}	2.07×10^{-36}
Rasgef1b	1.96	6.01	721.22	2.38×10^{-38}	2.86×10^{-36}
Rnf31	2.03	5.55	720.11	2.50×10^{-38}	2.97×10^{-36}
Gm17139	2.20	4.36	717.28	2.84×10^{-38}	3.34×10^{-36}
Setdb2	1.98	5.67	697.06	7.00×10^{-38}	8.16×10^{-36}
Zbtb12	1.97	6.08	675.62	1.87×10^{-37}	2.17×10^{-35}
n.R5s29	3.31	2.95	657.02	4.50×10^{-37}	5.15×10^{-35}
Gm16156	1.91	6.26	656.89	4.53×10^{-37}	5.15×10^{-35}
Rbl1	2.13	4.41	652.97	5.47×10^{-37}	6.16×10^{-35}
Mill2	3.74	3.52	1027.15	9.16×10^{-37}	1.02×10^{-34}
H2.T10	2.39	5.74	870.61	1.22×10^{-36}	1.35×10^{-34}

Ogfr	1.81	6.82	636.32	1.23×10^{-36}	1.35×10^{-34}
Psme1	1.86	7.21	633.45	1.41×10^{-36}	1.54×10^{-34}
Plac8	1.85	7.95	628.85	1.78×10^{-36}	1.92×10^{-34}
Ppm1k	1.98	5.00	619.46	2.84×10^{-36}	3.05×10^{-34}
Gm16464	6.28	1.52	610.29	4.52×10^{-36}	4.82×10^{-34}
Tmem184b	1.88	6.78	607.97	5.09×10^{-36}	5.38×10^{-34}
Tuba8	3.53	2.65	604.03	6.23×10^{-36}	6.54×10^{-34}
Xaf1	6.96	7.30	3812.81	6.99×10^{-36}	7.28×10^{-34}
Rnf213	3.87	10.60	1671.55	7.77×10^{-36}	8.03×10^{-34}
Trim12c	2.34	3.66	597.89	8.56×10^{-36}	8.78×10^{-34}
Tent4a	1.83	5.38	586.90	1.52×10^{-35}	1.55×10^{-33}
Trim30e.ps1	7.19	1.06	583.48	1.83×10^{-35}	1.84×10^{-33}
Trim26	1.74	6.47	582.29	1.95×10^{-35}	1.95×10^{-33}
H2.T22	2.08	4.23	581.11	2.07×10^{-35}	2.06×10^{-33}
Unc93b1	2.52	4.87	859.77	2.38×10^{-35}	2.36×10^{-33}
Gm47126	4.92	1.75	575.28	2.83×10^{-35}	2.78×10^{-33}
H2.M3	2.39	3.42	568.77	4.03×10^{-35}	3.92×10^{-33}
Naa20	1.74	5.85	566.74	4.50×10^{-35}	4.35×10^{-33}
Psme2	1.80	7.25	566.59	4.54×10^{-35}	4.35×10^{-33}
Ascc3	1.74	6.70	565.74	4.75×10^{-35}	4.53×10^{-33}
Cyren	1.84	6.00	563.61	5.34×10^{-35}	5.05×10^{-33}
Stoml1	1.80	5.18	550.48	1.11×10^{-34}	1.04×10^{-32}
AU020206	2.20	3.89	548.84	1.21×10^{-34}	1.13×10^{-32}
Ifna4	11.09	1.00	611.92	1.34×10^{-34}	1.24×10^{-32}
Gm11131	4.65	4.40	1815.20	1.49×10^{-34}	1.37×10^{-32}
Gm8909	3.31	2.70	543.25	1.66×10^{-34}	1.52×10^{-32}
Elf1	1.64	6.95	542.75	1.71×10^{-34}	1.55×10^{-32}
A230050P20Rik	2.66	4.11	766.80	2.73×10^{-34}	2.46×10^{-32}
Ccnd2	1.67	6.44	533.76	2.85×10^{-34}	2.56×10^{-32}
Car13	1.74	5.16	529.44	3.66×10^{-34}	3.26×10^{-32}
Atrip	1.86	5.98	563.37	4.59×10^{-34}	4.06×10^{-32}
Usp42	1.68	5.01	518.16	7.09×10^{-34}	6.23×10^{-32}
Atm	1.69	7.16	517.20	7.50×10^{-34}	6.55×10^{-32}
Cdca7l	2.24	3.58	508.88	1.23×10^{-33}	1.07×10^{-31}
Casp7	1.66	5.45	501.58	1.91×10^{-33}	1.65×10^{-31}
Grn	2.01	8.43	616.13	1.95×10^{-33}	1.67×10^{-31}
Mafk	1.70	5.59	499.76	2.14×10^{-33}	1.82×10^{-31}
Trim5	2.37	3.41	497.51	2.45×10^{-33}	2.08×10^{-31}
Tlr3	3.93	7.64	1780.22	3.00×10^{-33}	2.52×10^{-31}
Lgals8	1.59	7.12	493.70	3.10×10^{-33}	2.59×10^{-31}
Tor1aip1	1.60	7.48	492.98	3.24×10^{-33}	2.69×10^{-31}
Gm20663	10.67	0.47	536.11	3.48×10^{-33}	2.87×10^{-31}
Gm42917	3.02	2.64	490.56	3.76×10^{-33}	3.09×10^{-31}
Gm43068	8.82	0.63	509.02	4.33×10^{-33}	3.53×10^{-31}
Apof	4.07	1.94	486.88	4.73×10^{-33}	3.83×10^{-31}
Gm8979	10.61	0.40	523.60	6.93×10^{-33}	5.58×10^{-31}
Ehd4	1.57	6.07	476.77	8.95×10^{-33}	7.16×10^{-31}
Pcgf5	1.63	6.45	476.37	9.18×10^{-33}	7.30×10^{-31}
Tgtp2	8.66	0.48	487.67	1.55×10^{-32}	1.22×10^{-30}
Asb13	1.64	5.47	463.79	2.06×10^{-32}	1.62×10^{-30}
Gm5970	8.77	0.37	479.40	2.56×10^{-32}	2.01×10^{-30}
X9930111J21Rik1	7.16	1.02	500.29	2.60×10^{-32}	2.02×10^{-30}
Flt4	1.92	5.82	585.34	3.37×10^{-32}	2.61×10^{-30}
Asah2	1.60	6.44	454.31	3.84×10^{-32}	2.95×10^{-30}
Fbn1	6.21	0.56	451.49	4.64×10^{-32}	3.54×10^{-30}
Cmtr1	1.53	7.36	449.50	5.29×10^{-32}	4.02×10^{-30}

Crybg1	1.63	5.10	449.12	5.43×10^{-32}	4.11×10^{-30}
Trim6	2.22	4.72	670.09	5.59×10^{-32}	4.20×10^{-30}
Gm12216	2.83	2.73	446.90	6.30×10^{-32}	4.71×10^{-30}
Elmod2	1.68	6.03	468.96	6.70×10^{-32}	4.98×10^{-30}
Phf11c	1.91	4.13	448.75	1.19×10^{-31}	8.78×10^{-30}
X4933412E12Rik	2.13	3.44	434.43	1.47×10^{-31}	1.08×10^{-29}
Inpp1	2.59	5.82	935.86	1.59×10^{-31}	1.16×10^{-29}
Pnp2	2.62	2.76	432.62	1.67×10^{-31}	1.21×10^{-29}
Gm13822	4.33	1.24	430.42	1.94×10^{-31}	1.41×10^{-29}
Usp25	1.50	7.44	427.20	2.43×10^{-31}	1.75×10^{-29}
Pla1a	1.49	6.84	423.71	3.11×10^{-31}	2.23×10^{-29}
Gm17234	3.08	2.25	415.18	5.70×10^{-31}	4.06×10^{-29}
Isg20	1.57	7.79	413.51	6.43×10^{-31}	4.55×10^{-29}
Apobec3	1.58	5.39	411.75	7.29×10^{-31}	5.14×10^{-29}
Rnasel	3.10	2.29	399.47	1.79×10^{-30}	1.26×10^{-28}
Gm43126	1.78	3.89	398.69	1.90×10^{-30}	1.32×10^{-28}
Herc6	7.32	8.36	3698.68	2.48×10^{-30}	1.72×10^{-28}
Gm10136	4.50	1.30	388.15	4.18×10^{-30}	2.89×10^{-28}
Zfp365	2.43	2.80	385.55	5.10×10^{-30}	3.50×10^{-28}
N4bp1	1.42	6.45	385.51	5.11×10^{-30}	3.50×10^{-28}
Gm45712	3.68	1.62	384.85	5.38×10^{-30}	3.66×10^{-28}
AW011738	3.64	2.66	568.28	8.31×10^{-30}	5.62×10^{-28}
Ccdc167	1.40	6.85	376.28	1.04×10^{-29}	7.02×10^{-28}
Naip6	2.15	3.15	375.09	1.14×10^{-29}	7.66×10^{-28}
Naip2	1.42	5.57	373.96	1.25×10^{-29}	8.33×10^{-28}
Clec2d	1.40	5.94	370.93	1.59×10^{-29}	1.05×10^{-27}
Ptprh	4.29	1.68	446.29	1.82×10^{-29}	1.20×10^{-27}
Gm18068	3.49	3.82	955.95	1.91×10^{-29}	1.25×10^{-27}
Ankfy1	1.37	8.07	363.87	2.78×10^{-29}	1.82×10^{-27}
Gm15701	5.09	0.82	360.13	3.76×10^{-29}	2.44×10^{-27}
Rnf114	1.36	7.18	359.28	4.02×10^{-29}	2.60×10^{-27}
Tbrg1	1.39	7.38	351.89	7.37×10^{-29}	4.75×10^{-27}
Arap2	1.37	6.66	351.02	7.91×10^{-29}	5.07×10^{-27}
Gm28043	1.34	7.48	350.73	8.10×10^{-29}	5.17×10^{-27}
Trim14	1.43	6.01	349.96	8.64×10^{-29}	5.49×10^{-27}
Dll1	3.15	1.84	344.48	1.36×10^{-28}	8.63×10^{-27}
G3bp2	1.36	8.09	338.74	2.22×10^{-28}	1.39×10^{-26}
Tent5a	3.12	5.34	1148.98	2.61×10^{-28}	1.64×10^{-26}
Erap1	1.40	7.18	336.53	2.68×10^{-28}	1.67×10^{-26}
Ifit2	8.72	10.79	4165.48	3.55×10^{-28}	2.20×10^{-26}
Gm12186	4.27	0.71	332.44	3.81×10^{-28}	2.35×10^{-26}
Gm43196	3.96	2.88	716.14	3.88×10^{-28}	2.38×10^{-26}
Gm10271	2.13	3.00	330.96	4.32×10^{-28}	2.65×10^{-26}
Vwa5a	1.38	7.67	330.15	4.64×10^{-28}	2.83×10^{-26}
X9230109A22Rik	4.58	0.64	328.49	5.36×10^{-28}	3.25×10^{-26}
Nampt	1.50	7.91	364.18	6.34×10^{-28}	3.83×10^{-26}
Cenpj	1.43	4.35	324.38	7.69×10^{-28}	4.63×10^{-26}
Tapbpl	2.36	5.21	729.50	9.80×10^{-28}	5.87×10^{-26}
Ifi27l2b	3.09	1.81	316.26	1.59×10^{-27}	9.46×10^{-26}
Rnf19b	1.51	4.31	312.27	2.28×10^{-27}	1.35×10^{-25}
Il12rb1	4.07	0.99	312.23	2.29×10^{-27}	1.35×10^{-25}
Adamts6	1.82	4.98	471.90	2.78×10^{-27}	1.63×10^{-25}
Olfr1372.ps1	2.25	6.41	709.78	2.97×10^{-27}	1.74×10^{-25}
Pla2g4a	2.02	2.89	308.37	3.25×10^{-27}	1.90×10^{-25}
Arhgap45	1.76	3.41	306.62	3.83×10^{-27}	2.22×10^{-25}
Nsmaf	1.26	5.31	305.87	4.10×10^{-27}	2.37×10^{-25}

Usb1	1.29	5.13	303.31	5.20×10^{-27}	3.00×10^{-25}
Zcchc2	1.22	6.44	300.80	6.58×10^{-27}	3.77×10^{-25}
Gm17017	3.68	1.31	300.40	6.83×10^{-27}	3.90×10^{-25}
Axl	1.35	9.64	299.38	7.52×10^{-27}	4.27×10^{-25}
Eef1akmt4	1.99	3.29	325.51	7.99×10^{-27}	4.52×10^{-25}
H2.Q1	3.41	2.12	367.71	1.02×10^{-26}	5.75×10^{-25}
Tcf4	1.30	4.83	291.85	1.54×10^{-26}	8.64×10^{-25}
Prkce	1.52	3.69	289.32	1.97×10^{-26}	1.10×10^{-24}
Rbms2	1.24	5.64	286.29	2.64×10^{-26}	1.47×10^{-24}
Cox18	1.26	4.66	286.11	2.69×10^{-26}	1.49×10^{-24}
Serpinb9b	1.33	5.00	285.62	2.82×10^{-26}	1.56×10^{-24}
Rfx5	1.32	4.90	285.34	2.90×10^{-26}	1.59×10^{-24}
Gm15856	3.60	1.05	284.45	3.16×10^{-26}	1.73×10^{-24}
Ly6c1	4.84	1.45	446.30	3.24×10^{-26}	1.77×10^{-24}
Vamp1	1.27	5.90	283.65	3.42×10^{-26}	1.86×10^{-24}
X1700126G02Rik	1.41	8.23	319.79	3.69×10^{-26}	2.00×10^{-24}
Trex1	2.92	5.52	1008.70	4.48×10^{-26}	2.41×10^{-24}
Serping1	3.02	2.18	321.48	4.55×10^{-26}	2.44×10^{-24}
Gm44075	3.57	1.16	279.61	5.10×10^{-26}	2.73×10^{-24}
Mocos	1.31	4.56	279.14	5.35×10^{-26}	2.85×10^{-24}
Skap1	4.25	0.54	278.86	5.50×10^{-26}	2.92×10^{-24}
Nudt13	1.27	4.67	278.65	5.62×10^{-26}	2.97×10^{-24}
Gm20478	3.03	2.07	301.59	8.56×10^{-26}	4.51×10^{-24}
Cntnap5a	1.43	3.99	273.51	9.42×10^{-26}	4.94×10^{-24}
H2.Q7	3.12	3.12	560.69	9.74×10^{-26}	5.09×10^{-24}
Slfn5	10.91	0.88	516.43	1.33×10^{-25}	6.92×10^{-24}
Enpp5	1.19	6.69	269.37	1.44×10^{-25}	7.45×10^{-24}
Akt3	1.19	6.65	268.00	1.66×10^{-25}	8.55×10^{-24}
Ogfrl1	1.40	4.26	267.81	1.69×10^{-25}	8.69×10^{-24}
Rhoc	1.17	6.57	266.91	1.85×10^{-25}	9.50×10^{-24}
Tut7	1.16	7.84	265.97	2.04×10^{-25}	1.04×10^{-23}
Plekha4	2.14	2.72	264.48	2.38×10^{-25}	1.21×10^{-23}
Gm15821	4.62	4.89	1692.53	3.15×10^{-25}	1.60×10^{-23}
Gch1	1.23	6.09	260.98	4.19×10^{-25}	2.11×10^{-23}
Sftpd	6.58	4.10	1957.56	6.00×10^{-25}	3.02×10^{-23}
B230398E01Rik	1.39	4.05	254.94	6.53×10^{-25}	3.27×10^{-23}
Pla2g16	1.21	4.61	254.85	6.60×10^{-25}	3.29×10^{-23}
Plgrkt	1.11	6.27	252.63	8.37×10^{-25}	4.16×10^{-23}
March5	1.27	4.62	248.73	1.28×10^{-24}	6.34×10^{-23}
Fbxw17	3.01	4.45	903.58	1.82×10^{-24}	9.00×10^{-23}
Ifi44	8.31	7.82	3622.22	2.85×10^{-24}	1.40×10^{-22}
Napsa	1.18	5.21	239.08	3.74×10^{-24}	1.83×10^{-22}
Lima1	1.10	6.02	238.48	4.00×10^{-24}	1.95×10^{-22}
Gm15396	1.18	5.20	237.44	4.50×10^{-24}	2.19×10^{-22}
Ap3m2	1.24	4.68	237.25	4.60×10^{-24}	2.23×10^{-22}
Taok3	1.08	5.84	236.19	5.18×10^{-24}	2.51×10^{-22}
Car2	1.36	3.93	234.05	6.62×10^{-24}	3.19×10^{-22}
Triobp	1.16	5.20	232.41	7.99×10^{-24}	3.84×10^{-22}
Gcnt2	1.29	6.37	274.85	8.03×10^{-24}	3.84×10^{-22}
Ifi208	8.52	0.96	487.95	1.05×10^{-23}	5.00×10^{-22}
Rrbp1	1.12	8.81	229.98	1.06×10^{-23}	5.03×10^{-22}
Hist1h1d	2.04	2.64	229.85	1.08×10^{-23}	5.09×10^{-22}
Socs1	5.82	1.11	372.57	1.23×10^{-23}	5.78×10^{-22}
Abi3bp	3.36	0.64	227.48	1.42×10^{-23}	6.66×10^{-22}
Etohd2	1.09	5.25	227.31	1.45×10^{-23}	6.77×10^{-22}
Vcpip1	1.12	7.23	225.46	1.80×10^{-23}	8.39×10^{-22}

Ppa1	1.06	5.85	224.73	1.96×10^{-23}	9.11×10^{-22}
Gm19684	3.51	0.66	224.33	2.06×10^{-23}	9.53×10^{-22}
Ccdc6	1.06	6.21	221.97	2.72×10^{-23}	1.26×10^{-21}
Gm8739	2.99	4.48	877.84	3.32×10^{-23}	1.53×10^{-21}
Naip5	2.32	1.89	219.99	3.45×10^{-23}	1.58×10^{-21}
Casp2	1.07	5.80	219.99	3.45×10^{-23}	1.58×10^{-21}
Gm10865	1.23	4.23	218.69	4.04×10^{-23}	1.84×10^{-21}
Apol7a	2.64	3.63	546.36	4.10×10^{-23}	1.86×10^{-21}
Gm10499	2.30	1.94	217.44	4.70×10^{-23}	2.13×10^{-21}
B230307C23Rik	1.68	4.87	384.34	4.71×10^{-23}	2.13×10^{-21}
Vars	1.10	7.39	216.43	5.31×10^{-23}	2.39×10^{-21}
Usp12	1.03	5.88	216.33	5.37×10^{-23}	2.41×10^{-21}
Phc3	1.28	7.15	265.32	6.42×10^{-23}	2.87×10^{-21}
Etnk1	1.45	8.68	313.42	6.82×10^{-23}	3.03×10^{-21}
Grid2	2.87	1.32	213.20	7.88×10^{-23}	3.50×10^{-21}
Pttg1	2.38	1.91	212.41	8.69×10^{-23}	3.84×10^{-21}
Gm17058	1.06	5.52	212.04	9.10×10^{-23}	4.01×10^{-21}
Tasor2	1.07	6.70	211.26	1.00×10^{-22}	4.40×10^{-21}
Wdfy1	1.02	6.38	210.94	1.04×10^{-22}	4.56×10^{-21}
Ly6e	2.02	9.45	515.46	1.24×10^{-22}	5.42×10^{-21}
Gm7160	1.28	3.91	208.76	1.37×10^{-22}	5.95×10^{-21}
C130026I21Rik	3.12	2.18	321.31	1.38×10^{-22}	6.00×10^{-21}
Tmem192	1.11	4.58	207.83	1.54×10^{-22}	6.65×10^{-21}
Zdhhc18	1.24	4.09	205.11	2.17×10^{-22}	9.34×10^{-21}
Upp1	2.32	2.56	257.88	2.42×10^{-22}	1.04×10^{-20}
Emp2	1.49	3.19	203.86	2.54×10^{-22}	1.09×10^{-20}
Lmo2	6.09	0.56	344.58	3.01×10^{-22}	1.29×10^{-20}
Fer	1.04	4.94	200.22	4.05×10^{-22}	1.72×10^{-20}
Optn	1.34	6.28	283.75	4.06×10^{-22}	1.72×10^{-20}
Pigr	2.65	1.33	199.91	4.22×10^{-22}	1.78×10^{-20}
Gm26637	1.95	2.40	198.32	5.18×10^{-22}	2.19×10^{-20}
Smpdl3a	1.42	3.33	196.27	6.78×10^{-22}	2.85×10^{-20}
Gm26582	1.42	7.61	304.17	7.06×10^{-22}	2.96×10^{-20}
Trim56	1.00	7.11	195.37	7.63×10^{-22}	3.19×10^{-20}
Apol10b	4.26	2.82	664.20	7.86×10^{-22}	3.27×10^{-20}
X0610043K17Rik	2.42	1.50	194.93	8.08×10^{-22}	3.35×10^{-20}
D530033B14Rik	1.04	6.27	194.65	8.38×10^{-22}	3.47×10^{-20}
Gm49354	0.99	6.10	193.06	1.03×10^{-21}	4.27×10^{-20}
Fndc3a	1.18	7.16	228.31	1.22×10^{-21}	5.02×10^{-20}
Pxk	0.99	7.23	190.66	1.42×10^{-21}	5.84×10^{-20}
Ttc39b	1.05	4.93	189.58	1.65×10^{-21}	6.73×10^{-20}
Tor1aip2	0.99	9.38	188.93	1.80×10^{-21}	7.33×10^{-20}
X1600014C10Rik	1.02	6.37	188.15	2.00×10^{-21}	8.12×10^{-20}
Gm43069	2.67	4.57	718.20	2.71×10^{-21}	1.10×10^{-19}
Arid5a	3.70	2.73	500.31	2.90×10^{-21}	1.17×10^{-19}
Rad51	1.03	5.59	184.93	3.09×10^{-21}	1.25×10^{-19}
Klf4	1.28	3.76	183.90	3.57×10^{-21}	1.43×10^{-19}
Ccdc25	0.99	5.40	183.34	3.85×10^{-21}	1.54×10^{-19}
Rnf139	1.00	6.75	183.30	3.87×10^{-21}	1.55×10^{-19}
Myd88	1.63	5.06	357.46	4.15×10^{-21}	1.65×10^{-19}
Vps54	1.06	6.99	190.06	4.28×10^{-21}	1.70×10^{-19}
Zfp296	2.18	1.91	181.99	4.64×10^{-21}	1.83×10^{-19}
Rmdn3	1.02	5.98	181.65	4.86×10^{-21}	1.91×10^{-19}
Ilrun	0.97	7.77	180.98	5.34×10^{-21}	2.09×10^{-19}
Il18	1.49	3.05	180.42	5.77×10^{-21}	2.26×10^{-19}
Sertad4	1.28	3.65	174.68	1.30×10^{-20}	5.06×10^{-19}

Ano2	3.39	5.66	1108.36	1.32×10^{-20}	5.12×10^{-19}
Rfc3	1.52	2.91	173.76	1.48×10^{-20}	5.72×10^{-19}
Tmem209	0.95	6.32	173.00	1.65×10^{-20}	6.36×10^{-19}
Irf2	0.99	5.70	172.06	1.89×10^{-20}	7.26×10^{-19}
Efhb	2.74	0.92	171.29	2.11×10^{-20}	8.07×10^{-19}
Pon3	0.95	5.47	171.09	2.17×10^{-20}	8.29×10^{-19}
Sgms2	2.07	1.94	170.46	2.38×10^{-20}	9.03×10^{-19}
Tmem67	1.20	3.59	168.11	3.36×10^{-20}	1.27×10^{-18}
Cd47	1.82	8.02	426.62	3.68×10^{-20}	1.38×10^{-18}
A730081D07Rik	1.30	5.93	259.68	4.59×10^{-20}	1.72×10^{-18}
Anxa10	3.94	0.23	222.61	4.86×10^{-20}	1.82×10^{-18}
Mir6385	2.32	1.34	164.48	5.75×10^{-20}	2.14×10^{-18}
Pgap2	0.94	5.01	164.15	6.04×10^{-20}	2.24×10^{-18}
Rnf14	1.05	7.41	181.32	7.27×10^{-20}	2.70×10^{-18}
Nemp1	1.00	4.24	162.25	8.02×10^{-20}	2.96×10^{-18}
Gm28052	0.89	6.20	162.03	8.30×10^{-20}	3.06×10^{-18}
Parp3	0.95	5.28	161.84	8.53×10^{-20}	3.14×10^{-18}
BC051226	2.20	1.58	161.15	9.47×10^{-20}	3.47×10^{-18}
Gm17193	2.13	1.68	159.85	1.15×10^{-19}	4.22×10^{-18}
Gm49333	1.77	3.44	255.78	1.60×10^{-19}	5.82×10^{-18}
Aim2	3.26	0.53	182.33	1.65×10^{-19}	5.97×10^{-18}
Gm6904	2.73	0.83	157.24	1.72×10^{-19}	6.22×10^{-18}
Myc	0.93	6.19	155.99	2.08×10^{-19}	7.52×10^{-18}
Naa25	0.91	6.44	155.88	2.12×10^{-19}	7.63×10^{-18}
A630089N07Rik	1.39	5.63	282.21	2.36×10^{-19}	8.45×10^{-18}
Akr1b3	0.95	5.03	154.89	2.47×10^{-19}	8.83×10^{-18}
Gm14546	0.95	5.03	154.46	2.64×10^{-19}	9.41×10^{-18}
Heatr1	0.89	6.00	154.13	2.78×10^{-19}	9.88×10^{-18}
Stambpl1	0.94	4.77	153.22	3.21×10^{-19}	1.14×10^{-17}
Phc2	0.90	5.53	152.78	3.44×10^{-19}	1.22×10^{-17}
Gm6548	2.22	4.21	461.75	3.47×10^{-19}	1.22×10^{-17}
F11	1.73	2.25	152.72	3.47×10^{-19}	1.22×10^{-17}
Col9a3	1.81	2.22	152.67	3.50×10^{-19}	1.23×10^{-17}
Zup1	2.70	5.40	741.87	5.02×10^{-19}	1.76×10^{-17}
Sptlc2	0.86	7.14	148.62	6.64×10^{-19}	2.31×10^{-17}
Lama3	2.26	1.23	147.64	7.78×10^{-19}	2.70×10^{-17}
Ifi204	6.81	8.23	2440.73	8.43×10^{-19}	2.92×10^{-17}
Fnbp4	0.85	6.82	147.09	8.50×10^{-19}	2.93×10^{-17}
Pold3	0.85	5.45	145.23	1.15×10^{-18}	3.95×10^{-17}
Gm45698	1.57	2.63	144.83	1.22×10^{-18}	4.19×10^{-17}
Rab5c	0.86	7.62	143.81	1.45×10^{-18}	4.95×10^{-17}
Gca	1.08	5.31	180.49	1.54×10^{-18}	5.24×10^{-17}
Ppp1r12a	0.88	8.23	143.09	1.63×10^{-18}	5.54×10^{-17}
Zfhx4	1.02	6.22	171.19	1.76×10^{-18}	5.95×10^{-17}
Gdap10	1.39	2.79	141.00	2.30×10^{-18}	7.76×10^{-17}
Nub1	0.87	7.91	140.78	2.38×10^{-18}	8.02×10^{-17}
Plekha7	1.00	6.51	164.98	2.39×10^{-18}	8.03×10^{-17}
Esyt1	0.91	4.69	138.24	3.65×10^{-18}	1.22×10^{-16}
Lrrc51	0.90	5.01	137.45	4.16×10^{-18}	1.39×10^{-16}
Gsdmd	1.45	7.22	287.80	4.28×10^{-18}	1.42×10^{-16}
Kat2a	0.94	5.17	143.04	4.44×10^{-18}	1.47×10^{-16}
Chic1	1.39	3.46	172.61	4.94×10^{-18}	1.63×10^{-16}
Hip1r	0.83	7.03	135.62	5.68×10^{-18}	1.88×10^{-16}
Sppl2a	1.21	7.63	216.72	6.52×10^{-18}	2.15×10^{-16}
Mrpl27	0.90	4.44	133.44	8.24×10^{-18}	2.71×10^{-16}
Msn	0.82	7.79	133.19	8.61×10^{-18}	2.82×10^{-16}

E230025N22Rik	1.81	2.12	132.78	9.24×10^{-18}	3.02×10^{-16}
Gm22728	1.29	2.91	131.11	1.23×10^{-17}	4.00×10^{-16}
Zfpm2	2.41	0.97	130.83	1.30×10^{-17}	4.18×10^{-16}
Capn5	0.82	6.00	130.59	1.35×10^{-17}	4.35×10^{-16}
Trmt1l	0.81	6.17	130.42	1.39×10^{-17}	4.48×10^{-16}
Rnpep	0.82	5.63	130.20	1.45×10^{-17}	4.64×10^{-16}
X2610002M06Rik	0.90	4.71	129.93	1.52×10^{-17}	4.86×10^{-16}
Dnah11	2.05	1.42	129.91	1.52×10^{-17}	4.86×10^{-16}
Tmco4	1.05	3.49	129.79	1.55×10^{-17}	4.95×10^{-16}
Kdelc2	0.85	4.58	129.75	1.56×10^{-17}	4.97×10^{-16}
Jade2	0.84	6.61	129.72	1.57×10^{-17}	4.98×10^{-16}
Slc25a28	0.83	6.03	129.68	1.58×10^{-17}	5.01×10^{-16}
Hsd11b1	2.07	1.27	129.33	1.68×10^{-17}	5.30×10^{-16}
H2.Q6	3.97	4.15	915.09	1.87×10^{-17}	5.86×10^{-16}
Bbx	0.83	6.66	128.60	1.92×10^{-17}	6.01×10^{-16}
Iqgap2	0.99	3.84	128.53	1.94×10^{-17}	6.06×10^{-16}
Atp1b3	0.81	6.45	128.20	2.06×10^{-17}	6.40×10^{-16}
Gcnt1	0.84	4.93	127.02	2.53×10^{-17}	7.86×10^{-16}
Gm44250	0.80	5.83	126.04	3.02×10^{-17}	9.33×10^{-16}
Il1rap	1.18	3.28	125.04	3.61×10^{-17}	1.11×10^{-15}
Npc2	0.84	8.19	124.91	3.69×10^{-17}	1.13×10^{-15}
Resf1	0.82	7.02	124.28	4.14×10^{-17}	1.26×10^{-15}
Capza2	0.83	8.58	124.06	4.31×10^{-17}	1.31×10^{-15}
Fam84b	0.80	5.63	123.98	4.37×10^{-17}	1.33×10^{-15}
Isoc1	0.81	5.96	123.66	4.63×10^{-17}	1.41×10^{-15}
Specc1	0.82	5.88	123.16	5.07×10^{-17}	1.53×10^{-15}
Efcab14	0.78	6.87	122.94	5.27×10^{-17}	1.59×10^{-15}
Ddx24	0.80	6.50	122.74	5.47×10^{-17}	1.64×10^{-15}
X4930453N24Rik	0.82	4.73	122.67	5.54×10^{-17}	1.66×10^{-15}
Azi2	0.79	7.55	121.22	7.22×10^{-17}	2.16×10^{-15}
Gm26648	0.90	6.88	135.67	8.71×10^{-17}	2.60×10^{-15}
Cdh17	0.81	5.67	119.97	9.09×10^{-17}	2.71×10^{-15}
Adgrg2	0.79	5.77	119.47	9.96×10^{-17}	2.96×10^{-15}
Grina	0.91	7.29	135.83	1.04×10^{-16}	3.09×10^{-15}
Smg7	0.77	7.02	118.84	1.12×10^{-16}	3.31×10^{-15}
Ddx60	10.59	6.23	2705.48	1.30×10^{-16}	3.82×10^{-15}
Xrn1	0.79	6.86	117.76	1.37×10^{-16}	4.03×10^{-15}
Wdtc1	0.81	5.11	117.74	1.37×10^{-16}	4.03×10^{-15}
X4930551O13Rik	1.32	2.76	117.58	1.41×10^{-16}	4.14×10^{-15}
Ubc	0.83	5.01	117.42	1.46×10^{-16}	4.27×10^{-15}
Gm42047	1.06	3.45	117.40	1.46×10^{-16}	4.27×10^{-15}
Pi4k2a	0.76	6.61	116.96	1.59×10^{-16}	4.63×10^{-15}
Laptm4a	0.77	8.32	116.64	1.69×10^{-16}	4.89×10^{-15}
mt.Rnr1	2.69	9.87	641.36	1.73×10^{-16}	4.99×10^{-15}
Idnk	0.78	5.21	116.19	1.84×10^{-16}	5.30×10^{-15}
Fbxo42	0.85	4.65	115.54	2.08×10^{-16}	5.98×10^{-15}
D5Ertd579e	0.78	7.89	115.28	2.18×10^{-16}	6.24×10^{-15}
Epsti1	7.67	1.35	439.12	2.26×10^{-16}	6.45×10^{-15}
Ifi205	8.43	2.30	688.21	2.66×10^{-16}	7.56×10^{-15}
Gm42936	0.77	6.97	114.20	2.68×10^{-16}	7.62×10^{-15}
Keap1	0.77	6.76	113.84	2.87×10^{-16}	8.13×10^{-15}
Aftph	0.77	7.71	113.82	2.88×10^{-16}	8.14×10^{-15}
E130102H24Rik	1.17	2.97	113.50	3.06×10^{-16}	8.62×10^{-15}
Dnajc10	0.81	7.92	113.49	3.07×10^{-16}	8.63×10^{-15}
Smim3	0.85	4.34	113.36	3.14×10^{-16}	8.82×10^{-15}
Tbc1d8	0.77	5.19	112.18	3.94×10^{-16}	1.10×10^{-14}

Selenow	0.77	6.90	111.47	4.52×10^{-16}	1.26×10^{-14}
X4930570N18Rik	2.16	0.46	111.00	4.95×10^{-16}	1.38×10^{-14}
St6galnac4	1.62	1.87	110.21	5.77×10^{-16}	1.59×10^{-14}
Nedd9	0.76	5.52	109.58	6.53×10^{-16}	1.79×10^{-14}
Nlrc5	6.91	5.50	1918.39	7.25×10^{-16}	1.97×10^{-14}
Psmf1	0.77	5.61	108.98	7.34×10^{-16}	1.99×10^{-14}
Pik3ip1	0.80	5.13	108.16	8.62×10^{-16}	2.34×10^{-14}
Leprotl1	0.79	5.23	108.15	8.65×10^{-16}	2.34×10^{-14}
Clcn5	0.73	5.94	108.00	8.91×10^{-16}	2.41×10^{-14}
Tatdn1	0.76	7.07	107.54	9.76×10^{-16}	2.63×10^{-14}
Cutc	0.80	4.87	106.84	1.12×10^{-15}	3.01×10^{-14}
Wars	0.79	5.43	106.58	1.18×10^{-15}	3.16×10^{-14}
Gnl1	0.76	4.84	106.39	1.22×10^{-15}	3.27×10^{-14}
Slfn9	6.24	4.99	1633.06	1.39×10^{-15}	3.70×10^{-14}
Brd2	0.71	7.10	105.29	1.53×10^{-15}	4.04×10^{-14}
Agrn	0.77	9.40	104.74	1.70×10^{-15}	4.50×10^{-14}
Dcbld1	0.81	4.39	104.23	1.89×10^{-15}	4.98×10^{-14}
Lamp2	0.81	9.96	105.27	1.92×10^{-15}	5.04×10^{-14}
Pmepa1	0.78	4.52	103.67	2.11×10^{-15}	5.54×10^{-14}
Dbnl	0.72	6.60	103.26	2.30×10^{-15}	6.00×10^{-14}
Wdr43	0.75	6.78	102.77	2.54×10^{-15}	6.61×10^{-14}
Ifi209	9.47	1.13	407.97	2.65×10^{-15}	6.90×10^{-14}
Ubr4	0.76	8.18	102.11	2.90×10^{-15}	7.53×10^{-14}
Gm49721	1.40	2.28	101.57	3.24×10^{-15}	8.38×10^{-14}
Dtx4	0.72	5.96	101.32	3.41×10^{-15}	8.80×10^{-14}
Gm16754	0.73	6.25	101.29	3.43×10^{-15}	8.84×10^{-14}
Whamm	1.25	6.04	209.83	4.05×10^{-15}	1.03×10^{-13}
Plekhf2	0.80	4.30	100.31	4.19×10^{-15}	1.07×10^{-13}
M6pr	0.70	8.09	100.08	4.40×10^{-15}	1.12×10^{-13}
Wfdc2	0.75	5.32	100.03	4.44×10^{-15}	1.12×10^{-13}
Rnaset2b	1.40	2.28	100.02	4.46×10^{-15}	1.12×10^{-13}
Dapp1	1.18	2.93	99.72	4.82×10^{-15}	1.21×10^{-13}
Crim1	0.75	5.38	99.30	5.17×10^{-15}	1.30×10^{-13}
F530104D19Rik	1.61	1.75	99.27	5.20×10^{-15}	1.30×10^{-13}
Mrpl30	0.72	6.88	98.93	5.59×10^{-15}	1.39×10^{-13}
Gm15241	0.80	4.72	101.25	5.99×10^{-15}	1.49×10^{-13}
Gm18445	8.50	1.53	456.29	6.77×10^{-15}	1.68×10^{-13}
Cenpl	1.01	3.17	97.95	6.84×10^{-15}	1.70×10^{-13}
Sec22a	0.76	4.75	97.62	7.33×10^{-15}	1.81×10^{-13}
Il18bp	3.59	5.15	867.74	8.01×10^{-15}	1.97×10^{-13}
Xrn2	0.71	7.47	97.18	8.05×10^{-15}	1.98×10^{-13}
Minpp1	0.69	5.91	96.90	8.54×10^{-15}	2.09×10^{-13}
Tmem229b	1.75	2.01	113.24	9.11×10^{-15}	2.23×10^{-13}
Gm36099	1.30	2.50	96.33	9.62×10^{-15}	2.35×10^{-13}
Platr22	1.66	1.70	95.91	1.05×10^{-14}	2.56×10^{-13}
Jpt2	1.16	5.65	183.17	1.10×10^{-14}	2.67×10^{-13}
Map2k1	0.69	7.59	95.61	1.12×10^{-14}	2.72×10^{-13}
Thbs1	1.72	1.48	95.50	1.15×10^{-14}	2.78×10^{-13}
Misp	4.82	3.13	679.73	1.17×10^{-14}	2.83×10^{-13}
H2.Q5	3.54	2.63	357.61	1.20×10^{-14}	2.88×10^{-13}
Irgm1	4.04	6.52	1062.44	1.23×10^{-14}	2.94×10^{-13}
Kat6a	0.68	6.85	95.17	1.23×10^{-14}	2.95×10^{-13}
Cd93	5.74	0.45	262.08	1.31×10^{-14}	3.13×10^{-13}
H2.K2	3.64	3.27	507.22	1.32×10^{-14}	3.15×10^{-13}
Flt3l	1.41	2.34	94.61	1.39×10^{-14}	3.30×10^{-13}
Ifit1bl2	1.93	3.39	241.51	1.51×10^{-14}	3.57×10^{-13}

Fhl2	0.78	4.46	93.34	1.82×10^{-14}	4.28×10^{-13}
Anxa7	0.68	6.86	93.09	1.92×10^{-14}	4.50×10^{-13}
Slc35f5	0.68	7.15	92.81	2.04×10^{-14}	4.76×10^{-13}
Gm45542	1.69	1.29	92.80	2.04×10^{-14}	4.76×10^{-13}
Gm8750	3.44	2.60	335.08	2.05×10^{-14}	4.76×10^{-13}
Gm45753	1.12	3.07	95.43	2.14×10^{-14}	4.98×10^{-13}
Zbtb5	0.88	3.72	92.52	2.17×10^{-14}	5.04×10^{-13}
Mir1943	1.82	1.09	91.88	2.50×10^{-14}	5.77×10^{-13}
Gm17057	1.03	3.10	91.54	2.69×10^{-14}	6.19×10^{-13}
G430095P16Rik	1.35	2.44	91.76	2.69×10^{-14}	6.19×10^{-13}
Ttc9c	0.67	6.27	91.00	3.02×10^{-14}	6.92×10^{-13}
Stard5	0.73	4.87	90.70	3.23×10^{-14}	7.36×10^{-13}
Otud5	0.67	7.83	90.66	3.26×10^{-14}	7.42×10^{-13}
Nck2	0.70	5.90	90.64	3.27×10^{-14}	7.44×10^{-13}
Dtwd1	0.79	3.96	89.41	4.29×10^{-14}	9.70×10^{-13}
Chmp4b	0.67	7.33	88.62	5.10×10^{-14}	1.15×10^{-12}
Pitpnc1	0.72	4.63	88.56	5.17×10^{-14}	1.16×10^{-12}
Tspo	0.68	7.08	87.93	5.95×10^{-14}	1.33×10^{-12}
Trim30d	8.07	7.26	2065.10	6.66×10^{-14}	1.49×10^{-12}
Kat2b	0.67	6.19	87.01	7.30×10^{-14}	1.63×10^{-12}
Ankmy1	1.51	1.70	86.85	7.58×10^{-14}	1.69×10^{-12}
Prune2	0.91	3.39	86.79	7.68×10^{-14}	1.71×10^{-12}
Orm3	1.52	1.71	86.29	8.59×10^{-14}	1.91×10^{-12}
Syvn1	0.67	5.87	85.97	9.23×10^{-14}	2.04×10^{-12}
C1ra	2.04	4.84	368.54	1.02×10^{-13}	2.24×10^{-12}
Cct3	0.68	7.85	85.49	1.03×10^{-13}	2.27×10^{-12}
Tpst1	3.60	3.66	567.99	1.07×10^{-13}	2.35×10^{-12}
Tmem185a	0.68	5.45	85.23	1.09×10^{-13}	2.39×10^{-12}
Zeb1	0.72	5.88	89.41	1.27×10^{-13}	2.79×10^{-12}
Uts2b	0.72	5.23	85.86	1.50×10^{-13}	3.27×10^{-12}
Cyth1	0.66	6.19	83.63	1.57×10^{-13}	3.41×10^{-12}
Larp1	0.65	7.30	82.76	1.92×10^{-13}	4.14×10^{-12}
Tgif1	0.69	5.32	82.49	2.04×10^{-13}	4.40×10^{-12}
Ms4a4d	6.25	2.44	533.51	2.09×10^{-13}	4.49×10^{-12}
Cpsf2	0.66	5.80	82.26	2.15×10^{-13}	4.61×10^{-12}
Bcl9	0.69	5.37	82.25	2.16×10^{-13}	4.61×10^{-12}
C1s2	1.70	1.00	81.90	2.34×10^{-13}	4.98×10^{-12}
Nfix	1.84	6.10	334.70	2.62×10^{-13}	5.52×10^{-12}
Cenpe	1.40	2.47	93.40	2.76×10^{-13}	5.80×10^{-12}
Gm3086	0.64	5.58	81.17	2.77×10^{-13}	5.82×10^{-12}
Txn1	0.63	6.39	81.10	2.82×10^{-13}	5.91×10^{-12}
C1qtnf1	1.64	1.34	80.56	3.20×10^{-13}	6.68×10^{-12}
Dzip1l	0.65	5.89	80.18	3.50×10^{-13}	7.27×10^{-12}
Ythdf1	0.63	6.22	80.03	3.62×10^{-13}	7.53×10^{-12}
Gab2	0.68	4.89	80.02	3.63×10^{-13}	7.54×10^{-12}
Serpinb9	3.51	7.69	787.14	3.79×10^{-13}	7.86×10^{-12}
Pdia5	1.30	2.09	79.62	3.99×10^{-13}	8.22×10^{-12}
Tbk1	1.11	6.75	159.29	4.53×10^{-13}	9.32×10^{-12}
BC023719	0.86	3.59	79.00	4.61×10^{-13}	9.47×10^{-12}
Ankrd17	0.64	8.37	78.76	4.89×10^{-13}	9.99×10^{-12}
Apbb2	0.65	5.62	78.74	4.91×10^{-13}	1.00×10^{-11}
Gm11747	1.50	1.61	78.23	5.54×10^{-13}	1.13×10^{-11}
Plod3	0.63	5.62	78.09	5.73×10^{-13}	1.16×10^{-11}
Gfod2	0.95	3.01	77.91	5.98×10^{-13}	1.21×10^{-11}
Fyco1	0.63	7.05	77.65	6.36×10^{-13}	1.29×10^{-11}
Ptprt	0.98	2.93	77.57	6.48×10^{-13}	1.31×10^{-11}

Tnfrsf14	1.34	1.94	77.18	7.12×10^{-13}	1.44×10^{-11}
Sav1	0.63	6.68	76.99	7.44×10^{-13}	1.50×10^{-11}
Bmpr1a	0.62	7.19	76.71	7.97×10^{-13}	1.60×10^{-11}
Psmg4	0.82	3.61	76.67	8.05×10^{-13}	1.62×10^{-11}
Stat3	0.64	7.08	76.61	8.16×10^{-13}	1.64×10^{-11}
Cpq	0.60	6.85	76.44	8.49×10^{-13}	1.70×10^{-11}
Oas1c	2.63	5.17	505.02	8.72×10^{-13}	1.74×10^{-11}
Ipo4	0.65	5.14	76.14	9.13×10^{-13}	1.81×10^{-11}
Fam167a	0.94	3.09	76.04	9.37×10^{-13}	1.85×10^{-11}
Abhd5	0.63	4.98	75.97	9.53×10^{-13}	1.88×10^{-11}
Sorbs1	0.62	5.82	75.93	9.62×10^{-13}	1.89×10^{-11}
AC139579.1	0.89	3.30	75.88	9.74×10^{-13}	1.91×10^{-11}
Gla	0.66	4.51	75.56	1.05×10^{-12}	2.06×10^{-11}
Orm1	1.05	2.89	76.25	1.17×10^{-12}	2.28×10^{-11}
Fcf1	0.64	4.69	75.06	1.19×10^{-12}	2.31×10^{-11}
Tbc1d31	0.64	5.21	74.29	1.43×10^{-12}	2.77×10^{-11}
Zfp217	0.71	5.41	81.34	1.51×10^{-12}	2.91×10^{-11}
Gm49134	1.44	2.40	92.17	1.57×10^{-12}	3.04×10^{-11}
Gm23201	0.79	4.31	83.55	1.68×10^{-12}	3.23×10^{-11}
Psd4	1.63	0.96	73.54	1.72×10^{-12}	3.31×10^{-11}
Gm27857	1.47	1.41	73.39	1.79×10^{-12}	3.42×10^{-11}
Mvb12a	0.63	5.80	73.21	1.87×10^{-12}	3.57×10^{-11}
H2.T24	5.39	2.74	522.96	2.00×10^{-12}	3.81×10^{-11}
Max	0.61	5.18	72.78	2.07×10^{-12}	3.94×10^{-11}
Gm16042	1.66	1.02	72.72	2.11×10^{-12}	4.00×10^{-11}
Atp11b	0.62	6.54	72.61	2.16×10^{-12}	4.10×10^{-11}
Mpc1	0.65	5.33	72.57	2.19×10^{-12}	4.14×10^{-11}
Ccn5	1.71	1.05	72.50	2.22×10^{-12}	4.20×10^{-11}
Gda	0.81	5.16	95.52	2.24×10^{-12}	4.23×10^{-11}
Gm6545	4.79	3.69	712.60	2.37×10^{-12}	4.45×10^{-11}
Mcur1	0.61	5.72	71.90	2.58×10^{-12}	4.84×10^{-11}
Rbck1	0.64	6.16	71.75	2.68×10^{-12}	5.01×10^{-11}
Lmo4	0.67	4.56	71.58	2.79×10^{-12}	5.22×10^{-11}
Rnf122	1.27	1.99	71.52	2.83×10^{-12}	5.29×10^{-11}
Dgke	0.96	2.86	71.49	2.86×10^{-12}	5.33×10^{-11}
Gemin5	0.61	5.90	71.23	3.05×10^{-12}	5.66×10^{-11}
Arl6ip1	0.59	7.75	71.00	3.23×10^{-12}	5.99×10^{-11}
Gm12248	1.17	2.34	69.96	4.20×10^{-12}	7.70×10^{-11}
Mfsd12	0.65	4.65	69.90	4.26×10^{-12}	7.79×10^{-11}
Kars	0.60	7.00	69.74	4.44×10^{-12}	8.11×10^{-11}
Irf2bpl	0.65	4.82	69.72	4.46×10^{-12}	8.13×10^{-11}
C4bp	0.83	5.53	99.85	5.01×10^{-12}	9.08×10^{-11}
Jun	0.63	5.86	69.33	5.28×10^{-12}	9.52×10^{-11}
Sfmbt1	0.61	4.66	68.54	6.01×10^{-12}	1.08×10^{-10}
Ccdc171	0.78	3.48	68.35	6.30×10^{-12}	1.13×10^{-10}
Gm28085	0.81	3.31	68.19	6.57×10^{-12}	1.18×10^{-10}
Nod1	1.07	5.45	140.68	6.59×10^{-12}	1.18×10^{-10}
Gm15337	1.27	3.74	131.53	6.62×10^{-12}	1.18×10^{-10}
Il7	1.87	3.60	211.99	7.11×10^{-12}	1.27×10^{-10}
Plagl2	0.65	4.29	67.75	7.35×10^{-12}	1.31×10^{-10}
Vcan	1.16	2.39	67.61	7.62×10^{-12}	1.36×10^{-10}
Rtn4ip1	0.64	4.26	67.24	8.39×10^{-12}	1.49×10^{-10}
Ube2v1	0.65	4.29	67.10	8.70×10^{-12}	1.54×10^{-10}
Sp110	8.25	0.84	281.75	9.38×10^{-12}	1.65×10^{-10}
Fut10	1.07	2.39	66.46	1.03×10^{-11}	1.80×10^{-10}
Uvrag	0.60	5.69	66.37	1.05×10^{-11}	1.84×10^{-10}

Gbp2	7.29	8.05	1499.77	1.06×10^{-11}	1.86×10^{-10}
Vrk2	0.63	4.34	65.95	1.17×10^{-11}	2.04×10^{-10}
Prkd2	0.77	3.48	65.59	1.29×10^{-11}	2.22×10^{-10}
Tcirg1	2.10	7.83	346.62	1.39×10^{-11}	2.38×10^{-10}
Pgghg	0.66	5.06	69.57	1.40×10^{-11}	2.39×10^{-10}
Cmpk2	9.31	7.38	1715.53	1.43×10^{-11}	2.45×10^{-10}
Spryd7	0.65	4.31	65.06	1.48×10^{-11}	2.52×10^{-10}
Bcar3	0.62	4.54	64.99	1.50×10^{-11}	2.56×10^{-10}
X4931422A03Rik	1.53	1.50	69.79	1.54×10^{-11}	2.62×10^{-10}
Tmem171	2.12	0.98	89.27	1.71×10^{-11}	2.90×10^{-10}
Tubb2a	0.60	5.11	64.49	1.72×10^{-11}	2.90×10^{-10}
Gm4951	8.90	5.30	1438.84	1.75×10^{-11}	2.96×10^{-10}
Arsb	0.60	4.81	64.26	1.82×10^{-11}	3.07×10^{-10}
Msantd2	0.61	4.90	64.22	1.84×10^{-11}	3.10×10^{-10}
Mpdz	0.86	3.21	64.02	1.94×10^{-11}	3.25×10^{-10}
Akr1c14	0.60	6.06	63.88	2.02×10^{-11}	3.36×10^{-10}
Ephb6	1.27	1.96	63.87	2.02×10^{-11}	3.36×10^{-10}
Zfp90	0.64	4.27	63.68	2.12×10^{-11}	3.53×10^{-10}
Plekhm3	0.76	3.34	63.53	2.21×10^{-11}	3.67×10^{-10}
Gm26799	0.80	3.31	63.39	2.29×10^{-11}	3.80×10^{-10}
Tnfsf10	8.04	5.52	1362.86	2.33×10^{-11}	3.87×10^{-10}
Ttc26	0.68	4.09	63.30	2.35×10^{-11}	3.88×10^{-10}
Dcp2	1.85	6.46	290.99	2.67×10^{-11}	4.39×10^{-10}
Gm26527	0.74	3.59	62.81	2.67×10^{-11}	4.39×10^{-10}
Slc10a7	0.62	4.83	62.24	3.11×10^{-11}	5.10×10^{-10}
Trib1	0.71	3.53	61.94	3.37×10^{-11}	5.49×10^{-10}
Washc4	0.81	7.07	92.81	3.55×10^{-11}	5.78×10^{-10}
Sh3glb1	0.70	7.35	75.82	3.57×10^{-11}	5.80×10^{-10}
Avl9	0.80	6.83	90.91	3.79×10^{-11}	6.13×10^{-10}
Thbs3	1.44	1.18	61.18	4.14×10^{-11}	6.69×10^{-10}
Nr3c2	0.62	4.21	60.94	4.42×10^{-11}	7.09×10^{-10}
Nat2	0.69	4.27	63.99	4.48×10^{-11}	7.18×10^{-10}
Ovol1	1.30	1.49	60.84	4.54×10^{-11}	7.26×10^{-10}
Bco2	1.35	1.94	66.96	4.57×10^{-11}	7.31×10^{-10}
Phf11a	9.10	1.58	358.85	5.73×10^{-11}	9.05×10^{-10}
Gm16675	1.56	3.22	135.25	6.60×10^{-11}	1.04×10^{-09}
Apol9a	9.02	6.13	1396.29	7.01×10^{-11}	1.10×10^{-09}
Gm35330	1.17	2.51	68.51	7.48×10^{-11}	1.16×10^{-09}
Gm11842	1.41	1.16	58.98	7.52×10^{-11}	1.17×10^{-09}
Gm20489	0.65	4.89	64.70	7.94×10^{-11}	1.23×10^{-09}
Tmem106a	2.85	4.44	433.36	8.08×10^{-11}	1.25×10^{-09}
Fanca	1.02	4.18	103.26	8.39×10^{-11}	1.30×10^{-09}
Tbc1d9	0.70	3.64	58.51	8.56×10^{-11}	1.32×10^{-09}
Samd9l	3.64	9.58	626.83	9.26×10^{-11}	1.43×10^{-09}
Il2rg	0.64	4.87	63.71	9.45×10^{-11}	1.45×10^{-09}
Pitpnm1	0.65	3.89	57.67	1.08×10^{-10}	1.64×10^{-09}
Gm20319	1.21	2.48	68.57	1.19×10^{-10}	1.80×10^{-09}
Gm12250	8.04	5.72	1240.80	1.20×10^{-10}	1.82×10^{-09}
Lrrc32	1.24	1.67	57.19	1.23×10^{-10}	1.86×10^{-09}
Spty2d1	0.71	6.28	75.86	1.26×10^{-10}	1.89×10^{-09}
Pmf1	0.84	2.96	56.85	1.36×10^{-10}	2.03×10^{-09}
Nlgn2	2.46	6.36	395.55	1.38×10^{-10}	2.06×10^{-09}
Phf11d	9.55	6.29	1333.43	1.42×10^{-10}	2.11×10^{-09}
Ecscr	1.26	1.63	56.48	1.50×10^{-10}	2.23×10^{-09}
Mndal	8.18	9.60	1303.85	1.58×10^{-10}	2.34×10^{-09}
Sdc3	1.11	2.01	56.08	1.68×10^{-10}	2.48×10^{-09}

Trim46	0.72	3.46	55.97	1.74×10^{-10}	2.54×10^{-09}
Akr1c20	4.74	3.50	543.25	1.86×10^{-10}	2.72×10^{-09}
Zfas1	0.78	6.87	84.98	1.97×10^{-10}	2.87×10^{-09}
Emc9	0.67	3.88	55.29	2.10×10^{-10}	3.05×10^{-09}
Vrk1	0.66	3.85	55.08	2.23×10^{-10}	3.23×10^{-09}
Nrg1	0.82	3.69	66.23	2.59×10^{-10}	3.72×10^{-09}
B4galt5	0.72	5.02	72.70	2.81×10^{-10}	4.03×10^{-09}
Ube2l6	6.94	6.11	1071.21	2.87×10^{-10}	4.12×10^{-09}
Slco3a1	0.96	8.11	106.45	2.97×10^{-10}	4.24×10^{-09}
Tent5d	1.00	2.28	53.66	3.35×10^{-10}	4.77×10^{-09}
X4930404N11Rik	0.65	3.87	53.64	3.37×10^{-10}	4.80×10^{-09}
Enkur	1.33	1.18	53.23	3.79×10^{-10}	5.37×10^{-09}
F11r	0.75	7.55	78.34	3.87×10^{-10}	5.48×10^{-09}
Gbp7	8.21	7.54	1110.66	4.41×10^{-10}	6.19×10^{-09}
Zfp74	0.60	4.08	52.52	4.65×10^{-10}	6.51×10^{-09}
Ifi207	8.60	7.22	1079.77	5.30×10^{-10}	7.38×10^{-09}
Sp100	11.60	4.57	993.31	5.33×10^{-10}	7.41×10^{-09}
Isg15	8.85	7.91	1080.34	5.44×10^{-10}	7.55×10^{-09}
C4b	3.46	8.24	545.28	5.64×10^{-10}	7.80×10^{-09}
Gm48194	1.06	2.07	51.74	5.84×10^{-10}	8.06×10^{-09}
Dhrs9	0.81	2.95	51.68	5.93×10^{-10}	8.18×10^{-09}
Cd274	5.76	6.29	876.78	6.46×10^{-10}	8.85×10^{-09}
Ly6a	6.78	6.86	973.90	6.77×10^{-10}	9.22×10^{-09}
Gm2619	7.10	1.32	262.60	7.35×10^{-10}	9.95×10^{-09}
Asgr2	0.79	2.97	50.92	7.41×10^{-10}	1.00×10^{-08}
Atp10a	3.45	4.54	488.19	7.51×10^{-10}	1.01×10^{-08}
A730011C13Rik	0.72	3.26	50.72	7.86×10^{-10}	1.06×10^{-08}
Fam49b	0.60	5.78	58.11	8.04×10^{-10}	1.08×10^{-08}
Gbp10	8.36	6.98	974.67	8.30×10^{-10}	1.11×10^{-08}
Zfyve26	0.75	4.50	70.09	8.48×10^{-10}	1.13×10^{-08}
Gna13	0.62	6.93	59.69	9.26×10^{-10}	1.23×10^{-08}
Gm47248	4.77	3.49	483.44	9.30×10^{-10}	1.23×10^{-08}
Gm10701	0.62	4.22	51.58	9.74×10^{-10}	1.29×10^{-08}
Mpeg1	8.59	7.51	937.63	1.00×10^{-09}	1.32×10^{-08}
Casp12	2.89	3.88	328.61	1.00×10^{-09}	1.32×10^{-08}
Gm17111	0.78	3.09	49.76	1.04×10^{-09}	1.37×10^{-08}
Gm16549	1.01	2.21	49.48	1.13×10^{-09}	1.48×10^{-08}
Zfp324	0.64	3.94	49.43	1.15×10^{-09}	1.50×10^{-08}
Rusc1	0.65	3.46	49.23	1.22×10^{-09}	1.59×10^{-08}
X9930014A18Rik	0.67	3.67	49.18	1.24×10^{-09}	1.61×10^{-08}
Ifit1	9.81	10.51	886.83	1.27×10^{-09}	1.65×10^{-08}
Ccl5	10.15	4.53	837.21	1.31×10^{-09}	1.70×10^{-08}
Oas2	11.37	6.13	873.72	1.34×10^{-09}	1.74×10^{-08}
BC064078	1.02	2.11	48.85	1.37×10^{-09}	1.77×10^{-08}
X1700066B19Rik	1.13	1.80	48.69	1.44×10^{-09}	1.84×10^{-08}
Tmem170b	0.79	4.25	69.34	1.48×10^{-09}	1.89×10^{-08}
Fmr1	0.93	6.87	98.96	1.53×10^{-09}	1.95×10^{-08}
Zfp455	1.00	2.19	48.35	1.59×10^{-09}	2.02×10^{-08}
Syt7	1.60	2.52	90.95	1.61×10^{-09}	2.05×10^{-08}
Zbp1	9.73	6.67	825.21	1.70×10^{-09}	2.14×10^{-08}
Gm43302	7.19	7.90	818.30	1.72×10^{-09}	2.16×10^{-08}
Klf2	1.34	0.96	47.91	1.82×10^{-09}	2.28×10^{-08}
Ifi203	8.48	9.08	802.61	1.90×10^{-09}	2.37×10^{-08}
Gbp3	11.06	6.78	801.65	1.91×10^{-09}	2.38×10^{-08}
Gbp11	7.72	5.22	787.43	1.92×10^{-09}	2.39×10^{-08}
X1700034P13Rik	0.68	3.49	47.69	1.94×10^{-09}	2.41×10^{-08}

Ccnyl1	0.62	4.29	51.68	2.69×10^{-09}	3.29×10^{-08}
Arl6	0.64	3.64	46.56	2.73×10^{-09}	3.33×10^{-08}
C4a	0.98	2.22	46.05	3.19×10^{-09}	3.85×10^{-08}
Iigp1	9.34	8.41	705.52	3.20×10^{-09}	3.86×10^{-08}
Ifit1bl1	8.17	5.67	696.97	3.36×10^{-09}	4.04×10^{-08}
Ifit3	10.28	9.13	697.03	3.36×10^{-09}	4.04×10^{-08}
Oasl1	6.47	6.91	693.52	3.39×10^{-09}	4.06×10^{-08}
Hap1	6.45	4.88	670.74	3.55×10^{-09}	4.25×10^{-08}
Gm20547	2.14	10.22	260.71	3.64×10^{-09}	4.35×10^{-08}
Rps2.ps9	0.91	2.29	45.51	3.77×10^{-09}	4.48×10^{-08}
Zyx	0.62	6.55	57.65	3.91×10^{-09}	4.64×10^{-08}
Zc3h7a	0.63	6.92	58.68	4.15×10^{-09}	4.92×10^{-08}
Oas1g	11.49	5.52	656.28	4.29×10^{-09}	5.08×10^{-08}
Cnot6l	1.04	7.05	108.87	4.49×10^{-09}	5.27×10^{-08}
Ap5b1	0.74	3.15	44.71	4.83×10^{-09}	5.63×10^{-08}
Oasl2	9.85	8.00	633.51	4.95×10^{-09}	5.75×10^{-08}
Ifi203.ps	9.71	5.50	629.65	5.07×10^{-09}	5.88×10^{-08}
Gadd45g	0.78	5.07	72.43	5.34×10^{-09}	6.18×10^{-08}
Oas1a	11.06	6.20	621.27	5.36×10^{-09}	6.19×10^{-08}
Cyp4v3	1.12	4.20	100.75	5.55×10^{-09}	6.42×10^{-08}
Gbp8	5.17	9.35	595.91	5.75×10^{-09}	6.62×10^{-08}
Angptl6	0.81	2.66	43.87	6.26×10^{-09}	7.17×10^{-08}
Gbp6	6.48	6.83	592.14	6.49×10^{-09}	7.39×10^{-08}
Gm12764	0.88	2.48	43.70	6.60×10^{-09}	7.52×10^{-08}
Rnaset2a	0.69	3.23	43.36	7.35×10^{-09}	8.33×10^{-08}
Rnf152	0.85	3.91	66.16	7.36×10^{-09}	8.33×10^{-08}
Gm49673	0.69	3.23	43.24	7.63×10^{-09}	8.63×10^{-08}
Gm37893	0.84	2.63	43.22	7.68×10^{-09}	8.66×10^{-08}
Mx1	10.32	8.08	565.33	7.84×10^{-09}	8.83×10^{-08}
Meiob	2.96	4.64	355.85	8.17×10^{-09}	9.17×10^{-08}
Lrrk1	0.59	5.97	52.71	8.30×10^{-09}	9.29×10^{-08}
Gbp9	5.28	9.15	543.68	8.94×10^{-09}	9.98×10^{-08}
Phf11b	10.03	3.69	527.22	9.82×10^{-09}	1.09×10^{-07}
Gm31718	0.93	2.16	42.23	1.05×10^{-08}	1.15×10^{-07}
Stc2	0.59	3.77	41.86	1.18×10^{-08}	1.29×10^{-07}
X9330179D12Rik	0.77	2.78	41.73	1.23×10^{-08}	1.34×10^{-07}
Casp1	3.48	0.98	120.78	1.26×10^{-08}	1.37×10^{-07}
Tsacc	0.86	4.16	69.95	1.27×10^{-08}	1.38×10^{-07}
Ifit3b	10.74	9.89	498.17	1.30×10^{-08}	1.41×10^{-07}
Trim30a	10.42	8.61	494.24	1.35×10^{-08}	1.45×10^{-07}
Rttn	0.61	3.38	41.22	1.45×10^{-08}	1.55×10^{-07}
Gm4841	12.39	4.11	483.69	1.47×10^{-08}	1.57×10^{-07}
Sult6b1	1.10	1.58	40.91	1.60×10^{-08}	1.70×10^{-07}
Wdpcp	0.59	3.62	40.69	1.72×10^{-08}	1.82×10^{-07}
BC023105	10.47	2.92	420.94	1.82×10^{-08}	1.92×10^{-07}
Tmem236	1.21	1.39	43.12	1.98×10^{-08}	2.08×10^{-07}
Gm38392	0.78	4.26	62.32	2.04×10^{-08}	2.13×10^{-07}
Scn8a	0.76	2.68	40.11	2.07×10^{-08}	2.17×10^{-07}
Rbm41	0.64	4.06	46.86	2.16×10^{-08}	2.25×10^{-07}
Igsf8	0.64	5.95	56.39	2.33×10^{-08}	2.42×10^{-07}
Rnf150	0.64	3.12	39.53	2.50×10^{-08}	2.58×10^{-07}
A330040F15Rik	3.76	5.46	403.63	2.53×10^{-08}	2.61×10^{-07}
Gm19918	0.59	3.53	39.31	2.69×10^{-08}	2.77×10^{-07}
Mkx	0.77	2.66	39.24	2.75×10^{-08}	2.83×10^{-07}
Gm8995	12.17	4.56	412.36	2.79×10^{-08}	2.86×10^{-07}
Rpe	0.64	6.87	55.76	2.91×10^{-08}	2.98×10^{-07}

Gm28809	0.89	2.13	38.69	3.29×10^{-08}	3.34×10^{-07}
Rab21	0.84	6.51	75.77	3.49×10^{-08}	3.51×10^{-07}
Rgl1	0.81	2.63	38.74	3.51×10^{-08}	3.54×10^{-07}
Snord12	0.83	5.40	73.50	3.72×10^{-08}	3.73×10^{-07}
Rab29	0.67	3.19	38.07	4.06×10^{-08}	4.03×10^{-07}
X2700069I18Rik	1.16	1.02	38.06	4.06×10^{-08}	4.03×10^{-07}
Zfp976	0.63	3.36	38.13	4.42×10^{-08}	4.36×10^{-07}
Gbp2b	8.69	4.26	352.60	5.22×10^{-08}	5.09×10^{-07}
Zgrf1	0.97	1.68	37.28	5.26×10^{-08}	5.12×10^{-07}
Gbp4	6.98	7.29	346.84	5.58×10^{-08}	5.40×10^{-07}
Gm4117	0.62	3.27	36.66	6.47×10^{-08}	6.21×10^{-07}
X1700039E22Rik	1.09	2.06	43.07	6.77×10^{-08}	6.46×10^{-07}
Cybb	3.75	7.88	325.53	7.06×10^{-08}	6.73×10^{-07}
Pdp1	0.67	2.89	36.22	7.49×10^{-08}	7.11×10^{-07}
Mvp	0.62	6.78	50.97	7.51×10^{-08}	7.12×10^{-07}
Mfhas1	0.66	3.74	42.36	8.94×10^{-08}	8.35×10^{-07}
Tmem184a	0.68	2.80	35.57	9.33×10^{-08}	8.68×10^{-07}
Gm19144	0.90	1.84	35.12	1.09×10^{-07}	1.00×10^{-06}
Gm17089	1.04	1.38	35.02	1.12×10^{-07}	1.03×10^{-06}
C130074G19Rik	0.70	7.25	57.03	1.22×10^{-07}	1.11×10^{-06}
Gm19221	0.64	3.01	34.44	1.37×10^{-07}	1.24×10^{-06}
A730085K08Rik	0.66	2.81	34.40	1.39×10^{-07}	1.26×10^{-06}
Mtcp1	0.64	4.25	45.31	1.58×10^{-07}	1.41×10^{-06}
Cd276	0.59	3.34	33.85	1.68×10^{-07}	1.49×10^{-06}
Fut8	0.59	3.15	33.73	1.75×10^{-07}	1.55×10^{-06}
Slc7a11	1.38	1.26	43.02	1.80×10^{-07}	1.59×10^{-06}
Cxcl10	11.28	8.78	251.82	2.00×10^{-07}	1.75×10^{-06}
Riox2	0.69	2.67	33.30	2.03×10^{-07}	1.79×10^{-06}
Sp140	2.81	2.53	134.11	2.20×10^{-07}	1.90×10^{-06}
Rsad2	12.59	9.37	244.76	2.24×10^{-07}	1.93×10^{-06}
Slc25a22	1.42	5.87	127.38	2.28×10^{-07}	1.96×10^{-06}
Map3k1	0.63	6.68	49.63	2.31×10^{-07}	1.99×10^{-06}
Enpp4	3.24	7.11	240.36	2.39×10^{-07}	2.05×10^{-06}
Mppe1	0.60	4.79	44.58	2.40×10^{-07}	2.06×10^{-06}
Trp53inp1	1.03	8.55	84.64	2.41×10^{-07}	2.07×10^{-06}
Il34	0.60	3.04	32.70	2.50×10^{-07}	2.14×10^{-06}
Ccdc88a	0.59	5.42	45.19	2.73×10^{-07}	2.32×10^{-06}
Enox1	0.82	2.07	32.42	2.76×10^{-07}	2.35×10^{-06}
Art3	11.09	9.08	231.73	2.78×10^{-07}	2.36×10^{-06}
Acss2os	0.67	2.75	32.31	2.87×10^{-07}	2.42×10^{-06}
Aqp9	1.04	1.11	32.31	2.87×10^{-07}	2.42×10^{-06}
Stxbp1	1.70	5.19	146.65	3.26×10^{-07}	2.73×10^{-06}
X1700016L21Rik	1.30	0.57	35.53	3.53×10^{-07}	2.92×10^{-06}
Gm47260	0.79	2.23	31.40	3.96×10^{-07}	3.24×10^{-06}
Glrp1	2.14	3.21	125.94	4.20×10^{-07}	3.42×10^{-06}
Exph5	1.40	2.79	67.02	4.34×10^{-07}	3.54×10^{-06}
Stk40	0.67	4.21	44.27	4.49×10^{-07}	3.63×10^{-06}
F830016B08Rik	8.93	2.56	201.08	4.85×10^{-07}	3.89×10^{-06}
Gm28347	6.35	0.47	121.67	5.36×10^{-07}	4.27×10^{-06}
Ltbp1	0.71	2.51	30.55	5.36×10^{-07}	4.27×10^{-06}
Glcci1	1.56	3.09	82.38	5.87×10^{-07}	4.65×10^{-06}
Cfh	0.89	2.29	33.83	5.91×10^{-07}	4.68×10^{-06}
D16Ertd472e	1.00	1.16	30.03	6.45×10^{-07}	5.07×10^{-06}
Sinhcaf	0.65	2.87	29.89	6.78×10^{-07}	5.30×10^{-06}
Socs6	0.69	5.72	50.83	7.81×10^{-07}	6.03×10^{-06}
Arhgap10	0.68	2.57	29.37	8.19×10^{-07}	6.31×10^{-06}

Cnnm4	0.60	2.91	29.18	8.78×10^{-07}	6.70×10^{-06}
G630064G18Rik	0.70	2.44	29.08	9.09×10^{-07}	6.92×10^{-06}
Ccdc173	0.62	2.81	29.02	9.28×10^{-07}	7.05×10^{-06}
AI429214	0.71	2.40	28.87	9.80×10^{-07}	7.40×10^{-06}
Il17ra	0.63	2.77	28.71	1.04×10^{-06}	7.82×10^{-06}
Iqck	0.59	3.01	28.69	1.05×10^{-06}	7.87×10^{-06}
Ly96	0.95	1.64	29.95	1.11×10^{-06}	8.27×10^{-06}
AW112010	3.76	8.18	161.72	1.14×10^{-06}	8.47×10^{-06}
Atf3	2.70	4.30	159.46	1.17×10^{-06}	8.64×10^{-06}
Fbxo33	0.69	3.89	40.08	1.17×10^{-06}	8.64×10^{-06}
Ext1	1.32	7.16	102.07	1.18×10^{-06}	8.70×10^{-06}
Mir17hg	0.65	2.64	28.34	1.19×10^{-06}	8.78×10^{-06}
Sfxn2	1.21	6.65	92.83	1.21×10^{-06}	8.87×10^{-06}
Tead4	0.69	4.30	44.63	1.22×10^{-06}	8.95×10^{-06}
Hnmt	0.73	2.40	28.12	1.29×10^{-06}	9.44×10^{-06}
Tut4	1.12	6.53	84.94	1.30×10^{-06}	9.52×10^{-06}
Gm31166	0.68	2.40	27.87	1.41×10^{-06}	1.02×10^{-05}
X9530082P21Rik	1.96	5.01	140.09	1.43×10^{-06}	1.03×10^{-05}
Marcksl1	0.59	3.29	29.94	1.43×10^{-06}	1.04×10^{-05}
Asap3	2.80	4.06	141.72	1.89×10^{-06}	1.34×10^{-05}
Lrrc49	0.73	2.17	27.03	1.93×10^{-06}	1.37×10^{-05}
Cxcl11	10.83	6.59	139.65	2.02×10^{-06}	1.42×10^{-05}
Gm45857	0.75	2.04	26.50	2.35×10^{-06}	1.63×10^{-05}
Tbc1d1	1.24	5.81	89.46	2.36×10^{-06}	1.64×10^{-05}
Gm24718	0.74	2.03	26.43	2.42×10^{-06}	1.67×10^{-05}
Gins1	0.80	1.70	26.40	2.44×10^{-06}	1.69×10^{-05}
Ccrl2	8.78	4.53	130.76	2.61×10^{-06}	1.80×10^{-05}
Traf5	0.64	2.53	25.83	3.03×10^{-06}	2.06×10^{-05}
Rbms1	0.77	6.47	52.55	3.05×10^{-06}	2.06×10^{-05}
H2.Q10	2.82	3.17	118.94	3.12×10^{-06}	2.11×10^{-05}
Dnah7b	0.65	2.43	25.71	3.17×10^{-06}	2.14×10^{-05}
Gm2350	0.81	1.69	25.44	3.51×10^{-06}	2.34×10^{-05}
Cxcl9	8.73	6.48	120.52	3.58×10^{-06}	2.38×10^{-05}
Gm45449	0.71	2.06	25.36	3.61×10^{-06}	2.40×10^{-05}
Gm37116	0.86	1.31	25.29	3.71×10^{-06}	2.46×10^{-05}
Pax3	0.68	2.34	25.29	3.71×10^{-06}	2.46×10^{-05}
Pi4k2b	0.73	6.21	48.86	3.76×10^{-06}	2.49×10^{-05}
Ap1s3	0.73	2.09	25.22	3.81×10^{-06}	2.52×10^{-05}
Gm9869	5.67	0.83	101.68	3.95×10^{-06}	2.60×10^{-05}
Hmga1	0.97	4.81	61.67	4.12×10^{-06}	2.71×10^{-05}
Gm48427	1.73	1.47	48.08	4.24×10^{-06}	2.78×10^{-05}
X4930471E19Rik	0.82	1.49	24.85	4.40×10^{-06}	2.87×10^{-05}
X1200007C13Rik	1.15	1.17	29.55	4.90×10^{-06}	3.17×10^{-05}
Lmcd1	0.77	1.88	24.19	5.64×10^{-06}	3.61×10^{-05}
Il33	0.72	3.83	37.65	5.90×10^{-06}	3.76×10^{-05}
Cyp4a12a	2.26	3.07	92.78	6.72×10^{-06}	4.23×10^{-05}
Gm15564	2.63	6.67	102.14	6.74×10^{-06}	4.24×10^{-05}
Ccnf	0.77	1.79	23.44	7.55×10^{-06}	4.71×10^{-05}
C78334	0.62	2.54	23.40	7.66×10^{-06}	4.77×10^{-05}
Adap1	0.67	2.63	24.87	7.81×10^{-06}	4.85×10^{-05}
Arhgap8	2.06	3.22	86.86	7.89×10^{-06}	4.90×10^{-05}
Mir6972	2.17	5.40	96.90	8.24×10^{-06}	5.08×10^{-05}
Cfap47	0.80	1.66	23.10	8.61×10^{-06}	5.30×10^{-05}
Nav1	0.60	2.75	23.01	8.93×10^{-06}	5.47×10^{-05}
Gm42507	0.63	2.51	22.97	9.08×10^{-06}	5.55×10^{-05}
Cmc4	0.61	4.86	36.03	9.30×10^{-06}	5.67×10^{-05}

Cfb	1.97	9.67	93.53	9.37×10^{-06}	5.71×10^{-05}
Gm33782	1.86	1.58	49.22	9.58×10^{-06}	5.83×10^{-05}
Vnn1	3.31	4.35	92.71	9.74×10^{-06}	5.91×10^{-05}
Hist1h1c	0.72	6.13	43.87	9.87×10^{-06}	5.98×10^{-05}
Gm45481	0.90	1.07	22.73	9.95×10^{-06}	6.02×10^{-05}
Pde4a	0.60	2.59	22.60	1.05×10^{-05}	6.32×10^{-05}
Gm35876	0.66	2.25	22.51	1.08×10^{-05}	6.51×10^{-05}
Crybg3	0.72	1.79	22.39	1.14×10^{-05}	6.80×10^{-05}
Rapgef5	1.20	5.65	72.23	1.14×10^{-05}	6.82×10^{-05}
Nab1	0.99	6.12	60.06	1.21×10^{-05}	7.16×10^{-05}
Gm22858	0.84	1.23	21.95	1.35×10^{-05}	7.91×10^{-05}
Mastl	0.59	2.58	21.95	1.35×10^{-05}	7.92×10^{-05}
Rassf9	0.62	5.66	37.09	1.37×10^{-05}	8.04×10^{-05}
Gbp5	10.19	5.61	84.32	1.39×10^{-05}	8.14×10^{-05}
X2310001H17Rik	1.19	5.53	69.24	1.43×10^{-05}	8.35×10^{-05}
Vnn3	5.42	4.15	81.42	1.59×10^{-05}	9.17×10^{-05}
Daam1	1.18	4.89	65.30	1.63×10^{-05}	9.40×10^{-05}
Il13ra1	1.09	7.38	62.83	1.65×10^{-05}	9.49×10^{-05}
Ppp1r15a	1.83	5.77	79.66	1.72×10^{-05}	9.86×10^{-05}
Brip1	0.81	1.48	21.34	1.72×10^{-05}	9.87×10^{-05}
Npr3	0.81	1.26	21.29	1.76×10^{-05}	1.00×10^{-04}
Hcar2	8.01	1.95	78.25	1.84×10^{-05}	1.05×10^{-04}
Osmr	2.92	5.39	78.23	1.85×10^{-05}	1.05×10^{-04}
Ms4a4b	3.59	2.38	77.51	1.90×10^{-05}	1.08×10^{-04}
Rgs19	0.83	1.23	20.92	2.04×10^{-05}	1.14×10^{-04}
Gm43980	0.69	3.28	28.90	2.08×10^{-05}	1.16×10^{-04}
Gm20470	0.78	3.68	35.08	2.13×10^{-05}	1.19×10^{-04}
Ccl7	6.19	2.32	74.94	2.17×10^{-05}	1.21×10^{-04}
Gm25405	9.44	2.39	130.76	2.21×10^{-05}	1.23×10^{-04}
Acod1	7.40	3.15	74.27	2.24×10^{-05}	1.24×10^{-04}
Csrp1	1.19	6.56	65.41	2.26×10^{-05}	1.25×10^{-04}
Il3ra	0.59	2.52	20.58	2.34×10^{-05}	1.29×10^{-04}
Gm26574	0.64	2.17	20.43	2.48×10^{-05}	1.37×10^{-04}
Mansc1	0.82	1.21	20.41	2.51×10^{-05}	1.38×10^{-04}
Dusp28	1.04	4.25	51.30	2.58×10^{-05}	1.41×10^{-04}
Etv6	0.97	6.18	53.94	2.62×10^{-05}	1.43×10^{-04}
Gm43791	0.73	1.75	20.25	2.67×10^{-05}	1.46×10^{-04}
Eml6	0.66	1.99	20.21	2.72×10^{-05}	1.48×10^{-04}
X4930592A05Rik	0.85	4.85	45.28	2.72×10^{-05}	1.48×10^{-04}
Ubd	4.30	2.75	70.18	2.76×10^{-05}	1.50×10^{-04}
BE692007	5.42	1.14	68.55	3.01×10^{-05}	1.62×10^{-04}
Zeb2	1.15	1.72	28.71	3.10×10^{-05}	1.66×10^{-04}
Gm8369	5.86	1.12	66.83	3.31×10^{-05}	1.77×10^{-04}
Mir3473g	0.77	1.31	19.72	3.31×10^{-05}	1.77×10^{-04}
Mir6953	0.70	1.86	19.54	3.57×10^{-05}	1.90×10^{-04}
Itpkb	0.75	2.10	21.19	3.62×10^{-05}	1.92×10^{-04}
Xkr9	0.95	5.72	50.00	3.80×10^{-05}	2.00×10^{-04}
Irf1	2.13	7.97	64.06	3.87×10^{-05}	2.03×10^{-04}
Garnl3	0.78	1.28	19.11	4.24×10^{-05}	2.21×10^{-04}
Ms4a6c	5.56	0.88	62.39	4.26×10^{-05}	2.22×10^{-04}
Nemp2	0.67	1.91	19.05	4.36×10^{-05}	2.26×10^{-04}
Eif5a2	0.75	1.31	18.89	4.65×10^{-05}	2.41×10^{-04}
Pax2	1.16	5.48	55.50	5.09×10^{-05}	2.60×10^{-04}
Gm6627	0.66	1.80	18.61	5.22×10^{-05}	2.66×10^{-04}
Gm2164	1.66	3.34	56.38	5.37×10^{-05}	2.72×10^{-04}
Myom3	0.74	2.37	21.58	5.48×10^{-05}	2.78×10^{-04}

Lrmda	0.65	1.92	18.43	5.61×10^{-05}	2.83×10^{-04}
Hhat	0.71	1.37	18.38	5.75×10^{-05}	2.89×10^{-04}
mt.Rnr2	0.84	9.82	40.48	6.14×10^{-05}	3.07×10^{-04}
Nucb2	1.04	5.23	49.01	6.56×10^{-05}	3.26×10^{-04}
Uaca	2.59	5.65	55.14	6.67×10^{-05}	3.30×10^{-04}
Ifrd1	0.62	5.54	31.30	7.01×10^{-05}	3.45×10^{-04}
Gm26538	0.59	2.20	17.86	7.13×10^{-05}	3.51×10^{-04}
Casp4	4.92	3.87	53.62	7.38×10^{-05}	3.62×10^{-04}
Trdmt1	0.61	2.10	17.74	7.50×10^{-05}	3.67×10^{-04}
Rgs17	0.67	1.61	17.71	7.58×10^{-05}	3.71×10^{-04}
X2810457G06Rik	0.81	0.98	17.52	8.22×10^{-05}	3.99×10^{-04}
Nupr1	1.32	2.33	34.15	8.24×10^{-05}	4.00×10^{-04}
Alpk1	1.47	4.06	51.10	8.50×10^{-05}	4.12×10^{-04}
Gm23205	0.71	1.42	17.43	8.54×10^{-05}	4.14×10^{-04}
Gm25878	0.72	1.91	18.20	9.91×10^{-05}	4.72×10^{-04}
Gm47015	0.68	1.51	16.59	1.22×10^{-04}	5.72×10^{-04}
Kansl1l	0.65	6.67	30.73	1.28×10^{-04}	6.00×10^{-04}
Brinp3	1.65	2.05	35.58	1.36×10^{-04}	6.30×10^{-04}
Il1rn	3.00	0.92	43.62	1.37×10^{-04}	6.35×10^{-04}
Cchcr1	0.59	3.33	21.29	1.42×10^{-04}	6.54×10^{-04}
Acnat2	0.68	1.49	16.23	1.42×10^{-04}	6.55×10^{-04}
Otud1	0.62	1.90	16.16	1.46×10^{-04}	6.71×10^{-04}
Tlr2	5.87	5.54	43.84	1.51×10^{-04}	6.92×10^{-04}
Mier1	0.71	6.02	32.38	1.55×10^{-04}	7.06×10^{-04}
Ank1	0.74	1.28	16.00	1.56×10^{-04}	7.13×10^{-04}
Plekhs1	3.28	2.46	43.37	1.57×10^{-04}	7.14×10^{-04}
Gm47218	0.70	1.33	15.97	1.58×10^{-04}	7.22×10^{-04}
Ldb3	0.63	1.63	15.87	1.65×10^{-04}	7.49×10^{-04}
Arhgap24	1.01	4.57	39.76	1.67×10^{-04}	7.55×10^{-04}
Snx10	0.86	7.07	36.83	1.78×10^{-04}	8.01×10^{-04}
C430019N01Rik	0.63	1.82	15.63	1.83×10^{-04}	8.23×10^{-04}
Ssc4d	0.75	2.02	18.16	1.85×10^{-04}	8.29×10^{-04}
Lpar6	0.68	5.73	30.01	1.95×10^{-04}	8.69×10^{-04}
Mok	0.67	1.48	15.42	2.01×10^{-04}	8.92×10^{-04}
Zfp945	1.36	5.22	39.73	2.12×10^{-04}	9.39×10^{-04}
Peg10	0.69	1.32	15.18	2.23×10^{-04}	9.79×10^{-04}
Gm17334	2.05	3.49	39.07	2.25×10^{-04}	9.85×10^{-04}
Gm16567	0.77	2.17	18.68	2.28×10^{-04}	9.96×10^{-04}
Slc10a2	1.06	6.29	38.34	2.33×10^{-04}	1.01×10^{-03}
Gm44867	4.60	1.82	37.81	2.52×10^{-04}	1.08×10^{-03}
Clic4	1.11	7.08	37.63	2.53×10^{-04}	1.09×10^{-03}
Mylip	0.70	1.15	14.83	2.60×10^{-04}	1.11×10^{-03}
Gm49392	0.70	1.00	14.76	2.67×10^{-04}	1.14×10^{-03}
Snai2	1.26	3.25	34.63	2.76×10^{-04}	1.17×10^{-03}
Gdf15	1.28	3.37	35.06	2.83×10^{-04}	1.20×10^{-03}
Col11a2	0.73	3.58	24.22	2.92×10^{-04}	1.23×10^{-03}
C1s1	0.80	2.40	19.43	3.13×10^{-04}	1.31×10^{-03}
Katna1	0.73	4.17	26.68	3.17×10^{-04}	1.33×10^{-03}
Plscr2	0.95	8.71	32.99	3.67×10^{-04}	1.51×10^{-03}
Abtb2	1.35	3.82	33.48	3.79×10^{-04}	1.55×10^{-03}
Il5ra	0.63	5.31	25.30	3.89×10^{-04}	1.59×10^{-03}
Dusp22	1.18	5.10	32.72	4.11×10^{-04}	1.67×10^{-03}
Creb5	1.24	3.26	31.41	4.16×10^{-04}	1.69×10^{-03}
Gm13212	1.28	2.39	26.72	4.18×10^{-04}	1.69×10^{-03}
Il15	1.88	3.51	32.53	4.19×10^{-04}	1.70×10^{-03}
Epha7	1.02	4.70	32.19	4.21×10^{-04}	1.70×10^{-03}

Runx2	0.62	5.03	23.47	5.15×10^{-04}	2.04×10^{-03}
Csf1	2.55	7.33	29.89	5.55×10^{-04}	2.18×10^{-03}
Ch25h	6.58	0.92	29.81	5.60×10^{-04}	2.19×10^{-03}
Ube2e1	0.75	5.46	27.04	5.64×10^{-04}	2.20×10^{-03}
Spdl1	0.64	1.54	12.89	6.78×10^{-04}	2.60×10^{-03}
Egr1	1.47	6.12	27.98	6.89×10^{-04}	2.63×10^{-03}
Gprc5b	0.61	5.05	22.20	7.09×10^{-04}	2.70×10^{-03}
Dock11	1.18	1.05	17.95	7.50×10^{-04}	2.83×10^{-03}
Cd2ap	0.60	8.63	21.60	7.54×10^{-04}	2.85×10^{-03}
Gm20512	0.84	4.38	25.45	8.00×10^{-04}	3.00×10^{-03}
Atxn7l1os2	0.69	3.38	19.28	8.01×10^{-04}	3.00×10^{-03}
Kpna3	0.82	6.62	25.83	8.34×10^{-04}	3.11×10^{-03}
Gm26763	1.29	2.29	23.11	8.43×10^{-04}	3.13×10^{-03}
Gm7340	0.71	1.29	12.76	9.34×10^{-04}	3.44×10^{-03}
Anxa6	0.65	6.81	22.29	9.74×10^{-04}	3.56×10^{-03}
Atxn7l1	0.86	3.90	23.12	1.03×10^{-03}	3.74×10^{-03}
Gem	3.75	−0.09	24.34	1.07×10^{-03}	3.87×10^{-03}
Map3k8	3.01	2.41	24.16	1.10×10^{-03}	3.95×10^{-03}
Neurl3	3.48	6.16	23.70	1.17×10^{-03}	4.17×10^{-03}
Fos	2.65	2.94	23.69	1.17×10^{-03}	4.17×10^{-03}
Tnfaip8	1.31	2.52	22.29	1.24×10^{-03}	4.38×10^{-03}
Hpx	0.73	7.37	22.32	1.25×10^{-03}	4.40×10^{-03}
Jak2	1.14	8.12	23.23	1.25×10^{-03}	4.40×10^{-03}
Ptbp3	0.67	7.40	21.30	1.29×10^{-03}	4.53×10^{-03}
Ccl2	3.94	8.69	22.89	1.30×10^{-03}	4.57×10^{-03}
Gm17268	3.93	8.69	22.89	1.31×10^{-03}	4.57×10^{-03}
Ccnd3	0.73	7.01	21.50	1.46×10^{-03}	5.03×10^{-03}
Ly75	1.37	4.90	21.95	1.49×10^{-03}	5.10×10^{-03}
Glipr2	1.11	5.58	21.85	1.51×10^{-03}	5.16×10^{-03}
Gm10371	0.85	3.89	20.58	1.61×10^{-03}	5.47×10^{-03}
Zbtb10	0.88	4.84	21.09	1.67×10^{-03}	5.65×10^{-03}
Nectin2	1.02	5.72	20.94	1.72×10^{-03}	5.78×10^{-03}
Diaph2	0.69	5.68	19.86	1.84×10^{-03}	6.13×10^{-03}
Ntn1	1.58	2.08	20.12	1.89×10^{-03}	6.25×10^{-03}
Gls	0.71	7.01	19.78	1.93×10^{-03}	6.36×10^{-03}
Saa3	8.05	6.21	20.15	1.93×10^{-03}	6.36×10^{-03}
Gm26669	0.88	6.05	20.13	1.93×10^{-03}	6.37×10^{-03}
Serpinb8	0.79	4.53	19.85	1.96×10^{-03}	6.45×10^{-03}
Zfp36l1	0.88	6.20	19.87	2.01×10^{-03}	6.59×10^{-03}
Mxd1	1.94	4.83	19.77	2.04×10^{-03}	6.69×10^{-03}
Cd109	0.59	1.26	10.24	2.08×10^{-03}	6.79×10^{-03}
Nos2	10.19	5.48	19.42	2.15×10^{-03}	7.01×10^{-03}
Tnfrsf1b	1.54	4.56	18.62	2.44×10^{-03}	7.81×10^{-03}
Klf6	1.05	7.49	18.44	2.51×10^{-03}	8.00×10^{-03}
Mir155hg	2.21	2.55	18.34	2.55×10^{-03}	8.11×10^{-03}
Il6	4.67	−0.11	18.18	2.62×10^{-03}	8.29×10^{-03}
Tmem30a	0.67	7.75	17.73	2.63×10^{-03}	8.32×10^{-03}
Gng12	0.66	8.11	17.33	2.81×10^{-03}	8.78×10^{-03}
Serpina3f	6.14	0.38	17.74	2.81×10^{-03}	8.78×10^{-03}
Csrnp1	0.90	4.55	17.50	2.93×10^{-03}	9.09×10^{-03}
Skil	0.66	6.51	17.10	2.97×10^{-03}	9.20×10^{-03}
Raph1	1.09	6.21	17.08	3.14×10^{-03}	9.68×10^{-03}

Differentially expressed genes in DENV−infected/APAP−treated cells compared to Untreated. DENV−infected. and APAP−treated cells

Gene	logFC	logCPM	F	PValue	FDR
mt.Tl1	−0.90	4.71	59.38	2.73×10^{-10}	3.70×10^{-06}

Apob	−0.75	9.96	45.61	3.65×10^{-09}	1.24×10^{-05}
Sacs	−0.72	4.37	41.34	1.39×10^{-08}	1.76×10^{-05}
Hist1h1d	−1.05	2.64	33.66	1.80×10^{-07}	1.10×10^{-04}
Cep290	−0.63	5.16	31.68	7.00×10^{-07}	2.56×10^{-04}
Prex2	−0.69	3.97	29.64	7.57×10^{-07}	2.70×10^{-04}
Sfpq	−0.66	7.24	33.41	8.50×10^{-07}	2.74×10^{-04}
Cenpe	−1.09	2.47	29.59	1.39×10^{-06}	3.76×10^{-04}
Gm38372	−0.92	2.74	26.91	2.27×10^{-06}	5.11×10^{-04}
Zfp182	−0.66	4.59	28.77	4.58×10^{-06}	7.57×10^{-04}
Gm32461	−1.03	2.01	23.90	6.33×10^{-06}	9.24×10^{-04}
Zmat1	−0.65	4.26	26.25	6.66×10^{-06}	9.49×10^{-04}
Akap17b	−0.60	4.14	23.76	6.66×10^{-06}	9.49×10^{-04}
Ccdc68	−0.60	3.72	21.50	1.62×10^{-05}	1.67×10^{-03}
Zfp971	−0.65	3.27	21.08	1.91×10^{-05}	1.80×10^{-03}
Aspm	−1.59	0.99	25.13	1.99×10^{-05}	1.82×10^{-03}
Zfp101	−0.64	3.54	21.14	2.00×10^{-05}	1.82×10^{-03}
Ift74	−0.60	4.39	22.83	2.69×10^{-05}	2.15×10^{-03}
X1700061H18Rik	−0.60	3.45	19.87	3.12×10^{-05}	2.27×10^{-03}
n.R5s29	−0.76	2.95	19.17	4.15×10^{-05}	2.75×10^{-03}
Gm26540	−0.63	3.24	18.55	5.34×10^{-05}	3.09×10^{-03}
Smc2	−0.84	2.91	20.63	6.52×10^{-05}	3.46×10^{-03}
Gm41031	−0.96	1.96	18.07	6.52×10^{-05}	3.46×10^{-03}
X2810403D21Rik	−0.67	2.99	17.85	7.15×10^{-05}	3.66×10^{-03}
Kif15	−0.87	2.08	17.72	7.55×10^{-05}	3.78×10^{-03}
Ckap2	−0.95	1.93	17.28	1.02×10^{-04}	4.56×10^{-03}
Upp2	−0.72	2.65	16.93	1.05×10^{-04}	4.57×10^{-03}
Pgm2l1	−0.59	5.85	21.23	1.10×10^{-04}	4.69×10^{-03}
Chic1	−0.61	3.46	17.29	1.22×10^{-04}	5.05×10^{-03}
Gm15834	−0.67	2.91	16.34	1.35×10^{-04}	5.32×10^{-03}
Ccna2	−0.61	3.45	16.98	1.45×10^{-04}	5.52×10^{-03}
mt.Tm	−0.79	2.38	15.94	1.61×10^{-04}	5.88×10^{-03}
Dcdc2a	−0.60	5.97	20.05	2.25×10^{-04}	7.21×10^{-03}
Brca2	−0.59	3.13	15.14	2.27×10^{-04}	7.24×10^{-03}
Gm26348	−0.75	2.27	15.11	2.30×10^{-04}	7.29×10^{-03}
Atp5l	−0.64	2.91	14.97	2.44×10^{-04}	7.47×10^{-03}
Gm35330	−0.79	2.51	15.60	2.51×10^{-04}	7.54×10^{-03}
Sult6b1	−0.92	1.58	14.69	2.76×10^{-04}	7.96×10^{-03}
Zfp229	−0.60	3.32	15.21	3.03×10^{-04}	8.47×10^{-03}
Mis18bp1	−0.95	1.45	14.45	3.06×10^{-04}	8.50×10^{-03}
Gm47126	−1.06	1.75	14.21	3.40×10^{-04}	9.08×10^{-03}
Mbd4	−0.61	2.99	14.13	3.52×10^{-04}	9.19×10^{-03}
BC006965	−0.73	2.84	15.37	3.64×10^{-04}	9.30×10^{-03}
Zfp975	−0.61	2.99	14.06	3.64×10^{-04}	9.30×10^{-03}
Cep55	−1.23	1.75	17.77	3.76×10^{-04}	9.49×10^{-03}
Ccnb2	−0.95	1.41	13.98	3.77×10^{-04}	9.51×10^{-03}
Vgll3	−0.63	2.70	13.89	3.92×10^{-04}	9.67×10^{-03}
Spc25	−0.63	2.98	14.16	3.95×10^{-04}	9.71×10^{-03}
Cip2a	−0.83	2.65	15.88	4.00×10^{-04}	9.80×10^{-03}
Psmb10	0.72	5.89	46.29	2.97×10^{-09}	1.24×10^{-05}
Rabac1	0.80	5.84	51.77	3.59×10^{-09}	1.24×10^{-05}
Mrpl4	0.73	4.65	44.47	5.19×10^{-09}	1.40×10^{-05}
Pnpla2	0.69	6.25	43.45	7.14×10^{-09}	1.48×10^{-05}
Mir7067	0.73	6.16	45.56	7.65×10^{-09}	1.48×10^{-05}
Rhog	0.73	4.78	42.16	1.12×10^{-08}	1.76×10^{-05}
Snora3	0.75	5.98	45.90	1.22×10^{-08}	1.76×10^{-05}
Mt1	0.71	10.05	41.27	1.43×10^{-08}	1.76×10^{-05}

Fau	0.78	7.91	46.70	1.91×10^{-08}	2.15×10^{-05}
Snord37	0.67	4.92	38.88	3.10×10^{-08}	3.22×10^{-05}
Tspo	0.63	7.08	38.48	3.53×10^{-08}	3.41×10^{-05}
X2200002D01Rik	0.85	4.33	45.79	3.84×10^{-08}	3.47×10^{-05}
X2310039H08Rik	0.69	4.33	36.83	6.11×10^{-08}	5.17×10^{-05}
Ddr1	0.62	6.91	36.22	7.48×10^{-08}	5.95×10^{-05}
Bbc3	0.98	3.06	37.09	7.95×10^{-08}	5.98×10^{-05}
Rpl36	0.64	6.19	35.07	1.39×10^{-07}	9.42×10^{-05}
H2afj	0.65	5.45	35.27	1.39×10^{-07}	9.42×10^{-05}
Fam57b	0.60	7.74	34.07	1.56×10^{-07}	1.00×10^{-04}
H2.Q4	0.66	6.23	33.19	2.18×10^{-07}	1.21×10^{-04}
Foxp4	0.63	4.89	32.93	2.31×10^{-07}	1.21×10^{-04}
Atox1	0.62	5.94	33.59	2.32×10^{-07}	1.21×10^{-04}
Sf3b5	0.65	4.32	31.45	3.89×10^{-07}	1.88×10^{-04}
Uqcrq	0.59	6.83	31.16	4.30×10^{-07}	1.94×10^{-04}
Gm44729	0.63	6.08	33.03	4.73×10^{-07}	2.00×10^{-04}
Cox8a	0.60	8.38	30.33	6.51×10^{-07}	2.56×10^{-04}
Gng5	0.78	4.18	34.71	8.38×10^{-07}	2.74×10^{-04}
Eif4ebp1	0.60	6.13	30.64	8.98×10^{-07}	2.82×10^{-04}
Gm26330	0.67	5.46	33.68	1.01×10^{-06}	3.10×10^{-04}
Ppp1r18os	0.98	2.59	28.39	1.17×10^{-06}	3.44×10^{-04}
Gm44364	0.60	4.41	27.94	1.38×10^{-06}	3.76×10^{-04}
Ubald1	0.63	3.94	27.39	1.69×10^{-06}	4.39×10^{-04}
Cuta	0.61	4.28	26.82	2.09×10^{-06}	4.96×10^{-04}
Gm13257	1.02	2.44	27.00	2.19×10^{-06}	5.11×10^{-04}
Mrpl38	0.61	6.22	28.94	3.09×10^{-06}	6.43×10^{-04}
Zyx	0.62	6.55	29.38	3.53×10^{-06}	6.73×10^{-04}
Gm9843	0.82	2.81	25.37	3.60×10^{-06}	6.76×10^{-04}
Ppp1r35	0.80	2.89	25.13	3.95×10^{-06}	7.12×10^{-04}
Gm44995	1.19	1.83	25.97	4.01×10^{-06}	7.12×10^{-04}
Gm44771	1.15	1.80	25.05	4.07×10^{-06}	7.12×10^{-04}
Rpl13	0.60	7.26	27.63	5.19×10^{-06}	8.16×10^{-04}
Rpl26	0.81	3.12	25.70	5.25×10^{-06}	8.17×10^{-04}
X1700018L02Rik	0.84	2.82	24.34	5.33×10^{-06}	8.20×10^{-04}
Dpm3	0.66	3.59	24.10	5.86×10^{-06}	8.71×10^{-04}
Trp53	0.62	5.46	27.85	6.35×10^{-06}	9.24×10^{-04}
Ndufs7	0.74	5.57	32.72	8.04×10^{-06}	1.08×10^{-03}
Ptp4a3	0.90	2.59	23.17	9.10×10^{-06}	1.15×10^{-03}
B430305J03Rik	0.86	2.64	22.78	9.76×10^{-06}	1.20×10^{-03}
Snord34	0.60	5.66	25.68	1.37×10^{-05}	1.50×10^{-03}
Gm20476	0.65	7.03	27.45	1.58×10^{-05}	1.66×10^{-03}
Ddit3	0.62	5.17	25.50	1.65×10^{-05}	1.69×10^{-03}
Romo1	0.67	5.20	27.43	1.70×10^{-05}	1.72×10^{-03}
Gm14453	0.96	1.98	21.32	1.74×10^{-05}	1.72×10^{-03}
Gm28536	1.06	2.05	21.82	2.20×10^{-05}	1.94×10^{-03}
Polm	0.65	3.47	20.58	2.63×10^{-05}	2.12×10^{-03}
Gm11131	0.72	4.40	22.43	2.82×10^{-05}	2.21×10^{-03}
Gm44672	1.08	1.51	19.89	3.08×10^{-05}	2.26×10^{-03}
Ggt6	0.72	3.01	19.88	3.23×10^{-05}	2.32×10^{-03}
Zfp524	0.63	3.20	19.51	3.61×10^{-05}	2.52×10^{-03}
Zswim4	0.80	3.66	24.58	4.08×10^{-05}	2.73×10^{-03}
Gm45091	1.13	1.19	19.09	4.28×10^{-05}	2.80×10^{-03}
Pold4	0.61	3.48	18.98	4.47×10^{-05}	2.87×10^{-03}
Gm5617	0.65	3.75	20.83	4.62×10^{-05}	2.91×10^{-03}
Crtc2	0.62	4.78	23.16	4.77×10^{-05}	2.97×10^{-03}
Gm43863	0.60	3.74	19.16	5.24×10^{-05}	3.09×10^{-03}

Gm44794	1.09	1.17	18.21	6.15×10^{-05}	3.33×10^{-03}
Gm45828	1.21	1.02	18.40	6.37×10^{-05}	3.42×10^{-03}
Rbm38	0.75	2.57	17.86	7.13×10^{-05}	3.66×10^{-03}
Gm45628	1.24	1.19	19.16	7.40×10^{-05}	3.75×10^{-03}
Epb41l4aos	0.64	3.36	18.41	7.70×10^{-05}	3.83×10^{-03}
Ppdpf	0.60	3.61	18.12	8.09×10^{-05}	3.97×10^{-03}
Tagln2	0.60	7.59	21.98	8.36×10^{-05}	4.04×10^{-03}
Gm45412	0.84	2.20	17.46	8.41×10^{-05}	4.05×10^{-03}
Snord49b	0.67	4.11	21.24	9.72×10^{-05}	4.43×10^{-03}
Gm27184	0.99	1.38	17.07	9.92×10^{-05}	4.49×10^{-03}
Fbxl15	0.61	3.09	16.98	1.03×10^{-04}	4.56×10^{-03}
Gm26643	1.04	1.25	16.56	1.23×10^{-04}	5.06×10^{-03}
Gm23639	0.97	1.56	16.48	1.28×10^{-04}	5.15×10^{-03}
X9330160F10Rik	0.90	1.72	16.40	1.32×10^{-04}	5.25×10^{-03}
Chchd10	0.72	3.77	20.39	1.45×10^{-04}	5.52×10^{-03}
Borcs6	0.63	2.98	16.15	1.47×10^{-04}	5.53×10^{-03}
Lpar2	0.73	3.10	18.03	1.48×10^{-04}	5.53×10^{-03}
Gm23205	0.96	1.42	15.72	1.76×10^{-04}	6.29×10^{-03}
Gm43917	0.65	5.95	21.84	1.81×10^{-04}	6.39×10^{-03}
Snord49a	0.62	3.29	16.17	1.95×10^{-04}	6.71×10^{-03}
Gm44981	1.02	1.42	15.78	1.96×10^{-04}	6.71×10^{-03}
Gm23130	0.74	5.53	23.87	2.03×10^{-04}	6.86×10^{-03}
Gm5292	1.08	1.09	15.35	2.07×10^{-04}	6.93×10^{-03}
Rnd2	0.61	3.04	15.35	2.07×10^{-04}	6.93×10^{-03}
Gm44878	0.77	2.26	15.07	2.34×10^{-04}	7.33×10^{-03}
Gm12764	0.73	2.48	15.03	2.38×10^{-04}	7.42×10^{-03}
Gm7008	0.92	1.39	15.01	2.40×10^{-04}	7.43×10^{-03}
AI413582	0.91	3.02	19.80	2.46×10^{-04}	7.48×10^{-03}
Gm9905	0.86	2.01	15.04	2.47×10^{-04}	7.48×10^{-03}
Tomm6os	0.75	3.83	20.15	2.51×10^{-04}	7.54×10^{-03}
X4930524O07Rik	0.79	2.46	15.39	2.59×10^{-04}	7.70×10^{-03}
B430218F22Rik	1.12	1.78	17.42	2.70×10^{-04}	7.87×10^{-03}
Gm24106	0.88	1.57	14.69	2.76×10^{-04}	7.96×10^{-03}
Gm21981	0.59	4.92	18.73	2.76×10^{-04}	7.96×10^{-03}
Gm16174	1.55	0.25	17.24	2.96×10^{-04}	8.35×10^{-03}
Cd72	0.84	1.73	14.27	3.32×10^{-04}	8.91×10^{-03}
Gm42928	0.62	2.97	14.27	3.32×10^{-04}	8.91×10^{-03}
Alg3	0.67	2.86	14.56	3.83×10^{-04}	9.56×10^{-03}

logFC: logarithmic fold change; logCPM: logarithmic counts−per−million; F: quasi−likelihood F−test; PValue: two−sided p−value; FDR: false discovery rate.

Table S2. Gene Ontology/Biological Process categories significantly enriched for the lists of differentially expressed genes (DEGs) in AML^{-12} hepatocytes after infection by DENV-2 or treatment with APAP, and in gene clusters (2, 4, and 5) in AML^{-12} hepatocytes after infection by DENV-2 and treatment with APAP.

**Untreated cells vs. APAP–treated cells**								
**GO terms significantly enriched in downregulated genes**								
**ID**	**Description**	**GeneRatio**	**BgRatio**	**pvalue**	**p.adjust**	**qvalue**	**geneID**	**Cou**
GO:0042953	lipoprotein transport	3/47	15/21092	4.63×10^{-06}	2.43×10^{-03}	2.00×10^{-03}	ENSMUSG00000040613/ ENSMUSG00000000440/ ENSMUSG00000002992	3
GO:0044872	lipoprotein localization	3/47	15/21092	4.63×10^{-06}	2.43×10^{-03}	2.00×10^{-03}	ENSMUSG00000040613/ ENSMUSG00000000440/ ENSMUSG00000002992	3
GO:0006631	fatty acid metabolic process	7/47	370/21092	1.66×10^{-05}	5.82×10^{-03}	4.78×10^{-03}	ENSMUSG00000074882/ ENSMUSG00000025002/ ENSMUSG00000027533/ ENSMUSG00000000440/ ENSMUSG00000002992/ ENSMUSG00000067231/ ENSMUSG00000060317	7
GO:0019373	epoxygenase P450 pathway	3/47	31/21092	4.46×10^{-05}	1.17×10^{-02}	9.64×10^{-03}	ENSMUSG00000074882/ ENSMUSG00000025002/ ENSMUSG00000067231	3
GO:0033559	unsaturated fatty acid metabolic process	4/47	100/21092	7.25×10^{-05}	1.52×10^{-02}	1.25×10^{-02}	ENSMUSG00000074882/ ENSMUSG00000025002/ ENSMUSG00000027533/ ENSMUSG00000067231	4
GO:0006690	icosanoid metabolic process	4/47	108/21092	9.79×10^{-05}	1.71×10^{-02}	1.41×10^{-02}	ENSMUSG00000074882/ ENSMUSG00000025002/ ENSMUSG00000027533/ ENSMUSG00000067231	4
GO:1901568	fatty acid derivative metabolic process	4/47	131/21092	2.06×10^{-04}	2.72×10^{-02}	2.24×10^{-02}	ENSMUSG00000074882/ ENSMUSG00000025002/ ENSMUSG00000027533/ ENSMUSG00000067231	4
GO:0042738	exogenous drug catabolic process	3/47	53/21092	2.25×10^{-04}	2.72×10^{-02}	2.24×10^{-02}	ENSMUSG00000074882/ ENSMUSG00000025002/ ENSMUSG00000067231	3
GO:0051346	negative regulation of hydrolase activity	6/47	395/21092	2.33×10^{-04}	2.72×10^{-02}	2.24×10^{-02}	ENSMUSG00000021403/ ENSMUSG00000031271/ ENSMUSG00000028415/ ENSMUSG00000034675/ ENSMUSG00000031385/ ENSMUSG00000035403	6
GO:0019369	arachidonic acid metabolic process	3/47	56/21092	2.65×10^{-04}	2.78×10^{-02}	2.29×10^{-02}	ENSMUSG00000074882/ ENSMUSG00000025002/ ENSMUSG00000067231	3
GO:0052547	regulation of peptidase activity	6/47	414/21092	3.00×10^{-04}	2.86×10^{-02}	2.36×10^{-02}	ENSMUSG00000021403/ ENSMUSG00000031271/ ENSMUSG00000028415/ ENSMUSG00000021208/ ENSMUSG00000000440/ ENSMUSG00000035403	6

GO terms significantly enriched in upregulated genes								
**ID**	**Description**	**GeneRatio**	**BgRatio**	**pvalue**	**p.adjust**	**qvalue**	**geneID**	**Count**
GO:0010639	negative regulation of organelle organization	5/11	394/21092	9.34×10^{-07}	0.000	0.0002	ENSMUSG00000052565/ ENSMUSG00000037725/ ENSMUSG00000045328/ ENSMUSG00000027805/ ENSMUSG00000051235	5
GO:2001251	negative regulation of chromosome organization	3/11	145/21092	5.04×10^{-05}	0.009	0.005	ENSMUSG00000052565/ ENSMUSG00000045328/ ENSMUSG00000051235	3
GO:0007094	mitotic spindle assembly checkpoint	2/11	36/21092	0.0002	0.009	0.005	ENSMUSG00000045328/ ENSMUSG00000051235	2
GO:0071173	spindle assembly checkpoint	2/11	36/21092	0.0002	0.009	0.005	ENSMUSG00000045328/ ENSMUSG00000051235	2
GO:0071174	mitotic spindle checkpoint	2/11	38/21092	0.0002	0.009	0.005	ENSMUSG00000045328/ ENSMUSG00000051235	2
GO:0031577	spindle checkpoint	2/11	40/21092	0.0002	0.009	0.005	ENSMUSG00000045328/ ENSMUSG00000051235	2
GO:0045841	negative regulation of mitotic metaphase/anaphase transition	2/11	40/21092	0.0002	0.009	0.005	ENSMUSG00000045328/ ENSMUSG00000051235	2
GO:2000816	negative regulation of mitotic sister chromatid separation	2/11	41/21092	0.0002	0.009	0.005	ENSMUSG00000045328/ ENSMUSG00000051235	2
GO:1902100	negative regulation of metaphase/anaphase transition of cell cycle	2/11	42/21092	0.0002	0.009	0.005	ENSMUSG00000045328/ ENSMUSG00000051235	2
GO:1905819	negative regulation of chromosome separation	2/11	43/21092	0.0002	0.009	0.005	ENSMUSG00000045328/ ENSMUSG00000051235	2
GO:0033048	negative regulation of mitotic sister chromatid segregation	2/11	44/21092	0.0002	0.009	0.005	ENSMUSG00000045328/ ENSMUSG00000051235	2
GO:0033046	negative regulation of sister chromatid segregation	2/11	46/21092	0.0003	0.009	0.005	ENSMUSG00000045328/ ENSMUSG00000051235	2
GO:0051985	negative regulation of chromosome segregation	2/11	49/21092	0.0003	0.010	0.005	ENSMUSG00000045328/ ENSMUSG00000051235	2
GO:0030071	regulation of mitotic metaphase/anaphase transition	2/11	52/21092	0.0003	0.010	0.005	ENSMUSG00000045328/ ENSMUSG00000051235	2

GO:0045839	negative regulation of mitotic nuclear division	2/11	54/21092	0.0003	0.010	0.005	ENSMUSG00000045328/ ENSMUSG00000051235	2
GO:1902099	regulation of metaphase/anaphase transition of cell cycle	2/11	55/21092	0.0004	0.010	0.005	ENSMUSG00000045328/ ENSMUSG00000051235	2
GO:0007091	metaphase/anaphase transition of mitotic cell cycle	2/11	56/21092	0.0004	0.010	0.005	ENSMUSG00000045328/ ENSMUSG00000051235	2
GO:0010965	regulation of mitotic sister chromatid separation	2/11	57/21092	0.0004	0.010	0.005	ENSMUSG00000045328/ ENSMUSG00000051235	2
GO:0044784	metaphase/anaphase transition of cell cycle	2/11	59/21092	0.0004	0.010	0.005	ENSMUSG00000045328/ ENSMUSG00000051235	2
GO:0051306	mitotic sister chromatid separation	2/11	60/21092	0.0004	0.010	0.005	ENSMUSG00000045328/ ENSMUSG00000051235	2
GO:1905818	regulation of chromosome separation	2/11	63/21092	0.0005	0.010	0.005	ENSMUSG00000045328/ ENSMUSG00000051235	2
GO:0051784	negative regulation of nuclear division	2/11	64/21092	0.0005	0.010	0.005	ENSMUSG00000045328/ ENSMUSG00000051235	2
GO:0033047	regulation of mitotic sister chromatid segregation	2/11	69/21092	0.0006	0.011	0.006	ENSMUSG00000045328/ ENSMUSG00000051235	2
GO:0033044	regulation of chromosome organization	3/11	349/21092	0.0007	0.013	0.006	ENSMUSG00000052565/ ENSMUSG00000045328/ ENSMUSG00000051235	3
GO:0033045	regulation of sister chromatid segregation	2/11	83/21092	0.0008	0.015	0.007	ENSMUSG00000045328/ ENSMUSG00000051235	2
GO:0051304	chromosome separation	2/11	92/21092	0.0010	0.017	0.009	ENSMUSG00000045328/ ENSMUSG00000051235	2
GO:0000280	nuclear division	3/11	421/21092	0.0012	0.019	0.010	ENSMUSG00000045328/ ENSMUSG00000051235/ ENSMUSG00000025912	3
GO:0051983	regulation of chromosome segregation	2/11	106/21092	0.0013	0.021	0.011	ENSMUSG00000045328/ ENSMUSG00000051235	2
GO:0048285	organelle fission	3/11	470/21092	0.0016	0.025	0.012	ENSMUSG00000045328/ ENSMUSG00000051235/ ENSMUSG00000025912	3
GO:0007093	mitotic cell cycle checkpoint	2/11	131/21092	0.0020	0.030	0.015	ENSMUSG00000045328/ ENSMUSG00000051235	2
GO:1902904	negative regulation of supramolecular fiber organization	2/11	144/21092	0.0024	0.035	0.018	ENSMUSG00000037725/ ENSMUSG00000027805	2

GO:1901991	negative regulation of mitotic cell cycle phase transition	2/11	146/21092	0.0025	0.035	0.018	ENSMUSG00000045328/ ENSMUSG00000051235	2
GO:0000070	mitotic sister chromatid segregation	2/11	148/21092	0.0026	0.035	0.018	ENSMUSG00000045328/ ENSMUSG00000051235	2
GO:0071478	cellular response to radiation	2/11	152/21092	0.0027	0.035	0.018	ENSMUSG00000051235/ ENSMUSG00000038418	2
GO:0051494	negative regulation of cytoskeleton organization	2/11	153/21092	0.0028	0.035	0.018	ENSMUSG00000037725/ ENSMUSG00000027805	2
GO:1901988	negative regulation of cell cycle phase transition	2/11	164/21092	0.0032	0.039	0.020	ENSMUSG00000045328/ ENSMUSG00000051235	2
GO:0045931	positive regulation of mitotic cell cycle	2/11	165/21092	0.0032	0.039	0.020	ENSMUSG00000045328/ ENSMUSG00000051235	2
GO:0065004	protein−DNA complex assembly	2/11	170/21092	0.0034	0.040	0.020	ENSMUSG00000052565/ ENSMUSG00000045328	2
GO:0000075	cell cycle checkpoint	2/11	176/21092	0.0036	0.042	0.021	ENSMUSG00000045328/ ENSMUSG00000051235	2
GO:0007088	regulation of mitotic nuclear division	2/11	181/21092	0.0038	0.042	0.021	ENSMUSG00000045328/ ENSMUSG00000051235	2
GO:0000819	sister chromatid segregation	2/11	182/21092	0.0039	0.042	0.021	ENSMUSG00000045328/ ENSMUSG00000051235	2
GO:0070507	regulation of microtubule cytoskeleton organization	2/11	200/21092	0.0047	0.048	0.024	ENSMUSG00000037725/ ENSMUSG00000051235	2
GO:0071824	protein−DNA complex subunit organization	2/11	210/21092	0.0051	0.048	0.024	ENSMUSG00000052565/ ENSMUSG00000045328	2
GO:0051783	regulation of nuclear division	2/11	211/21092	0.0052	0.048	0.024	ENSMUSG00000045328/ ENSMUSG00000051235	2
GO:0072203	cell proliferation involved in metanephros development	1/11	10/21092	0.0052	0.048	0.024	ENSMUSG00000038418	1
GO:1905820	positive regulation of chromosome separation	1/11	10/21092	0.0052	0.048	0.024	ENSMUSG00000045328	1
GO:0072124	regulation of glomerular mesangial cell proliferation	1/11	11/21092	0.0057	0.048	0.024	ENSMUSG00000038418	1
GO:0010948	negative regulation of cell cycle process	2/11	228/21092	0.0060	0.048	0.024	ENSMUSG00000045328/ ENSMUSG00000051235	2
GO:0042033	chemokine biosynthetic process	1/11	12/21092	0.0062	0.048	0.024	ENSMUSG00000038418	1
GO:0045073	regulation of chemokine biosynthetic process	1/11	12/21092	0.0062	0.048	0.024	ENSMUSG00000038418	1
GO:0070914	UV−damage excision repair	1/11	12/21092	0.0062	0.048	0.024	ENSMUSG00000051235	1

GO:0072110	glomerular mesangial cell proliferation	1/11	12/21092	0.0062	0.048	0.024	ENSMUSG00000038418	1
GO:0090193	positive regulation of glomerulus development	1/11	12/21092	0.0062	0.048	0.024	ENSMUSG00000038418	1
GO:0032886	regulation of microtubule−based process	2/11	234/21092	0.0063	0.048	0.024	ENSMUSG00000037725/ ENSMUSG00000051235	2
GO:0045930	negative regulation of mitotic cell cycle	2/11	238/21092	0.0065	0.048	0.024	ENSMUSG00000045328/ ENSMUSG00000051235	2
GO:0061418	regulation of transcription from RNA polymerase II promoter in response to hypoxia	1/11	13/21092	0.0068	0.048	0.024	ENSMUSG00000038418	1
GO:0072216	positive regulation of metanephros development	1/11	13/21092	0.0068	0.048	0.024	ENSMUSG00000038418	1
GO:0090266	regulation of mitotic cell cycle spindle assembly checkpoint	1/11	13/21092	0.0068	0.048	0.024	ENSMUSG00000051235	1
GO:1903504	regulation of mitotic spindle checkpoint	1/11	13/21092	0.0068	0.048	0.024	ENSMUSG00000051235	1
GO:0090068	positive regulation of cell cycle process	2/11	246/21092	0.0070	0.048	0.024	ENSMUSG00000045328/ ENSMUSG00000051235	2
GO:0051315	attachment of mitotic spindle microtubules to kinetochore	1/11	14/21092	0.0073	0.048	0.024	ENSMUSG00000045328	1
GO:0051988	regulation of attachment of spindle microtubules to kinetochore	1/11	14/21092	0.0073	0.048	0.024	ENSMUSG00000045328	1
GO:0072224	metanephric glomerulus development	1/11	14/21092	0.0073	0.048	0.024	ENSMUSG00000038418	1
GO:0098813	nuclear chromosome segregation	2/11	258/21092	0.0076	0.048	0.024	ENSMUSG00000045328/ ENSMUSG00000051235	2
GO:1901990	regulation of mitotic cell cycle phase transition	2/11	260/21092	0.0077	0.048	0.024	ENSMUSG00000045328/ ENSMUSG00000051235	2
GO:0042448	progesterone metabolic process	1/11	15/21092	0.0078	0.048	0.024	ENSMUSG00000038418	1
GO:0051382	kinetochore assembly	1/11	15/21092	0.0078	0.048	0.024	ENSMUSG00000045328	1
GO:0072109	glomerular mesangium development	1/11	15/21092	0.0078	0.048	0.024	ENSMUSG00000038418	1
GO:0090231	regulation of spindle checkpoint	1/11	15/21092	0.0078	0.048	0.024	ENSMUSG00000051235	1
GO:1901722	regulation of cell proliferation involved in kidney development	1/11	15/21092	0.0078	0.048	0.024	ENSMUSG00000038418	1

GO:0007018	microtubule−based movement	2/11	264/21092	0.0080	0.048	0.024	ENSMUSG00000045328/ ENSMUSG00000036768	2
GO:0006310	DNA recombination	2/11	268/21092	0.0082	0.048	0.024	ENSMUSG00000052565/ ENSMUSG00000051235	2
GO:0010566	regulation of ketone biosynthetic process	1/11	16/21092	0.0083	0.048	0.024	ENSMUSG00000038418	1
GO:0016584	nucleosome positioning	1/11	16/21092	0.0083	0.048	0.024	ENSMUSG00000052565	1
GO:0050755	chemokine metabolic process	1/11	16/21092	0.0083	0.048	0.024	ENSMUSG00000038418	1
GO:0090192	regulation of glomerulus development	1/11	16/21092	0.0083	0.048	0.024	ENSMUSG00000038418	1
GO:0098885	modification of postsynaptic actin cytoskeleton	1/11	16/21092	0.0083	0.048	0.024	ENSMUSG00000027805	1
GO:0140014	mitotic nuclear division	2/11	276/21092	0.0087	0.048	0.024	ENSMUSG00000045328/ ENSMUSG00000051235	2
GO:0006700	C21−steroid hormone biosynthetic process	1/11	17/21092	0.0088	0.048	0.024	ENSMUSG00000038418	1
GO:0062033	positive regulation of mitotic sister chromatid segregation	1/11	17/21092	0.0088	0.048	0.024	ENSMUSG00000045328	1
GO:0080182	histone H3−K4 trimethylation	1/11	17/21092	0.0088	0.048	0.024	ENSMUSG00000052565	1
GO:0010631	epithelial cell migration	2/11	282/21092	0.0090	0.048	0.024	ENSMUSG00000027805/ ENSMUSG00000072437	2
GO:0090132	epithelium migration	2/11	284/21092	0.0092	0.048	0.024	ENSMUSG00000027805/ ENSMUSG00000072437	2
GO:0090130	tissue migration	2/11	286/21092	0.0093	0.048	0.024	ENSMUSG00000027805/ ENSMUSG00000072437	2
GO:0034587	piRNA metabolic process	1/11	18/21092	0.0093	0.048	0.024	ENSMUSG00000025912	1
GO:0071214	cellular response to abiotic stimulus	2/11	290/21092	0.0095	0.048	0.024	ENSMUSG00000051235/ ENSMUSG00000038418	2
GO:0104004	cellular response to environmental stimulus	2/11	290/21092	0.0095	0.048	0.024	ENSMUSG00000051235/ ENSMUSG00000038418	2
GO:1901987	regulation of cell cycle phase transition	2/11	291/21092	0.0096	0.048	0.024	ENSMUSG00000045328/ ENSMUSG00000051235	2
GO:0010529	negative regulation of transposition	1/11	19/21092	0.0099	0.048	0.024	ENSMUSG00000025912	1
GO:0031936	negative regulation of chromatin silencing	1/11	19/21092	0.0099	0.048	0.024	ENSMUSG00000052565	1
GO:0046886	positive regulation of hormone biosynthetic process	1/11	19/21092	0.0099	0.048	0.024	ENSMUSG00000038418	1
GO:1900151	regulation of nuclear−transcribed mRNA catabolic process. deadenylation−dependent decay	1/11	19/21092	0.0099	0.048	0.024	ENSMUSG00000072437	1

GO:1900153	positive regulation of nuclear−transcribed mRNA catabolic process. deadenylation−dependent decay	1/11	19/21092	0.0099	0.048	0.024	ENSMUSG00000072437	1
GO:0010528	regulation of transposition	1/11	20/21092	0.0104	0.048	0.025	ENSMUSG00000025912	1
GO:0051383	kinetochore organization	1/11	20/21092	0.0104	0.048	0.025	ENSMUSG00000045328	1
GO:0070498	interleukin−1−mediated signaling pathway	1/11	20/21092	0.0104	0.048	0.025	ENSMUSG00000038418	1
**Untreated cells vs. DENV−infected cells**								
**GO terms significantly enriched in downregulated genes**								
**ID**	**Description**	**GeneRatio**	**BgRatio**	**pvalue**	**p.adjust**	**qvalue**	**geneID**	**Cou**
GO:0044282	small molecule catabolic process	33/350	308/21092	1.73×10^{-17}	2.68×10^{-14}	2.35×10^{-14}	ENSMUSG00000024507/ ENSMUSG00000090150/ ENSMUSG00000034456/ ENSMUSG00000010651/ ENSMUSG00000026385/ ENSMUSG00000034371/ ENSMUSG00000003809/ ENSMUSG00000027227/ ENSMUSG00000031173/ ENSMUSG00000116378/ ENSMUSG00000023832/ ENSMUSG00000006378/ ENSMUSG00000034424/ ENSMUSG00000010051/ ENSMUSG00000027332/ ENSMUSG00000036880/ ENSMUSG00000002985/ ENSMUSG00000035936/ ENSMUSG00000062908/ ENSMUSG00000021794/ ENSMUSG00000030826/ ENSMUSG00000021884/ ENSMUSG00000009614/ ENSMUSG00000017453/ ENSMUSG00000001155/ ENSMUSG00000010047/ ENSMUSG00000028307/ ENSMUSG00000011305/ ENSMUSG00000024579/ ENSMUSG00000029597/ ENSMUSG00000028755/ ENSMUSG00000054422/ ENSMUSG0000036892	33
GO:0016054	organic acid catabolic process	28/350	213/21092	2.46×10^{-17}	2.68×10^{-14}	2.35×10^{-14}	ENSMUSG00000024507/ ENSMUSG00000090150/ ENSMUSG00000034456/ ENSMUSG00000010651/ ENSMUSG00000026385/ ENSMUSG00000003809/ ENSMUSG00000031173/ ENSMUSG00000116378/ ENSMUSG00000023832/ ENSMUSG00000006378/ ENSMUSG00000034424/ ENSMUSG00000010051/ ENSMUSG00000027332/ ENSMUSG00000036880/ ENSMUSG00000035936/ ENSMUSG00000062908/ ENSMUSG00000021794/ ENSMUSG00000030826/	28

							ENSMUSG00000021884/ ENSMUSG00000009614/ ENSMUSG00000017453/ ENSMUSG00000001155/ ENSMUSG00000010047/ ENSMUSG00000011305/ ENSMUSG00000024579/ ENSMUSG00000029597/ ENSMUSG00000054422/ ENSMUSG00000036892	
GO:0046395	carboxylic acid catabolic process	28/350	213/21092	2.46×10^{-17}	2.68×10^{-14}	2.35×10^{-14}	ENSMUSG00000024507/ ENSMUSG00000090150/ ENSMUSG00000034456/ ENSMUSG00000010651/ ENSMUSG00000026385/ ENSMUSG00000003809/ ENSMUSG00000031173/ ENSMUSG00000116378/ ENSMUSG00000023832/ ENSMUSG00000006378/ ENSMUSG00000034424/ ENSMUSG00000010051/ ENSMUSG00000027332/ ENSMUSG00000036880/ ENSMUSG00000035936/ ENSMUSG00000062908/ ENSMUSG00000021794/ ENSMUSG00000030826/ ENSMUSG00000021884/ ENSMUSG00000009614/ ENSMUSG00000017453/ ENSMUSG00000001155/ ENSMUSG00000010047/ ENSMUSG00000011305/ ENSMUSG00000024579/ ENSMUSG00000029597/ ENSMUSG00000054422/ ENSMUSG00000036892	28
GO:0006631	fatty acid metabolic process	30/350	370/21092	8.95×10^{-13}	7.30×10^{-10}	6.41×10^{-10}	ENSMUSG00000024507/ ENSMUSG00000021620/ ENSMUSG00000090150/ ENSMUSG00000010651/ ENSMUSG00000025002/ ENSMUSG00000026385/ ENSMUSG00000003809/ ENSMUSG00000041220/ ENSMUSG00000025203/ ENSMUSG00000018796/ ENSMUSG00000023832/ ENSMUSG00000027332/ ENSMUSG00000036880/ ENSMUSG00000035936/ ENSMUSG00000062908/ ENSMUSG00000032080/ ENSMUSG00000021884/ ENSMUSG00000026853/ ENSMUSG00000002944/ ENSMUSG00000094806/ ENSMUSG00000002992/ ENSMUSG00000011305/ ENSMUSG00000022445/ ENSMUSG00000062624/ ENSMUSG00000054422/ ENSMUSG00000066072/ ENSMUSG00000023070/ ENSMUSG00000067231/ ENSMUSG00000017950/ ENSMUSG00000017146	30
GO:0072521	purine−containing compound metabolic process	32/350	469/21092	1.52×10^{-11}	9.95×10^{-09}	8.73×10^{-09}	ENSMUSG00000024507/ ENSMUSG00000002769/ ENSMUSG00000021620/ ENSMUSG00000013593/ ENSMUSG00000034371/ ENSMUSG00000003809/ ENSMUSG00000021236/ ENSMUSG00000024248/ ENSMUSG00000050856/	32

							ENSMUSG00000018796/ ENSMUSG00000027875/ ENSMUSG00000074218/ ENSMUSG00000006057/ ENSMUSG00000036880/ ENSMUSG00000020150/ ENSMUSG00000054428/ ENSMUSG00000060600/ ENSMUSG00000017453/ ENSMUSG00000046329/ ENSMUSG00000073435/ ENSMUSG00000033610/ ENSMUSG00000019989/ ENSMUSG00000026576/ ENSMUSG00000032081/ ENSMUSG00000028307/ ENSMUSG00000064370/ ENSMUSG00000049422/ ENSMUSG00000064363/ ENSMUSG00000021699/ ENSMUSG00000059434/ ENSMUSG00000017950/ ENSMUSG00000064367	
GO:1901605	alpha−amino acid metabolic process	21/350	200/21092	2.14×10^{-11}	1.06×10^{-08}	9.27×10^{-09}	ENSMUSG00000002769/ ENSMUSG00000034456/ ENSMUSG00000028179/ ENSMUSG00000031173/ ENSMUSG00000039648/ ENSMUSG00000116378/ ENSMUSG00000006378/ ENSMUSG00000034424/ ENSMUSG00000027332/ ENSMUSG00000035936/ ENSMUSG00000020150/ ENSMUSG00000024640/ ENSMUSG00000021794/ ENSMUSG00000030826/ ENSMUSG00000009614/ ENSMUSG00000017453/ ENSMUSG00000001155/ ENSMUSG00000029597/ ENSMUSG00000076441/ ENSMUSG00000036892/ ENSMUSG00000017950	21
GO:0006520	cellular amino acid metabolic process	24/350	268/21092	2.32×10^{-11}	1.06×10^{-08}	9.27×10^{-09}	ENSMUSG00000002769/ ENSMUSG00000034456/ ENSMUSG00000028179/ ENSMUSG00000031173/ ENSMUSG00000039648/ ENSMUSG00000023262/ ENSMUSG00000116378/ ENSMUSG00000006378/ ENSMUSG00000034424/ ENSMUSG00000027332/ ENSMUSG00000035936/ ENSMUSG00000020150/ ENSMUSG00000024640/ ENSMUSG00000021794/ ENSMUSG00000030826/ ENSMUSG00000009614/ ENSMUSG00000017453/ ENSMUSG00000001155/ ENSMUSG00000024579/ ENSMUSG00000029597/ ENSMUSG00000076441/ ENSMUSG00000036892/ ENSMUSG00000020182/ ENSMUSG00000017950	24
GO:0009063	cellular amino acid catabolic process	15/350	91/21092	2.59×10^{-11}	1.06×10^{-08}	9.27×10^{-09}	ENSMUSG00000034456/ ENSMUSG00000031173/ ENSMUSG00000116378/ ENSMUSG00000006378/ ENSMUSG00000034424/ ENSMUSG00000027332/ ENSMUSG00000035936/ ENSMUSG00000021794/ ENSMUSG00000030826/	15

							ENSMUSG00000009614/ ENSMUSG00000017453/ ENSMUSG00000001155/ ENSMUSG00000024579/ ENSMUSG00000029597/ ENSMUSG00000036892	
GO:0019693	ribose phosphate metabolic process	30/350	427/21092	3.31×10^{-11}	1.10×10^{-08}	9.64×10^{-09}	ENSMUSG00000024507/ ENSMUSG00000021620/ ENSMUSG00000013593/ ENSMUSG00000034371/ ENSMUSG00000003809/ ENSMUSG00000021236/ ENSMUSG00000024248/ ENSMUSG00000050856/ ENSMUSG00000018796/ ENSMUSG00000027875/ ENSMUSG00000074218/ ENSMUSG00000006057/ ENSMUSG00000036880/ ENSMUSG00000054428/ ENSMUSG00000060600/ ENSMUSG00000005951/ ENSMUSG00000017453/ ENSMUSG00000046329/ ENSMUSG00000073435/ ENSMUSG00000033610/ ENSMUSG00000019989/ ENSMUSG00000026576/ ENSMUSG00000032081/ ENSMUSG00000028307/ ENSMUSG00000064370/ ENSMUSG00000049422/ ENSMUSG00000064363/ ENSMUSG00000021699/ ENSMUSG00000017950/ ENSMUSG00000064367	30
GO:0009150	purine ribonucleotide metabolic process	29/350	400/21092	3.37×10^{-11}	1.10×10^{-08}	9.64×10^{-09}	ENSMUSG00000024507/ ENSMUSG00000021620/ ENSMUSG00000013593/ ENSMUSG00000034371/ ENSMUSG00000003809/ ENSMUSG00000021236/ ENSMUSG00000024248/ ENSMUSG00000050856/ ENSMUSG00000018796/ ENSMUSG00000027875/ ENSMUSG00000074218/ ENSMUSG00000006057/ ENSMUSG00000036880/ ENSMUSG00000054428/ ENSMUSG00000060600/ ENSMUSG00000017453/ ENSMUSG00000046329/ ENSMUSG00000073435/ ENSMUSG00000033610/ ENSMUSG00000019989/ ENSMUSG00000026576/ ENSMUSG00000032081/ ENSMUSG00000028307/ ENSMUSG00000064370/ ENSMUSG00000049422/ ENSMUSG00000064363/ ENSMUSG00000021699/ ENSMUSG00000017950/ ENSMUSG00000064367	29
GO:0044242	cellular lipid catabolic process	20/350	189/21092	5.62×10^{-11}	1.65×10^{-08}	1.45×10^{-08}	ENSMUSG00000024507/ ENSMUSG00000090150/ ENSMUSG00000010651/ ENSMUSG00000026385/ ENSMUSG00000003809/ ENSMUSG00000020609/ ENSMUSG00000023832/ ENSMUSG00000027332/ ENSMUSG00000036880/ ENSMUSG00000002985/ ENSMUSG00000027761/ ENSMUSG00000062908/ ENSMUSG00000032080/ ENSMUSG00000021884/ ENSMUSG00000032081/	20

							ENSMUSG00000002992/ ENSMUSG00000011305/ ENSMUSG00000054422/ ENSMUSG00000032083/ ENSMUSG00000051177	
GO:0009259	ribonucleotide metabolic process	29/350	410/21092	6.08×10^{-11}	1.65×10^{-08}	1.45×10^{-08}	ENSMUSG00000024507/ ENSMUSG00000021620/ ENSMUSG00000013593/ ENSMUSG00000034371/ ENSMUSG00000003809/ ENSMUSG00000021236/ ENSMUSG00000024248/ ENSMUSG00000050856/ ENSMUSG00000018796/ ENSMUSG00000027875/ ENSMUSG00000074218/ ENSMUSG00000006057/ ENSMUSG00000036880/ ENSMUSG00000054428/ ENSMUSG00000060600/ ENSMUSG00000017453/ ENSMUSG00000046329/ ENSMUSG00000073435/ ENSMUSG00000033610/ ENSMUSG00000019989/ ENSMUSG00000026576/ ENSMUSG00000032081/ ENSMUSG00000028307/ ENSMUSG00000064370/ ENSMUSG00000049422/ ENSMUSG00000064363/ ENSMUSG00000021699/ ENSMUSG00000017950/ ENSMUSG00000064367	29
GO:0006163	purine nucleotide metabolic process	29/350	426/21092	1.51×10^{-10}	3.79×10^{-08}	3.33×10^{-08}	ENSMUSG00000024507/ ENSMUSG00000021620/ ENSMUSG00000013593/ ENSMUSG00000034371/ ENSMUSG00000003809/ ENSMUSG00000021236/ ENSMUSG00000024248/ ENSMUSG00000050856/ ENSMUSG00000018796/ ENSMUSG00000027875/ ENSMUSG00000074218/ ENSMUSG00000006057/ ENSMUSG00000036880/ ENSMUSG00000054428/ ENSMUSG00000060600/ ENSMUSG00000017453/ ENSMUSG00000046329/ ENSMUSG00000073435/ ENSMUSG00000033610/ ENSMUSG00000019989/ ENSMUSG00000026576/ ENSMUSG00000032081/ ENSMUSG00000028307/ ENSMUSG00000064370/ ENSMUSG00000049422/ ENSMUSG00000064363/ ENSMUSG00000021699/ ENSMUSG00000017950/ ENSMUSG00000064367	29
GO:0046889	positive regulation of lipid biosynthetic process	14/350	90/21092	2.68×10^{-10}	6.26×10^{-08}	5.49×10^{-08}	ENSMUSG00000026385/ ENSMUSG00000002985/ ENSMUSG00000024892/ ENSMUSG00000032080/ ENSMUSG00000003585/ ENSMUSG00000022150/ ENSMUSG00000003555/ ENSMUSG00000032081/ ENSMUSG00000002992/ ENSMUSG00000011305/ ENSMUSG00000036585/ ENSMUSG00000032083/ ENSMUSG00000023070/ ENSMUSG00000017950	14

GO:1901606	alpha−amino acid catabolic process	13/350	77/21092	4.09×10^{-10}	8.91×10^{-08}	7.81×10^{-08}	ENSMUSG00000034456/ ENSMUSG00000031173/ ENSMUSG00000116378/ ENSMUSG00000006378/ ENSMUSG00000034424/ ENSMUSG00000027332/ ENSMUSG00000021794/ ENSMUSG00000030826/ ENSMUSG00000009614/ ENSMUSG00000017453/ ENSMUSG00000001155/ ENSMUSG00000029597/ ENSMUSG00000036892	13
GO:0045834	positive regulation of lipid metabolic process	17/350	151/21092	6.04×10^{-10}	1.23×10^{-07}	1.08×10^{-07}	ENSMUSG00000026385/ ENSMUSG00000002985/ ENSMUSG00000027761/ ENSMUSG00000024892/ ENSMUSG00000032080/ ENSMUSG00000003585/ ENSMUSG00000002944/ ENSMUSG00000022150/ ENSMUSG00000003555/ ENSMUSG00000032081/ ENSMUSG00000002992/ ENSMUSG00000011305/ ENSMUSG00000036585/ ENSMUSG00000054422/ ENSMUSG00000032083/ ENSMUSG00000023070/ ENSMUSG00000017950	17
GO:0006641	triglyceride metabolic process	14/350	97/21092	7.48×10^{-10}	1.44×10^{-07}	1.26×10^{-07}	ENSMUSG00000026385/ ENSMUSG00000020609/ ENSMUSG00000018796/ ENSMUSG00000002985/ ENSMUSG00000027761/ ENSMUSG00000032080/ ENSMUSG00000028158/ ENSMUSG00000002944/ ENSMUSG00000040564/ ENSMUSG00000032081/ ENSMUSG00000002992/ ENSMUSG00000011305/ ENSMUSG00000032083/ ENSMUSG00000023070	14
GO:0051186	cofactor metabolic process	29/350	478/21092	2.18×10^{-09}	3.95×10^{-07}	3.46×10^{-07}	ENSMUSG00000024507/ ENSMUSG00000002769/ ENSMUSG00000021620/ ENSMUSG00000034371/ ENSMUSG00000003809/ ENSMUSG00000028179/ ENSMUSG00000021236/ ENSMUSG00000025453/ ENSMUSG00000116207/ ENSMUSG00000018796/ ENSMUSG00000042770/ ENSMUSG00000027875/ ENSMUSG00000026489/ ENSMUSG00000036880/ ENSMUSG00000035936/ ENSMUSG00000020150/ ENSMUSG00000054428/ ENSMUSG00000039450/ ENSMUSG00000060600/ ENSMUSG00000032080/ ENSMUSG00000005951/ ENSMUSG00000026198/ ENSMUSG00000009614/ ENSMUSG00000017453/ ENSMUSG00000033610/ ENSMUSG00000028307/ ENSMUSG00000028743/ ENSMUSG00000023070/ ENSMUSG00000017950	29

GO:0019433	triglyceride catabolic process	8/350	24/21092	3.10×10^{-09}	5.32×10^{-07}	4.67×10^{-07}	ENSMUSG00000020609/ ENSMUSG00000002985/ ENSMUSG00000027761/ ENSMUSG00000032080/ ENSMUSG00000032081/ ENSMUSG00000002992/ ENSMUSG00000011305/ ENSMUSG00000032083	8
GO:0019395	fatty acid oxidation	13/350	96/21092	6.77×10^{-09}	1.11×10^{-06}	9.70×10^{-07}	ENSMUSG00000024507/ ENSMUSG00000090150/ ENSMUSG00000010651/ ENSMUSG00000026385/ ENSMUSG00000003809/ ENSMUSG00000023832/ ENSMUSG00000027332/ ENSMUSG00000036880/ ENSMUSG00000062908/ ENSMUSG00000021884/ ENSMUSG00000002944/ ENSMUSG00000011305/ ENSMUSG00000054422	13
GO:0034440	lipid oxidation	13/350	98/21092	8.75×10^{-09}	1.36×10^{-06}	1.19×10^{-06}	ENSMUSG00000024507/ ENSMUSG00000090150/ ENSMUSG00000010651/ ENSMUSG00000026385/ ENSMUSG00000003809/ ENSMUSG00000023832/ ENSMUSG00000027332/ ENSMUSG00000036880/ ENSMUSG00000062908/ ENSMUSG00000021884/ ENSMUSG00000002944/ ENSMUSG00000011305/ ENSMUSG00000054422	13
GO:0006635	fatty acid beta−oxidation	11/350	67/21092	1.26×10^{-08}	1.80×10^{-06}	1.58×10^{-06}	ENSMUSG00000024507/ ENSMUSG00000090150/ ENSMUSG00000010651/ ENSMUSG00000026385/ ENSMUSG00000003809/ ENSMUSG00000023832/ ENSMUSG00000027332/ ENSMUSG00000036880/ ENSMUSG00000062908/ ENSMUSG00000011305/ ENSMUSG00000054422	11
GO:0006732	coenzyme metabolic process	22/350	311/21092	1.33×10^{-08}	1.80×10^{-06}	1.58×10^{-06}	ENSMUSG00000024507/ ENSMUSG00000002769/ ENSMUSG00000021620/ ENSMUSG00000034371/ ENSMUSG00000003809/ ENSMUSG00000021236/ ENSMUSG00000025453/ ENSMUSG00000116207/ ENSMUSG00000018796/ ENSMUSG00000027875/ ENSMUSG00000026489/ ENSMUSG00000036880/ ENSMUSG00000020150/ ENSMUSG00000039450/ ENSMUSG00000060600/ ENSMUSG00000005951/ ENSMUSG00000009614/ ENSMUSG00000017453/ ENSMUSG00000033610/ ENSMUSG00000028307/ ENSMUSG00000023070/ ENSMUSG00000017950	22
GO:0006639	acylglycerol metabolic process	14/350	121/21092	1.41×10^{-08}	1.80×10^{-06}	1.58×10^{-06}	ENSMUSG00000026385/ ENSMUSG00000020609/ ENSMUSG00000018796/ ENSMUSG00000002985/ ENSMUSG00000027761/ ENSMUSG00000032080/	14

							ENSMUSG00000028158/ ENSMUSG00000002944/ ENSMUSG00000040564/ ENSMUSG00000032081/ ENSMUSG00000002992/ ENSMUSG00000011305/ ENSMUSG00000032083/ ENSMUSG00000023070	
GO:0046890	regulation of lipid biosynthetic process	16/350	163/21092	1.43×10^{-08}	1.80×10^{-06}	1.58×10^{-06}	ENSMUSG00000026385/ ENSMUSG00000020609/ ENSMUSG00000002985/ ENSMUSG00000024892/ ENSMUSG00000032080/ ENSMUSG00000003585/ ENSMUSG00000022150/ ENSMUSG00000003555/ ENSMUSG00000032081/ ENSMUSG00000002992/ ENSMUSG00000011305/ ENSMUSG00000036585/ ENSMUSG00000032083/ ENSMUSG00000023070/ ENSMUSG00000017950/ ENSMUSG00000017146	16
GO:0062012	regulation of small molecule metabolic process	23/350	340/21092	1.43×10^{-08}	1.80×10^{-06}	1.58×10^{-06}	ENSMUSG00000002769/ ENSMUSG00000026385/ ENSMUSG00000020609/ ENSMUSG00000021236/ ENSMUSG00000024248/ ENSMUSG00000074218/ ENSMUSG00000002985/ ENSMUSG00000054428/ ENSMUSG00000062908/ ENSMUSG00000032080/ ENSMUSG00000003585/ ENSMUSG00000046329/ ENSMUSG00000022150/ ENSMUSG00000032081/ ENSMUSG00000002992/ ENSMUSG00000011305/ ENSMUSG00000028755/ ENSMUSG00000036585/ ENSMUSG00000054422/ ENSMUSG00000059434/ ENSMUSG00000023070/ ENSMUSG00000017950/ ENSMUSG00000017146	23
GO:0046394	carboxylic acid biosynthetic process	23/350	341/21092	1.51×10^{-08}	1.83×10^{-06}	1.61×10^{-06}	ENSMUSG00000021620/ ENSMUSG00000034371/ ENSMUSG00000028179/ ENSMUSG00000031173/ ENSMUSG00000046402/ ENSMUSG00000021236/ ENSMUSG00000041220/ ENSMUSG00000025203/ ENSMUSG00000010051/ ENSMUSG00000020150/ ENSMUSG00000024640/ ENSMUSG00000021794/ ENSMUSG00000030826/ ENSMUSG00000060600/ ENSMUSG00000032080/ ENSMUSG00000056148/ ENSMUSG00000028307/ ENSMUSG00000002992/ ENSMUSG00000029597/ ENSMUSG00000076441/ ENSMUSG00000023070/ ENSMUSG00000017950/ ENSMUSG00000017146	23
GO:0016053	organic acid biosynthetic process	23/350	342/21092	1.60×10^{-08}	1.86×10^{-06}	1.63×10^{-06}	ENSMUSG00000021620/ ENSMUSG00000034371/ ENSMUSG00000028179/ ENSMUSG00000031173/ ENSMUSG00000046402/ ENSMUSG00000021236/	23

							ENSMUSG00000041220/ ENSMUSG00000025203/ ENSMUSG00000010051/ ENSMUSG00000020150/ ENSMUSG00000024640/ ENSMUSG00000021794/ ENSMUSG00000030826/ ENSMUSG00000060600/ ENSMUSG00000032080/ ENSMUSG00000056148/ ENSMUSG00000028307/ ENSMUSG00000002992/ ENSMUSG00000029597/ ENSMUSG00000076441/ ENSMUSG00000023070/ ENSMUSG00000017950/ ENSMUSG00000017146	
GO:0006638	neutral lipid metabolic process	14/350	123/21092	1.75×10^{-08}	1.97×10^{-06}	1.73×10^{-06}	ENSMUSG00000026385/ ENSMUSG00000020609/ ENSMUSG00000018796/ ENSMUSG00000002985/ ENSMUSG00000027761/ ENSMUSG00000032080/ ENSMUSG00000028158/ ENSMUSG00000002944/ ENSMUSG00000040564/ ENSMUSG00000032081/ ENSMUSG00000002992/ ENSMUSG00000011305/ ENSMUSG00000032083/ ENSMUSG00000023070	14
GO:0072329	monocarboxylic acid catabolic process	13/350	111/21092	4.03×10^{-08}	4.38×10^{-06}	3.85×10^{-06}	ENSMUSG00000024507/ ENSMUSG00000090150/ ENSMUSG00000010651/ ENSMUSG00000026385/ ENSMUSG00000003809/ ENSMUSG00000023832/ ENSMUSG00000027332/ ENSMUSG00000036880/ ENSMUSG00000035936/ ENSMUSG00000062908/ ENSMUSG00000021884/ ENSMUSG00000011305/ ENSMUSG00000054422	13
GO:0009062	fatty acid catabolic process	12/350	93/21092	4.51×10^{-08}	4.75×10^{-06}	4.17×10^{-06}	ENSMUSG00000024507/ ENSMUSG00000090150/ ENSMUSG00000010651/ ENSMUSG00000026385/ ENSMUSG00000003809/ ENSMUSG00000023832/ ENSMUSG00000027332/ ENSMUSG00000036880/ ENSMUSG00000062908/ ENSMUSG00000021884/ ENSMUSG00000011305/ ENSMUSG00000054422	12
GO:0009205	purine ribonucleoside triphosphate metabolic process	19/350	252/21092	4.83×10^{-08}	4.79×10^{-06}	4.20×10^{-06}	ENSMUSG00000013593/ ENSMUSG00000034371/ ENSMUSG00000021236/ ENSMUSG00000024248/ ENSMUSG00000050856/ ENSMUSG00000074218/ ENSMUSG00000006057/ ENSMUSG00000054428/ ENSMUSG00000060600/ ENSMUSG00000046329/ ENSMUSG00000073435/ ENSMUSG00000019989/ ENSMUSG00000026576/ ENSMUSG00000032081/ ENSMUSG00000028307/ ENSMUSG00000064370/ ENSMUSG00000049422/ ENSMUSG00000064363/ ENSMUSG00000064367	19

GO:0046034	ATP metabolic process	18/350	227/21092	5.08×10^{-08}	4.79×10^{-06}	4.20×10^{-06}	ENSMUSG00000013593/ ENSMUSG00000034371/ ENSMUSG00000021236/ ENSMUSG00000024248/ ENSMUSG00000050856/ ENSMUSG00000074218/ ENSMUSG00000006057/ ENSMUSG00000054428/ ENSMUSG00000060600/ ENSMUSG00000046329/ ENSMUSG00000019989/ ENSMUSG00000026576/ ENSMUSG00000032081/ ENSMUSG00000028307/ ENSMUSG00000064370/ ENSMUSG00000049422/ ENSMUSG00000064363/ ENSMUSG00000064367	18
GO:0046461	neutral lipid catabolic process	8/350	33/21092	5.13×10^{-08}	4.79×10^{-06}	4.20×10^{-06}	ENSMUSG00000020609/ ENSMUSG00000002985/ ENSMUSG00000027761/ ENSMUSG00000032080/ ENSMUSG00000032081/ ENSMUSG00000002992/ ENSMUSG00000011305/ ENSMUSG00000032083	8
GO:0046464	acylglycerol catabolic process	8/350	33/21092	5.13×10^{-08}	4.79×10^{-06}	4.20×10^{-06}	ENSMUSG00000020609/ ENSMUSG00000002985/ ENSMUSG00000027761/ ENSMUSG00000032080/ ENSMUSG00000032081/ ENSMUSG00000002992/ ENSMUSG00000011305/ ENSMUSG00000032083	8
GO:0009199	ribonucleoside triphosphate metabolic process	19/350	256/21092	6.21×10^{-08}	5.63×10^{-06}	4.94×10^{-06}	ENSMUSG00000013593/ ENSMUSG00000034371/ ENSMUSG00000021236/ ENSMUSG00000024248/ ENSMUSG00000050856/ ENSMUSG00000074218/ ENSMUSG00000006057/ ENSMUSG00000054428/ ENSMUSG00000060600/ ENSMUSG00000046329/ ENSMUSG00000073435/ ENSMUSG00000019989/ ENSMUSG00000026576/ ENSMUSG00000032081/ ENSMUSG00000028307/ ENSMUSG00000064370/ ENSMUSG00000049422/ ENSMUSG00000064363/ ENSMUSG00000064367	19
GO:0055090	acylglycerol homeostasis	8/350	34/21092	6.61×10^{-08}	5.68×10^{-06}	4.99×10^{-06}	ENSMUSG00000002985/ ENSMUSG00000032080/ ENSMUSG00000032081/ ENSMUSG00000002992/ ENSMUSG00000074336/ ENSMUSG00000032083/ ENSMUSG00000059434/ ENSMUSG00000017950	8
GO:0070328	triglyceride homeostasis	8/350	34/21092	6.61×10^{-08}	5.68×10^{-06}	4.99×10^{-06}	ENSMUSG00000002985/ ENSMUSG00000032080/ ENSMUSG00000032081/ ENSMUSG00000002992/ ENSMUSG00000074336/ ENSMUSG00000032083/ ENSMUSG00000059434/ ENSMUSG00000017950	8
GO:0097006	regulation of plasma lipoprotein particle levels	10/350	62/21092	6.96×10^{-08}	5.83×10^{-06}	5.11×10^{-06}	ENSMUSG00000020609/ ENSMUSG00000018800/ ENSMUSG00000002985/ ENSMUSG00000032080/ ENSMUSG00000028158/ ENSMUSG00000023045/	10

							ENSMUSG00000002944/ ENSMUSG00000032081/ ENSMUSG00000002992/ ENSMUSG00000032083	
GO:0009144	purine nucleoside triphosphate metabolic process	19/350	260/21092	7.93×10^{-08}	6.47×10^{-06}	5.68×10^{-06}	ENSMUSG00000013593/ ENSMUSG00000034371/ ENSMUSG00000021236/ ENSMUSG00000024248/ ENSMUSG00000050856/ ENSMUSG00000074218/ ENSMUSG00000006057/ ENSMUSG00000054428/ ENSMUSG00000060600/ ENSMUSG00000046329/ ENSMUSG00000073435/ ENSMUSG00000019989/ ENSMUSG00000026576/ ENSMUSG00000032081/ ENSMUSG00000028307/ ENSMUSG00000064370/ ENSMUSG00000049422/ ENSMUSG00000064363/ ENSMUSG00000064367	19
GO:0016042	lipid catabolic process	20/350	294/21092	1.17×10^{-07}	9.28×10^{-06}	8.15×10^{-06}	ENSMUSG00000024507/ ENSMUSG00000090150/ ENSMUSG00000010651/ ENSMUSG00000026385/ ENSMUSG00000003809/ ENSMUSG00000020609/ ENSMUSG00000023832/ ENSMUSG00000027332/ ENSMUSG00000036880/ ENSMUSG00000002985/ ENSMUSG00000027761/ ENSMUSG00000062908/ ENSMUSG00000032080/ ENSMUSG00000021884/ ENSMUSG00000032081/ ENSMUSG00000002992/ ENSMUSG00000011305/ ENSMUSG00000054422/ ENSMUSG00000032083/ ENSMUSG00000051177	20
GO:0009123	nucleoside monophosphate metabolic process	19/350	267/21092	1.20×10^{-07}	9.36×10^{-06}	8.21×10^{-06}	ENSMUSG00000013593/ ENSMUSG00000034371/ ENSMUSG00000021236/ ENSMUSG00000024248/ ENSMUSG00000050856/ ENSMUSG00000074218/ ENSMUSG00000006057/ ENSMUSG00000054428/ ENSMUSG00000036813/ ENSMUSG00000060600/ ENSMUSG00000046329/ ENSMUSG00000019989/ ENSMUSG00000026576/ ENSMUSG00000032081/ ENSMUSG00000028307/ ENSMUSG00000064370/ ENSMUSG00000049422/ ENSMUSG00000064363/ ENSMUSG00000064367	19
GO:0046503	glycerolipid catabolic process	9/350	51/21092	1.42×10^{-07}	1.06×10^{-05}	9.30×10^{-06}	ENSMUSG00000020609/ ENSMUSG00000002985/ ENSMUSG00000027761/ ENSMUSG00000032080/ ENSMUSG00000032081/ ENSMUSG00000002992/ ENSMUSG00000011305/ ENSMUSG00000032083/ ENSMUSG00000051177	9

GO:0030258	lipid modification	17/350	217/21092	1.43×10^{-07}	1.06×10^{-05}	9.30×10^{-06}	ENSMUSG00000024507/ ENSMUSG00000090150/ ENSMUSG00000010651/ ENSMUSG00000026385/ ENSMUSG00000003809/ ENSMUSG00000023832/ ENSMUSG00000027332/ ENSMUSG00000036880/ ENSMUSG00000002985/ ENSMUSG00000062908/ ENSMUSG00000032080/ ENSMUSG00000021884/ ENSMUSG00000023045/ ENSMUSG00000002944/ ENSMUSG00000011305/ ENSMUSG00000054422/ ENSMUSG00000032083	17
GO:0090208	positive regulation of triglyceride metabolic process	7/350	26/21092	1.64×10^{-07}	1.19×10^{-05}	1.04×10^{-05}	ENSMUSG00000027761/ ENSMUSG00000032080/ ENSMUSG00000002944/ ENSMUSG00000032081/ ENSMUSG00000002992/ ENSMUSG00000011305/ ENSMUSG00000023070	7
GO:0006091	generation of precursor metabolites and energy	22/350	359/21092	1.69×10^{-07}	1.20×10^{-05}	1.05×10^{-05}	ENSMUSG00000002769/ ENSMUSG00000013593/ ENSMUSG00000034371/ ENSMUSG00000021236/ ENSMUSG00000024248/ ENSMUSG00000037710/ ENSMUSG00000067279/ ENSMUSG00000074218/ ENSMUSG00000035936/ ENSMUSG00000062908/ ENSMUSG00000033400/ ENSMUSG00000060600/ ENSMUSG00000026500/ ENSMUSG00000046329/ ENSMUSG00000032081/ ENSMUSG00000028307/ ENSMUSG00000064370/ ENSMUSG00000049422/ ENSMUSG00000064363/ ENSMUSG00000084897/ ENSMUSG00000033208/ ENSMUSG00000064367	22
GO:0009141	nucleoside triphosphate metabolic process	19/350	276/21092	2.02×10^{-07}	1.40×10^{-05}	1.23×10^{-05}	ENSMUSG00000013593/ ENSMUSG00000034371/ ENSMUSG00000021236/ ENSMUSG00000024248/ ENSMUSG00000050856/ ENSMUSG00000074218/ ENSMUSG00000006057/ ENSMUSG00000054428/ ENSMUSG00000060600/ ENSMUSG00000046329/ ENSMUSG00000073435/ ENSMUSG00000019989/ ENSMUSG00000026576/ ENSMUSG00000032081/ ENSMUSG00000028307/ ENSMUSG00000064370/ ENSMUSG00000049422/ ENSMUSG00000064363/ ENSMUSG00000064367	19
GO:0009167	purine ribonucleoside monophosphate metabolic process	18/350	252/21092	2.46×10^{-07}	1.67×10^{-05}	1.47×10^{-05}	ENSMUSG00000013593/ ENSMUSG00000034371/ ENSMUSG00000021236/ ENSMUSG00000024248/ ENSMUSG00000050856/ ENSMUSG00000074218/ ENSMUSG00000006057/ ENSMUSG00000054428/ ENSMUSG00000060600/	18

							ENSMUSG00000046329/ ENSMUSG00000019989/ ENSMUSG00000026576/ ENSMUSG00000032081/ ENSMUSG00000028307/ ENSMUSG00000064370/ ENSMUSG00000049422/ ENSMUSG00000064363/ ENSMUSG00000064367	
GO:0009126	purine nucleoside monophosphate metabolic process	18/350	253/21092	2.61×10^{-07}	1.74×10^{-05}	1.52×10^{-05}	ENSMUSG00000013593/ ENSMUSG00000034371/ ENSMUSG00000021236/ ENSMUSG00000024248/ ENSMUSG00000050856/ ENSMUSG00000074218/ ENSMUSG00000006057/ ENSMUSG00000054428/ ENSMUSG00000060600/ ENSMUSG00000046329/ ENSMUSG00000019989/ ENSMUSG00000026576/ ENSMUSG00000032081/ ENSMUSG00000028307/ ENSMUSG00000064370/ ENSMUSG00000049422/ ENSMUSG00000064363/ ENSMUSG00000064367	18
GO:0009161	ribonucleoside monophosphate metabolic process	18/350	256/21092	3.10×10^{-07}	2.03×10^{-05}	1.78×10^{-05}	ENSMUSG00000013593/ ENSMUSG00000034371/ ENSMUSG00000021236/ ENSMUSG00000024248/ ENSMUSG00000050856/ ENSMUSG00000074218/ ENSMUSG00000006057/ ENSMUSG00000054428/ ENSMUSG00000060600/ ENSMUSG00000046329/ ENSMUSG00000019989/ ENSMUSG00000026576/ ENSMUSG00000032081/ ENSMUSG00000028307/ ENSMUSG00000064370/ ENSMUSG00000049422/ ENSMUSG00000064363/ ENSMUSG00000064367	18
GO:0033344	cholesterol efflux	8/350	43/21092	4.64×10^{-07}	2.97×10^{-05}	2.60×10^{-05}	ENSMUSG00000020609/ ENSMUSG00000018800/ ENSMUSG00000002985/ ENSMUSG00000032080/ ENSMUSG00000040249/ ENSMUSG00000023045/ ENSMUSG00000002992/ ENSMUSG00000032083	8
GO:0090207	regulation of triglyceride metabolic process	8/350	44/21092	5.59×10^{-07}	3.51×10^{-05}	3.08×10^{-05}	ENSMUSG00000002985/ ENSMUSG00000027761/ ENSMUSG00000032080/ ENSMUSG00000002944/ ENSMUSG00000032081/ ENSMUSG00000002992/ ENSMUSG00000011305/ ENSMUSG00000023070	8
GO:0008203	cholesterol metabolic process	12/350	117/21092	5.81×10^{-07}	3.58×10^{-05}	3.14×10^{-05}	ENSMUSG00000020609/ ENSMUSG00000027875/ ENSMUSG00000002985/ ENSMUSG00000032080/ ENSMUSG00000003585/ ENSMUSG00000028158/ ENSMUSG00000040249/ ENSMUSG00000023045/ ENSMUSG00000040564/ ENSMUSG00000032081/ ENSMUSG00000036585/ ENSMUSG00000032083	12
GO:0015918	sterol transport	10/350	78/21092	6.44×10^{-07}	3.82×10^{-05}	3.35×10^{-05}	ENSMUSG00000020609/ ENSMUSG00000018800/ ENSMUSG00000023832/ ENSMUSG00000002985/ ENSMUSG00000032080/ ENSMUSG00000040249/	10

							ENSMUSG00000023045/ ENSMUSG00000002944/ ENSMUSG00000002992/ ENSMUSG00000032083	
GO:0030301	cholesterol transport	10/350	78/21092	6.44×10^{-07}	3.82×10^{-05}	3.35×10^{-05}	ENSMUSG00000020609/ ENSMUSG00000018800/ ENSMUSG00000023832/ ENSMUSG00000002985/ ENSMUSG00000032080/ ENSMUSG00000040249/ ENSMUSG00000023045/ ENSMUSG00000002944/ ENSMUSG00000002992/ ENSMUSG00000032083	10
GO:0019216	regulation of lipid metabolic process	20/350	328/21092	6.69×10^{-07}	3.90×10^{-05}	3.42×10^{-05}	ENSMUSG00000026385/ ENSMUSG00000020609/ ENSMUSG00000036151/ ENSMUSG00000002985/ ENSMUSG00000027761/ ENSMUSG00000024892/ ENSMUSG00000032080/ ENSMUSG00000003585/ ENSMUSG00000002944/ ENSMUSG00000022150/ ENSMUSG00000003555/ ENSMUSG00000032081/ ENSMUSG00000002992/ ENSMUSG00000011305/ ENSMUSG00000036585/ ENSMUSG00000054422/ ENSMUSG00000032083/ ENSMUSG00000023070/ ENSMUSG00000017950/ ENSMUSG00000017146	20
GO:1902652	secondary alcohol metabolic process	12/350	121/21092	8.36×10^{-07}	4.79×10^{-05}	4.20×10^{-05}	ENSMUSG00000020609/ ENSMUSG00000027875/ ENSMUSG00000002985/ ENSMUSG00000032080/ ENSMUSG00000003585/ ENSMUSG00000028158/ ENSMUSG00000040249/ ENSMUSG00000023045/ ENSMUSG00000040564/ ENSMUSG00000032081/ ENSMUSG00000036585/ ENSMUSG00000032083	12
GO:0071827	plasma lipoprotein particle organization	7/350	33/21092	9.62×10^{-07}	5.41×10^{-05}	4.75×10^{-05}	ENSMUSG00000020609/ ENSMUSG00000018800/ ENSMUSG00000002985/ ENSMUSG00000032080/ ENSMUSG00000028158/ ENSMUSG00000023045/ ENSMUSG00000032083	7
GO:0010896	regulation of triglyceride catabolic process	5/350	13/21092	1.41×10^{-06}	7.80×10^{-05}	6.85×10^{-05}	ENSMUSG00000027761/ ENSMUSG00000032080/ ENSMUSG00000032081/ ENSMUSG00000002992/ ENSMUSG00000011305	5
GO:0045940	positive regulation of steroid metabolic process	7/350	35/21092	1.47×10^{-06}	7.90×10^{-05}	6.93×10^{-05}	ENSMUSG00000002985/ ENSMUSG00000032080/ ENSMUSG00000003585/ ENSMUSG00000022150/ ENSMUSG00000003555/ ENSMUSG00000036585/ ENSMUSG00000032083	7
GO:0006066	alcohol metabolic process	18/350	285/21092	1.48×10^{-06}	7.90×10^{-05}	6.93×10^{-05}	ENSMUSG00000034371/ ENSMUSG00000027227/ ENSMUSG00000020609/ ENSMUSG00000046402/ ENSMUSG00000027875/ ENSMUSG00000002985/	18

							ENSMUSG00000032080/ ENSMUSG00000003585/ ENSMUSG00000028158/ ENSMUSG00000040249/ ENSMUSG00000023045/ ENSMUSG00000022150/ ENSMUSG00000040564/ ENSMUSG00000032081/ ENSMUSG00000028743/ ENSMUSG00000036585/ ENSMUSG00000032083/ ENSMUSG00000051177	
GO:0016125	sterol metabolic process	12/350	128/21092	1.53×10^{-06}	8.05×10^{-05}	7.06×10^{-05}	ENSMUSG00000020609/ ENSMUSG00000027875/ ENSMUSG00000002985/ ENSMUSG00000032080/ ENSMUSG00000003585/ ENSMUSG00000028158/ ENSMUSG00000040249/ ENSMUSG00000023045/ ENSMUSG00000040564/ ENSMUSG00000032081/ ENSMUSG00000036585/ ENSMUSG00000032083	12
GO:0071825	protein−lipid complex subunit organization	7/350	37/21092	2.19×10^{-06}	0.0001	9.95×10^{-05}	ENSMUSG00000020609/ ENSMUSG00000018800/ ENSMUSG00000002985/ ENSMUSG00000032080/ ENSMUSG00000028158/ ENSMUSG00000023045/ ENSMUSG00000032083	7
GO:1901615	organic hydroxy compound metabolic process	23/350	453/21092	2.33×10^{-06}	0.0001	0.0001	ENSMUSG00000024140/ ENSMUSG00000034371/ ENSMUSG00000027227/ ENSMUSG00000020609/ ENSMUSG00000046402/ ENSMUSG00000001666/ ENSMUSG00000027875/ ENSMUSG00000002985/ ENSMUSG00000032080/ ENSMUSG00000003585/ ENSMUSG00000028158/ ENSMUSG00000040249/ ENSMUSG00000023045/ ENSMUSG00000022150/ ENSMUSG00000040564/ ENSMUSG00000032081/ ENSMUSG00000028743/ ENSMUSG00000036585/ ENSMUSG00000038216/ ENSMUSG00000032083/ ENSMUSG00000051177/ ENSMUSG00000020182/ ENSMUSG00000084897	23
GO:0006869	lipid transport	19/350	325/21092	2.38×10^{-06}	0.0001	0.0001	ENSMUSG00000020609/ ENSMUSG00000018800/ ENSMUSG00000018796/ ENSMUSG00000023832/ ENSMUSG00000032120/ ENSMUSG00000002985/ ENSMUSG00000032080/ ENSMUSG00000028158/ ENSMUSG00000040249/ ENSMUSG00000023045/ ENSMUSG00000002944/ ENSMUSG00000022150/ ENSMUSG00000040564/ ENSMUSG00000032081/ ENSMUSG00000002992/ ENSMUSG00000054422/ ENSMUSG00000074336/ ENSMUSG00000032083/ ENSMUSG00000049791	19

GO:0055088	lipid homeostasis	12/350	135/21092	2.69 × 10⁻⁰⁶	0.0001	0.0001	ENSMUSG00000020609/ ENSMUSG00000046402/ ENSMUSG00000002985/ ENSMUSG00000032080/ ENSMUSG00000028158/ ENSMUSG00000023045/ ENSMUSG00000032081/ ENSMUSG00000002992/ ENSMUSG00000074336/ ENSMUSG00000032083/ ENSMUSG00000059434/ ENSMUSG00000017950	12
GO:0042953	lipoprotein transport	5/350	15/21092	3.20 × 10⁻⁰⁶	0.0002	0.0001	ENSMUSG00000020609/ ENSMUSG00000028158/ ENSMUSG00000002944/ ENSMUSG00000032081/ ENSMUSG00000002992	5
GO:0044241	lipid digestion	5/350	15/21092	3.20 × 10⁻⁰⁶	0.0002	0.0001	ENSMUSG00000023832/ ENSMUSG00000032080/ ENSMUSG00000004655/ ENSMUSG00000002944/ ENSMUSG00000032083	5
GO:0044872	lipoprotein localization	5/350	15/21092	3.20 × 10⁻⁰⁶	0.0002	0.0001	ENSMUSG00000020609/ ENSMUSG00000028158/ ENSMUSG00000002944/ ENSMUSG00000032081/ ENSMUSG00000002992	5
GO:0010876	lipid localization	20/350	364/21092	3.32 × 10⁻⁰⁶	0.0002	0.0001	ENSMUSG00000020609/ ENSMUSG00000018800/ ENSMUSG00000018796/ ENSMUSG00000023832/ ENSMUSG00000032120/ ENSMUSG00000002985/ ENSMUSG00000032080/ ENSMUSG00000028158/ ENSMUSG00000040249/ ENSMUSG00000023045/ ENSMUSG00000002944/ ENSMUSG00000022150/ ENSMUSG00000040564/ ENSMUSG00000032081/ ENSMUSG00000002992/ ENSMUSG00000011305/ ENSMUSG00000054422/ ENSMUSG00000074336/ ENSMUSG00000032083/ ENSMUSG00000049791	20
GO:0006695	cholesterol biosynthetic process	7/350	42/21092	5.34 × 10⁻⁰⁶	0.0002	0.0002	ENSMUSG00000020609/ ENSMUSG00000027875/ ENSMUSG00000002985/ ENSMUSG00000032080/ ENSMUSG00000003585/ ENSMUSG00000036585/ ENSMUSG00000032083	7
GO:0009066	aspartate family amino acid metabolic process	7/350	42/21092	5.34 × 10⁻⁰⁶	0.0002	0.0002	ENSMUSG00000002769/ ENSMUSG00000028179/ ENSMUSG00000116378/ ENSMUSG00000006378/ ENSMUSG00000017453/ ENSMUSG00000029597/ ENSMUSG00000076441	7
GO:1902653	secondary alcohol biosynthetic process	7/350	43/21092	6.29 × 10⁻⁰⁶	0.0003	0.0002	ENSMUSG00000020609/ ENSMUSG00000027875/ ENSMUSG00000002985/ ENSMUSG00000032080/ ENSMUSG00000003585/ ENSMUSG00000036585/ ENSMUSG00000032083	7

GO:0046486	glycerolipid metabolic process	18/350	325/21092	9.23×10^{-06}	0.0004	0.0004	ENSMUSG00000026385/ ENSMUSG00000020609/ ENSMUSG00000073678/ ENSMUSG00000018796/ ENSMUSG00000002985/ ENSMUSG00000035936/ ENSMUSG00000027761/ ENSMUSG00000032080/ ENSMUSG00000028158/ ENSMUSG00000002944/ ENSMUSG00000042737/ ENSMUSG00000040564/ ENSMUSG00000032081/ ENSMUSG00000002992/ ENSMUSG00000011305/ ENSMUSG00000032083/ ENSMUSG00000051177/ ENSMUSG00000023070	18
GO:0042180	cellular ketone metabolic process	13/350	184/21092	1.31×10^{-05}	0.0006	0.0005	ENSMUSG00000026385/ ENSMUSG00000039648/ ENSMUSG00000026489/ ENSMUSG00000032080/ ENSMUSG00000022150/ ENSMUSG00000003555/ ENSMUSG00000002992/ ENSMUSG00000011305/ ENSMUSG00000028743/ ENSMUSG00000054422/ ENSMUSG00000023070/ ENSMUSG00000017950/ ENSMUSG00000017146	13
GO:0006000	fructose metabolic process	4/350	10/21092	1.45×10^{-05}	0.0006	0.0005	ENSMUSG00000034371/ ENSMUSG00000027227/ ENSMUSG00000028307/ ENSMUSG00000021456	4
GO:0072330	monocarboxylic acid biosynthetic process	15/350	244/21092	1.53×10^{-05}	0.0006	0.0006	ENSMUSG00000021620/ ENSMUSG00000034371/ ENSMUSG00000046402/ ENSMUSG00000021236/ ENSMUSG00000041220/ ENSMUSG00000025203/ ENSMUSG00000060600/ ENSMUSG00000032080/ ENSMUSG00000056148/ ENSMUSG00000028307/ ENSMUSG00000002992/ ENSMUSG00000029597/ ENSMUSG00000023070/ ENSMUSG00000017950/ ENSMUSG00000017146	15
GO:0034368	protein−lipid complex remodeling	5/350	20/21092	1.54×10^{-05}	0.0006	0.0006	ENSMUSG00000020609/ ENSMUSG00000018800/ ENSMUSG00000002985/ ENSMUSG00000032080/ ENSMUSG00000032083	5
GO:0034369	plasma lipoprotein particle remodeling	5/350	20/21092	1.54×10^{-05}	0.0006	0.0006	ENSMUSG00000020609/ ENSMUSG00000018800/ ENSMUSG00000002985/ ENSMUSG00000032080/ ENSMUSG00000032083	5
GO:0034377	plasma lipoprotein particle assembly	5/350	20/21092	1.54×10^{-05}	0.0006	0.0006	ENSMUSG00000002985/ ENSMUSG00000032080/ ENSMUSG00000028158/ ENSMUSG00000023045/ ENSMUSG00000032083	5
GO:0016126	sterol biosynthetic process	7/350	50/21092	1.76×10^{-05}	0.0007	0.0006	ENSMUSG00000020609/ ENSMUSG00000027875/ ENSMUSG00000002985/ ENSMUSG00000032080/ ENSMUSG00000003585/ ENSMUSG00000036585/ ENSMUSG00000032083	7

GO:0033539	fatty acid beta−oxidation using acyl−CoA dehydrogenase	4/350	11/21092	2.24×10^{-05}	0.0009	0.0008	ENSMUSG00000090150/ ENSMUSG00000003809/ ENSMUSG00000027332/ ENSMUSG00000062908	4
GO:0033700	phospholipid efflux	4/350	11/21092	2.24×10^{-05}	0.0009	0.0008	ENSMUSG00000002985/ ENSMUSG00000032080/ ENSMUSG00000002992/ ENSMUSG00000032083	4
GO:0001676	long−chain fatty acid metabolic process	9/350	93/21092	2.43×10^{-05}	0.0009	0.0008	ENSMUSG00000021620/ ENSMUSG00000025002/ ENSMUSG00000041220/ ENSMUSG00000018796/ ENSMUSG00000002944/ ENSMUSG00000094806/ ENSMUSG00000022445/ ENSMUSG00000062624/ ENSMUSG00000067231	9
GO:0008202	steroid metabolic process	16/350	285/21092	2.45×10^{-05}	0.0009	0.0008	ENSMUSG00000024507/ ENSMUSG00000020609/ ENSMUSG00000034308/ ENSMUSG00000027875/ ENSMUSG00000002985/ ENSMUSG00000032080/ ENSMUSG00000003585/ ENSMUSG00000028158/ ENSMUSG00000040249/ ENSMUSG00000023045/ ENSMUSG00000022150/ ENSMUSG00000040564/ ENSMUSG00000003555/ ENSMUSG00000032081/ ENSMUSG00000036585/ ENSMUSG00000032083	16
GO:0034367	protein−containing complex remodeling	5/350	22/21092	2.55×10^{-05}	0.0010	0.0009	ENSMUSG00000020609/ ENSMUSG00000018800/ ENSMUSG00000002985/ ENSMUSG00000032080/ ENSMUSG00000032083	5
GO:0006119	oxidative phosphorylation	9/350	94/21092	2.65×10^{-05}	0.0010	0.0009	ENSMUSG00000013593/ ENSMUSG00000024248/ ENSMUSG00000074218/ ENSMUSG00000046329/ ENSMUSG00000032081/ ENSMUSG00000064370/ ENSMUSG00000049422/ ENSMUSG00000064363/ ENSMUSG00000064367	9
GO:1905952	regulation of lipid localization	11/350	143/21092	2.76×10^{-05}	0.0010	0.0009	ENSMUSG00000020609/ ENSMUSG00000023832/ ENSMUSG00000002985/ ENSMUSG00000032080/ ENSMUSG00000040249/ ENSMUSG00000002944/ ENSMUSG00000022150/ ENSMUSG00000002992/ ENSMUSG00000011305/ ENSMUSG00000074336/ ENSMUSG00000032083	11
GO:0060191	regulation of lipase activity	8/350	73/21092	2.79×10^{-05}	0.0010	0.0009	ENSMUSG00000045875/ ENSMUSG00000032080/ ENSMUSG00000040249/ ENSMUSG00000032081/ ENSMUSG00000002992/ ENSMUSG00000011305/ ENSMUSG00000068747/ ENSMUSG00000032083	8

GO:0050994	regulation of lipid catabolic process	7/350	54/21092	2.96×10^{-05}	0.0011	0.0009	ENSMUSG00000026385/ ENSMUSG00000027761/ ENSMUSG00000032080/ ENSMUSG00000032081/ ENSMUSG00000002992/ ENSMUSG00000011305/ ENSMUSG00000054422	7
GO:0033865	nucleoside bisphosphate metabolic process	9/350	98/21092	3.70×10^{-05}	0.0013	0.0011	ENSMUSG00000024507/ ENSMUSG00000021620/ ENSMUSG00000003809/ ENSMUSG00000018796/ ENSMUSG00000027875/ ENSMUSG00000036880/ ENSMUSG00000017453/ ENSMUSG00000033610/ ENSMUSG00000017950	9
GO:0033875	ribonucleoside bisphosphate metabolic process	9/350	98/21092	3.70×10^{-05}	0.0013	0.0011	ENSMUSG00000024507/ ENSMUSG00000021620/ ENSMUSG00000003809/ ENSMUSG00000018796/ ENSMUSG00000027875/ ENSMUSG00000036880/ ENSMUSG00000017453/ ENSMUSG00000033610/ ENSMUSG00000017950	9
GO:0034032	purine nucleoside bisphosphate metabolic process	9/350	98/21092	3.70×10^{-05}	0.0013	0.0011	ENSMUSG00000024507/ ENSMUSG00000021620/ ENSMUSG00000003809/ ENSMUSG00000018796/ ENSMUSG00000027875/ ENSMUSG00000036880/ ENSMUSG00000017453/ ENSMUSG00000033610/ ENSMUSG00000017950	9
GO:0065005	protein−lipid complex assembly	5/350	24/21092	4.01×10^{-05}	0.0014	0.0012	ENSMUSG00000002985/ ENSMUSG00000032080/ ENSMUSG00000028158/ ENSMUSG00000023045/ ENSMUSG00000032083	5
GO:0006805	xenobiotic metabolic process	9/350	100/21092	4.35×10^{-05}	0.0015	0.0013	ENSMUSG00000010651/ ENSMUSG00000025002/ ENSMUSG00000018796/ ENSMUSG00000022809/ ENSMUSG00000094806/ ENSMUSG00000022445/ ENSMUSG00000062624/ ENSMUSG00000067231/ ENSMUSG00000017950	9
GO:0009068	aspartate family amino acid catabolic process	4/350	13/21092	4.73×10^{-05}	0.0016	0.0014	ENSMUSG00000116378/ ENSMUSG00000006378/ ENSMUSG00000017453/ ENSMUSG00000029597	4
GO:0030299	intestinal cholesterol absorption	4/350	13/21092	4.73×10^{-05}	0.0016	0.0014	ENSMUSG00000023832/ ENSMUSG00000032080/ ENSMUSG00000002944/ ENSMUSG00000032083	4
GO:0043691	reverse cholesterol transport	4/350	13/21092	4.73×10^{-05}	0.0016	0.0014	ENSMUSG00000018800/ ENSMUSG00000002985/ ENSMUSG00000032080/ ENSMUSG00000032083	4
GO:0045923	positive regulation of fatty acid metabolic process	6/350	40/21092	4.77×10^{-05}	0.0016	0.0014	ENSMUSG00000032080/ ENSMUSG00000002992/ ENSMUSG00000011305/ ENSMUSG00000054422/ ENSMUSG00000023070/ ENSMUSG00000017950	6
GO:0050996	positive regulation of lipid catabolic process	5/350	25/21092	4.94×10^{-05}	0.0016	0.0014	ENSMUSG00000027761/ ENSMUSG00000032080/ ENSMUSG00000002992/ ENSMUSG00000011305/ ENSMUSG00000054422	5

GO:0046364	monosaccharide biosynthetic process	8/350	79/21092	4.97×10^{-05}	0.0016	0.0014	ENSMUSG00000002769/ ENSMUSG00000027227/ ENSMUSG00000024892/ ENSMUSG00000062908/ ENSMUSG00000021456/ ENSMUSG00000029597/ ENSMUSG00000023070/ ENSMUSG00000017950	8
GO:1901617	organic hydroxy compound biosynthetic process	13/350	212/21092	5.77×10^{-05}	0.0018	0.0016	ENSMUSG00000024140/ ENSMUSG00000020609/ ENSMUSG00000001666/ ENSMUSG00000027875/ ENSMUSG00000002985/ ENSMUSG00000032080/ ENSMUSG00000003585/ ENSMUSG00000022150/ ENSMUSG00000036585/ ENSMUSG00000038216/ ENSMUSG00000032083/ ENSMUSG00000020182/ ENSMUSG00000084897	13
GO:1901607	alpha−amino acid biosynthetic process	7/350	60/21092	5.92×10^{-05}	0.0019	0.0016	ENSMUSG00000028179/ ENSMUSG00000031173/ ENSMUSG00000020150/ ENSMUSG00000024640/ ENSMUSG00000021794/ ENSMUSG00000030826/ ENSMUSG00000076441	7
GO:0006637	acyl−CoA metabolic process	8/350	81/21092	5.95×10^{-05}	0.0019	0.0016	ENSMUSG00000024507/ ENSMUSG00000021620/ ENSMUSG00000003809/ ENSMUSG00000018796/ ENSMUSG00000027875/ ENSMUSG00000036880/ ENSMUSG00000017453/ ENSMUSG00000017950	8
GO:0035383	thioester metabolic process	8/350	81/21092	5.95×10^{-05}	0.0019	0.0016	ENSMUSG00000024507/ ENSMUSG00000021620/ ENSMUSG00000003809/ ENSMUSG00000018796/ ENSMUSG00000027875/ ENSMUSG00000036880/ ENSMUSG00000017453/ ENSMUSG00000017950	8
GO:0019217	regulation of fatty acid metabolic process	8/350	82/21092	6.50×10^{-05}	0.0019	0.0017	ENSMUSG00000026385/ ENSMUSG00000032080/ ENSMUSG00000002992/ ENSMUSG00000011305/ ENSMUSG00000054422/ ENSMUSG00000023070/ ENSMUSG00000017950/ ENSMUSG00000017146	8
GO:0034433	steroid esterification	4/350	14/21092	6.54×10^{-05}	0.0019	0.0017	ENSMUSG00000002985/ ENSMUSG00000032080/ ENSMUSG00000023045/ ENSMUSG00000032083	4
GO:0034434	sterol esterification	4/350	14/21092	6.54×10^{-05}	0.0019	0.0017	ENSMUSG00000002985/ ENSMUSG00000032080/ ENSMUSG00000023045/ ENSMUSG00000032083	4
GO:0034435	cholesterol esterification	4/350	14/21092	6.54×10^{-05}	0.0019	0.0017	ENSMUSG00000002985/ ENSMUSG00000032080/ ENSMUSG00000023045/ ENSMUSG00000032083	4

GO:0051004	regulation of lipoprotein lipase activity	4/350	14/21092	6.54×10^{-05}	0.0019	0.0017	ENSMUSG00000032080/ ENSMUSG00000032081/ ENSMUSG00000002992/ ENSMUSG00000068747	4
GO:0051271	negative regulation of cellular component movement	16/350	311/21092	6.97×10^{-05}	0.0021	0.0018	ENSMUSG00000001666/ ENSMUSG00000028927/ ENSMUSG00000002985/ ENSMUSG00000030257/ ENSMUSG00000031389/ ENSMUSG00000040249/ ENSMUSG00000010047/ ENSMUSG00000025810/ ENSMUSG00000034675/ ENSMUSG00000000753/ ENSMUSG00000032702/ ENSMUSG00000112129/ ENSMUSG00000027805/ ENSMUSG00000051177/ ENSMUSG00000023070/ ENSMUSG00000017950	16
GO:0042632	cholesterol homeostasis	8/350	83/21092	7.09×10^{-05}	0.0021	0.0018	ENSMUSG00000020609/ ENSMUSG00000002985/ ENSMUSG00000032080/ ENSMUSG00000028158/ ENSMUSG00000023045/ ENSMUSG00000032081/ ENSMUSG00000032083/ ENSMUSG00000017950	8
GO:0032371	regulation of sterol transport	6/350	43/21092	7.26×10^{-05}	0.0021	0.0018	ENSMUSG00000023832/ ENSMUSG00000002985/ ENSMUSG00000032080/ ENSMUSG00000040249/ ENSMUSG00000002992/ ENSMUSG00000032083	6
GO:0032374	regulation of cholesterol transport	6/350	43/21092	7.26×10^{-05}	0.0021	0.0018	ENSMUSG00000023832/ ENSMUSG00000002985/ ENSMUSG00000032080/ ENSMUSG00000040249/ ENSMUSG00000002992/ ENSMUSG00000032083	6
GO:0055092	sterol homeostasis	8/350	84/21092	7.73×10^{-05}	0.0022	0.0019	ENSMUSG00000020609/ ENSMUSG00000002985/ ENSMUSG00000032080/ ENSMUSG00000028158/ ENSMUSG00000023045/ ENSMUSG00000032081/ ENSMUSG00000032083/ ENSMUSG00000017950	8
GO:0008652	cellular amino acid biosynthetic process	7/350	63/21092	8.13×10^{-05}	0.0023	0.0020	ENSMUSG00000028179/ ENSMUSG00000031173/ ENSMUSG00000020150/ ENSMUSG00000024640/ ENSMUSG00000021794/ ENSMUSG00000030826/ ENSMUSG00000076441	7
GO:0060192	negative regulation of lipase activity	4/350	15/21092	8.80×10^{-05}	0.0024	0.0021	ENSMUSG00000032081/ ENSMUSG00000011305/ ENSMUSG00000068747/ ENSMUSG00000032083	4
GO:0098856	intestinal lipid absorption	4/350	15/21092	8.80×10^{-05}	0.0024	0.0021	ENSMUSG00000023832/ ENSMUSG00000032080/ ENSMUSG00000002944/ ENSMUSG00000032083	4
GO:0019318	hexose metabolic process	13/350	221/21092	8.81×10^{-05}	0.0024	0.0021	ENSMUSG00000002769/ ENSMUSG00000034371/ ENSMUSG00000027227/ ENSMUSG00000035936/ ENSMUSG00000024892/ ENSMUSG00000062908/	13

							ENSMUSG00000039450/ ENSMUSG00000028307/ ENSMUSG00000021456/ ENSMUSG00000029597/ ENSMUSG00000059434/ ENSMUSG00000023070/ ENSMUSG00000017950	
GO:0006575	cellular modified amino acid metabolic process	11/350	165/21092	0.0001	0.0028	0.0024	ENSMUSG00000002769/ ENSMUSG00000028179/ ENSMUSG00000039648/ ENSMUSG00000035936/ ENSMUSG00000020150/ ENSMUSG00000062908/ ENSMUSG00000009614/ ENSMUSG00000017453/ ENSMUSG00000026853/ ENSMUSG00000024579/ ENSMUSG00000076441	11
GO:1905954	positive regulation of lipid localization	8/350	88/21092	0.0001	0.0029	0.0025	ENSMUSG00000020609/ ENSMUSG00000023832/ ENSMUSG00000002985/ ENSMUSG00000040249/ ENSMUSG00000002944/ ENSMUSG00000022150/ ENSMUSG00000011305/ ENSMUSG00000074336	8
GO:0045540	regulation of cholesterol biosynthetic process	4/350	16/21092	0.0001	0.0031	0.0027	ENSMUSG00000020609/ ENSMUSG00000002985/ ENSMUSG00000003585/ ENSMUSG00000036585	4
GO:0106118	regulation of sterol biosynthetic process	4/350	16/21092	0.0001	0.0031	0.0027	ENSMUSG00000020609/ ENSMUSG00000002985/ ENSMUSG00000003585/ ENSMUSG00000036585	4
GO:0043062	extracellular structure organization	15/350	293/21092	0.0001	0.0033	0.0029	ENSMUSG00000020609/ ENSMUSG00000040998/ ENSMUSG00000018800/ ENSMUSG00000010051/ ENSMUSG00000002985/ ENSMUSG00000032080/ ENSMUSG00000006931/ ENSMUSG00000028158/ ENSMUSG00000023045/ ENSMUSG00000002944/ ENSMUSG00000045672/ ENSMUSG00000006538/ ENSMUSG00000032083/ ENSMUSG00000027750/ ENSMUSG00000038224	15
GO:0040013	negative regulation of locomotion	16/350	328/21092	0.0001	0.0034	0.0030	ENSMUSG00000001666/ ENSMUSG00000028927/ ENSMUSG00000002985/ ENSMUSG00000030257/ ENSMUSG00000031389/ ENSMUSG00000040249/ ENSMUSG00000010047/ ENSMUSG00000025810/ ENSMUSG00000034675/ ENSMUSG00000000753/ ENSMUSG00000032702/ ENSMUSG00000112129/ ENSMUSG00000027805/ ENSMUSG00000051177/ ENSMUSG00000023070/ ENSMUSG00000017950	16
GO:0006790	sulfur compound metabolic process	14/350	265/21092	0.0001	0.0039	0.0034	ENSMUSG00000024507/ ENSMUSG00000002769/ ENSMUSG00000021620/ ENSMUSG00000003809/ ENSMUSG00000028179/ ENSMUSG00000018796/	14

							ENSMUSG00000027875/ ENSMUSG00000036880/ ENSMUSG00000035936/ ENSMUSG00000020150/ ENSMUSG00000017453/ ENSMUSG00000024579/ ENSMUSG00000049858/ ENSMUSG00000017950	
GO:0062013	positive regulation of small molecule metabolic process	10/350	146/21092	0.0002	0.0042	0.0037	ENSMUSG00000021236/ ENSMUSG00000032080/ ENSMUSG00000003585/ ENSMUSG00000022150/ ENSMUSG00000002992/ ENSMUSG00000011305/ ENSMUSG00000036585/ ENSMUSG00000054422/ ENSMUSG00000023070/ ENSMUSG00000017950	10
GO:0071466	cellular response to xenobiotic stimulus	10/350	146/21092	0.0002	0.0042	0.0037	ENSMUSG00000010651/ ENSMUSG00000025002/ ENSMUSG00000018796/ ENSMUSG00000022809/ ENSMUSG00000004655/ ENSMUSG00000094806/ ENSMUSG00000022445/ ENSMUSG00000062624/ ENSMUSG00000067231/ ENSMUSG00000017950	10
GO:0010565	regulation of cellular ketone metabolic process	9/350	119/21092	0.0002	0.0042	0.0037	ENSMUSG00000026385/ ENSMUSG00000032080/ ENSMUSG00000022150/ ENSMUSG00000002992/ ENSMUSG00000011305/ ENSMUSG00000054422/ ENSMUSG00000023070/ ENSMUSG00000017950/ ENSMUSG00000017146	9
GO:0042157	lipoprotein metabolic process	9/350	121/21092	0.0002	0.0048	0.0042	ENSMUSG00000020609/ ENSMUSG00000073678/ ENSMUSG00000002985/ ENSMUSG00000032080/ ENSMUSG00000028158/ ENSMUSG00000042737/ ENSMUSG00000040564/ ENSMUSG00000032081/ ENSMUSG00000032083	9
GO:0015850	organic hydroxy compound transport	13/350	239/21092	0.0002	0.0048	0.0042	ENSMUSG00000020609/ ENSMUSG00000018800/ ENSMUSG00000073678/ ENSMUSG00000023832/ ENSMUSG00000002985/ ENSMUSG00000032080/ ENSMUSG00000040249/ ENSMUSG00000023045/ ENSMUSG00000004655/ ENSMUSG00000002944/ ENSMUSG00000022150/ ENSMUSG00000002992/ ENSMUSG00000032083	13
GO:0019319	hexose biosynthetic process	7/350	73/21092	0.0002	0.0051	0.0045	ENSMUSG00000002769/ ENSMUSG00000027227/ ENSMUSG00000024892/ ENSMUSG00000062908/ ENSMUSG00000021456/ ENSMUSG00000029597/ ENSMUSG00000017950	7
GO:0042737	drug catabolic process	10/350	150/21092	0.0002	0.0051	0.0045	ENSMUSG00000025002/ ENSMUSG00000034424/ ENSMUSG00000027333/ ENSMUSG00000022809/ ENSMUSG00000032080/ ENSMUSG00000017453/	10

							ENSMUSG00000094806/ ENSMUSG00000022445/ ENSMUSG00000062624/ ENSMUSG00000067231	
GO:1900024	regulation of substrate adhesion−dependent cell spreading	6/350	52/21092	0.0002	0.0052	0.0046	ENSMUSG00000022150/ ENSMUSG00000025810/ ENSMUSG00000034675/ ENSMUSG00000032702/ ENSMUSG00000032083/ ENSMUSG00000027750	6
GO:0006694	steroid biosynthetic process	10/350	152/21092	0.0002	0.0056	0.0049	ENSMUSG00000020609/ ENSMUSG00000034308/ ENSMUSG00000027875/ ENSMUSG00000002985/ ENSMUSG00000032080/ ENSMUSG00000003585/ ENSMUSG00000022150/ ENSMUSG00000003555/ ENSMUSG00000036585/ ENSMUSG00000032083	10
GO:2000146	negative regulation of cell motility	14/350	277/21092	0.0002	0.0057	0.0050	ENSMUSG00000001666/ ENSMUSG00000028927/ ENSMUSG00000002985/ ENSMUSG00000030257/ ENSMUSG00000031389/ ENSMUSG00000040249/ ENSMUSG00000010047/ ENSMUSG00000000753/ ENSMUSG00000032702/ ENSMUSG00000112129/ ENSMUSG00000027805/ ENSMUSG00000051177/ ENSMUSG00000023070/ ENSMUSG00000017950	14
GO:0042738	exogenous drug catabolic process	6/350	53/21092	0.0002	0.0057	0.0050	ENSMUSG00000025002/ ENSMUSG00000022809/ ENSMUSG00000094806/ ENSMUSG00000022445/ ENSMUSG00000062624/ ENSMUSG00000067231	6
GO:0019218	regulation of steroid metabolic process	8/350	101/21092	0.0003	0.0066	0.0058	ENSMUSG00000020609/ ENSMUSG00000002985/ ENSMUSG00000032080/ ENSMUSG00000003585/ ENSMUSG00000022150/ ENSMUSG00000003555/ ENSMUSG00000036585/ ENSMUSG00000032083	8
GO:0005996	monosaccharide metabolic process	13/350	250/21092	0.0003	0.0070	0.0061	ENSMUSG00000002769/ ENSMUSG00000034371/ ENSMUSG00000027227/ ENSMUSG00000035936/ ENSMUSG00000024892/ ENSMUSG00000062908/ ENSMUSG00000039450/ ENSMUSG00000028307/ ENSMUSG00000021456/ ENSMUSG00000029597/ ENSMUSG00000059434/ ENSMUSG00000023070/ ENSMUSG00000017950	13
GO:0034381	plasma lipoprotein particle clearance	5/350	36/21092	0.0003	0.0070	0.0061	ENSMUSG00000020609/ ENSMUSG00000002985/ ENSMUSG00000002944/ ENSMUSG00000032081/ ENSMUSG00000002992	5
GO:0050892	intestinal absorption	5/350	36/21092	0.0003	0.0070	0.0061	ENSMUSG00000023832/ ENSMUSG00000032080/ ENSMUSG00000002944/ ENSMUSG00000054422/ ENSMUSG00000032083	5

GO:0015980	energy derivation by oxidation of organic compounds	13/350	252/21092	0.0003	0.0074	0.0065	ENSMUSG00000002769/ ENSMUSG00000013593/ ENSMUSG00000037710/ ENSMUSG00000067279/ ENSMUSG00000035936/ ENSMUSG00000062908/ ENSMUSG00000033400/ ENSMUSG00000026500/ ENSMUSG00000046329/ ENSMUSG00000064370/ ENSMUSG00000064363/ ENSMUSG00000033208/ ENSMUSG00000064367	13
GO:0051346	negative regulation of hydrolase activity	17/350	395/21092	0.0004	0.0081	0.0071	ENSMUSG00000054428/ ENSMUSG00000052572/ ENSMUSG00000025650/ ENSMUSG00000028415/ ENSMUSG00000004655/ ENSMUSG00000032081/ ENSMUSG00000011305/ ENSMUSG00000034675/ ENSMUSG00000066366/ ENSMUSG00000000753/ ENSMUSG00000068747/ ENSMUSG00000044433/ ENSMUSG00000006522/ ENSMUSG00000054422/ ENSMUSG00000032083/ ENSMUSG00000023070/ ENSMUSG00000038224	17
GO:0042304	regulation of fatty acid biosynthetic process	5/350	38/21092	0.0004	0.0088	0.0078	ENSMUSG00000032080/ ENSMUSG00000002992/ ENSMUSG00000023070/ ENSMUSG00000017950/ ENSMUSG00000017146	5
GO:0090407	organophosphate biosynthetic process	19/350	474/21092	0.0004	0.0088	0.0078	ENSMUSG00000034371/ ENSMUSG00000003809/ ENSMUSG00000020649/ ENSMUSG00000021236/ ENSMUSG00000073678/ ENSMUSG00000050856/ ENSMUSG00000018796/ ENSMUSG00000027875/ ENSMUSG00000025137/ ENSMUSG00000006057/ ENSMUSG00000024892/ ENSMUSG00000036813/ ENSMUSG00000060600/ ENSMUSG00000073435/ ENSMUSG00000033610/ ENSMUSG00000042737/ ENSMUSG00000028307/ ENSMUSG00000049422/ ENSMUSG00000032083	19
GO:0043462	regulation of ATPase activity	6/350	59/21092	0.0004	0.0096	0.0084	ENSMUSG00000054428/ ENSMUSG00000026576/ ENSMUSG00000028307/ ENSMUSG00000034675/ ENSMUSG00000027805/ ENSMUSG00000023070	6
GO:0045723	positive regulation of fatty acid biosynthetic process	4/350	22/21092	0.0004	0.0096	0.0084	ENSMUSG00000032080/ ENSMUSG00000002992/ ENSMUSG00000023070/ ENSMUSG00000017950	4
GO:0030336	negative regulation of cell migration	13/350	263/21092	0.0005	0.0106	0.0093	ENSMUSG00000001666/ ENSMUSG00000028927/ ENSMUSG00000002985/ ENSMUSG00000030257/ ENSMUSG00000031389/ ENSMUSG00000040249/ ENSMUSG00000010047/ ENSMUSG00000000753/ ENSMUSG00000032702/	13

							ENSMUSG00000112129/ ENSMUSG00000027805/ ENSMUSG00000051177/ ENSMUSG00000017950	
GO:0034380	high−density lipoprotein particle assembly	3/350	10/21092	0.0005	0.0108	0.0095	ENSMUSG00000002985/ ENSMUSG00000032080/ ENSMUSG00000032083	3
GO:0090209	negative regulation of triglyceride metabolic process	3/350	10/21092	0.0005	0.0108	0.0095	ENSMUSG00000002985/ ENSMUSG00000032081/ ENSMUSG00000011305	3
GO:0098754	detoxification	5/350	40/21092	0.0005	0.0108	0.0095	ENSMUSG00000010651/ ENSMUSG00000031765/ ENSMUSG00000032080/ ENSMUSG00000002944/ ENSMUSG00000023070	5
GO:0010884	positive regulation of lipid storage	4/350	23/21092	0.0005	0.0110	0.0097	ENSMUSG00000020609/ ENSMUSG00000002944/ ENSMUSG00000011305/ ENSMUSG00000074336	4
GO:0009152	purine ribonucleotide biosynthetic process	11/350	202/21092	0.0006	0.0125	0.0109	ENSMUSG00000034371/ ENSMUSG00000003809/ ENSMUSG00000021236/ ENSMUSG00000050856/ ENSMUSG00000018796/ ENSMUSG00000006057/ ENSMUSG00000060600/ ENSMUSG00000073435/ ENSMUSG00000033610/ ENSMUSG00000028307/ ENSMUSG00000049422	11
GO:0007005	mitochondrion organization	18/350	452/21092	0.0006	0.0127	0.0111	ENSMUSG00000024140/ ENSMUSG00000034993/ ENSMUSG00000031059/ ENSMUSG00000024248/ ENSMUSG00000049760/ ENSMUSG00000074218/ ENSMUSG00000037152/ ENSMUSG00000016427/ ENSMUSG00000036880/ ENSMUSG00000054428/ ENSMUSG00000031158/ ENSMUSG00000026500/ ENSMUSG00000067847/ ENSMUSG00000018411/ ENSMUSG00000049422/ ENSMUSG00000007033/ ENSMUSG00000074211/ ENSMUSG00000024500	18
GO:0010872	regulation of cholesterol esterification	3/350	11/21092	0.0007	0.0142	0.0124	ENSMUSG00000002985/ ENSMUSG00000032080/ ENSMUSG00000032083	3
GO:0034375	high−density lipoprotein particle remodeling	3/350	11/21092	0.0007	0.0142	0.0124	ENSMUSG00000018800/ ENSMUSG00000002985/ ENSMUSG00000032083	3
GO:1901293	nucleoside phosphate biosynthetic process	13/350	274/21092	0.0007	0.0147	0.0129	ENSMUSG00000034371/ ENSMUSG00000003809/ ENSMUSG00000020649/ ENSMUSG00000021236/ ENSMUSG00000050856/ ENSMUSG00000018796/ ENSMUSG00000006057/ ENSMUSG00000036813/ ENSMUSG00000060600/	13

							ENSMUSG00000073435/ ENSMUSG00000033610/ ENSMUSG00000028307/ ENSMUSG00000049422	
GO:0034446	substrate adhesion−dependent cell spreading	7/350	91/21092	0.0008	0.0164	0.0144	ENSMUSG00000022150/ ENSMUSG00000025810/ ENSMUSG00000034675/ ENSMUSG00000032702/ ENSMUSG00000032083/ ENSMUSG00000027750/ ENSMUSG00000049791	7
GO:1902600	proton transmembrane transport	7/350	91/21092	0.0008	0.0164	0.0144	ENSMUSG00000025453/ ENSMUSG00000116207/ ENSMUSG00000050856/ ENSMUSG00000006057/ ENSMUSG00000064370/ ENSMUSG00000064363/ ENSMUSG00000051177	7
GO:0009260	ribonucleotide biosynthetic process	11/350	211/21092	0.0008	0.0171	0.0150	ENSMUSG00000034371/ ENSMUSG00000003809/ ENSMUSG00000021236/ ENSMUSG00000050856/ ENSMUSG00000018796/ ENSMUSG00000006057/ ENSMUSG00000060600/ ENSMUSG00000073435/ ENSMUSG00000033610/ ENSMUSG00000028307/ ENSMUSG00000049422	11
GO:0032488	Cdc42 protein signal transduction	3/350	12/21092	0.0009	0.0180	0.0158	ENSMUSG00000002985/ ENSMUSG00000025810/ ENSMUSG00000032083	3
GO:1904478	regulation of intestinal absorption	3/350	12/21092	0.0009	0.0180	0.0158	ENSMUSG00000023832/ ENSMUSG00000032080/ ENSMUSG00000032083	3
GO:0010893	positive regulation of steroid biosynthetic process	4/350	27/21092	0.0010	0.0194	0.0170	ENSMUSG00000003585/ ENSMUSG00000022150/ ENSMUSG00000003555/ ENSMUSG00000036585	4
GO:0046165	alcohol biosynthetic process	8/350	122/21092	0.0010	0.0197	0.0173	ENSMUSG00000020609/ ENSMUSG00000027875/ ENSMUSG00000002985/ ENSMUSG00000032080/ ENSMUSG00000003585/ ENSMUSG00000022150/ ENSMUSG00000036585/ ENSMUSG00000032083	8
GO:0019915	lipid storage	6/350	69/21092	0.0010	0.0197	0.0173	ENSMUSG00000020609/ ENSMUSG00000002985/ ENSMUSG00000002944/ ENSMUSG00000011305/ ENSMUSG00000074336/ ENSMUSG00000032083	6
GO:0006094	gluconeogenesis	6/350	70/21092	0.0011	0.0211	0.0185	ENSMUSG00000002769/ ENSMUSG00000024892/ ENSMUSG00000062908/ ENSMUSG00000021456/ ENSMUSG00000029597/ ENSMUSG00000017950	6
GO:0046390	ribose phosphate biosynthetic process	11/350	218/21092	0.0011	0.0213	0.0187	ENSMUSG00000034371/ ENSMUSG00000003809/ ENSMUSG00000021236/ ENSMUSG00000050856/ ENSMUSG00000018796/ ENSMUSG00000006057/ ENSMUSG00000060600/ ENSMUSG00000073435/ ENSMUSG00000033610/ ENSMUSG00000028307/ ENSMUSG00000049422	11

GO:0002082	regulation of oxidative phosphorylation	4/350	28/21092	0.0011	0.0214	0.0188	ENSMUSG00000024248/ ENSMUSG00000074218/ ENSMUSG00000046329/ ENSMUSG00000032081	4
GO:0003413	chondrocyte differentiation involved in endochondral bone morphogenesis	4/350	28/21092	0.0011	0.0214	0.0188	ENSMUSG00000022324/ ENSMUSG00000025650/ ENSMUSG00000045672/ ENSMUSG00000006538	4
GO:0090181	regulation of cholesterol metabolic process	4/350	28/21092	0.0011	0.0214	0.0188	ENSMUSG00000020609/ ENSMUSG00000002985/ ENSMUSG00000003585/ ENSMUSG00000036585	4
GO:1902930	regulation of alcohol biosynthetic process	5/350	48/21092	0.0012	0.0222	0.0195	ENSMUSG00000020609/ ENSMUSG00000002985/ ENSMUSG00000003585/ ENSMUSG00000022150/ ENSMUSG00000036585	5
GO:0006140	regulation of nucleotide metabolic process	7/350	97/21092	0.0012	0.0222	0.0195	ENSMUSG00000021236/ ENSMUSG00000024248/ ENSMUSG00000074218/ ENSMUSG00000054428/ ENSMUSG00000046329/ ENSMUSG00000032081/ ENSMUSG00000028755	7
GO:0050810	regulation of steroid biosynthetic process	6/350	72/21092	0.0012	0.0235	0.0206	ENSMUSG00000020609/ ENSMUSG00000002985/ ENSMUSG00000003585/ ENSMUSG00000022150/ ENSMUSG00000003555/ ENSMUSG00000036585	6
GO:0006164	purine nucleotide biosynthetic process	11/350	222/21092	0.0013	0.0237	0.0208	ENSMUSG00000034371/ ENSMUSG00000003809/ ENSMUSG00000021236/ ENSMUSG00000050856/ ENSMUSG00000018796/ ENSMUSG00000006057/ ENSMUSG00000060600/ ENSMUSG00000073435/ ENSMUSG00000033610/ ENSMUSG00000028307/ ENSMUSG00000049422	11
GO:0046189	phenol−containing compound biosynthetic process	5/350	49/21092	0.0013	0.0238	0.0209	ENSMUSG00000024140/ ENSMUSG00000001666/ ENSMUSG00000038216/ ENSMUSG00000020182/ ENSMUSG00000084897	5
GO:0009206	purine ribonucleoside triphosphate biosynthetic process	8/350	127/21092	0.0013	0.0238	0.0209	ENSMUSG00000034371/ ENSMUSG00000021236/ ENSMUSG00000050856/ ENSMUSG00000006057/ ENSMUSG00000060600/ ENSMUSG00000073435/ ENSMUSG00000028307/ ENSMUSG00000049422	8
GO:0010035	response to inorganic substance	18/350	484/21092	0.0013	0.0240	0.0211	ENSMUSG00000031765/ ENSMUSG00000027227/ ENSMUSG00000002580/ ENSMUSG00000021794/ ENSMUSG00000032080/ ENSMUSG00000052572/ ENSMUSG00000004655/ ENSMUSG00000046329/ ENSMUSG00000002944/ ENSMUSG00000018411/ ENSMUSG00000027762/ ENSMUSG00000028307/	18

							ENSMUSG00000064370/ ENSMUSG00000076441/ ENSMUSG00000054422/ ENSMUSG00000019761/ ENSMUSG00000051177/ ENSMUSG00000023070	
GO:0009145	purine nucleoside triphosphate biosynthetic process	8/350	128/21092	0.0014	0.0248	0.0217	ENSMUSG00000034371/ ENSMUSG00000021236/ ENSMUSG00000050856/ ENSMUSG00000006057/ ENSMUSG00000060600/ ENSMUSG00000073435/ ENSMUSG00000028307/ ENSMUSG00000049422	8
GO:0006006	glucose metabolic process	10/350	192/21092	0.0014	0.0261	0.0229	ENSMUSG00000002769/ ENSMUSG00000035936/ ENSMUSG00000024892/ ENSMUSG00000062908/ ENSMUSG00000039450/ ENSMUSG00000021456/ ENSMUSG00000029597/ ENSMUSG00000059434/ ENSMUSG00000023070/ ENSMUSG00000017950	10
GO:0071941	nitrogen cycle metabolic process	3/350	14/21092	0.0014	0.0261	0.0229	ENSMUSG00000031173/ ENSMUSG00000049858/ ENSMUSG00000076441	3
GO:0006733	oxidoreduction coenzyme metabolic process	9/350	160/21092	0.0014	0.0261	0.0229	ENSMUSG00000034371/ ENSMUSG00000021236/ ENSMUSG00000025453/ ENSMUSG00000116207/ ENSMUSG00000026489/ ENSMUSG00000039450/ ENSMUSG00000060600/ ENSMUSG00000005951/ ENSMUSG00000028307	9
GO:0006633	fatty acid biosynthetic process	8/350	130/21092	0.0015	0.0266	0.0234	ENSMUSG00000021620/ ENSMUSG00000041220/ ENSMUSG00000025203/ ENSMUSG00000032080/ ENSMUSG00000002992/ ENSMUSG00000023070/ ENSMUSG00000017950/ ENSMUSG00000017146	8
GO:0009201	ribonucleoside triphosphate biosynthetic process	8/350	130/21092	0.0015	0.0266	0.0234	ENSMUSG00000034371/ ENSMUSG00000021236/ ENSMUSG00000050856/ ENSMUSG00000006057/ ENSMUSG00000060600/ ENSMUSG00000073435/ ENSMUSG00000028307/ ENSMUSG00000049422	8
GO:0072522	purine−containing compound biosynthetic process	11/350	227/21092	0.0015	0.0268	0.0235	ENSMUSG00000034371/ ENSMUSG00000003809/ ENSMUSG00000021236/ ENSMUSG00000050856/ ENSMUSG00000018796/ ENSMUSG00000006057/ ENSMUSG00000060600/ ENSMUSG00000073435/ ENSMUSG00000033610/ ENSMUSG00000028307/ ENSMUSG00000049422	11
GO:0051188	cofactor biosynthetic process	11/350	228/21092	0.0016	0.0273	0.0240	ENSMUSG00000034371/ ENSMUSG00000003809/ ENSMUSG00000021236/ ENSMUSG00000018796/ ENSMUSG00000026489/ ENSMUSG00000054428/ ENSMUSG00000060600/ ENSMUSG00000026198/ ENSMUSG00000033610/ ENSMUSG00000028307/ ENSMUSG00000023070	11

GO:0006090	pyruvate metabolic process	7/350	102/21092	0.0016	0.0273	0.0240	ENSMUSG00000034371/ ENSMUSG00000021236/ ENSMUSG00000039648/ ENSMUSG00000024892/ ENSMUSG00000060600/ ENSMUSG00000028307/ ENSMUSG00000029597	7
GO:1901568	fatty acid derivative metabolic process	8/350	131/21092	0.0016	0.0273	0.0240	ENSMUSG00000024507/ ENSMUSG00000025002/ ENSMUSG00000003809/ ENSMUSG00000018796/ ENSMUSG00000094806/ ENSMUSG00000022445/ ENSMUSG00000062624/ ENSMUSG00000067231	8
GO:0009165	nucleotide biosynthetic process	12/350	267/21092	0.0018	0.0302	0.0265	ENSMUSG00000034371/ ENSMUSG00000003809/ ENSMUSG00000020649/ ENSMUSG00000021236/ ENSMUSG00000050856/ ENSMUSG00000018796/ ENSMUSG00000006057/ ENSMUSG00000060600/ ENSMUSG00000073435/ ENSMUSG00000033610/ ENSMUSG00000028307/ ENSMUSG00000049422	12
GO:0009084	glutamine family amino acid biosynthetic process	3/350	15/21092	0.0018	0.0302	0.0265	ENSMUSG00000031173/ ENSMUSG00000021794/ ENSMUSG00000076441	3
GO:0016114	terpenoid biosynthetic process	3/350	15/21092	0.0018	0.0302	0.0265	ENSMUSG00000046402/ ENSMUSG00000027875/ ENSMUSG00000056148	3
GO:0035337	fatty−acyl−CoA metabolic process	3/350	15/21092	0.0018	0.0302	0.0265	ENSMUSG00000024507/ ENSMUSG00000003809/ ENSMUSG00000018796	3
GO:1990000	amyloid fibril formation	3/350	15/21092	0.0018	0.0302	0.0265	ENSMUSG00000002985/ ENSMUSG00000002944/ ENSMUSG00000018411	3
GO:0006084	acetyl−CoA metabolic process	4/350	32/21092	0.0019	0.0314	0.0275	ENSMUSG00000021620/ ENSMUSG00000027875/ ENSMUSG00000036880/ ENSMUSG00000017453	4
GO:0097164	ammonium ion metabolic process	9/350	166/21092	0.0019	0.0314	0.0275	ENSMUSG00000024140/ ENSMUSG00000026385/ ENSMUSG00000027333/ ENSMUSG00000062908/ ENSMUSG00000032080/ ENSMUSG00000026853/ ENSMUSG00000028743/ ENSMUSG00000032083/ ENSMUSG00000020182	9
GO:1903578	regulation of ATP metabolic process	6/350	78/21092	0.0019	0.0314	0.0276	ENSMUSG00000021236/ ENSMUSG00000024248/ ENSMUSG00000074218/ ENSMUSG00000054428/ ENSMUSG00000046329/ ENSMUSG00000032081	6
GO:0010038	response to metal ion	12/350	269/21092	0.0019	0.0314	0.0276	ENSMUSG00000031765/ ENSMUSG00000027227/ ENSMUSG00000021794/ ENSMUSG00000052572/ ENSMUSG00000004655/ ENSMUSG00000046329/ ENSMUSG00000018411/ ENSMUSG00000027762/ ENSMUSG00000028307/ ENSMUSG00000064370/ ENSMUSG00000076441/ ENSMUSG00000019761	12

GO:0022600	digestive system process	7/350	106/21092	0.0020	0.0324	0.0284	ENSMUSG00000023832/ ENSMUSG00000032080/ ENSMUSG00000004655/ ENSMUSG00000002944/ ENSMUSG00000112129/ ENSMUSG00000054422/ ENSMUSG00000032083	7
GO:0019682	glyceraldehyde−3−phosphate metabolic process	3/350	16/21092	0.0022	0.0356	0.0313	ENSMUSG00000034371/ ENSMUSG00000005951/ ENSMUSG00000028307	3
GO:0015748	organophosphate ester transport	6/350	81/21092	0.0023	0.0375	0.0329	ENSMUSG00000002985/ ENSMUSG00000032080/ ENSMUSG00000028158/ ENSMUSG00000046329/ ENSMUSG00000002992/ ENSMUSG00000032083	6
GO:0019369	arachidonic acid metabolic process	5/350	56/21092	0.0023	0.0381	0.0334	ENSMUSG00000025002/ ENSMUSG00000094806/ ENSMUSG00000022445/ ENSMUSG00000062624/ ENSMUSG00000067231	5
GO:0009142	nucleoside triphosphate biosynthetic process	8/350	141/21092	0.0025	0.0404	0.0354	ENSMUSG00000034371/ ENSMUSG00000021236/ ENSMUSG00000050856/ ENSMUSG00000006057/ ENSMUSG00000060600/ ENSMUSG00000073435/ ENSMUSG00000028307/ ENSMUSG00000049422	8
GO:0010810	regulation of cell−substrate adhesion	10/350	208/21092	0.0026	0.0412	0.0362	ENSMUSG00000040998/ ENSMUSG00000002944/ ENSMUSG00000022150/ ENSMUSG00000025810/ ENSMUSG00000034675/ ENSMUSG00000032702/ ENSMUSG00000044433/ ENSMUSG00000032083/ ENSMUSG00000027750/ ENSMUSG00000049791	10
GO:0010867	positive regulation of triglyceride biosynthetic process	3/350	17/21092	0.0026	0.0412	0.0362	ENSMUSG00000032081/ ENSMUSG00000011305/ ENSMUSG00000023070	3
GO:0032780	negative regulation of ATPase activity	3/350	17/21092	0.0026	0.0412	0.0362	ENSMUSG00000054428/ ENSMUSG00000034675/ ENSMUSG00000023070	3
GO:0046496	nicotinamide nucleotide metabolic process	8/350	142/21092	0.0026	0.0412	0.0362	ENSMUSG00000034371/ ENSMUSG00000021236/ ENSMUSG00000025453/ ENSMUSG00000116207/ ENSMUSG00000039450/ ENSMUSG00000060600/ ENSMUSG00000005951/ ENSMUSG00000028307	8
GO:0019432	triglyceride biosynthetic process	4/350	35/21092	0.0026	0.0412	0.0362	ENSMUSG00000018796/ ENSMUSG00000032081/ ENSMUSG00000011305/ ENSMUSG00000023070	4
GO:0032368	regulation of lipid transport	7/350	112/21092	0.0027	0.0421	0.0369	ENSMUSG00000023832/ ENSMUSG00000002985/ ENSMUSG00000032080/ ENSMUSG00000040249/ ENSMUSG00000022150/ ENSMUSG00000002992/ ENSMUSG00000032083	7

GO:0009108	coenzyme biosynthetic process	9/350	176/21092	0.0028	0.0435	0.0382	ENSMUSG00000034371/ ENSMUSG00000003809/ ENSMUSG00000021236/ ENSMUSG00000018796/ ENSMUSG00000026489/ ENSMUSG00000060600/ ENSMUSG00000033610/ ENSMUSG00000028307/ ENSMUSG00000023070	9
GO:0019362	pyridine nucleotide metabolic process	8/350	144/21092	0.0028	0.0442	0.0388	ENSMUSG00000034371/ ENSMUSG00000021236/ ENSMUSG00000025453/ ENSMUSG00000116207/ ENSMUSG00000039450/ ENSMUSG00000060600/ ENSMUSG00000005951/ ENSMUSG00000028307	8
GO:0010770	positive regulation of cell morphogenesis involved in differentiation	9/350	177/21092	0.0029	0.0445	0.0390	ENSMUSG00000032526/ ENSMUSG00000001313/ ENSMUSG00000002985/ ENSMUSG00000018411/ ENSMUSG00000022150/ ENSMUSG00000025810/ ENSMUSG00000034675/ ENSMUSG00000002274/ ENSMUSG00000032083	9
GO:0006739	NADP metabolic process	4/350	36/21092	0.0029	0.0445	0.0390	ENSMUSG00000025453/ ENSMUSG00000116207/ ENSMUSG00000039450/ ENSMUSG00000005951	4
GO:1900026	positive regulation of substrate adhesion−dependent cell spreading	4/350	36/21092	0.0029	0.0445	0.0390	ENSMUSG00000022150/ ENSMUSG00000025810/ ENSMUSG00000034675/ ENSMUSG00000032083	4
GO:0045833	negative regulation of lipid metabolic process	6/350	85/21092	0.0029	0.0446	0.0392	ENSMUSG00000026385/ ENSMUSG00000002985/ ENSMUSG00000032081/ ENSMUSG00000002992/ ENSMUSG00000011305/ ENSMUSG00000017146	6
GO:0030952	establishment or maintenance of cytoskeleton polarity	3/350	18/21092	0.0031	0.0466	0.0409	ENSMUSG00000001313/ ENSMUSG00000004655/ ENSMUSG00000044433	3
GO:0042219	cellular modified amino acid catabolic process	3/350	18/21092	0.0031	0.0466	0.0409	ENSMUSG00000041625/ ENSMUSG00000009614/ ENSMUSG00000024579	3
GO:0044764	multi−organism cellular process	4/350	37/21092	0.0032	0.0483	0.0424	ENSMUSG00000034371/ ENSMUSG00000029368/ ENSMUSG00000067847/ ENSMUSG00000010047	4
**GO terms significantly enriched in upregulated genes**								
**ID**	**Description**	**GeneRatio**	**BgRatio**	**pvalue**	**p.adjust**	**qvalue**	**geneID**	**Co**
GO:0051607	defense response to virus	69/815	226/21092	9.64×10^{-43}	4.58×10^{-39}	3.98×10^{-39}	ENSMUSG00000032661/ ENSMUSG00000023341/ ENSMUSG00000025498/ ENSMUSG00000026104/ ENSMUSG00000025492/ ENSMUSG00000017830/ ENSMUSG00000026896/ ENSMUSG00000049502/ ENSMUSG00000046718/ ENSMUSG00000022906/ ENSMUSG00000056144/ ENSMUSG00000040033/	69

							ENSMUSG00000027639/ ENSMUSG00000040296/ ENSMUSG00000024079/ ENSMUSG00000029605/ ENSMUSG00000027951/ ENSMUSG00000033355/ ENSMUSG00000029780/ ENSMUSG00000040613/ ENSMUSG00000036986/ ENSMUSG00000029826/ ENSMUSG00000048806/ ENSMUSG00000000275/ ENSMUSG00000040987/ ENSMUSG00000036908/ ENSMUSG00000070904/ ENSMUSG00000038884/ ENSMUSG00000031639/ ENSMUSG00000072244/ ENSMUSG00000035151/ ENSMUSG00000039236/ ENSMUSG00000009585/ ENSMUSG00000066800/ ENSMUSG00000045932/ ENSMUSG00000000791/ ENSMUSG00000060550/ ENSMUSG00000043279/ ENSMUSG00000031627/ ENSMUSG00000037860/ ENSMUSG00000037921/ ENSMUSG00000069793/ ENSMUSG00000067297/ ENSMUSG00000020115/ ENSMUSG00000055204/ ENSMUSG00000001166/ ENSMUSG00000035692/ ENSMUSG00000034459/ ENSMUSG00000035042/ ENSMUSG00000032690/ ENSMUSG00000027514/ ENSMUSG00000079339/ ENSMUSG00000074896/ ENSMUSG00000041827/ ENSMUSG00000066861/ ENSMUSG00000029561/ ENSMUSG00000052776/ ENSMUSG00000000386/ ENSMUSG00000062488/ ENSMUSG00000030921/ ENSMUSG00000040264/ ENSMUSG00000079363/ ENSMUSG00000034855/ ENSMUSG00000020641/ ENSMUSG00000029417/ ENSMUSG00000024810/ ENSMUSG00000018899/ ENSMUSG00000031712/ ENSMUSG00000025746	
GO:0009615	response to virus	74/815	270/21092	3.84×10^{-42}	9.13×10^{-39}	7.93×10^{-39}	ENSMUSG00000032661/ ENSMUSG00000023341/ ENSMUSG00000025498/ ENSMUSG00000026104/ ENSMUSG00000025492/ ENSMUSG00000017830/ ENSMUSG00000069874/ ENSMUSG00000026896/ ENSMUSG00000049502/ ENSMUSG00000046718/ ENSMUSG00000022906/ ENSMUSG00000056144/ ENSMUSG00000040033/ ENSMUSG00000027639/ ENSMUSG00000040296/ ENSMUSG00000024079/ ENSMUSG00000029605/ ENSMUSG00000027951/ ENSMUSG00000033355/ ENSMUSG00000029780/ ENSMUSG00000040613/ ENSMUSG00000036986/ ENSMUSG00000029826/ ENSMUSG00000048806/	74

							ENSMUSG00000000275/ ENSMUSG00000001123/ ENSMUSG00000040987/ ENSMUSG00000036908/ ENSMUSG00000070904/ ENSMUSG00000038884/ ENSMUSG00000031639/ ENSMUSG00000057554/ ENSMUSG00000072244/ ENSMUSG00000035151/ ENSMUSG00000039236/ ENSMUSG00000009585/ ENSMUSG00000066800/ ENSMUSG00000045932/ ENSMUSG00000000791/ ENSMUSG00000060550/ ENSMUSG00000043279/ ENSMUSG00000032508/ ENSMUSG00000031627/ ENSMUSG00000037860/ ENSMUSG00000037921/ ENSMUSG00000069793/ ENSMUSG00000067297/ ENSMUSG00000020115/ ENSMUSG00000055204/ ENSMUSG00000001166/ ENSMUSG00000035692/ ENSMUSG00000034459/ ENSMUSG00000035042/ ENSMUSG00000032690/ ENSMUSG00000000838/ ENSMUSG00000027514/ ENSMUSG00000079339/ ENSMUSG00000074896/ ENSMUSG00000041827/ ENSMUSG00000066861/ ENSMUSG00000029561/ ENSMUSG00000052776/ ENSMUSG00000000386/ ENSMUSG00000062488/ ENSMUSG00000030921/ ENSMUSG00000040264/ ENSMUSG00000079363/ ENSMUSG00000034855/ ENSMUSG00000020641/ ENSMUSG00000029417/ ENSMUSG00000024810/ ENSMUSG00000018899/ ENSMUSG00000031712/ ENSMUSG00000025746	
GO:0035456	response to interferon−beta	37/815	63/21092	3.26×10^{-36}	5.16×10^{-33}	4.49×10^{-33}	ENSMUSG00000048852/ ENSMUSG00000078922/ ENSMUSG00000026104/ ENSMUSG00000025492/ ENSMUSG00000026535/ ENSMUSG00000069874/ ENSMUSG00000046718/ ENSMUSG00000058163/ ENSMUSG00000078853/ ENSMUSG00000020464/ ENSMUSG00000048806/ ENSMUSG00000040483/ ENSMUSG00000078921/ ENSMUSG00000069893/ ENSMUSG00000072244/ ENSMUSG00000049734/ ENSMUSG00000037860/ ENSMUSG00000073489/ ENSMUSG00000054203/ ENSMUSG00000043263/ ENSMUSG00000046879/ ENSMUSG00000067297/ ENSMUSG00000028270/ ENSMUSG00000073555/ ENSMUSG00000034459/ ENSMUSG00000039997/ ENSMUSG00000028268/ ENSMUSG00000054072/ ENSMUSG00000079339/ ENSMUSG00000074896/	37

							ENSMUSG00001104713/ ENSMUSG00000062488/ ENSMUSG00000068606/ ENSMUSG00000040264/ ENSMUSG00000090942/ ENSMUSG00000022126/ ENSMUSG00000018899	
GO:0035458	cellular response to interferon−beta	34/815	53/21092	2.76 × 10^{-35}	3.28 × 10^{-32}	2.85 × 10^{-32}	ENSMUSG00000048852/ ENSMUSG00000078922/ ENSMUSG00000026104/ ENSMUSG00000026535/ ENSMUSG00000069874/ ENSMUSG00000058163/ ENSMUSG00000078853/ ENSMUSG00000020464/ ENSMUSG00000048806/ ENSMUSG00000078921/ ENSMUSG00000069893/ ENSMUSG00000072244/ ENSMUSG00000049734/ ENSMUSG00000037860/ ENSMUSG00000073489/ ENSMUSG00000054203/ ENSMUSG00000043263/ ENSMUSG00000046879/ ENSMUSG00000067297/ ENSMUSG00000028270/ ENSMUSG00000073555/ ENSMUSG00000034459/ ENSMUSG00000039997/ ENSMUSG00000028268/ ENSMUSG00000054072/ ENSMUSG00000079339/ ENSMUSG00000074896/ ENSMUSG00001104713/ ENSMUSG00000062488/ ENSMUSG00000068606/ ENSMUSG00000040264/ ENSMUSG00000090942/ ENSMUSG00000022126/ ENSMUSG00000018899	34
GO:0034341	response to interferon−gamma	39/815	139/21092	4.18 × 10^{-23}	3.97 × 10^{-20}	3.45 × 10^{-20}	ENSMUSG00000078922/ ENSMUSG00000026104/ ENSMUSG00000025492/ ENSMUSG00000069874/ ENSMUSG00000034422/ ENSMUSG00000046718/ ENSMUSG00000022906/ ENSMUSG00000030966/ ENSMUSG00000026946/ ENSMUSG00000038884/ ENSMUSG00000000791/ ENSMUSG00000060550/ ENSMUSG00000037580/ ENSMUSG00000038037/ ENSMUSG00000074151/ ENSMUSG00000046879/ ENSMUSG00000028270/ ENSMUSG00000040253/ ENSMUSG00000105096/ ENSMUSG00000035042/ ENSMUSG00000028268/ ENSMUSG00000029860/ ENSMUSG00000034438/ ENSMUSG00001104713/ ENSMUSG00000029298/ ENSMUSG00000025888/ ENSMUSG00000040264/ ENSMUSG00000079363/ ENSMUSG00000026797/ ENSMUSG00000105504/ ENSMUSG00000035373/ ENSMUSG00000022126/ ENSMUSG00000035186/	39

							ENSMUSG00000018899/ ENSMUSG00000027995/ ENSMUSG00000030895/ ENSMUSG00000024789/ ENSMUSG00000035385/ ENSMUSG00000020826	
GO:0043900	regulation of multi−organism process	61/815	394/21092	1.30 × $10^{−20}$	1.03 × $10^{−17}$	8.92 × $10^{−18}$	ENSMUSG00000032661/ ENSMUSG00000026104/ ENSMUSG00000025492/ ENSMUSG00000017830/ ENSMUSG00000049502/ ENSMUSG00000046718/ ENSMUSG00000022906/ ENSMUSG00000040296/ ENSMUSG00000024079/ ENSMUSG00000029605/ ENSMUSG00000030966/ ENSMUSG00000027951/ ENSMUSG00000063268/ ENSMUSG00000036986/ ENSMUSG00000029826/ ENSMUSG00000048806/ ENSMUSG00000000275/ ENSMUSG00000001123/ ENSMUSG00000040987/ ENSMUSG00000024457/ ENSMUSG00000038884/ ENSMUSG00000072244/ ENSMUSG00000035151/ ENSMUSG00000039236/ ENSMUSG00000009585/ ENSMUSG00000066800/ ENSMUSG00000039853/ ENSMUSG00000000791/ ENSMUSG00000021795/ ENSMUSG00000026672/ ENSMUSG00000043279/ ENSMUSG00000032508/ ENSMUSG00000039217/ ENSMUSG00000037860/ ENSMUSG00000037921/ ENSMUSG00000038467/ ENSMUSG00000037331/ ENSMUSG00000020115/ ENSMUSG00000055204/ ENSMUSG00000001166/ ENSMUSG00000031813/ ENSMUSG00000052684/ ENSMUSG00000038058/ ENSMUSG00000032501/ ENSMUSG00000035692/ ENSMUSG00000035042/ ENSMUSG00000000838/ ENSMUSG00000041827/ ENSMUSG00000066861/ ENSMUSG00000029561/ ENSMUSG00000052776/ ENSMUSG00000030921/ ENSMUSG00000079363/ ENSMUSG00000020641/ ENSMUSG00000025779/ ENSMUSG00000034610/ ENSMUSG00000022126/ ENSMUSG00000027995/ ENSMUSG00000031712/ ENSMUSG00000062300/ ENSMUSG00000020826	61
GO:0002697	regulation of immune effector process	61/815	399/21092	2.49 × $10^{−20}$	1.69 × $10^{−17}$	1.47 × $10^{−17}$	ENSMUSG00000026104/ ENSMUSG00000017830/ ENSMUSG00000049502/ ENSMUSG00000037321/ ENSMUSG00000024339/ ENSMUSG00000022906/ ENSMUSG00000035929/ ENSMUSG00000061232/ ENSMUSG00000073411/ ENSMUSG00000040296/ ENSMUSG00000115338/ ENSMUSG00000036986/	61

							ENSMUSG00000060802/ ENSMUSG00000029826/ ENSMUSG00000048806/ ENSMUSG00000092243/ ENSMUSG00000001123/ ENSMUSG00000067212/ ENSMUSG00000040987/ ENSMUSG00000079491/ ENSMUSG00000056116/ ENSMUSG00000016206/ ENSMUSG00000073402/ ENSMUSG00000031639/ ENSMUSG00000072244/ ENSMUSG00000035151/ ENSMUSG00000009585/ ENSMUSG00000030157/ ENSMUSG00000000791/ ENSMUSG00000079507/ ENSMUSG00000023224/ ENSMUSG00000060550/ ENSMUSG00000000127/ ENSMUSG00000037447/ ENSMUSG00000039217/ ENSMUSG00000037860/ ENSMUSG00000073409/ ENSMUSG00000037921/ ENSMUSG00000004508/ ENSMUSG00000045827/ ENSMUSG00000055204/ ENSMUSG00000042333/ ENSMUSG00000053835/ ENSMUSG00000031948/ ENSMUSG00000022378/ ENSMUSG00000035042/ ENSMUSG00000079363/ ENSMUSG00000020641/ ENSMUSG00000026797/ ENSMUSG00000026365/ ENSMUSG00000067235/ ENSMUSG00000024810/ ENSMUSG00000018899/ ENSMUSG00000027995/ ENSMUSG00000069255/ ENSMUSG00000031712/ ENSMUSG00000008734/ ENSMUSG00000030895/ ENSMUSG00000035385/ ENSMUSG00000062300/ ENSMUSG00000025746	
GO:0048525	negative regulation of viral process	30/815	92/21092	3.71×10^{-20}	2.21×10^{-17}	1.92×10^{-17}	ENSMUSG00000032661/ ENSMUSG00000026104/ ENSMUSG00000025492/ ENSMUSG00000046718/ ENSMUSG00000024079/ ENSMUSG00000029605/ ENSMUSG00000030966/ ENSMUSG00000027951/ ENSMUSG00000063268/ ENSMUSG00000036986/ ENSMUSG00000029826/ ENSMUSG00000048806/ ENSMUSG00000000275/ ENSMUSG00000024457/ ENSMUSG00000038884/ ENSMUSG00000072244/ ENSMUSG00000039236/ ENSMUSG00000009585/ ENSMUSG00000066800/ ENSMUSG00000039853/ ENSMUSG00000043279/ ENSMUSG00000001166/ ENSMUSG00000052684/ ENSMUSG00000035692/ ENSMUSG00000035042/ ENSMUSG00000041827/ ENSMUSG00000066861/ ENSMUSG00000029561/ ENSMUSG00000052776/ ENSMUSG00000020641	30

GO:1903901	negative regulation of viral life cycle	27/815	77/21092	3.25×10^{-19}	1.72×10^{-16}	1.49×10^{-16}	ENSMUSG00000032661/ ENSMUSG00000025492/ ENSMUSG00000046718/ ENSMUSG00000024079/ ENSMUSG00000029605/ ENSMUSG00000030966/ ENSMUSG00000027951/ ENSMUSG00000063268/ ENSMUSG00000036986/ ENSMUSG00000029826/ ENSMUSG00000048806/ ENSMUSG00000000275/ ENSMUSG00000024457/ ENSMUSG00000038884/ ENSMUSG00000072244/ ENSMUSG00000039236/ ENSMUSG00000009585/ ENSMUSG00000066800/ ENSMUSG00000043279/ ENSMUSG00000001166/ ENSMUSG00000035692/ ENSMUSG00000035042/ ENSMUSG00000041827/ ENSMUSG00000066861/ ENSMUSG00000029561/ ENSMUSG00000052776/ ENSMUSG00000020641	27
GO:0045088	regulation of innate immune response	47/815	258/21092	4.73×10^{-19}	2.25×10^{-16}	1.95×10^{-16}	ENSMUSG00000025498/ ENSMUSG00000017830/ ENSMUSG00000069874/ ENSMUSG00000026896/ ENSMUSG00000034422/ ENSMUSG00000037321/ ENSMUSG00000024339/ ENSMUSG00000022906/ ENSMUSG00000027639/ ENSMUSG00000042726/ ENSMUSG00000040296/ ENSMUSG00000026946/ ENSMUSG00000027951/ ENSMUSG00000029826/ ENSMUSG00000020707/ ENSMUSG00000001123/ ENSMUSG00000067212/ ENSMUSG00000040987/ ENSMUSG00000036908/ ENSMUSG00000016206/ ENSMUSG00000031639/ ENSMUSG00000072244/ ENSMUSG00000030157/ ENSMUSG00000045038/ ENSMUSG00000023224/ ENSMUSG00000038037/ ENSMUSG00000073643/ ENSMUSG00000032508/ ENSMUSG00000037860/ ENSMUSG00000037921/ ENSMUSG00000074151/ ENSMUSG00000046879/ ENSMUSG00000045827/ ENSMUSG00000020115/ ENSMUSG00000055204/ ENSMUSG00000027514/ ENSMUSG00000030921/ ENSMUSG00000070056/ ENSMUSG00000020641/ ENSMUSG00000025779/ ENSMUSG00000105504/ ENSMUSG00000022126/ ENSMUSG00000018899/ ENSMUSG00000028028/ ENSMUSG00000027995/ ENSMUSG00000030895/ ENSMUSG00000062300	47
GO:0071346	cellular response to interferon−gamma	32/815	117/21092	7.95×10^{-19}	3.44×10^{-16}	2.99×10^{-16}	ENSMUSG00000026104/ ENSMUSG00000069874/ ENSMUSG00000034422/ ENSMUSG00000022906/ ENSMUSG00000026946/ ENSMUSG00000000791/	32

							ENSMUSG00000060550/ ENSMUSG00000038037/ ENSMUSG00000074151/ ENSMUSG00000046879/ ENSMUSG00000028270/ ENSMUSG00000040253/ ENSMUSG00000105096/ ENSMUSG00000035042/ ENSMUSG00000028268/ ENSMUSG00000029860/ ENSMUSG00000034438/ ENSMUSG00000104713/ ENSMUSG00000029298/ ENSMUSG00000025888/ ENSMUSG00000040264/ ENSMUSG00000079363/ ENSMUSG00000026797/ ENSMUSG00000105504/ ENSMUSG00000035373/ ENSMUSG00000022126/ ENSMUSG00000018899/ ENSMUSG00000027995/ ENSMUSG00000030895/ ENSMUSG00000024789/ ENSMUSG00000035385/ ENSMUSG00000020826	
GO:0002483	antigen processing and presentation of endogenous peptide antigen	19/815	35/21092	2.63×10^{-18}	1.04×10^{-15}	9.06×10^{-16}	ENSMUSG00000037321/ ENSMUSG00000024339/ ENSMUSG00000035929/ ENSMUSG00000061232/ ENSMUSG00000073411/ ENSMUSG00000024308/ ENSMUSG00000060802/ ENSMUSG00000092243/ ENSMUSG00000067212/ ENSMUSG00000079491/ ENSMUSG00000056116/ ENSMUSG00000016206/ ENSMUSG00000073402/ ENSMUSG00000021583/ ENSMUSG00000079507/ ENSMUSG00000060550/ ENSMUSG00000073409/ ENSMUSG00000053835/ ENSMUSG00000067235	19
GO:0045071	negative regulation of viral genome replication	22/815	52/21092	5.63×10^{-18}	2.06×10^{-15}	1.79×10^{-15}	ENSMUSG00000032661/ ENSMUSG00000025492/ ENSMUSG00000046718/ ENSMUSG00000024079/ ENSMUSG00000029605/ ENSMUSG00000027951/ ENSMUSG00000063268/ ENSMUSG00000029826/ ENSMUSG00000048806/ ENSMUSG00000038884/ ENSMUSG00000072244/ ENSMUSG00000039236/ ENSMUSG00000009585/ ENSMUSG00000066800/ ENSMUSG00000001166/ ENSMUSG00000035692/ ENSMUSG00000035042/ ENSMUSG00000041827/ ENSMUSG00000066861/ ENSMUSG00000029561/ ENSMUSG00000052776/ ENSMUSG00000020641	22
GO:0044403	symbiont process	53/815	352/21092	1.63×10^{-17}	5.54×10^{-15}	4.81×10^{-15}	ENSMUSG00000032661/ ENSMUSG00000026104/ ENSMUSG00000025492/ ENSMUSG00000069874/ ENSMUSG00000046718/ ENSMUSG00000024079/ ENSMUSG00000029605/ ENSMUSG00000030966/ ENSMUSG00000027951/	53

							ENSMUSG00000063268/ ENSMUSG00000036986/ ENSMUSG00000029826/ ENSMUSG00000048806/ ENSMUSG00000000275/ ENSMUSG00000024457/ ENSMUSG00000038884/ ENSMUSG00000072244/ ENSMUSG00000039236/ ENSMUSG00000009585/ ENSMUSG00000066800/ ENSMUSG00000039853/ ENSMUSG00000002602/ ENSMUSG00000021795/ ENSMUSG00000043279/ ENSMUSG00000032508/ ENSMUSG00000022346/ ENSMUSG00000046879/ ENSMUSG00000038467/ ENSMUSG00000037331/ ENSMUSG00000045827/ ENSMUSG00000001166/ ENSMUSG00000031813/ ENSMUSG00000052684/ ENSMUSG00000035354/ ENSMUSG00000028270/ ENSMUSG00000040253/ ENSMUSG00000035692/ ENSMUSG00000035042/ ENSMUSG00000000838/ ENSMUSG00000027514/ ENSMUSG00000028268/ ENSMUSG00000041827/ ENSMUSG00000066861/ ENSMUSG00000029561/ ENSMUSG00000052776/ ENSMUSG00000104713/ ENSMUSG00000029298/ ENSMUSG00000030921/ ENSMUSG00000026433/ ENSMUSG00000040264/ ENSMUSG00000020641/ ENSMUSG00000027995/ ENSMUSG00000062300	
GO:0019883	antigen processing and presentation of endogenous antigen	19/815	38/21092	2.05×10^{-17}	6.51×10^{-15}	5.65×10^{-15}	ENSMUSG00000037321/ ENSMUSG00000024339/ ENSMUSG00000035929/ ENSMUSG00000061232/ ENSMUSG00000073411/ ENSMUSG00000024308/ ENSMUSG00000060802/ ENSMUSG00000092243/ ENSMUSG00000067212/ ENSMUSG00000079491/ ENSMUSG00000056116/ ENSMUSG00000016206/ ENSMUSG00000073402/ ENSMUSG00000021583/ ENSMUSG00000079507/ ENSMUSG00000060550/ ENSMUSG00000073409/ ENSMUSG00000053835/ ENSMUSG00000067235	19
GO:0002460	adaptive immune response based on somatic recombination of immune receptors built from immunoglobulin superfamily domains	48/815	296/21092	2.72×10^{-17}	8.09×10^{-15}	7.03×10^{-15}	ENSMUSG00000025498/ ENSMUSG00000024339/ ENSMUSG00000035929/ ENSMUSG00000061232/ ENSMUSG00000073411/ ENSMUSG00000115338/ ENSMUSG00000060802/ ENSMUSG00000024371/ ENSMUSG00000048806/ ENSMUSG00000092243/ ENSMUSG00000067212/ ENSMUSG00000040987/ ENSMUSG00000079491/ ENSMUSG00000056116/ ENSMUSG00000016206/	48

							ENSMUSG00000073402/ ENSMUSG00000000791/ ENSMUSG00000079507/ ENSMUSG00000021403/ ENSMUSG00000023224/ ENSMUSG00000060550/ ENSMUSG00000022505/ ENSMUSG00000037447/ ENSMUSG00000032508/ ENSMUSG00000039217/ ENSMUSG00000073409/ ENSMUSG00000070427/ ENSMUSG00000031154/ ENSMUSG00000055172/ ENSMUSG00000079343/ ENSMUSG00000045827/ ENSMUSG00000004040/ ENSMUSG00000053835/ ENSMUSG00000026405/ ENSMUSG00000029869/ ENSMUSG00000073418/ ENSMUSG00000016496/ ENSMUSG00000022378/ ENSMUSG00000021453/ ENSMUSG00000020641/ ENSMUSG00000067235/ ENSMUSG00000024810/ ENSMUSG00000018899/ ENSMUSG00000038521/ ENSMUSG00000069255/ ENSMUSG00000030895/ ENSMUSG00000062300/ ENSMUSG00000025746	
GO:1903900	regulation of viral life cycle	33/815	140/21092	3.11×10^{-17}	8.71×10^{-15}	7.56×10^{-15}	ENSMUSG00000032661/ ENSMUSG00000025492/ ENSMUSG00000046718/ ENSMUSG00000024079/ ENSMUSG00000029605/ ENSMUSG00000030966/ ENSMUSG00000027951/ ENSMUSG00000063268/ ENSMUSG00000036986/ ENSMUSG00000029826/ ENSMUSG00000048806/ ENSMUSG00000000275/ ENSMUSG00000024457/ ENSMUSG00000038884/ ENSMUSG00000072244/ ENSMUSG00000039236/ ENSMUSG00000009585/ ENSMUSG00000066800/ ENSMUSG00000043279/ ENSMUSG00000038467/ ENSMUSG00000037331/ ENSMUSG00000001166/ ENSMUSG00000031813/ ENSMUSG00000035692/ ENSMUSG00000035042/ ENSMUSG00000000838/ ENSMUSG00000041827/ ENSMUSG00000066861/ ENSMUSG00000029561/ ENSMUSG00000052776/ ENSMUSG00000030921/ ENSMUSG00000020641/ ENSMUSG00000062300	33
GO:0044419	interspecies interaction between organisms	56/815	401/21092	6.31×10^{-17}	1.67×10^{-14}	1.45×10^{-14}	ENSMUSG00000032661/ ENSMUSG00000026104/ ENSMUSG00000025492/ ENSMUSG00000069874/ ENSMUSG00000046718/ ENSMUSG00000024079/ ENSMUSG00000029605/ ENSMUSG00000030966/ ENSMUSG00000027951/ ENSMUSG00000063268/ ENSMUSG00000036986/ ENSMUSG00000029826/ ENSMUSG00000048806/ ENSMUSG00000000275/ ENSMUSG00000024457/	56

							ENSMUSG00000038884/ ENSMUSG00000072244/ ENSMUSG00000039236/ ENSMUSG00000009585/ ENSMUSG00000066800/ ENSMUSG00000039853/ ENSMUSG00000002602/ ENSMUSG00000021795/ ENSMUSG00000043279/ ENSMUSG00000032508/ ENSMUSG00000022346/ ENSMUSG00000022575/ ENSMUSG00000046879/ ENSMUSG00000038467/ ENSMUSG00000037331/ ENSMUSG00000045827/ ENSMUSG00000001166/ ENSMUSG00000031813/ ENSMUSG00000052684/ ENSMUSG00000035354/ ENSMUSG00000028270/ ENSMUSG00000040253/ ENSMUSG00000035692/ ENSMUSG00000035042/ ENSMUSG00000000838/ ENSMUSG00000027514/ ENSMUSG00000028268/ ENSMUSG00000041827/ ENSMUSG00000066861/ ENSMUSG00000029561/ ENSMUSG00000052776/ ENSMUSG00000104713/ ENSMUSG00000029298/ ENSMUSG00000030921/ ENSMUSG00000026433/ ENSMUSG00000040264/ ENSMUSG00000020641/ ENSMUSG00000027995/ ENSMUSG00000035385/ ENSMUSG00000062300/ ENSMUSG00000020826	
GO:0002250	adaptive immune response	57/815	420/21092	1.23×10^{-16}	3.09×10^{-14}	2.68×10^{-14}	ENSMUSG00000025498/ ENSMUSG00000037321/ ENSMUSG00000024339/ ENSMUSG00000035929/ ENSMUSG00000061232/ ENSMUSG00000073411/ ENSMUSG00000115338/ ENSMUSG00000060802/ ENSMUSG00000024371/ ENSMUSG00000048806/ ENSMUSG00000092243/ ENSMUSG00000067212/ ENSMUSG00000040987/ ENSMUSG00000079491/ ENSMUSG00000056116/ ENSMUSG00000036908/ ENSMUSG00000016206/ ENSMUSG00000070904/ ENSMUSG00000073402/ ENSMUSG00000021583/ ENSMUSG00000028793/ ENSMUSG00000000791/ ENSMUSG00000079507/ ENSMUSG00000021403/ ENSMUSG00000023224/ ENSMUSG00000057058/ ENSMUSG00000060550/ ENSMUSG00000022505/ ENSMUSG00000037447/ ENSMUSG00000032508/ ENSMUSG00000039217/ ENSMUSG00000073409/ ENSMUSG00000020476/ ENSMUSG00000070427/ ENSMUSG00000031154/ ENSMUSG00000055172/ ENSMUSG00000079343/ ENSMUSG00000045827/ ENSMUSG00000004040/	57

							ENSMUSG00000053835/ ENSMUSG00000026405/ ENSMUSG00000041187/ ENSMUSG00000029869/ ENSMUSG00000073418/ ENSMUSG00000016496/ ENSMUSG00000022378/ ENSMUSG00000021453/ ENSMUSG00000020641/ ENSMUSG00000067235/ ENSMUSG00000024810/ ENSMUSG00000018899/ ENSMUSG00000038521/ ENSMUSG00000069255/ ENSMUSG00000030895/ ENSMUSG00000024789/ ENSMUSG00000062300/ ENSMUSG00000025746	
GO:0019221	cytokine−mediated signaling pathway	50/815	332/21092	1.35×10^{-16}	3.20×10^{-14}	2.78×10^{-14}	ENSMUSG00000025498/ ENSMUSG00000026104/ ENSMUSG00000025492/ ENSMUSG00000069874/ ENSMUSG00000034422/ ENSMUSG00000022906/ ENSMUSG00000040033/ ENSMUSG00000027639/ ENSMUSG00000026946/ ENSMUSG00000027951/ ENSMUSG00000048806/ ENSMUSG00000070904/ ENSMUSG00000072244/ ENSMUSG00000002602/ ENSMUSG00000038037/ ENSMUSG00000000127/ ENSMUSG00000032508/ ENSMUSG00000039217/ ENSMUSG00000037860/ ENSMUSG00000022514/ ENSMUSG00000074151/ ENSMUSG00000046879/ ENSMUSG00000004040/ ENSMUSG00000031948/ ENSMUSG00000040329/ ENSMUSG00000064090/ ENSMUSG00000035042/ ENSMUSG00000032690/ ENSMUSG00000027514/ ENSMUSG00000054072/ ENSMUSG00000025888/ ENSMUSG00000034855/ ENSMUSG00000034610/ ENSMUSG00000060183/ ENSMUSG00000029417/ ENSMUSG00000017057/ ENSMUSG00000022146/ ENSMUSG00000035373/ ENSMUSG00000068758/ ENSMUSG00000018899/ ENSMUSG00000033538/ ENSMUSG00000026981/ ENSMUSG00000031712/ ENSMUSG00000014599/ ENSMUSG00000038418/ ENSMUSG00000030895/ ENSMUSG00000024789/ ENSMUSG00000035385/ ENSMUSG00000000078/ ENSMUSG00000025746	50
GO:0043901	negative regulation of multi−organism process	36/815	176/21092	1.48×10^{-16}	3.20×10^{-14}	2.78×10^{-14}	ENSMUSG00000032661/ ENSMUSG00000026104/ ENSMUSG00000025492/ ENSMUSG00000017830/ ENSMUSG00000046718/ ENSMUSG00000024079/ ENSMUSG00000029605/ ENSMUSG00000030966/ ENSMUSG00000027951/ ENSMUSG00000063268/ ENSMUSG00000036986/ ENSMUSG00000029826/	36

							ENSMUSG00000048806/ ENSMUSG00000000275/ ENSMUSG00000040987/ ENSMUSG00000024457/ ENSMUSG00000038884/ ENSMUSG00000072244/ ENSMUSG00000039236/ ENSMUSG00000009585/ ENSMUSG00000066800/ ENSMUSG00000039853/ ENSMUSG00000043279/ ENSMUSG00000032508/ ENSMUSG00000001166/ ENSMUSG00000052684/ ENSMUSG00000032501/ ENSMUSG00000035692/ ENSMUSG00000035042/ ENSMUSG00000041827/ ENSMUSG00000066861/ ENSMUSG00000029561/ ENSMUSG00000052776/ ENSMUSG00000020641/ ENSMUSG00000022126/ ENSMUSG00000027995	
GO:0050792	regulation of viral process	36/815	176/21092	1.48×10^{-16}	3.20×10^{-14}	2.78×10^{-14}	ENSMUSG00000032661/ ENSMUSG00000026104/ ENSMUSG00000025492/ ENSMUSG00000046718/ ENSMUSG00000024079/ ENSMUSG00000029605/ ENSMUSG00000030966/ ENSMUSG00000027951/ ENSMUSG00000063268/ ENSMUSG00000036986/ ENSMUSG00000029826/ ENSMUSG00000048806/ ENSMUSG00000000275/ ENSMUSG00000024457/ ENSMUSG00000038884/ ENSMUSG00000072244/ ENSMUSG00000039236/ ENSMUSG00000009585/ ENSMUSG00000066800/ ENSMUSG00000039853/ ENSMUSG00000043279/ ENSMUSG00000038467/ ENSMUSG00000037331/ ENSMUSG00000001166/ ENSMUSG00000031813/ ENSMUSG00000052684/ ENSMUSG00000035692/ ENSMUSG00000035042/ ENSMUSG00000000838/ ENSMUSG00000041827/ ENSMUSG00000066861/ ENSMUSG00000029561/ ENSMUSG00000052776/ ENSMUSG00000030921/ ENSMUSG00000020641/ ENSMUSG00000062300	36
GO:0002711	positive regulation of T cell mediated immunity	23/815	67/21092	2.48×10^{-16}	5.14×10^{-14}	4.46×10^{-14}	ENSMUSG00000024339/ ENSMUSG00000035929/ ENSMUSG00000061232/ ENSMUSG00000073411/ ENSMUSG00000115338/ ENSMUSG00000060802/ ENSMUSG00000092243/ ENSMUSG00000067212/ ENSMUSG00000079491/ ENSMUSG00000056116/ ENSMUSG00000016206/ ENSMUSG00000073402/ ENSMUSG00000079507/ ENSMUSG00000060550/ ENSMUSG00000037447/ ENSMUSG00000039217/ ENSMUSG00000073409/ ENSMUSG00000053835/	23

							ENSMUSG00000022378/ ENSMUSG00000020641/ ENSMUSG00000067235/ ENSMUSG00000062300/ ENSMUSG00000025746	
GO:0043903	regulation of symbiosis. encompassing mutualism through parasitism	39/815	212/21092	3.57×10^{-16}	7.07×10^{-14}	6.14×10^{-14}	ENSMUSG00000032661/ ENSMUSG00000026104/ ENSMUSG00000025492/ ENSMUSG00000046718/ ENSMUSG00000024079/ ENSMUSG00000029605/ ENSMUSG00000030966/ ENSMUSG00000027951/ ENSMUSG00000063268/ ENSMUSG00000036986/ ENSMUSG00000029826/ ENSMUSG00000048806/ ENSMUSG00000000275/ ENSMUSG00000024457/ ENSMUSG00000038884/ ENSMUSG00000072244/ ENSMUSG00000039236/ ENSMUSG00000009585/ ENSMUSG00000066800/ ENSMUSG00000039853/ ENSMUSG00000021795/ ENSMUSG00000043279/ ENSMUSG00000032508/ ENSMUSG00000038467/ ENSMUSG00000037331/ ENSMUSG00000001166/ ENSMUSG00000031813/ ENSMUSG00000052684/ ENSMUSG00000035692/ ENSMUSG00000035042/ ENSMUSG00000000838/ ENSMUSG00000041827/ ENSMUSG00000066861/ ENSMUSG00000029561/ ENSMUSG00000052776/ ENSMUSG00000030921/ ENSMUSG00000020641/ ENSMUSG00000027995/ ENSMUSG00000062300	39
GO:0001819	positive regulation of cytokine production	57/815	435/21092	6.09×10^{-16}	1.16×10^{-13}	1.01×10^{-13}	ENSMUSG00000025498/ ENSMUSG00000026104/ ENSMUSG00000017830/ ENSMUSG00000026896/ ENSMUSG00000040296/ ENSMUSG00000024079/ ENSMUSG00000060802/ ENSMUSG00000029826/ ENSMUSG00000020707/ ENSMUSG00000001123/ ENSMUSG00000067212/ ENSMUSG00000036908/ ENSMUSG00000016206/ ENSMUSG00000031639/ ENSMUSG00000020357/ ENSMUSG00000072244/ ENSMUSG00000000791/ ENSMUSG00000060550/ ENSMUSG00000071203/ ENSMUSG00000026672/ ENSMUSG00000037447/ ENSMUSG00000032508/ ENSMUSG00000039217/ ENSMUSG00000037860/ ENSMUSG00000022575/ ENSMUSG00000022514/ ENSMUSG00000037921/ ENSMUSG00000040152/ ENSMUSG00000020115/ ENSMUSG00000055204/ ENSMUSG00000042333/ ENSMUSG00000031948/ ENSMUSG00000038058/ ENSMUSG00000040329/ ENSMUSG00000041187/ ENSMUSG00000016496/	57

							ENSMUSG00000022378/ ENSMUSG00000035042/ ENSMUSG00000025888/ ENSMUSG00000015340/ ENSMUSG00000035914/ ENSMUSG00000020641/ ENSMUSG00000002897/ ENSMUSG00000025779/ ENSMUSG00000034610/ ENSMUSG00000024810/ ENSMUSG00000105504/ ENSMUSG00000018899/ ENSMUSG00000033538/ ENSMUSG00000026981/ ENSMUSG00000027995/ ENSMUSG00000031712/ ENSMUSG00000038418/ ENSMUSG00000008734/ ENSMUSG00000024789/ ENSMUSG00000035385/ ENSMUSG00000025746	
GO:0002456	T cell mediated immunity	30/815	128/21092	1.02×10^{-15}	1.87×10^{-13}	1.63×10^{-13}	ENSMUSG00000024339/ ENSMUSG00000035929/ ENSMUSG00000061232/ ENSMUSG00000073411/ ENSMUSG00000115338/ ENSMUSG00000060802/ ENSMUSG00000048806/ ENSMUSG00000092243/ ENSMUSG00000067212/ ENSMUSG00000040987/ ENSMUSG00000079491/ ENSMUSG00000056116/ ENSMUSG00000016206/ ENSMUSG00000073402/ ENSMUSG00000079507/ ENSMUSG00000021403/ ENSMUSG00000060550/ ENSMUSG00000022505/ ENSMUSG00000037447/ ENSMUSG00000039217/ ENSMUSG00000073409/ ENSMUSG00000045827/ ENSMUSG00000053835/ ENSMUSG00000029869/ ENSMUSG00000022378/ ENSMUSG00000020641/ ENSMUSG00000067235/ ENSMUSG00000069255/ ENSMUSG00000062300/ ENSMUSG00000025746	30
GO:0002443	leukocyte mediated immunity	51/815	363/21092	1.25×10^{-15}	2.19×10^{-13}	1.91×10^{-13}	ENSMUSG00000025498/ ENSMUSG00000037321/ ENSMUSG00000024339/ ENSMUSG00000035929/ ENSMUSG00000061232/ ENSMUSG00000073411/ ENSMUSG00000040296/ ENSMUSG00000115338/ ENSMUSG00000060802/ ENSMUSG00000024371/ ENSMUSG00000048806/ ENSMUSG00000092243/ ENSMUSG00000001123/ ENSMUSG00000067212/ ENSMUSG00000040987/ ENSMUSG00000079491/ ENSMUSG00000056116/ ENSMUSG00000016206/ ENSMUSG00000073402/ ENSMUSG00000031639/ ENSMUSG00000030157/ ENSMUSG00000028793/ ENSMUSG00000079507/ ENSMUSG00000021403/ ENSMUSG00000023224/ ENSMUSG00000060550/ ENSMUSG00000022505/ ENSMUSG00000000127/ ENSMUSG00000037447/ ENSMUSG00000032508/	51

							ENSMUSG00000039217/ ENSMUSG00000073409/ ENSMUSG00000025178/ ENSMUSG00000055172/ ENSMUSG00000079343/ ENSMUSG00000004508/ ENSMUSG00000045827/ ENSMUSG00000053835/ ENSMUSG00000026405/ ENSMUSG00000029869/ ENSMUSG00000073418/ ENSMUSG00000022378/ ENSMUSG00000020641/ ENSMUSG00000026797/ ENSMUSG00000067235/ ENSMUSG00000027995/ ENSMUSG00000038521/ ENSMUSG00000069255/ ENSMUSG00000030895/ ENSMUSG00000062300/ ENSMUSG00000025746	
GO:0002449	lymphocyte mediated immunity	44/815	277/21092	1.29×10^{-15}	2.19×10^{-13}	1.91×10^{-13}	ENSMUSG00000025498/ ENSMUSG00000037321/ ENSMUSG00000024339/ ENSMUSG00000035929/ ENSMUSG00000061232/ ENSMUSG00000073411/ ENSMUSG00000115338/ ENSMUSG00000060802/ ENSMUSG00000024371/ ENSMUSG00000048806/ ENSMUSG00000092243/ ENSMUSG00000001123/ ENSMUSG00000067212/ ENSMUSG00000040987/ ENSMUSG00000079491/ ENSMUSG00000056116/ ENSMUSG00000016206/ ENSMUSG00000073402/ ENSMUSG00000030157/ ENSMUSG00000028793/ ENSMUSG00000079507/ ENSMUSG00000021403/ ENSMUSG00000023224/ ENSMUSG00000060550/ ENSMUSG00000022505/ ENSMUSG00000037447/ ENSMUSG00000032508/ ENSMUSG00000039217/ ENSMUSG00000073409/ ENSMUSG00000055172/ ENSMUSG00000079343/ ENSMUSG00000045827/ ENSMUSG00000053835/ ENSMUSG00000026405/ ENSMUSG00000029869/ ENSMUSG00000073418/ ENSMUSG00000022378/ ENSMUSG00000020641/ ENSMUSG00000067235/ ENSMUSG00000038521/ ENSMUSG00000069255/ ENSMUSG00000030895/ ENSMUSG00000062300/ ENSMUSG00000025746	44
GO:0019882	antigen processing and presentation	27/815	103/21092	1.38×10^{-15}	2.26×10^{-13}	1.96×10^{-13}	ENSMUSG00000024338/ ENSMUSG00000096727/ ENSMUSG00000037321/ ENSMUSG00000024339/ ENSMUSG00000035929/ ENSMUSG00000061232/ ENSMUSG00000073411/ ENSMUSG00000024308/ ENSMUSG00000060802/ ENSMUSG00000092243/ ENSMUSG00000067212/ ENSMUSG00000079491/ ENSMUSG00000022216/ ENSMUSG00000056116/ ENSMUSG00000036908/	27

							ENSMUSG00000016206/ ENSMUSG00000079197/ ENSMUSG00000073402/ ENSMUSG00000021583/ ENSMUSG00000038213/ ENSMUSG00000079507/ ENSMUSG00000060550/ ENSMUSG00000073409/ ENSMUSG00000040152/ ENSMUSG00000053835/ ENSMUSG00000038058/ ENSMUSG00000067235	
GO:0001913	T cell mediated cytotoxicity	23/815	72/21092	1.52×10^{-15}	2.41×10^{-13}	2.09×10^{-13}	ENSMUSG00000024339/ ENSMUSG00000035929/ ENSMUSG00000061232/ ENSMUSG00000073411/ ENSMUSG00000115338/ ENSMUSG00000060802/ ENSMUSG00000092243/ ENSMUSG00000067212/ ENSMUSG00000040987/ ENSMUSG00000079491/ ENSMUSG00000056116/ ENSMUSG00000016206/ ENSMUSG00000073402/ ENSMUSG00000079507/ ENSMUSG00000021403/ ENSMUSG00000060550/ ENSMUSG00000022505/ ENSMUSG00000073409/ ENSMUSG00000045827/ ENSMUSG00000053835/ ENSMUSG00000022378/ ENSMUSG00000067235/ ENSMUSG00000062300	23
GO:0001916	positive regulation of T cell mediated cytotoxicity	19/815	46/21092	1.80×10^{-15}	2.76×10^{-13}	2.40×10^{-13}	ENSMUSG00000024339/ ENSMUSG00000035929/ ENSMUSG00000061232/ ENSMUSG00000073411/ ENSMUSG00000115338/ ENSMUSG00000060802/ ENSMUSG00000092243/ ENSMUSG00000067212/ ENSMUSG00000079491/ ENSMUSG00000056116/ ENSMUSG00000016206/ ENSMUSG00000073402/ ENSMUSG00000079507/ ENSMUSG00000060550/ ENSMUSG00000073409/ ENSMUSG00000053835/ ENSMUSG00000022378/ ENSMUSG00000067235/ ENSMUSG00000062300	19
GO:0002709	regulation of T cell mediated immunity	25/815	88/21092	1.98×10^{-15}	2.94×10^{-13}	2.56×10^{-13}	ENSMUSG00000024339/ ENSMUSG00000035929/ ENSMUSG00000061232/ ENSMUSG00000073411/ ENSMUSG00000115338/ ENSMUSG00000060802/ ENSMUSG00000048806/ ENSMUSG00000092243/ ENSMUSG00000067212/ ENSMUSG00000079491/ ENSMUSG00000056116/ ENSMUSG00000016206/ ENSMUSG00000073402/ ENSMUSG00000079507/ ENSMUSG00000060550/ ENSMUSG00000037447/ ENSMUSG00000039217/ ENSMUSG00000073409/ ENSMUSG00000053835/ ENSMUSG00000022378/ ENSMUSG00000020641/	25

							ENSMUSG00000067235/ ENSMUSG00000069255/ ENSMUSG00000062300/ ENSMUSG00000025746	
GO:0019058	viral life cycle	36/815	193/21092	3.15×10^{-15}	4.55×10^{-13}	3.95×10^{-13}	ENSMUSG00000032661/ ENSMUSG00000025492/ ENSMUSG00000046718/ ENSMUSG00000024079/ ENSMUSG00000029605/ ENSMUSG00000030966/ ENSMUSG00000027951/ ENSMUSG00000063268/ ENSMUSG00000036986/ ENSMUSG00000029826/ ENSMUSG00000048806/ ENSMUSG00000000275/ ENSMUSG00000024457/ ENSMUSG00000038884/ ENSMUSG00000072244/ ENSMUSG00000039236/ ENSMUSG00000009585/ ENSMUSG00000066800/ ENSMUSG00000002602/ ENSMUSG00000043279/ ENSMUSG00000038467/ ENSMUSG00000037331/ ENSMUSG00000001166/ ENSMUSG00000031813/ ENSMUSG00000035354/ ENSMUSG00000035692/ ENSMUSG00000035042/ ENSMUSG00000000838/ ENSMUSG00000041827/ ENSMUSG00000066861/ ENSMUSG00000029561/ ENSMUSG00000052776/ ENSMUSG00000030921/ ENSMUSG00000026433/ ENSMUSG00000020641/ ENSMUSG00000062300	36
GO:0002428	antigen processing and presentation of peptide antigen via MHC class Ib	16/815	31/21092	3.73×10^{-15}	5.22×10^{-13}	4.54×10^{-13}	ENSMUSG00000024339/ ENSMUSG00000035929/ ENSMUSG00000061232/ ENSMUSG00000073411/ ENSMUSG00000060802/ ENSMUSG00000092243/ ENSMUSG00000067212/ ENSMUSG00000079491/ ENSMUSG00000056116/ ENSMUSG00000016206/ ENSMUSG00000073402/ ENSMUSG00000079507/ ENSMUSG00000060550/ ENSMUSG00000073409/ ENSMUSG00000053835/ ENSMUSG00000067235	16
GO:0002699	positive regulation of immune effector process	41/815	250/21092	3.97×10^{-15}	5.39×10^{-13}	4.68×10^{-13}	ENSMUSG00000017830/ ENSMUSG00000024339/ ENSMUSG00000035929/ ENSMUSG00000061232/ ENSMUSG00000073411/ ENSMUSG00000040296/ ENSMUSG00000115338/ ENSMUSG00000060802/ ENSMUSG00000029826/ ENSMUSG00000092243/ ENSMUSG00000067212/ ENSMUSG00000040987/ ENSMUSG00000079491/ ENSMUSG00000056116/ ENSMUSG00000016206/ ENSMUSG00000073402/ ENSMUSG00000072244/ ENSMUSG00000079507/ ENSMUSG00000060550/ ENSMUSG00000000127/ ENSMUSG00000037447/	41

							ENSMUSG00000039217/ ENSMUSG00000073409/ ENSMUSG00000037921/ ENSMUSG00000004508/ ENSMUSG00000055204/ ENSMUSG00000042333/ ENSMUSG00000053835/ ENSMUSG00000031948/ ENSMUSG00000022378/ ENSMUSG00000020641/ ENSMUSG00000026797/ ENSMUSG00000067235/ ENSMUSG00000024810/ ENSMUSG00000018899/ ENSMUSG00000027995/ ENSMUSG00000008734/ ENSMUSG00000030895/ ENSMUSG00000035385/ ENSMUSG00000062300/ ENSMUSG00000025746	
GO:0001914	regulation of T cell mediated cytotoxicity	19/815	51/21092	1.75×10^{-14}	2.31×10^{-12}	2.01×10^{-12}	ENSMUSG00000024339/ ENSMUSG00000035929/ ENSMUSG00000061232/ ENSMUSG00000073411/ ENSMUSG00000115338/ ENSMUSG00000060802/ ENSMUSG00000092243/ ENSMUSG00000067212/ ENSMUSG00000079491/ ENSMUSG00000056116/ ENSMUSG00000016206/ ENSMUSG00000073402/ ENSMUSG00000079507/ ENSMUSG00000060550/ ENSMUSG00000073409/ ENSMUSG00000053835/ ENSMUSG00000022378/ ENSMUSG00000067235/ ENSMUSG00000062300	19
GO:0048002	antigen processing and presentation of peptide antigen	21/815	66/21092	2.84×10^{-14}	3.59×10^{-12}	3.12×10^{-12}	ENSMUSG00000037321/ ENSMUSG00000024339/ ENSMUSG00000035929/ ENSMUSG00000061232/ ENSMUSG00000073411/ ENSMUSG00000024308/ ENSMUSG00000060802/ ENSMUSG00000092243/ ENSMUSG00000067212/ ENSMUSG00000079491/ ENSMUSG00000056116/ ENSMUSG00000036908/ ENSMUSG00000016206/ ENSMUSG00000073402/ ENSMUSG00000021583/ ENSMUSG00000038213/ ENSMUSG00000079507/ ENSMUSG00000060550/ ENSMUSG00000073409/ ENSMUSG00000053835/ ENSMUSG00000067235	21
GO:0001909	leukocyte mediated cytotoxicity	29/815	134/21092	2.87×10^{-14}	3.59×10^{-12}	3.12×10^{-12}	ENSMUSG00000037321/ ENSMUSG00000024339/ ENSMUSG00000035929/ ENSMUSG00000061232/ ENSMUSG00000073411/ ENSMUSG00000115338/ ENSMUSG00000060802/ ENSMUSG00000092243/ ENSMUSG00000001123/ ENSMUSG00000067212/ ENSMUSG00000040987/ ENSMUSG00000079491/ ENSMUSG00000056116/ ENSMUSG00000016206/ ENSMUSG00000073402/ ENSMUSG00000030157/ ENSMUSG00000028793/ ENSMUSG00000079507/	29

							ENSMUSG00000021403/ ENSMUSG00000060550/ ENSMUSG00000022505/ ENSMUSG00000039217/ ENSMUSG00000073409/ ENSMUSG00000045827/ ENSMUSG00000053835/ ENSMUSG00000022378/ ENSMUSG00000067235/ ENSMUSG00000035385/ ENSMUSG00000062300	
GO:0045069	regulation of viral genome replication	24/815	90/21092	3.45×10^{-14}	4.21×10^{-12}	3.66×10^{-12}	ENSMUSG00000032661/ ENSMUSG00000025492/ ENSMUSG00000046718/ ENSMUSG00000024079/ ENSMUSG00000029605/ ENSMUSG00000027951/ ENSMUSG00000063268/ ENSMUSG00000029826/ ENSMUSG00000048806/ ENSMUSG00000038884/ ENSMUSG00000072244/ ENSMUSG00000039236/ ENSMUSG00000009585/ ENSMUSG00000066800/ ENSMUSG00000037331/ ENSMUSG00000001166/ ENSMUSG00000035692/ ENSMUSG00000035042/ ENSMUSG00000000838/ ENSMUSG00000041827/ ENSMUSG00000066861/ ENSMUSG00000029561/ ENSMUSG00000052776/ ENSMUSG00000020641	24
GO:0002475	antigen processing and presentation via MHC class Ib	16/815	35/21092	4.36×10^{-14}	5.19×10^{-12}	4.51×10^{-12}	ENSMUSG00000024339/ ENSMUSG00000035929/ ENSMUSG00000061232/ ENSMUSG00000073411/ ENSMUSG00000060802/ ENSMUSG00000092243/ ENSMUSG00000067212/ ENSMUSG00000079491/ ENSMUSG00000056116/ ENSMUSG00000016206/ ENSMUSG00000073402/ ENSMUSG00000079507/ ENSMUSG00000060550/ ENSMUSG00000073409/ ENSMUSG00000053835/ ENSMUSG00000067235	16
GO:0002476	antigen processing and presentation of endogenous peptide antigen via MHC class Ib	15/815	30/21092	5.08×10^{-14}	5.90×10^{-12}	5.12×10^{-12}	ENSMUSG00000024339/ ENSMUSG00000035929/ ENSMUSG00000061232/ ENSMUSG00000073411/ ENSMUSG00000092243/ ENSMUSG00000067212/ ENSMUSG00000079491/ ENSMUSG00000056116/ ENSMUSG00000016206/ ENSMUSG00000073402/ ENSMUSG00000079507/ ENSMUSG00000060550/ ENSMUSG00000073409/ ENSMUSG00000053835/ ENSMUSG00000067235	15
GO:0001910	regulation of leukocyte mediated cytotoxicity	25/815	102/21092	8.39×10^{-14}	9.26×10^{-12}	8.04×10^{-12}	ENSMUSG00000037321/ ENSMUSG00000024339/ ENSMUSG00000035929/ ENSMUSG00000061232/ ENSMUSG00000073411/ ENSMUSG00000115338/ ENSMUSG00000060802/ ENSMUSG00000092243/ ENSMUSG00000001123/ ENSMUSG00000067212/ ENSMUSG00000040987/ ENSMUSG00000079491/	25

							ENSMUSG00000056116/ ENSMUSG00000016206/ ENSMUSG00000073402/ ENSMUSG00000030157/ ENSMUSG00000079507/ ENSMUSG00000060550/ ENSMUSG00000073409/ ENSMUSG00000045827/ ENSMUSG00000053835/ ENSMUSG00000022378/ ENSMUSG00000067235/ ENSMUSG00000035385/ ENSMUSG00000062300	
GO:0031349	positive regulation of defense response	46/815	336/21092	8.45×10^{-14}	9.26×10^{-12}	8.04×10^{-12}	ENSMUSG00000025498/ ENSMUSG00000017830/ ENSMUSG00000069874/ ENSMUSG00000026896/ ENSMUSG00000022906/ ENSMUSG00000040296/ ENSMUSG00000029826/ ENSMUSG00000001123/ ENSMUSG00000067212/ ENSMUSG00000040987/ ENSMUSG00000036908/ ENSMUSG00000016206/ ENSMUSG00000031639/ ENSMUSG00000072244/ ENSMUSG00000056220/ ENSMUSG00000045038/ ENSMUSG00000073643/ ENSMUSG00000026672/ ENSMUSG00000032508/ ENSMUSG00000055447/ ENSMUSG00000037860/ ENSMUSG00000037921/ ENSMUSG00000074151/ ENSMUSG00000046879/ ENSMUSG00000020115/ ENSMUSG00000055204/ ENSMUSG00000031948/ ENSMUSG00000038058/ ENSMUSG00000035042/ ENSMUSG00000027514/ ENSMUSG00000030921/ ENSMUSG00000070056/ ENSMUSG00000020641/ ENSMUSG00000002897/ ENSMUSG00000025779/ ENSMUSG00000024810/ ENSMUSG00000105504/ ENSMUSG00000022126/ ENSMUSG00000018899/ ENSMUSG00000028028/ ENSMUSG00000027995/ ENSMUSG00000008734/ ENSMUSG00000030895/ ENSMUSG00000024789/ ENSMUSG00000062300/ ENSMUSG00000025746	46
GO:0016032	viral process	41/815	273/21092	8.57×10^{-14}	9.26×10^{-12}	8.04×10^{-12}	ENSMUSG00000032661/ ENSMUSG00000026104/ ENSMUSG00000025492/ ENSMUSG00000046718/ ENSMUSG00000024079/ ENSMUSG00000029605/ ENSMUSG00000030966/ ENSMUSG00000027951/ ENSMUSG00000063268/ ENSMUSG00000036986/ ENSMUSG00000029826/ ENSMUSG00000048806/ ENSMUSG00000000275/ ENSMUSG00000024457/ ENSMUSG00000038884/ ENSMUSG00000072244/ ENSMUSG00000039236/ ENSMUSG00000009585/	41

							ENSMUSG00000066800/ ENSMUSG00000039853/ ENSMUSG00000002602/ ENSMUSG00000043279/ ENSMUSG00000022346/ ENSMUSG00000038467/ ENSMUSG00000037331/ ENSMUSG00000001166/ ENSMUSG00000031813/ ENSMUSG00000052684/ ENSMUSG00000035354/ ENSMUSG00000035692/ ENSMUSG00000035042/ ENSMUSG00000000838/ ENSMUSG00000027514/ ENSMUSG00000041827/ ENSMUSG00000066861/ ENSMUSG00000029561/ ENSMUSG00000052776/ ENSMUSG00000030921/ ENSMUSG00000026433/ ENSMUSG00000020641/ ENSMUSG00000062300	
GO:0002831	regulation of response to biotic stimulus	29/815	144/21092	2.04×10^{-13}	2.16×10^{-11}	1.88×10^{-11}	ENSMUSG00000026104/ ENSMUSG00000017830/ ENSMUSG00000049502/ ENSMUSG00000022906/ ENSMUSG00000040296/ ENSMUSG00000036986/ ENSMUSG00000029826/ ENSMUSG00000001123/ ENSMUSG00000040987/ ENSMUSG00000072244/ ENSMUSG00000035151/ ENSMUSG00000009585/ ENSMUSG00000000791/ ENSMUSG00000026672/ ENSMUSG00000037860/ ENSMUSG00000037921/ ENSMUSG00000020115/ ENSMUSG00000055204/ ENSMUSG00000038058/ ENSMUSG00000032501/ ENSMUSG00000016496/ ENSMUSG00000035042/ ENSMUSG00000079363/ ENSMUSG00000025779/ ENSMUSG00000034610/ ENSMUSG00000022126/ ENSMUSG00000027995/ ENSMUSG00000031712/ ENSMUSG00000062300	29
GO:0019079	viral genome replication	25/815	107/21092	2.72×10^{-13}	2.81×10^{-11}	2.44×10^{-11}	ENSMUSG00000032661/ ENSMUSG00000025492/ ENSMUSG00000046718/ ENSMUSG00000024079/ ENSMUSG00000029605/ ENSMUSG00000027951/ ENSMUSG00000063268/ ENSMUSG00000029826/ ENSMUSG00000048806/ ENSMUSG00000038884/ ENSMUSG00000072244/ ENSMUSG00000039236/ ENSMUSG00000009585/ ENSMUSG00000066800/ ENSMUSG00000037331/ ENSMUSG00000001166/ ENSMUSG00000035692/ ENSMUSG00000035042/ ENSMUSG00000000838/ ENSMUSG00000041827/ ENSMUSG00000066861/ ENSMUSG00000029561/ ENSMUSG00000052776/ ENSMUSG00000026433/ ENSMUSG00000020641	25

GO:0031341	regulation of cell killing	26/815	117/21092	3.21×10^{-13}	3.24×10^{-11}	2.82×10^{-11}	ENSMUSG00000037321/ ENSMUSG00000024339/ ENSMUSG00000035929/ ENSMUSG00000061232/ ENSMUSG00000073411/ ENSMUSG00000115338/ ENSMUSG00000060802/ ENSMUSG00000092243/ ENSMUSG00000001123/ ENSMUSG00000067212/ ENSMUSG00000040987/ ENSMUSG00000079491/ ENSMUSG00000056116/ ENSMUSG00000016206/ ENSMUSG00000073402/ ENSMUSG00000030157/ ENSMUSG00000079507/ ENSMUSG00000060550/ ENSMUSG00000073409/ ENSMUSG00000045827/ ENSMUSG00000053835/ ENSMUSG00000022378/ ENSMUSG00000067235/ ENSMUSG00000035385/ ENSMUSG00000062300/ ENSMUSG00000020826	26
GO:0045089	positive regulation of innate immune response	35/815	215/21092	5.19×10^{-13}	5.15×10^{-11}	4.47×10^{-11}	ENSMUSG00000025498/ ENSMUSG00000017830/ ENSMUSG00000069874/ ENSMUSG00000026896/ ENSMUSG00000022906/ ENSMUSG00000040296/ ENSMUSG00000029826/ ENSMUSG00000001123/ ENSMUSG00000067212/ ENSMUSG00000040987/ ENSMUSG00000036908/ ENSMUSG00000016206/ ENSMUSG00000031639/ ENSMUSG00000072244/ ENSMUSG00000045038/ ENSMUSG00000073643/ ENSMUSG00000032508/ ENSMUSG00000037860/ ENSMUSG00000037921/ ENSMUSG00000074151/ ENSMUSG00000046879/ ENSMUSG00000020115/ ENSMUSG00000055204/ ENSMUSG00000027514/ ENSMUSG00000030921/ ENSMUSG00000070056/ ENSMUSG00000020641/ ENSMUSG00000025779/ ENSMUSG00000105504/ ENSMUSG00000022126/ ENSMUSG00000018899/ ENSMUSG00000028028/ ENSMUSG00000027995/ ENSMUSG00000030895/ ENSMUSG00000062300	35
GO:0002703	regulation of leukocyte mediated immunity	37/815	240/21092	6.25×10^{-13}	6.06×10^{-11}	5.27×10^{-11}	ENSMUSG00000037321/ ENSMUSG00000024339/ ENSMUSG00000035929/ ENSMUSG00000061232/ ENSMUSG00000073411/ ENSMUSG00000040296/ ENSMUSG00000115338/ ENSMUSG00000060802/ ENSMUSG00000048806/ ENSMUSG00000092243/ ENSMUSG00000001123/ ENSMUSG00000067212/ ENSMUSG00000040987/ ENSMUSG00000079491/ ENSMUSG00000056116/ ENSMUSG00000016206/ ENSMUSG00000073402/ ENSMUSG00000031639/	37

							ENSMUSG00000030157/ ENSMUSG00000079507/ ENSMUSG00000060550/ ENSMUSG00000000127/ ENSMUSG00000037447/ ENSMUSG00000039217/ ENSMUSG00000073409/ ENSMUSG00000004508/ ENSMUSG00000045827/ ENSMUSG00000053835/ ENSMUSG00000022378/ ENSMUSG00000020641/ ENSMUSG00000026797/ ENSMUSG00000067235/ ENSMUSG00000027995/ ENSMUSG00000069255/ ENSMUSG00000030895/ ENSMUSG00000062300/ ENSMUSG00000025746	
GO:0001906	cell killing	30/815	164/21092	1.10×10^{-12}	1.05×10^{-10}	9.08×10^{-11}	ENSMUSG00000037321/ ENSMUSG00000024339/ ENSMUSG00000035929/ ENSMUSG00000061232/ ENSMUSG00000073411/ ENSMUSG00000115338/ ENSMUSG00000060802/ ENSMUSG00000092243/ ENSMUSG00000001123/ ENSMUSG00000067212/ ENSMUSG00000040987/ ENSMUSG00000079491/ ENSMUSG00000056116/ ENSMUSG00000016206/ ENSMUSG00000073402/ ENSMUSG00000030157/ ENSMUSG00000028793/ ENSMUSG00000079507/ ENSMUSG00000021403/ ENSMUSG00000060550/ ENSMUSG00000022505/ ENSMUSG00000039217/ ENSMUSG00000073409/ ENSMUSG00000045827/ ENSMUSG00000053835/ ENSMUSG00000022378/ ENSMUSG00000067235/ ENSMUSG00000035385/ ENSMUSG00000062300/ ENSMUSG00000020826	30
GO:0002819	regulation of adaptive immune response	33/815	198/21092	1.23×10^{-12}	1.15×10^{-10}	9.96×10^{-11}	ENSMUSG00000025498/ ENSMUSG00000024339/ ENSMUSG00000035929/ ENSMUSG00000061232/ ENSMUSG00000073411/ ENSMUSG00000115338/ ENSMUSG00000060802/ ENSMUSG00000048806/ ENSMUSG00000092243/ ENSMUSG00000067212/ ENSMUSG00000079491/ ENSMUSG00000056116/ ENSMUSG00000016206/ ENSMUSG00000073402/ ENSMUSG00000000791/ ENSMUSG00000079507/ ENSMUSG00000057058/ ENSMUSG00000060550/ ENSMUSG00000037447/ ENSMUSG00000039217/ ENSMUSG00000073409/ ENSMUSG00000031154/ ENSMUSG00000053835/ ENSMUSG00000016496/ ENSMUSG00000022378/ ENSMUSG00000020641/ ENSMUSG00000067235/	33

							ENSMUSG00000024810/ ENSMUSG00000018899/ ENSMUSG00000069255/ ENSMUSG00000030895/ ENSMUSG00000062300/ ENSMUSG00000025746	
GO:0002821	positive regulation of adaptive immune response	28/815	144/21092	1.32×10^{-12}	1.21×10^{-10}	1.05×10^{-10}	ENSMUSG00000024339/ ENSMUSG00000035929/ ENSMUSG00000061232/ ENSMUSG00000073411/ ENSMUSG00000115338/ ENSMUSG00000060802/ ENSMUSG00000092243/ ENSMUSG00000067212/ ENSMUSG00000079491/ ENSMUSG00000056116/ ENSMUSG00000016206/ ENSMUSG00000073402/ ENSMUSG00000000791/ ENSMUSG00000079507/ ENSMUSG00000057058/ ENSMUSG00000060550/ ENSMUSG00000037447/ ENSMUSG00000039217/ ENSMUSG00000073409/ ENSMUSG00000053835/ ENSMUSG00000016496/ ENSMUSG00000022378/ ENSMUSG00000020641/ ENSMUSG00000067235/ ENSMUSG00000018899/ ENSMUSG00000030895/ ENSMUSG00000062300/ ENSMUSG00000025746	28
GO:0001912	positive regulation of leukocyte mediated cytotoxicity	21/815	79/21092	1.48×10^{-12}	1.33×10^{-10}	1.15×10^{-10}	ENSMUSG00000024339/ ENSMUSG00000035929/ ENSMUSG00000061232/ ENSMUSG00000073411/ ENSMUSG00000115338/ ENSMUSG00000060802/ ENSMUSG00000092243/ ENSMUSG00000067212/ ENSMUSG00000040987/ ENSMUSG00000079491/ ENSMUSG00000056116/ ENSMUSG00000016206/ ENSMUSG00000073402/ ENSMUSG00000079507/ ENSMUSG00000060550/ ENSMUSG00000073409/ ENSMUSG00000053835/ ENSMUSG00000022378/ ENSMUSG00000067235/ ENSMUSG00000035385/ ENSMUSG00000062300	21
GO:0002824	positive regulation of adaptive immune response based on somatic recombination of immune receptors built from immunoglobulin superfamily domains	27/815	137/21092	2.38×10^{-12}	2.09×10^{-10}	1.82×10^{-10}	ENSMUSG00000024339/ ENSMUSG00000035929/ ENSMUSG00000061232/ ENSMUSG00000073411/ ENSMUSG00000115338/ ENSMUSG00000060802/ ENSMUSG00000092243/ ENSMUSG00000067212/ ENSMUSG00000079491/ ENSMUSG00000056116/ ENSMUSG00000016206/ ENSMUSG00000073402/ ENSMUSG00000000791/ ENSMUSG00000079507/ ENSMUSG00000060550/ ENSMUSG00000037447/ ENSMUSG00000039217/ ENSMUSG00000073409/ ENSMUSG00000053835/ ENSMUSG00000016496/ ENSMUSG00000022378/	27

							ENSMUSG00000020641/ ENSMUSG00000067235/ ENSMUSG00000018899/ ENSMUSG00000030895/ ENSMUSG00000062300/ ENSMUSG00000025746	
GO:0031343	positive regulation of cell killing	22/815	91/21092	3.51×10^{-12}	3.03×10^{-10}	2.63×10^{-10}	ENSMUSG00000024339/ ENSMUSG00000035929/ ENSMUSG00000061232/ ENSMUSG00000073411/ ENSMUSG00000115338/ ENSMUSG00000060802/ ENSMUSG00000092243/ ENSMUSG00000067212/ ENSMUSG00000040987/ ENSMUSG00000079491/ ENSMUSG00000056116/ ENSMUSG00000016206/ ENSMUSG00000073402/ ENSMUSG00000079507/ ENSMUSG00000060550/ ENSMUSG00000073409/ ENSMUSG00000053835/ ENSMUSG00000022378/ ENSMUSG00000067235/ ENSMUSG00000035385/ ENSMUSG00000062300/ ENSMUSG00000020826	22
GO:0002706	regulation of lymphocyte mediated immunity	31/815	184/21092	4.43×10^{-12}	3.69×10^{-10}	3.21×10^{-10}	ENSMUSG00000037321/ ENSMUSG00000024339/ ENSMUSG00000035929/ ENSMUSG00000061232/ ENSMUSG00000073411/ ENSMUSG00000115338/ ENSMUSG00000060802/ ENSMUSG00000048806/ ENSMUSG00000092243/ ENSMUSG00000001123/ ENSMUSG00000067212/ ENSMUSG00000040987/ ENSMUSG00000079491/ ENSMUSG00000056116/ ENSMUSG00000016206/ ENSMUSG00000073402/ ENSMUSG00000030157/ ENSMUSG00000079507/ ENSMUSG00000060550/ ENSMUSG00000037447/ ENSMUSG00000039217/ ENSMUSG00000073409/ ENSMUSG00000045827/ ENSMUSG00000053835/ ENSMUSG00000022378/ ENSMUSG00000020641/ ENSMUSG00000067235/ ENSMUSG00000069255/ ENSMUSG00000030895/ ENSMUSG00000062300/ ENSMUSG00000025746	31
GO:0002822	regulation of adaptive immune response based on somatic recombination of immune receptors built from immunoglobulin superfamily domains	31/815	184/21092	4.43×10^{-12}	3.69×10^{-10}	3.21×10^{-10}	ENSMUSG00000024339/ ENSMUSG00000035929/ ENSMUSG00000061232/ ENSMUSG00000073411/ ENSMUSG00000115338/ ENSMUSG00000060802/ ENSMUSG00000048806/ ENSMUSG00000092243/ ENSMUSG00000067212/ ENSMUSG00000079491/ ENSMUSG00000056116/ ENSMUSG00000016206/ ENSMUSG00000073402/ ENSMUSG00000000791/ ENSMUSG00000079507/ ENSMUSG00000060550/ ENSMUSG00000037447/ ENSMUSG00000039217/	31

							ENSMUSG00000073409/ ENSMUSG00000031154/ ENSMUSG00000053835/ ENSMUSG00000016496/ ENSMUSG00000022378/ ENSMUSG00000020641/ ENSMUSG00000067235/ ENSMUSG00000024810/ ENSMUSG00000018899/ ENSMUSG00000069255/ ENSMUSG00000030895/ ENSMUSG00000062300/ ENSMUSG00000025746	
GO:0060760	positive regulation of response to cytokine stimulus	17/815	52/21092	4.99×10^{-12}	4.09×10^{-10}	3.55×10^{-10}	ENSMUSG00000025498/ ENSMUSG00000069874/ ENSMUSG00000026896/ ENSMUSG00000034422/ ENSMUSG00000022906/ ENSMUSG00000040296/ ENSMUSG00000072244/ ENSMUSG00000002602/ ENSMUSG00000074151/ ENSMUSG00000046879/ ENSMUSG00000040329/ ENSMUSG00000027514/ ENSMUSG00000025888/ ENSMUSG00000033538/ ENSMUSG00000027995/ ENSMUSG00000014599/ ENSMUSG00000030895	17
GO:0034340	response to type I interferon	15/815	39/21092	5.95×10^{-12}	4.80×10^{-10}	4.17×10^{-10}	ENSMUSG00000025498/ ENSMUSG00000026104/ ENSMUSG00000025492/ ENSMUSG00000040033/ ENSMUSG00000027639/ ENSMUSG00000027951/ ENSMUSG00000048806/ ENSMUSG00000038884/ ENSMUSG00000072244/ ENSMUSG00000043279/ ENSMUSG00000032508/ ENSMUSG00000074151/ ENSMUSG00000035692/ ENSMUSG00000032690/ ENSMUSG00000027514	15
GO:0002253	activation of immune response	45/815	365/21092	6.46×10^{-12}	5.12×10^{-10}	4.45×10^{-10}	ENSMUSG00000025498/ ENSMUSG00000017830/ ENSMUSG00000069874/ ENSMUSG00000026896/ ENSMUSG00000040296/ ENSMUSG00000029826/ ENSMUSG00000024371/ ENSMUSG00000001123/ ENSMUSG00000047098/ ENSMUSG00000040987/ ENSMUSG00000036908/ ENSMUSG00000036461/ ENSMUSG00000031639/ ENSMUSG00000045038/ ENSMUSG00000023224/ ENSMUSG00000057058/ ENSMUSG00000029640/ ENSMUSG00000073643/ ENSMUSG00000032508/ ENSMUSG00000037860/ ENSMUSG00000037921/ ENSMUSG00000055172/ ENSMUSG00000079343/ ENSMUSG00000020115/ ENSMUSG00000055204/ ENSMUSG00000027466/ ENSMUSG00000026405/ ENSMUSG00000041187/ ENSMUSG00000073418/ ENSMUSG00000015451/ ENSMUSG00000030921/ ENSMUSG00000026433/ ENSMUSG00000070056/	45

							ENSMUSG00000035914/ ENSMUSG00000020641/ ENSMUSG00000026365/ ENSMUSG00000025779/ ENSMUSG00000090231/ ENSMUSG00000022126/ ENSMUSG00000018899/ ENSMUSG00000028028/ ENSMUSG00000027995/ ENSMUSG00000038521/ ENSMUSG00000069255/ ENSMUSG00000062300	
GO:0060759	regulation of response to cytokine stimulus	24/815	115/21092	1.09×10^{-11}	8.53×10^{-10}	7.41×10^{-10}	ENSMUSG00000025498/ ENSMUSG00000069874/ ENSMUSG00000026896/ ENSMUSG00000034422/ ENSMUSG00000022906/ ENSMUSG00000027639/ ENSMUSG00000040296/ ENSMUSG00000027951/ ENSMUSG00000072244/ ENSMUSG00000002602/ ENSMUSG00000038037/ ENSMUSG00000003032/ ENSMUSG00000074151/ ENSMUSG00000046879/ ENSMUSG00000040329/ ENSMUSG00000064090/ ENSMUSG00000027514/ ENSMUSG00000025888/ ENSMUSG00000033538/ ENSMUSG00000026981/ ENSMUSG00000027995/ ENSMUSG00000014599/ ENSMUSG00000030895/ ENSMUSG00000025746	24
GO:0002705	positive regulation of leukocyte mediated immunity	28/815	169/21092	7.24×10^{-11}	5.56×10^{-09}	4.83×10^{-09}	ENSMUSG00000024339/ ENSMUSG00000035929/ ENSMUSG00000061232/ ENSMUSG00000073411/ ENSMUSG00000040296/ ENSMUSG00000115338/ ENSMUSG00000060802/ ENSMUSG00000092243/ ENSMUSG00000067212/ ENSMUSG00000040987/ ENSMUSG00000079491/ ENSMUSG00000056116/ ENSMUSG00000016206/ ENSMUSG00000073402/ ENSMUSG00000079507/ ENSMUSG00000060550/ ENSMUSG00000037447/ ENSMUSG00000039217/ ENSMUSG00000073409/ ENSMUSG00000004508/ ENSMUSG00000053835/ ENSMUSG00000022378/ ENSMUSG00000020641/ ENSMUSG00000026797/ ENSMUSG00000067235/ ENSMUSG00000030895/ ENSMUSG00000062300/ ENSMUSG00000025746	28
GO:0002708	positive regulation of lymphocyte mediated immunity	25/815	137/21092	8.91×10^{-11}	6.73×10^{-09}	5.85×10^{-09}	ENSMUSG00000024339/ ENSMUSG00000035929/ ENSMUSG00000061232/ ENSMUSG00000073411/ ENSMUSG00000115338/ ENSMUSG00000060802/ ENSMUSG00000092243/ ENSMUSG00000067212/ ENSMUSG00000040987/ ENSMUSG00000079491/ ENSMUSG00000056116/ ENSMUSG00000016206/ ENSMUSG00000073402/ ENSMUSG00000079507/ ENSMUSG00000060550/	25

							ENSMUSG00000037447/ ENSMUSG00000039217/ ENSMUSG00000073409/ ENSMUSG00000053835/ ENSMUSG00000022378/ ENSMUSG00000020641/ ENSMUSG00000067235/ ENSMUSG00000030895/ ENSMUSG00000062300/ ENSMUSG00000025746	
GO:0002474	antigen processing and presentation of peptide antigen via MHC class I	11/815	22/21092	1.28×10^{-10}	9.52×10^{-09}	8.27×10^{-09}	ENSMUSG00000037321/ ENSMUSG00000024339/ ENSMUSG00000061232/ ENSMUSG00000073411/ ENSMUSG00000024308/ ENSMUSG00000060802/ ENSMUSG00000067212/ ENSMUSG00000021583/ ENSMUSG00000038213/ ENSMUSG00000060550/ ENSMUSG00000067235	11
GO:0060333	interferon−gamma−mediated signaling pathway	11/815	23/21092	2.37×10^{-10}	1.73×10^{-08}	1.51×10^{-08}	ENSMUSG00000026104/ ENSMUSG00000069874/ ENSMUSG00000034422/ ENSMUSG00000022906/ ENSMUSG00000026946/ ENSMUSG00000038037/ ENSMUSG00000074151/ ENSMUSG00000046879/ ENSMUSG00000018899/ ENSMUSG00000030895/ ENSMUSG00000024789	11
GO:0035455	response to interferon−alpha	11/815	24/21092	4.22×10^{-10}	3.04×10^{-08}	2.64×10^{-08}	ENSMUSG00000078922/ ENSMUSG00000025492/ ENSMUSG00000046718/ ENSMUSG00000024079/ ENSMUSG00000027951/ ENSMUSG00000045932/ ENSMUSG00000002602/ ENSMUSG00000022346/ ENSMUSG00000073489/ ENSMUSG00000034459/ ENSMUSG00000074896	11
GO:0050688	regulation of defense response to virus	18/815	76/21092	4.66×10^{-10}	3.31×10^{-08}	2.87×10^{-08}	ENSMUSG00000026104/ ENSMUSG00000017830/ ENSMUSG00000049502/ ENSMUSG00000022906/ ENSMUSG00000040296/ ENSMUSG00000036986/ ENSMUSG00000029826/ ENSMUSG00000040987/ ENSMUSG00000072244/ ENSMUSG00000035151/ ENSMUSG00000009585/ ENSMUSG00000000791/ ENSMUSG00000037860/ ENSMUSG00000037921/ ENSMUSG00000055204/ ENSMUSG00000035042/ ENSMUSG00000079363/ ENSMUSG00000031712	18
GO:0001961	positive regulation of cytokine−mediated signaling pathway	14/815	45/21092	8.11×10^{-10}	5.67×10^{-08}	4.93×10^{-08}	ENSMUSG00000025498/ ENSMUSG00000069874/ ENSMUSG00000034422/ ENSMUSG00000022906/ ENSMUSG00000072244/ ENSMUSG00000002602/ ENSMUSG00000074151/ ENSMUSG00000046879/ ENSMUSG00000040329/ ENSMUSG00000027514/ ENSMUSG00000025888/ ENSMUSG00000033538/ ENSMUSG00000014599/ ENSMUSG00000030895	14

GO:0045824	negative regulation of innate immune response	15/815	53/21092	8.93×10^{-10}	6.15×10^{-08}	5.34×10^{-08}	ENSMUSG00000017830/ ENSMUSG00000034422/ ENSMUSG00000037321/ ENSMUSG00000024339/ ENSMUSG00000027639/ ENSMUSG00000042726/ ENSMUSG00000026946/ ENSMUSG00000027951/ ENSMUSG00000001123/ ENSMUSG00000067212/ ENSMUSG00000030157/ ENSMUSG00000023224/ ENSMUSG00000074151/ ENSMUSG00000045827/ ENSMUSG00000022126	15
GO:0032606	type I interferon production	18/815	81/21092	1.42×10^{-09}	9.62×10^{-08}	8.36×10^{-08}	ENSMUSG00000025498/ ENSMUSG00000026104/ ENSMUSG00000017830/ ENSMUSG00000026896/ ENSMUSG00000040296/ ENSMUSG00000026946/ ENSMUSG00000002325/ ENSMUSG00000029826/ ENSMUSG00000020707/ ENSMUSG00000031639/ ENSMUSG00000043279/ ENSMUSG00000032508/ ENSMUSG00000039285/ ENSMUSG00000020115/ ENSMUSG00000079363/ ENSMUSG00000022126/ ENSMUSG00000018899/ ENSMUSG00000027995	18
GO:0060337	type I interferon signaling pathway	12/815	33/21092	1.72×10^{-09}	1.13×10^{-07}	9.85×10^{-08}	ENSMUSG00000025498/ ENSMUSG00000026104/ ENSMUSG00000025492/ ENSMUSG00000040033/ ENSMUSG00000027639/ ENSMUSG00000027951/ ENSMUSG00000048806/ ENSMUSG00000072244/ ENSMUSG00000032508/ ENSMUSG00000074151/ ENSMUSG00000032690/ ENSMUSG00000027514	12
GO:0071357	cellular response to type I interferon	12/815	33/21092	1.72×10^{-09}	1.13×10^{-07}	9.85×10^{-08}	ENSMUSG00000025498/ ENSMUSG00000026104/ ENSMUSG00000025492/ ENSMUSG00000040033/ ENSMUSG00000027639/ ENSMUSG00000027951/ ENSMUSG00000048806/ ENSMUSG00000072244/ ENSMUSG00000032508/ ENSMUSG00000074151/ ENSMUSG00000032690/ ENSMUSG00000027514	12
GO:0001959	regulation of cytokine−mediated signaling pathway	20/815	103/21092	2.19×10^{-09}	1.42×10^{-07}	1.24×10^{-07}	ENSMUSG00000025498/ ENSMUSG00000069874/ ENSMUSG00000034422/ ENSMUSG00000022906/ ENSMUSG00000027639/ ENSMUSG00000027951/ ENSMUSG00000072244/ ENSMUSG00000002602/ ENSMUSG00000038037/ ENSMUSG00000074151/ ENSMUSG00000046879/ ENSMUSG00000040329/ ENSMUSG00000064090/ ENSMUSG00000027514/ ENSMUSG00000025888/ ENSMUSG00000033538/ ENSMUSG00000026981/ ENSMUSG00000014599/ ENSMUSG00000030895/ ENSMUSG00000025746	20

GO:0002218	activation of innate immune response	24/815	148/21092	2.56×10^{-09}	1.65×10^{-07}	1.43×10^{-07}	ENSMUSG00000025498/ ENSMUSG00000017830/ ENSMUSG00000069874/ ENSMUSG00000026896/ ENSMUSG00000040296/ ENSMUSG00000029826/ ENSMUSG00000001123/ ENSMUSG00000036908/ ENSMUSG00000031639/ ENSMUSG00000045038/ ENSMUSG00000073643/ ENSMUSG00000032508/ ENSMUSG00000037860/ ENSMUSG00000037921/ ENSMUSG00000020115/ ENSMUSG00000055204/ ENSMUSG00000030921/ ENSMUSG00000070056/ ENSMUSG00000020641/ ENSMUSG00000025779/ ENSMUSG00000022126/ ENSMUSG00000018899/ ENSMUSG00000028028/ ENSMUSG00000027995	24
GO:0002221	pattern recognition receptor signaling pathway	22/815	128/21092	3.86×10^{-09}	2.45×10^{-07}	2.13×10^{-07}	ENSMUSG00000025498/ ENSMUSG00000017830/ ENSMUSG00000069874/ ENSMUSG00000026896/ ENSMUSG00000040296/ ENSMUSG00000029826/ ENSMUSG00000001123/ ENSMUSG00000036908/ ENSMUSG00000031639/ ENSMUSG00000045038/ ENSMUSG00000073643/ ENSMUSG00000032508/ ENSMUSG00000037921/ ENSMUSG00000055204/ ENSMUSG00000030921/ ENSMUSG00000070056/ ENSMUSG00000020641/ ENSMUSG00000025779/ ENSMUSG00000022126/ ENSMUSG00000018899/ ENSMUSG00000028028/ ENSMUSG00000027995	22
GO:0002758	innate immune response–activating signal transduction	22/815	130/21092	5.21×10^{-09}	3.26×10^{-07}	2.83×10^{-07}	ENSMUSG00000025498/ ENSMUSG00000017830/ ENSMUSG00000069874/ ENSMUSG00000026896/ ENSMUSG00000040296/ ENSMUSG00000029826/ ENSMUSG00000001123/ ENSMUSG00000036908/ ENSMUSG00000031639/ ENSMUSG00000045038/ ENSMUSG00000073643/ ENSMUSG00000032508/ ENSMUSG00000037921/ ENSMUSG00000055204/ ENSMUSG00000030921/ ENSMUSG00000070056/ ENSMUSG00000020641/ ENSMUSG00000025779/ ENSMUSG00000022126/ ENSMUSG00000018899/ ENSMUSG00000028028/ ENSMUSG00000027995	22
GO:0002237	response to molecule of bacterial origin	39/815	358/21092	6.06×10^{-09}	3.75×10^{-07}	3.25×10^{-07}	ENSMUSG00000026104/ ENSMUSG00000069874/ ENSMUSG00000024339/ ENSMUSG00000024079/ ENSMUSG00000060802/ ENSMUSG00000001123/ ENSMUSG00000016206/ ENSMUSG00000072244/ ENSMUSG00000002602/	39

							ENSMUSG00000045038/ ENSMUSG00000037580/ ENSMUSG00000000127/ ENSMUSG00000037447/ ENSMUSG00000032508/ ENSMUSG00000039217/ ENSMUSG00000052684/ ENSMUSG00000028270/ ENSMUSG00000020638/ ENSMUSG00000032501/ ENSMUSG00000016496/ ENSMUSG00000105096/ ENSMUSG00000035042/ ENSMUSG00000104713/ ENSMUSG00000025888/ ENSMUSG00000034855/ ENSMUSG00000025779/ ENSMUSG00000034610/ ENSMUSG00000060183/ ENSMUSG00000029417/ ENSMUSG00000022126/ ENSMUSG00000026981/ ENSMUSG00000027995/ ENSMUSG00000032372/ ENSMUSG00000024789/ ENSMUSG00000035385/ ENSMUSG00000020826/ ENSMUSG00000028599/ ENSMUSG00000025746/ ENSMUSG00000036402	
GO:0002764	immune response−regulating signaling pathway	36/815	315/21092	6.62×10^{-09}	4.04×10^{-07}	3.51×10^{-07}	ENSMUSG00000025498/ ENSMUSG00000017830/ ENSMUSG00000069874/ ENSMUSG00000026896/ ENSMUSG00000040296/ ENSMUSG00000029826/ ENSMUSG00000001123/ ENSMUSG00000047098/ ENSMUSG00000040987/ ENSMUSG00000036908/ ENSMUSG00000016206/ ENSMUSG00000036461/ ENSMUSG00000031639/ ENSMUSG00000045038/ ENSMUSG00000057058/ ENSMUSG00000029640/ ENSMUSG00000073643/ ENSMUSG00000000127/ ENSMUSG00000026417/ ENSMUSG00000032508/ ENSMUSG00000037921/ ENSMUSG00000055204/ ENSMUSG00000027466/ ENSMUSG00000041187/ ENSMUSG00000030921/ ENSMUSG00000026433/ ENSMUSG00000070056/ ENSMUSG00000035914/ ENSMUSG00000020641/ ENSMUSG00000025779/ ENSMUSG00000022126/ ENSMUSG00000018899/ ENSMUSG00000028028/ ENSMUSG00000027995/ ENSMUSG00000069255/ ENSMUSG00000062300	36
GO:0042110	T cell activation	47/815	485/21092	7.61×10^{-09}	4.58×10^{-07}	3.98×10^{-07}	ENSMUSG00000031897/ ENSMUSG00000115338/ ENSMUSG00000060802/ ENSMUSG00000048806/ ENSMUSG00000001123/ ENSMUSG00000067212/ ENSMUSG00000040987/ ENSMUSG00000016206/ ENSMUSG00000070904/ ENSMUSG00000057554/ ENSMUSG00000000791/ ENSMUSG00000021795/ ENSMUSG00000038037/ ENSMUSG00000039217/ ENSMUSG00000055447/	47

							ENSMUSG00000020918/ ENSMUSG00000031207/ ENSMUSG00000039285/ ENSMUSG00000031154/ ENSMUSG00000066877/ ENSMUSG00000024238/ ENSMUSG00000042333/ ENSMUSG00000004040/ ENSMUSG00000040329/ ENSMUSG00000029869/ ENSMUSG00000032815/ ENSMUSG00000031304/ ENSMUSG00000090958/ ENSMUSG00000016496/ ENSMUSG00000022378/ ENSMUSG00000035042/ ENSMUSG00000021453/ ENSMUSG00000026433/ ENSMUSG00000035914/ ENSMUSG00000020641/ ENSMUSG00000056153/ ENSMUSG00000037440/ ENSMUSG00000038855/ ENSMUSG00000018899/ ENSMUSG00000069255/ ENSMUSG00000031712/ ENSMUSG00000039153/ ENSMUSG00000038418/ ENSMUSG00000035385/ ENSMUSG00000034165/ ENSMUSG00000021127/ ENSMUSG00000025746	
GO:0098586	cellular response to virus	13/815	47/21092	1.62×10^{-08}	9.64×10^{-07}	8.37×10^{-07}	ENSMUSG00000017830/ ENSMUSG00000069874/ ENSMUSG00000026896/ ENSMUSG00000040296/ ENSMUSG00000027951/ ENSMUSG00000029826/ ENSMUSG00000048806/ ENSMUSG00000001123/ ENSMUSG00000057554/ ENSMUSG00000072244/ ENSMUSG00000037921/ ENSMUSG00000055204/ ENSMUSG00000000838	13
GO:0042742	defense response to bacterium	32/815	272/21092	2.29×10^{-08}	1.35×10^{-06}	1.17×10^{-06}	ENSMUSG00000069874/ ENSMUSG00000061232/ ENSMUSG00000048806/ ENSMUSG00000001123/ ENSMUSG00000067212/ ENSMUSG00000029322/ ENSMUSG00000016206/ ENSMUSG00000078942/ ENSMUSG00000078945/ ENSMUSG00000021795/ ENSMUSG00000071203/ ENSMUSG00000026672/ ENSMUSG00000032508/ ENSMUSG00000022575/ ENSMUSG00000045827/ ENSMUSG00000020115/ ENSMUSG00000055204/ ENSMUSG00000042333/ ENSMUSG00000038058/ ENSMUSG00000028270/ ENSMUSG00000040253/ ENSMUSG00000035692/ ENSMUSG00000105096/ ENSMUSG00000046805/ ENSMUSG00000028268/ ENSMUSG00000054072/ ENSMUSG00000104713/ ENSMUSG00000029298/ ENSMUSG00000040264/ ENSMUSG00000027995/ ENSMUSG00000062210/ ENSMUSG00000020826	32

GO:0060700	regulation of ribonuclease activity	8/815	15/21092	2.43×10^{-08}	1.41×10^{-06}	1.22×10^{-06}	ENSMUSG00000032661/ ENSMUSG00000029605/ ENSMUSG00000001166/ ENSMUSG00000032690/ ENSMUSG00000041827/ ENSMUSG00000066861/ ENSMUSG00000029561/ ENSMUSG00000052776	8
GO:0002683	negative regulation of immune system process	44/815	459/21092	3.17×10^{-08}	1.82×10^{-06}	1.58×10^{-06}	ENSMUSG00000017830/ ENSMUSG00000034422/ ENSMUSG00000037321/ ENSMUSG00000024339/ ENSMUSG00000027639/ ENSMUSG00000042726/ ENSMUSG00000026946/ ENSMUSG00000027951/ ENSMUSG00000048806/ ENSMUSG00000001123/ ENSMUSG00000067212/ ENSMUSG00000040987/ ENSMUSG00000016206/ ENSMUSG00000036461/ ENSMUSG00000034218/ ENSMUSG00000027204/ ENSMUSG00000030157/ ENSMUSG00000014773/ ENSMUSG00000038213/ ENSMUSG00000002602/ ENSMUSG00000023224/ ENSMUSG00000021795/ ENSMUSG00000038037/ ENSMUSG00000032698/ ENSMUSG00000000127/ ENSMUSG00000039217/ ENSMUSG00000022346/ ENSMUSG00000074151/ ENSMUSG00000040152/ ENSMUSG00000045827/ ENSMUSG00000042333/ ENSMUSG00000032501/ ENSMUSG00000090958/ ENSMUSG00000016496/ ENSMUSG00000030921/ ENSMUSG00000070056/ ENSMUSG00000035914/ ENSMUSG00000056153/ ENSMUSG00000024810/ ENSMUSG00000022126/ ENSMUSG00000038855/ ENSMUSG00000018899/ ENSMUSG00000069255/ ENSMUSG00000021127	44
GO:0050691	regulation of defense response to virus by host	12/815	43/21092	5.20×10^{-08}	2.94×10^{-06}	2.56×10^{-06}	ENSMUSG00000026104/ ENSMUSG00000049502/ ENSMUSG00000022906/ ENSMUSG00000040296/ ENSMUSG00000036986/ ENSMUSG00000029826/ ENSMUSG00000040987/ ENSMUSG00000072244/ ENSMUSG00000000791/ ENSMUSG00000037860/ ENSMUSG00000035042/ ENSMUSG00000031712	12
GO:0032607	interferon−alpha production	10/815	29/21092	7.21×10^{-08}	4.04×10^{-06}	3.51×10^{-06}	ENSMUSG00000025498/ ENSMUSG00000026104/ ENSMUSG00000026896/ ENSMUSG00000040296/ ENSMUSG00000026946/ ENSMUSG00000029826/ ENSMUSG00000031639/ ENSMUSG00000039285/ ENSMUSG00000020115/ ENSMUSG00000079363	10

GO:0002757	immune response−activating signal transduction	33/815	301/21092	7.68×10^{-08}	4.25×10^{-06}	3.69×10^{-06}	ENSMUSG00000025498/ ENSMUSG00000017830/ ENSMUSG00000069874/ ENSMUSG00000026896/ ENSMUSG00000040296/ ENSMUSG00000029826/ ENSMUSG00000001123/ ENSMUSG00000047098/ ENSMUSG00000040987/ ENSMUSG00000036908/ ENSMUSG00000036461/ ENSMUSG00000031639/ ENSMUSG00000045038/ ENSMUSG00000057058/ ENSMUSG00000029640/ ENSMUSG00000073643/ ENSMUSG00000032508/ ENSMUSG00000037921/ ENSMUSG00000055204/ ENSMUSG00000027466/ ENSMUSG00000041187/ ENSMUSG00000030921/ ENSMUSG00000026433/ ENSMUSG00000070056/ ENSMUSG00000035914/ ENSMUSG00000020641/ ENSMUSG00000025779/ ENSMUSG00000022126/ ENSMUSG00000018899/ ENSMUSG00000028028/ ENSMUSG00000027995/ ENSMUSG00000069255/ ENSMUSG00000062300	33
GO:0002833	positive regulation of response to biotic stimulus	13/815	53/21092	7.82×10^{-08}	4.28×10^{-06}	3.72×10^{-06}	ENSMUSG00000017830/ ENSMUSG00000029826/ ENSMUSG00000001123/ ENSMUSG00000026672/ ENSMUSG00000037921/ ENSMUSG00000020115/ ENSMUSG00000055204/ ENSMUSG00000038058/ ENSMUSG00000016496/ ENSMUSG00000025779/ ENSMUSG00000022126/ ENSMUSG00000027995/ ENSMUSG00000062300	13
GO:0044406	adhesion of symbiont to host	8/815	17/21092	8.56×10^{-08}	4.63×10^{-06}	4.02×10^{-06}	ENSMUSG00000021795/ ENSMUSG00000028270/ ENSMUSG00000040253/ ENSMUSG00000028268/ ENSMUSG00000104713/ ENSMUSG00000029298/ ENSMUSG00000040264/ ENSMUSG00000062300	8
GO:0050663	cytokine secretion	28/815	232/21092	1.00×10^{-07}	5.35×10^{-06}	4.65×10^{-06}	ENSMUSG00000026896/ ENSMUSG00000040296/ ENSMUSG00000036986/ ENSMUSG00000001123/ ENSMUSG00000036908/ ENSMUSG00000072244/ ENSMUSG00000028793/ ENSMUSG00000038037/ ENSMUSG00000026672/ ENSMUSG00000037447/ ENSMUSG00000037860/ ENSMUSG00000022575/ ENSMUSG00000022514/ ENSMUSG00000031154/ ENSMUSG00000042333/ ENSMUSG00000031948/ ENSMUSG00000029869/ ENSMUSG00000090958/ ENSMUSG00000016496/ ENSMUSG00000025888/ ENSMUSG00000035914/ ENSMUSG00000002897/ ENSMUSG00000024810/ ENSMUSG00000105504/	28

							ENSMUSG00000033538/ ENSMUSG00000027995/ ENSMUSG00000020826/ ENSMUSG00000025746	
GO:0032496	response to lipopolysaccharide	35/815	340/21092	1.49×10^{-07}	7.85×10^{-06}	6.82×10^{-06}	ENSMUSG00000026104/ ENSMUSG00000069874/ ENSMUSG00000024079/ ENSMUSG00000001123/ ENSMUSG00000072244/ ENSMUSG00000002602/ ENSMUSG00000045038/ ENSMUSG00000037580/ ENSMUSG00000000127/ ENSMUSG00000037447/ ENSMUSG00000032508/ ENSMUSG00000039217/ ENSMUSG00000052684/ ENSMUSG00000028270/ ENSMUSG00000020638/ ENSMUSG00000032501/ ENSMUSG00000016496/ ENSMUSG00000105096/ ENSMUSG00000035042/ ENSMUSG00000104713/ ENSMUSG00000025888/ ENSMUSG00000034855/ ENSMUSG00000025779/ ENSMUSG00000034610/ ENSMUSG00000060183/ ENSMUSG00000029417/ ENSMUSG00000022126/ ENSMUSG00000026981/ ENSMUSG00000032372/ ENSMUSG00000024789/ ENSMUSG00000035385/ ENSMUSG00000020826/ ENSMUSG00000028599/ ENSMUSG00000025746/ ENSMUSG00000036402	35
GO:0050867	positive regulation of cell activation	34/815	328/21092	1.89×10^{-07}	9.90×10^{-06}	8.60×10^{-06}	ENSMUSG00000115338/ ENSMUSG00000027950/ ENSMUSG00000001123/ ENSMUSG00000067212/ ENSMUSG00000016206/ ENSMUSG00000057554/ ENSMUSG00000000791/ ENSMUSG00000002602/ ENSMUSG00000038037/ ENSMUSG00000032508/ ENSMUSG00000039217/ ENSMUSG00000055447/ ENSMUSG00000040152/ ENSMUSG00000110206/ ENSMUSG00000066877/ ENSMUSG00000004508/ ENSMUSG00000031948/ ENSMUSG00000040329/ ENSMUSG00000029869/ ENSMUSG00000031304/ ENSMUSG00000016496/ ENSMUSG00000022378/ ENSMUSG00000035042/ ENSMUSG00000035914/ ENSMUSG00000026797/ ENSMUSG00000024810/ ENSMUSG00000037440/ ENSMUSG00000038855/ ENSMUSG00000018899/ ENSMUSG00000031712/ ENSMUSG00000024789/ ENSMUSG00000035385/ ENSMUSG00000062300/ ENSMUSG00000025746	34

GO:0002696	positive regulation of leukocyte activation	33/815	313/21092	1.93×10^{-07}	9.98×10^{-06}	8.67×10^{-06}	ENSMUSG00000115338/ ENSMUSG00000027950/ ENSMUSG00000001123/ ENSMUSG00000067212/ ENSMUSG00000016206/ ENSMUSG00000057554/ ENSMUSG00000000791/ ENSMUSG00000002602/ ENSMUSG00000038037/ ENSMUSG00000032508/ ENSMUSG00000039217/ ENSMUSG00000055447/ ENSMUSG00000040152/ ENSMUSG00000110206/ ENSMUSG00000066877/ ENSMUSG00000004508/ ENSMUSG00000031948/ ENSMUSG00000040329/ ENSMUSG00000029869/ ENSMUSG00000031304/ ENSMUSG00000016496/ ENSMUSG00000022378/ ENSMUSG00000035042/ ENSMUSG00000035914/ ENSMUSG00000026797/ ENSMUSG00000024810/ ENSMUSG00000037440/ ENSMUSG00000038855/ ENSMUSG00000018899/ ENSMUSG00000031712/ ENSMUSG00000035385/ ENSMUSG00000062300/ ENSMUSG00000025746	33
GO:0022407	regulation of cell−cell adhesion	38/815	392/21092	2.08×10^{-07}	1.05×10^{-05}	9.10×10^{-06}	ENSMUSG00000115338/ ENSMUSG00000048806/ ENSMUSG00000001123/ ENSMUSG00000067212/ ENSMUSG00000016206/ ENSMUSG00000057554/ ENSMUSG00000000791/ ENSMUSG00000057058/ ENSMUSG00000021795/ ENSMUSG00000021360/ ENSMUSG00000038037/ ENSMUSG00000003032/ ENSMUSG00000039217/ ENSMUSG00000055447/ ENSMUSG00000004936/ ENSMUSG00000066877/ ENSMUSG00000017446/ ENSMUSG00000042333/ ENSMUSG00000040329/ ENSMUSG00000029869/ ENSMUSG00000031304/ ENSMUSG00000090958/ ENSMUSG00000016496/ ENSMUSG00000022378/ ENSMUSG00000035042/ ENSMUSG00000035914/ ENSMUSG00000056153/ ENSMUSG00000037440/ ENSMUSG00000038855/ ENSMUSG00000018899/ ENSMUSG00000026981/ ENSMUSG00000022676/ ENSMUSG00000069255/ ENSMUSG00000031712/ ENSMUSG00000028289/ ENSMUSG00000024789/ ENSMUSG00000035385/ ENSMUSG00000025746	38
GO:0042832	defense response to protozoan	10/815	32/21092	2.09×10^{-07}	1.05×10^{-05}	9.10×10^{-06}	ENSMUSG00000069874/ ENSMUSG00000028270/ ENSMUSG00000040253/ ENSMUSG00000105096/ ENSMUSG00000028268/ ENSMUSG00000054072/	10

							ENSMUSG00000104713/ ENSMUSG00000029298/ ENSMUSG00000040264/ ENSMUSG00000025746	
GO:1903037	regulation of leukocyte cell−cell adhesion	31/815	284/21092	2.09×10^{-07}	1.05×10^{-05}	9.10×10^{-06}	ENSMUSG00000115338/ ENSMUSG00000048806/ ENSMUSG00000001123/ ENSMUSG00000067212/ ENSMUSG00000016206/ ENSMUSG00000057554/ ENSMUSG00000000791/ ENSMUSG00000057058/ ENSMUSG00000021795/ ENSMUSG00000038037/ ENSMUSG00000003032/ ENSMUSG00000039217/ ENSMUSG00000055447/ ENSMUSG00000066877/ ENSMUSG00000042333/ ENSMUSG00000040329/ ENSMUSG00000029869/ ENSMUSG00000031304/ ENSMUSG00000090958/ ENSMUSG00000016496/ ENSMUSG00000022378/ ENSMUSG00000035042/ ENSMUSG00000035914/ ENSMUSG00000056153/ ENSMUSG00000037440/ ENSMUSG00000038855/ ENSMUSG00000018899/ ENSMUSG00000069255/ ENSMUSG00000031712/ ENSMUSG00000035385/ ENSMUSG00000025746	31
GO:0050863	regulation of T cell activation	32/815	301/21092	2.44×10^{-07}	1.21×10^{-05}	1.05×10^{-05}	ENSMUSG00000115338/ ENSMUSG00000048806/ ENSMUSG00000001123/ ENSMUSG00000067212/ ENSMUSG00000016206/ ENSMUSG00000057554/ ENSMUSG00000000791/ ENSMUSG00000021795/ ENSMUSG00000038037/ ENSMUSG00000039217/ ENSMUSG00000055447/ ENSMUSG00000020918/ ENSMUSG00000066877/ ENSMUSG00000024238/ ENSMUSG00000042333/ ENSMUSG00000040329/ ENSMUSG00000029869/ ENSMUSG00000032815/ ENSMUSG00000031304/ ENSMUSG00000090958/ ENSMUSG00000016496/ ENSMUSG00000022378/ ENSMUSG00000035042/ ENSMUSG00000035914/ ENSMUSG00000056153/ ENSMUSG00000037440/ ENSMUSG00000038855/ ENSMUSG00000018899/ ENSMUSG00000069255/ ENSMUSG00000031712/ ENSMUSG00000035385/ ENSMUSG00000025746	32
GO:0032479	regulation of type I interferon production	15/815	78/21092	2.53×10^{-07}	1.24×10^{-05}	1.08×10^{-05}	ENSMUSG00000025498/ ENSMUSG00000026104/ ENSMUSG00000017830/ ENSMUSG00000026896/ ENSMUSG00000040296/ ENSMUSG00000026946/ ENSMUSG00000029826/ ENSMUSG00000020707/ ENSMUSG00000031639/	15

							ENSMUSG00000032508/ ENSMUSG00000020115/ ENSMUSG00000079363/ ENSMUSG00000022126/ ENSMUSG00000018899/ ENSMUSG00000027995	
GO:0007159	leukocyte cell−cell adhesion	33/815	317/21092	2.59×10^{-07}	1.26×10^{-05}	1.09×10^{-05}	ENSMUSG00000115338/ ENSMUSG00000048806/ ENSMUSG00000001123/ ENSMUSG00000067212/ ENSMUSG00000016206/ ENSMUSG00000057554/ ENSMUSG00000000791/ ENSMUSG00000057058/ ENSMUSG00000021795/ ENSMUSG00000038037/ ENSMUSG00000003032/ ENSMUSG00000039217/ ENSMUSG00000055447/ ENSMUSG00000031207/ ENSMUSG00000038843/ ENSMUSG00000066877/ ENSMUSG00000042333/ ENSMUSG00000040329/ ENSMUSG00000029869/ ENSMUSG00000031304/ ENSMUSG00000090958/ ENSMUSG00000016496/ ENSMUSG00000022378/ ENSMUSG00000035042/ ENSMUSG00000035914/ ENSMUSG00000056153/ ENSMUSG00000037440/ ENSMUSG00000038855/ ENSMUSG00000018899/ ENSMUSG00000069255/ ENSMUSG00000031712/ ENSMUSG00000035385/ ENSMUSG00000025746	33
GO:0071219	cellular response to molecule of bacterial origin	30/815	273/21092	2.84×10^{-07}	1.36×10^{-05}	1.18×10^{-05}	ENSMUSG00000026104/ ENSMUSG00000069874/ ENSMUSG00000002602/ ENSMUSG00000045038/ ENSMUSG00000037580/ ENSMUSG00000037447/ ENSMUSG00000032508/ ENSMUSG00000039217/ ENSMUSG00000028270/ ENSMUSG00000020638/ ENSMUSG00000032501/ ENSMUSG00000016496/ ENSMUSG00000105096/ ENSMUSG00000035042/ ENSMUSG00000104713/ ENSMUSG00000025888/ ENSMUSG00000034855/ ENSMUSG00000025779/ ENSMUSG00000034610/ ENSMUSG00000060183/ ENSMUSG00000029417/ ENSMUSG00000022126/ ENSMUSG00000026981/ ENSMUSG00000027995/ ENSMUSG00000032372/ ENSMUSG00000024789/ ENSMUSG00000035385/ ENSMUSG00000020826/ ENSMUSG00000028599/ ENSMUSG00000025746	30
GO:1903708	positive regulation of hemopoiesis	25/815	204/21092	3.59×10^{-07}	1.71×10^{-05}	1.48×10^{-05}	ENSMUSG00000026104/ ENSMUSG00000115338/ ENSMUSG00000001123/ ENSMUSG00000016206/ ENSMUSG00000002602/ ENSMUSG00000027562/ ENSMUSG00000038037/ ENSMUSG00000039217/ ENSMUSG00000110206/ ENSMUSG00000004040/ ENSMUSG00000052684/ ENSMUSG00000040329/	25

							ENSMUSG00000032501/ ENSMUSG00000031304/ ENSMUSG00000035692/ ENSMUSG00000035042/ ENSMUSG00000031750/ ENSMUSG00000037440/ ENSMUSG00000038855/ ENSMUSG00000018899/ ENSMUSG00000031712/ ENSMUSG00000014599/ ENSMUSG00000021250/ ENSMUSG00000021127/ ENSMUSG00000025746	
GO:0042089	cytokine biosynthetic process	18/815	114/21092	3.75×10^{-07}	1.76×10^{-05}	1.53×10^{-05}	ENSMUSG00000025498/ ENSMUSG00000026946/ ENSMUSG00000002325/ ENSMUSG00000031639/ ENSMUSG00000003032/ ENSMUSG00000032508/ ENSMUSG00000039217/ ENSMUSG00000022514/ ENSMUSG00000040152/ ENSMUSG00000020115/ ENSMUSG00000038058/ ENSMUSG00000021453/ ENSMUSG00000015340/ ENSMUSG00000035914/ ENSMUSG00000018899/ ENSMUSG00000027995/ ENSMUSG00000038418/ ENSMUSG00000025746	18
GO:1902107	positive regulation of leukocyte differentiation	22/815	164/21092	3.77×10^{-07}	1.76×10^{-05}	1.53×10^{-05}	ENSMUSG00000115338/ ENSMUSG00000001123/ ENSMUSG00000016206/ ENSMUSG00000002602/ ENSMUSG00000027562/ ENSMUSG00000038037/ ENSMUSG00000039217/ ENSMUSG00000110206/ ENSMUSG00000052684/ ENSMUSG00000040329/ ENSMUSG00000032501/ ENSMUSG00000031304/ ENSMUSG00000035042/ ENSMUSG00000031750/ ENSMUSG00000037440/ ENSMUSG00000038855/ ENSMUSG00000018899/ ENSMUSG00000031712/ ENSMUSG00000014599/ ENSMUSG00000021250/ ENSMUSG00000021127/ ENSMUSG00000025746	22
GO:0032481	positive regulation of type I interferon production	12/815	51/21092	4.05×10^{-07}	1.87×10^{-05}	1.62×10^{-05}	ENSMUSG00000025498/ ENSMUSG00000026104/ ENSMUSG00000017830/ ENSMUSG00000026896/ ENSMUSG00000040296/ ENSMUSG00000029826/ ENSMUSG00000020707/ ENSMUSG00000031639/ ENSMUSG00000032508/ ENSMUSG00000020115/ ENSMUSG00000018899/ ENSMUSG00000027995	12
GO:0071706	tumor necrosis factor superfamily cytokine production	21/815	153/21092	4.69×10^{-07}	2.14×10^{-05}	1.86×10^{-05}	ENSMUSG00000026896/ ENSMUSG00000040296/ ENSMUSG00000001123/ ENSMUSG00000067212/ ENSMUSG00000031639/ ENSMUSG00000002602/ ENSMUSG00000037447/ ENSMUSG00000032508/ ENSMUSG00000039217/ ENSMUSG00000055447/ ENSMUSG00000039285/ ENSMUSG00000040152/	21

							ENSMUSG00000041736/ ENSMUSG00000038058/ ENSMUSG00000016496/ ENSMUSG00000030921/ ENSMUSG00000015340/ ENSMUSG00000025779/ ENSMUSG00000027995/ ENSMUSG00000024789/ ENSMUSG00000035385	
GO:0031331	positive regulation of cellular catabolic process	34/815	341/21092	4.73×10^{-07}	2.14×10^{-05}	1.86×10^{-05}	ENSMUSG00000069874/ ENSMUSG00000049502/ ENSMUSG00000030966/ ENSMUSG00000020464/ ENSMUSG00000002227/ ENSMUSG00000029826/ ENSMUSG00000034218/ ENSMUSG00000028793/ ENSMUSG00000026672/ ENSMUSG00000037075/ ENSMUSG00000049488/ ENSMUSG00000060450/ ENSMUSG00000022346/ ENSMUSG00000021036/ ENSMUSG00000028954/ ENSMUSG00000031207/ ENSMUSG00000004936/ ENSMUSG00000046879/ ENSMUSG00000037331/ ENSMUSG00000020115/ ENSMUSG00000025241/ ENSMUSG00000032540/ ENSMUSG00000038058/ ENSMUSG00000032501/ ENSMUSG00000037062/ ENSMUSG00000073599/ ENSMUSG00000000838/ ENSMUSG00000034724/ ENSMUSG00000047496/ ENSMUSG00000028211/ ENSMUSG00000024810/ ENSMUSG00000027995/ ENSMUSG00000021127/ ENSMUSG00000028599	34
GO:0071222	cellular response to lipopolysaccharide	29/815	265/21092	4.87×10^{-07}	2.19×10^{-05}	1.90×10^{-05}	ENSMUSG00000026104/ ENSMUSG00000069874/ ENSMUSG00000002602/ ENSMUSG00000045038/ ENSMUSG00000037580/ ENSMUSG00000037447/ ENSMUSG00000032508/ ENSMUSG00000039217/ ENSMUSG00000028270/ ENSMUSG00000020638/ ENSMUSG00000032501/ ENSMUSG00000016496/ ENSMUSG00000105096/ ENSMUSG00000035042/ ENSMUSG00000104713/ ENSMUSG00000025888/ ENSMUSG00000034855/ ENSMUSG00000025779/ ENSMUSG00000034610/ ENSMUSG00000060183/ ENSMUSG00000029417/ ENSMUSG00000022126/ ENSMUSG00000026981/ ENSMUSG00000032372/ ENSMUSG00000024789/ ENSMUSG00000035385/ ENSMUSG00000020826/ ENSMUSG00000028599/ ENSMUSG00000025746	29
GO:0001562	response to protozoan	10/815	35/21092	5.35×10^{-07}	2.36×10^{-05}	2.05×10^{-05}	ENSMUSG00000069874/ ENSMUSG00000028270/ ENSMUSG00000040253/ ENSMUSG00000105096/ ENSMUSG00000028268/ ENSMUSG00000054072/	10

							ENSMUSG00000104713/ ENSMUSG00000029298/ ENSMUSG00000040264/ ENSMUSG00000025746	
GO:0009896	positive regulation of catabolic process	38/815	407/21092	5.36×10^{-07}	2.36×10^{-05}	2.05×10^{-05}	ENSMUSG00000069874/ ENSMUSG00000049502/ ENSMUSG00000030966/ ENSMUSG00000020464/ ENSMUSG00000002227/ ENSMUSG00000029826/ ENSMUSG00000042350/ ENSMUSG00000034218/ ENSMUSG00000028793/ ENSMUSG00000045038/ ENSMUSG00000026672/ ENSMUSG00000037075/ ENSMUSG00000049488/ ENSMUSG00000060450/ ENSMUSG00000022346/ ENSMUSG00000021036/ ENSMUSG00000028954/ ENSMUSG00000031207/ ENSMUSG00000004936/ ENSMUSG00000046879/ ENSMUSG00000037331/ ENSMUSG00000020115/ ENSMUSG00000025241/ ENSMUSG00000032540/ ENSMUSG00000038058/ ENSMUSG00000032501/ ENSMUSG00000037062/ ENSMUSG00000073599/ ENSMUSG00000000838/ ENSMUSG00000034724/ ENSMUSG00000047496/ ENSMUSG00000030921/ ENSMUSG00000028211/ ENSMUSG00000024810/ ENSMUSG00000027995/ ENSMUSG00000038175/ ENSMUSG00000021127/ ENSMUSG00000028599	38
GO:1903039	positive regulation of leukocyte cell−cell adhesion	24/815	196/21092	6.22×10^{-07}	2.72×10^{-05}	2.36×10^{-05}	ENSMUSG00000115338/ ENSMUSG00000001123/ ENSMUSG00000067212/ ENSMUSG00000016206/ ENSMUSG00000057554/ ENSMUSG00000000791/ ENSMUSG00000057058/ ENSMUSG00000038037/ ENSMUSG00000039217/ ENSMUSG00000055447/ ENSMUSG00000066877/ ENSMUSG00000040329/ ENSMUSG00000029869/ ENSMUSG00000031304/ ENSMUSG00000016496/ ENSMUSG00000022378/ ENSMUSG00000035042/ ENSMUSG00000035914/ ENSMUSG00000037440/ ENSMUSG00000038855/ ENSMUSG00000018899/ ENSMUSG00000031712/ ENSMUSG00000035385/ ENSMUSG00000025746	24
GO:0032647	regulation of interferon−alpha production	9/815	28/21092	6.55×10^{-07}	2.83×10^{-05}	2.46×10^{-05}	ENSMUSG00000025498/ ENSMUSG00000026104/ ENSMUSG00000026896/ ENSMUSG00000040296/ ENSMUSG00000026946/ ENSMUSG00000029826/ ENSMUSG00000031639/ ENSMUSG00000020115/ ENSMUSG00000079363	9

GO:0050870	positive regulation of T cell activation	23/815	185/21092	8.12×10^{-07}	3.48×10^{-05}	3.02×10^{-05}	ENSMUSG00000115338/ ENSMUSG00000001123/ ENSMUSG00000067212/ ENSMUSG00000016206/ ENSMUSG00000057554/ ENSMUSG00000000791/ ENSMUSG00000038037/ ENSMUSG00000039217/ ENSMUSG00000055447/ ENSMUSG00000066877/ ENSMUSG00000040329/ ENSMUSG00000029869/ ENSMUSG00000031304/ ENSMUSG00000016496/ ENSMUSG00000022378/ ENSMUSG00000035042/ ENSMUSG00000035914/ ENSMUSG00000037440/ ENSMUSG00000038855/ ENSMUSG00000018899/ ENSMUSG00000031712/ ENSMUSG00000035385/ ENSMUSG00000025746	23
GO:0042107	cytokine metabolic process	18/815	120/21092	8.20×10^{-07}	3.48×10^{-05}	3.02×10^{-05}	ENSMUSG00000025498/ ENSMUSG00000026946/ ENSMUSG00000002325/ ENSMUSG00000031639/ ENSMUSG00000003032/ ENSMUSG00000032508/ ENSMUSG00000039217/ ENSMUSG00000022514/ ENSMUSG00000040152/ ENSMUSG00000020115/ ENSMUSG00000038058/ ENSMUSG00000021453/ ENSMUSG00000015340/ ENSMUSG00000035914/ ENSMUSG00000018899/ ENSMUSG00000027995/ ENSMUSG00000038418/ ENSMUSG00000025746	18
GO:0032635	interleukin−6 production	20/815	147/21092	1.01×10^{-06}	4.21×10^{-05}	3.65×10^{-05}	ENSMUSG00000026896/ ENSMUSG00000040296/ ENSMUSG00000001123/ ENSMUSG00000036908/ ENSMUSG00000031639/ ENSMUSG00000037447/ ENSMUSG00000032508/ ENSMUSG00000039217/ ENSMUSG00000055447/ ENSMUSG00000022514/ ENSMUSG00000039285/ ENSMUSG00000038058/ ENSMUSG00000055148/ ENSMUSG00000030921/ ENSMUSG00000034610/ ENSMUSG00000024810/ ENSMUSG00000026981/ ENSMUSG00000027995/ ENSMUSG00000020826/ ENSMUSG00000025746	20
GO:0050777	negative regulation of immune response	20/815	147/21092	1.01×10^{-06}	4.21×10^{-05}	3.65×10^{-05}	ENSMUSG00000017830/ ENSMUSG00000034422/ ENSMUSG00000037321/ ENSMUSG00000024339/ ENSMUSG00000027639/ ENSMUSG00000042726/ ENSMUSG00000026946/ ENSMUSG00000027951/ ENSMUSG00000048806/ ENSMUSG00000001123/ ENSMUSG00000067212/ ENSMUSG00000030157/ ENSMUSG00000023224/ ENSMUSG00000000127/ ENSMUSG00000074151/	20

							ENSMUSG00000045827/ ENSMUSG00000024810/ ENSMUSG00000022126/ ENSMUSG00000018899/ ENSMUSG00000069255	
GO:0051249	regulation of lymphocyte activation	38/815	421/21092	1.23×10^{-06}	5.09×10^{-05}	4.42×10^{-05}	ENSMUSG00000115338/ ENSMUSG00000048806/ ENSMUSG00000027950/ ENSMUSG00000001123/ ENSMUSG00000067212/ ENSMUSG00000016206/ ENSMUSG00000034218/ ENSMUSG00000057554/ ENSMUSG00000000791/ ENSMUSG00000002602/ ENSMUSG00000021795/ ENSMUSG00000038037/ ENSMUSG00000032508/ ENSMUSG00000039217/ ENSMUSG00000055447/ ENSMUSG00000020918/ ENSMUSG00000110206/ ENSMUSG00000066877/ ENSMUSG00000024238/ ENSMUSG00000042333/ ENSMUSG00000040329/ ENSMUSG00000029869/ ENSMUSG00000032815/ ENSMUSG00000031304/ ENSMUSG00000090958/ ENSMUSG00000016496/ ENSMUSG00000022378/ ENSMUSG00000035042/ ENSMUSG00000035914/ ENSMUSG00000056153/ ENSMUSG00000037440/ ENSMUSG00000038855/ ENSMUSG00000018899/ ENSMUSG00000069255/ ENSMUSG00000031712/ ENSMUSG00000035385/ ENSMUSG00000021127/ ENSMUSG00000025746	38
GO:0002230	positive regulation of defense response to virus by host	9/815	30/21092	1.27×10^{-06}	5.16×10^{-05}	4.48×10^{-05}	ENSMUSG00000026104/ ENSMUSG00000049502/ ENSMUSG00000022906/ ENSMUSG00000040296/ ENSMUSG00000036986/ ENSMUSG00000072244/ ENSMUSG00000000791/ ENSMUSG00000037860/ ENSMUSG00000035042	9
GO:0052548	regulation of endopeptidase activity	33/815	340/21092	1.27×10^{-06}	5.16×10^{-05}	4.48×10^{-05}	ENSMUSG00000024338/ ENSMUSG00000026104/ ENSMUSG00000096727/ ENSMUSG00000024066/ ENSMUSG00000064215/ ENSMUSG00000036986/ ENSMUSG00000042842/ ENSMUSG00000022216/ ENSMUSG00000079197/ ENSMUSG00000078945/ ENSMUSG00000021208/ ENSMUSG00000021403/ ENSMUSG00000023224/ ENSMUSG00000003032/ ENSMUSG00000037860/ ENSMUSG00000022346/ ENSMUSG00000000915/ ENSMUSG00000040152/ ENSMUSG00000045827/ ENSMUSG00000004040/ ENSMUSG00000030654/ ENSMUSG00000038058/ ENSMUSG00000039304/ ENSMUSG00000025887/ ENSMUSG00000025888/ ENSMUSG00000004231/ ENSMUSG00000034485/	33

							ENSMUSG00000033538/ ENSMUSG00000028289/ ENSMUSG00000024789/ ENSMUSG00000026315/ ENSMUSG00000025746/ ENSMUSG00000066363	
GO:0032760	positive regulation of tumor necrosis factor production	15/815	88/21092	1.29 × 10^{−06}	5.18 × 10^{−05}	4.50 × 10^{−05}	ENSMUSG00000026896/ ENSMUSG00000040296/ ENSMUSG00000001123/ ENSMUSG00000067212/ ENSMUSG00000031639/ ENSMUSG00000037447/ ENSMUSG00000032508/ ENSMUSG00000039217/ ENSMUSG00000040152/ ENSMUSG00000038058/ ENSMUSG00000015340/ ENSMUSG00000025779/ ENSMUSG00000027995/ ENSMUSG00000024789/ ENSMUSG00000035385	15
GO:0070661	leukocyte proliferation	32/815	325/21092	1.34 × 10^{−06}	5.37 × 10^{−05}	4.67 × 10^{−05}	ENSMUSG00000031897/ ENSMUSG00000115338/ ENSMUSG00000048806/ ENSMUSG00000027950/ ENSMUSG00000001123/ ENSMUSG00000067212/ ENSMUSG00000016206/ ENSMUSG00000070904/ ENSMUSG00000034218/ ENSMUSG00000000791/ ENSMUSG00000021795/ ENSMUSG00000032508/ ENSMUSG00000039217/ ENSMUSG00000031207/ ENSMUSG00000039285/ ENSMUSG00000110206/ ENSMUSG00000066877/ ENSMUSG00000020115/ ENSMUSG00000042333/ ENSMUSG00000040329/ ENSMUSG00000029869/ ENSMUSG00000090958/ ENSMUSG00000016496/ ENSMUSG00000035042/ ENSMUSG00000035914/ ENSMUSG00000024810/ ENSMUSG00000022206/ ENSMUSG00000018899/ ENSMUSG00000031712/ ENSMUSG00000014599/ ENSMUSG00000034165/ ENSMUSG00000025746	32
GO:0032640	tumor necrosis factor production	20/815	150/21092	1.39 × 10^{−06}	5.48 × 10^{−05}	4.76 × 10^{−05}	ENSMUSG00000026896/ ENSMUSG00000040296/ ENSMUSG00000001123/ ENSMUSG00000067212/ ENSMUSG00000031639/ ENSMUSG00000002602/ ENSMUSG00000037447/ ENSMUSG00000032508/ ENSMUSG00000039217/ ENSMUSG00000055447/ ENSMUSG00000039285/ ENSMUSG00000040152/ ENSMUSG00000041736/ ENSMUSG00000038058/ ENSMUSG00000030921/ ENSMUSG00000015340/ ENSMUSG00000025779/ ENSMUSG00000027995/ ENSMUSG00000024789/ ENSMUSG00000035385	20
GO:1903555	regulation of tumor necrosis factor superfamily cytokine production	20/815	150/21092	1.39 × 10^{−06}	5.48 × 10^{−05}	4.76 × 10^{−05}	ENSMUSG00000026896/ ENSMUSG00000040296/ ENSMUSG00000001123/ ENSMUSG00000067212/ ENSMUSG00000031639/ ENSMUSG00000002602/	20

							ENSMUSG00000037447/ ENSMUSG00000032508/ ENSMUSG00000039217/ ENSMUSG00000055447/ ENSMUSG00000040152/ ENSMUSG00000041736/ ENSMUSG00000038058/ ENSMUSG00000016496/ ENSMUSG00000030921/ ENSMUSG00000015340/ ENSMUSG00000025779/ ENSMUSG00000027995/ ENSMUSG00000024789/ ENSMUSG00000035385	
GO:1903557	positive regulation of tumor necrosis factor superfamily cytokine production	15/815	89/21092	1.49×10^{-06}	5.82×10^{-05}	5.05×10^{-05}	ENSMUSG00000026896/ ENSMUSG00000040296/ ENSMUSG00000001123/ ENSMUSG00000067212/ ENSMUSG00000031639/ ENSMUSG00000037447/ ENSMUSG00000032508/ ENSMUSG00000039217/ ENSMUSG00000040152/ ENSMUSG00000038058/ ENSMUSG00000015340/ ENSMUSG00000025779/ ENSMUSG00000027995/ ENSMUSG00000024789/ ENSMUSG00000035385	15
GO:0071216	cellular response to biotic stimulus	30/815	296/21092	1.58×10^{-06}	6.10×10^{-05}	5.30×10^{-05}	ENSMUSG00000026104/ ENSMUSG00000069874/ ENSMUSG00000002602/ ENSMUSG00000045038/ ENSMUSG00000037580/ ENSMUSG00000037447/ ENSMUSG00000032508/ ENSMUSG00000039217/ ENSMUSG00000028270/ ENSMUSG00000020638/ ENSMUSG00000032501/ ENSMUSG00000016496/ ENSMUSG00000105096/ ENSMUSG00000035042/ ENSMUSG00000104713/ ENSMUSG00000025888/ ENSMUSG00000034855/ ENSMUSG00000025779/ ENSMUSG00000034610/ ENSMUSG00000060183/ ENSMUSG00000029417/ ENSMUSG00000022126/ ENSMUSG00000026981/ ENSMUSG00000027995/ ENSMUSG00000032372/ ENSMUSG00000024789/ ENSMUSG00000035385/ ENSMUSG00000020826/ ENSMUSG00000028599/ ENSMUSG00000025746	30
GO:0002720	positive regulation of cytokine production involved in immune response	11/815	48/21092	1.64×10^{-06}	6.27×10^{-05}	5.45×10^{-05}	ENSMUSG00000040296/ ENSMUSG00000060802/ ENSMUSG00000072244/ ENSMUSG00000037447/ ENSMUSG00000039217/ ENSMUSG00000042333/ ENSMUSG00000031948/ ENSMUSG00000020641/ ENSMUSG00000027995/ ENSMUSG00000008734/ ENSMUSG00000025746	11
GO:0019884	antigen processing and presentation of exogenous antigen	9/815	31/21092	1.72×10^{-06}	6.50×10^{-05}	5.65×10^{-05}	ENSMUSG00000037321/ ENSMUSG00000024339/ ENSMUSG00000061232/ ENSMUSG00000024308/ ENSMUSG00000060802/ ENSMUSG00000022216/ ENSMUSG00000036908/ ENSMUSG00000016206/ ENSMUSG00000079197	9

GO:0060330	regulation of response to interferon−gamma	7/815	17/21092	1.74 × 10−06	6.50 × 10−05	5.65 × 10−05	ENSMUSG00000069874/ ENSMUSG00000034422/ ENSMUSG00000022906/ ENSMUSG00000038037/ ENSMUSG00000074151/ ENSMUSG00000046879/ ENSMUSG00000030895	7
GO:0060334	regulation of interferon−gamma−mediated signaling pathway	7/815	17/21092	1.74 × 10−06	6.50 × 10−05	5.65 × 10−05	ENSMUSG00000069874/ ENSMUSG00000034422/ ENSMUSG00000022906/ ENSMUSG00000038037/ ENSMUSG00000074151/ ENSMUSG00000046879/ ENSMUSG00000030895	7
GO:1903706	regulation of hemopoiesis	36/815	398/21092	2.23 × 10−06	8.28 × 10−05	7.19 × 10−05	ENSMUSG00000026104/ ENSMUSG00000024079/ ENSMUSG00000115338/ ENSMUSG00000060802/ ENSMUSG00000048806/ ENSMUSG00000001123/ ENSMUSG00000016206/ ENSMUSG00000027204/ ENSMUSG00000030157/ ENSMUSG00000014773/ ENSMUSG00000002602/ ENSMUSG00000027562/ ENSMUSG00000038037/ ENSMUSG00000032698/ ENSMUSG00000039217/ ENSMUSG00000022346/ ENSMUSG00000020918/ ENSMUSG00000110206/ ENSMUSG00000024238/ ENSMUSG00000004040/ ENSMUSG00000052684/ ENSMUSG00000040329/ ENSMUSG00000032501/ ENSMUSG00000032815/ ENSMUSG00000031304/ ENSMUSG00000035692/ ENSMUSG00000035042/ ENSMUSG00000031750/ ENSMUSG00000037440/ ENSMUSG00000038855/ ENSMUSG00000018899/ ENSMUSG00000031712/ ENSMUSG00000014599/ ENSMUSG00000021250/ ENSMUSG00000021127/ ENSMUSG00000025746	36
GO:0032728	positive regulation of interferon−beta production	9/815	32/21092	2.31 × 10−06	8.53 × 10−05	7.41 × 10−05	ENSMUSG00000025498/ ENSMUSG00000026896/ ENSMUSG00000040296/ ENSMUSG00000029826/ ENSMUSG00000020707/ ENSMUSG00000031639/ ENSMUSG00000020115/ ENSMUSG00000018899/ ENSMUSG00000027995	9
GO:0035457	cellular response to interferon−alpha	6/815	12/21092	2.47 × 10−06	9.05 × 10−05	7.86 × 10−05	ENSMUSG00000045932/ ENSMUSG00000002602/ ENSMUSG00000022346/ ENSMUSG00000073489/ ENSMUSG00000034459/ ENSMUSG00000074896	6
GO:0043902	positive regulation of multi−organism process	21/815	171/21092	2.95 × 10−06	0.0001	9.30 × 10−05	ENSMUSG00000017830/ ENSMUSG00000030966/ ENSMUSG00000027951/ ENSMUSG00000029826/ ENSMUSG00000001123/ ENSMUSG00000026672/ ENSMUSG00000039217/ ENSMUSG00000037921/ ENSMUSG00000038467/ ENSMUSG00000037331/ ENSMUSG00000020115/ ENSMUSG00000055204/	21

							ENSMUSG00000031813/ ENSMUSG00000052684/ ENSMUSG00000038058/ ENSMUSG00000000838/ ENSMUSG00000030921/ ENSMUSG00000025779/ ENSMUSG00000022126/ ENSMUSG00000027995/ ENSMUSG00000020826	
GO:1902105	regulation of leukocyte differentiation	29/815	290/21092	3.07×10^{-06}	0.0001	9.61×10^{-05}	ENSMUSG00000115338/ ENSMUSG00000048806/ ENSMUSG00000001123/ ENSMUSG00000016206/ ENSMUSG00000027204/ ENSMUSG00000030157/ ENSMUSG00000002602/ ENSMUSG00000027562/ ENSMUSG00000038037/ ENSMUSG00000039217/ ENSMUSG00000022346/ ENSMUSG00000020918/ ENSMUSG00000110206/ ENSMUSG00000024238/ ENSMUSG00000052684/ ENSMUSG00000040329/ ENSMUSG00000032501/ ENSMUSG00000032815/ ENSMUSG00000031304/ ENSMUSG00000035042/ ENSMUSG00000031750/ ENSMUSG00000037440/ ENSMUSG00000038855/ ENSMUSG00000018899/ ENSMUSG00000031712/ ENSMUSG00000014599/ ENSMUSG00000021250/ ENSMUSG00000021127/ ENSMUSG00000025746	29
GO:0032103	positive regulation of response to external stimulus	29/815	291/21092	3.29×10^{-06}	0.0001	0.0001	ENSMUSG00000017830/ ENSMUSG00000029826/ ENSMUSG00000001123/ ENSMUSG00000031639/ ENSMUSG00000056220/ ENSMUSG00000026672/ ENSMUSG00000032508/ ENSMUSG00000055447/ ENSMUSG00000037921/ ENSMUSG00000040152/ ENSMUSG00000020115/ ENSMUSG00000055204/ ENSMUSG00000031948/ ENSMUSG00000038058/ ENSMUSG00000041187/ ENSMUSG00000035042/ ENSMUSG00000034855/ ENSMUSG00000002897/ ENSMUSG00000025779/ ENSMUSG00000024810/ ENSMUSG00000105504/ ENSMUSG00000022126/ ENSMUSG00000027995/ ENSMUSG00000022676/ ENSMUSG00000014599/ ENSMUSG00000008734/ ENSMUSG00000024789/ ENSMUSG00000035385/ ENSMUSG00000025746	29
GO:0043123	positive regulation of I−kappaB kinase/NF−kappaB signaling	16/815	107/21092	3.46×10^{-06}	0.0001	0.0001	ENSMUSG00000029826/ ENSMUSG00000000275/ ENSMUSG00000047098/ ENSMUSG00000031639/ ENSMUSG00000045038/ ENSMUSG00000032508/ ENSMUSG00000055204/ ENSMUSG00000027466/ ENSMUSG00000038058/ ENSMUSG00000078923/ ENSMUSG00000025888/ ENSMUSG00000026637/	16

							ENSMUSG00000035186/ ENSMUSG00000028028/ ENSMUSG00000026981/ ENSMUSG00000008734	
GO:0007249	I−kappaB kinase/NF−kappaB signaling	22/815	187/21092	3.53 × 10^{-06}	0.0001	0.0001	ENSMUSG00000026104/ ENSMUSG00000029826/ ENSMUSG00000000275/ ENSMUSG00000047098/ ENSMUSG00000031639/ ENSMUSG00000045038/ ENSMUSG00000026672/ ENSMUSG00000032508/ ENSMUSG00000039285/ ENSMUSG00000055204/ ENSMUSG00000027466/ ENSMUSG00000038058/ ENSMUSG00000078923/ ENSMUSG00000025888/ ENSMUSG00000026637/ ENSMUSG00000035186/ ENSMUSG00000018899/ ENSMUSG00000028028/ ENSMUSG00000026981/ ENSMUSG00000027995/ ENSMUSG00000008734/ ENSMUSG00000040026	22
GO:0050707	regulation of cytokine secretion	23/815	203/21092	4.07 × 10^{-06}	0.0001	0.0001	ENSMUSG00000026896/ ENSMUSG00000040296/ ENSMUSG00000036986/ ENSMUSG00000001123/ ENSMUSG00000036908/ ENSMUSG00000072244/ ENSMUSG00000038037/ ENSMUSG00000037447/ ENSMUSG00000037860/ ENSMUSG00000022575/ ENSMUSG00000022514/ ENSMUSG00000031154/ ENSMUSG00000042333/ ENSMUSG00000031948/ ENSMUSG00000090958/ ENSMUSG00000016496/ ENSMUSG00000025888/ ENSMUSG00000035914/ ENSMUSG00000002897/ ENSMUSG00000024810/ ENSMUSG00000033538/ ENSMUSG00000027995/ ENSMUSG00000025746	23
GO:0051251	positive regulation of lymphocyte activation	27/815	263/21092	4.08 × 10^{-06}	0.0001	0.0001	ENSMUSG00000115338/ ENSMUSG00000027950/ ENSMUSG00000001123/ ENSMUSG00000067212/ ENSMUSG00000016206/ ENSMUSG00000057554/ ENSMUSG00000000791/ ENSMUSG00000002602/ ENSMUSG00000038037/ ENSMUSG00000032508/ ENSMUSG00000039217/ ENSMUSG00000055447/ ENSMUSG00000110206/ ENSMUSG00000066877/ ENSMUSG00000040329/ ENSMUSG00000029869/ ENSMUSG00000031304/ ENSMUSG00000016496/ ENSMUSG00000022378/ ENSMUSG00000035042/ ENSMUSG00000035914/ ENSMUSG00000037440/ ENSMUSG00000038855/ ENSMUSG00000018899/ ENSMUSG00000031712/ ENSMUSG00000035385/ ENSMUSG00000025746	27

GO:0032609	interferon−gamma production	17/815	121/21092	4.14 × 10−06	0.0001	0.0001	ENSMUSG00000001123/ ENSMUSG00000016206/ ENSMUSG00000031639/ ENSMUSG00000028793/ ENSMUSG00000000791/ ENSMUSG00000002602/ ENSMUSG00000060550/ ENSMUSG00000037447/ ENSMUSG00000039217/ ENSMUSG00000055447/ ENSMUSG00000039285/ ENSMUSG00000035692/ ENSMUSG00000016496/ ENSMUSG00000022378/ ENSMUSG00000021453/ ENSMUSG00000035914/ ENSMUSG00000024810	17
GO:0032680	regulation of tumor necrosis factor production	19/815	148/21092	4.49 × 10−06	0.0002	0.0001	ENSMUSG00000026896/ ENSMUSG00000040296/ ENSMUSG00000001123/ ENSMUSG00000067212/ ENSMUSG00000031639/ ENSMUSG00000002602/ ENSMUSG00000037447/ ENSMUSG00000032508/ ENSMUSG00000039217/ ENSMUSG00000055447/ ENSMUSG00000040152/ ENSMUSG00000041736/ ENSMUSG00000038058/ ENSMUSG00000030921/ ENSMUSG00000015340/ ENSMUSG00000025779/ ENSMUSG00000027995/ ENSMUSG00000024789/ ENSMUSG00000035385	19
GO:0032608	interferon−beta production	11/815	53/21092	4.63 × 10−06	0.0002	0.0001	ENSMUSG00000025498/ ENSMUSG00000026896/ ENSMUSG00000040296/ ENSMUSG00000026946/ ENSMUSG00000029826/ ENSMUSG00000020707/ ENSMUSG00000031639/ ENSMUSG00000043279/ ENSMUSG00000020115/ ENSMUSG00000018899/ ENSMUSG00000027995	11
GO:0022409	positive regulation of cell−cell adhesion	25/815	236/21092	5.29 × 10−06	0.0002	0.0002	ENSMUSG00000115338/ ENSMUSG00000001123/ ENSMUSG00000067212/ ENSMUSG00000016206/ ENSMUSG00000057554/ ENSMUSG00000000791/ ENSMUSG00000057058/ ENSMUSG00000021360/ ENSMUSG00000038037/ ENSMUSG00000039217/ ENSMUSG00000055447/ ENSMUSG00000066877/ ENSMUSG00000040329/ ENSMUSG00000029869/ ENSMUSG00000031304/ ENSMUSG00000016496/ ENSMUSG00000022378/ ENSMUSG00000035042/ ENSMUSG00000035914/ ENSMUSG00000037440/ ENSMUSG00000038855/ ENSMUSG00000018899/ ENSMUSG00000031712/ ENSMUSG00000035385/ ENSMUSG00000025746	25

GO:0002718	regulation of cytokine production involved in immune response	13/815	75/21092	5.37×10^{-06}	0.0002	0.0002	ENSMUSG00000040296/ ENSMUSG00000060802/ ENSMUSG00000048806/ ENSMUSG00000031639/ ENSMUSG00000072244/ ENSMUSG00000037447/ ENSMUSG00000039217/ ENSMUSG00000042333/ ENSMUSG00000031948/ ENSMUSG00000020641/ ENSMUSG00000027995/ ENSMUSG00000008734/ ENSMUSG00000025746	13
GO:0052547	regulation of peptidase activity	36/815	414/21092	5.46×10^{-06}	0.0002	0.0002	ENSMUSG00000024338/ ENSMUSG00000026104/ ENSMUSG00000096727/ ENSMUSG00000024066/ ENSMUSG00000064215/ ENSMUSG00000036986/ ENSMUSG00000042842/ ENSMUSG00000022216/ ENSMUSG00000079197/ ENSMUSG00000078945/ ENSMUSG00000021208/ ENSMUSG00000021403/ ENSMUSG00000023224/ ENSMUSG00000003032/ ENSMUSG00000037860/ ENSMUSG00000022346/ ENSMUSG00000000915/ ENSMUSG00000017723/ ENSMUSG00000040152/ ENSMUSG00000045827/ ENSMUSG00000004040/ ENSMUSG00000030654/ ENSMUSG00000031948/ ENSMUSG00000038058/ ENSMUSG00000039304/ ENSMUSG00000025887/ ENSMUSG00000025888/ ENSMUSG00000004231/ ENSMUSG00000034485/ ENSMUSG00000033538/ ENSMUSG00000028289/ ENSMUSG00000024789/ ENSMUSG00000026315/ ENSMUSG00000046186/ ENSMUSG00000025746/ ENSMUSG00000066363	36
GO:0032069	regulation of nuclease activity	8/815	27/21092	5.54×10^{-06}	0.0002	0.0002	ENSMUSG00000032661/ ENSMUSG00000029605/ ENSMUSG00000001166/ ENSMUSG00000032690/ ENSMUSG00000041827/ ENSMUSG00000066861/ ENSMUSG00000029561/ ENSMUSG00000052776	8
GO:0042116	macrophage activation	13/815	76/21092	6.25×10^{-06}	0.0002	0.0002	ENSMUSG00000048806/ ENSMUSG00000001123/ ENSMUSG00000034218/ ENSMUSG00000031639/ ENSMUSG00000020572/ ENSMUSG00000045038/ ENSMUSG00000040152/ ENSMUSG00000031948/ ENSMUSG00000052684/ ENSMUSG00000025888/ ENSMUSG00000070056/ ENSMUSG00000024810/ ENSMUSG00000027995	13
GO:0002274	myeloid leukocyte activation	22/815	195/21092	7.01×10^{-06}	0.0002	0.0002	ENSMUSG00000048806/ ENSMUSG00000001123/ ENSMUSG00000034218/ ENSMUSG00000031639/ ENSMUSG00000020572/ ENSMUSG00000045038/	22

							ENSMUSG00000000127/ ENSMUSG00000039217/ ENSMUSG00000025178/ ENSMUSG00000040152/ ENSMUSG00000110206/ ENSMUSG00000004508/ ENSMUSG00000031948/ ENSMUSG00000052684/ ENSMUSG00000035042/ ENSMUSG00000025888/ ENSMUSG00000070056/ ENSMUSG00000026797/ ENSMUSG00000024810/ ENSMUSG00000035186/ ENSMUSG00000027995/ ENSMUSG00000062300	
GO:0045351	type I interferon biosynthetic process	6/815	14/21092	7.52×10^{-06}	0.0002	0.0002	ENSMUSG00000025498/ ENSMUSG00000026946/ ENSMUSG00000002325/ ENSMUSG00000031639/ ENSMUSG00000032508/ ENSMUSG00000020115	6
GO:0045785	positive regulation of cell adhesion	35/815	405/21092	8.41×10^{-06}	0.0003	0.0002	ENSMUSG00000115338/ ENSMUSG00000001123/ ENSMUSG00000067212/ ENSMUSG00000016206/ ENSMUSG00000034218/ ENSMUSG00000057554/ ENSMUSG00000000791/ ENSMUSG00000045038/ ENSMUSG00000057058/ ENSMUSG00000033088/ ENSMUSG00000021360/ ENSMUSG00000038037/ ENSMUSG00000035258/ ENSMUSG00000022505/ ENSMUSG00000039217/ ENSMUSG00000055447/ ENSMUSG00000021365/ ENSMUSG00000040152/ ENSMUSG00000066877/ ENSMUSG00000040329/ ENSMUSG00000041187/ ENSMUSG00000029869/ ENSMUSG00000031304/ ENSMUSG00000016496/ ENSMUSG00000022378/ ENSMUSG00000035042/ ENSMUSG00000035914/ ENSMUSG00000037440/ ENSMUSG00000038855/ ENSMUSG00000018899/ ENSMUSG00000031712/ ENSMUSG00000014599/ ENSMUSG00000024789/ ENSMUSG00000035385/ ENSMUSG00000025746	35
GO:0032755	positive regulation of interleukin−6 production	14/815	90/21092	8.75×10^{-06}	0.0003	0.0002	ENSMUSG00000026896/ ENSMUSG00000040296/ ENSMUSG00000001123/ ENSMUSG00000036908/ ENSMUSG00000031639/ ENSMUSG00000037447/ ENSMUSG00000032508/ ENSMUSG00000022514/ ENSMUSG00000038058/ ENSMUSG00000034610/ ENSMUSG00000024810/ ENSMUSG00000026981/ ENSMUSG00000027995/ ENSMUSG00000025746	14
GO:0030099	myeloid cell differentiation	34/815	392/21092	1.04×10^{-05}	0.0003	0.0003	ENSMUSG00000026104/ ENSMUSG00000027951/ ENSMUSG00000036986/ ENSMUSG00000060802/ ENSMUSG00000048806/ ENSMUSG00000027204/	34

							ENSMUSG00000030157/ ENSMUSG00000014773/ ENSMUSG00000027562/ ENSMUSG00000032698/ ENSMUSG00000022346/ ENSMUSG00000031540/ ENSMUSG00000004508/ ENSMUSG00000004040/ ENSMUSG00000052684/ ENSMUSG00000032501/ ENSMUSG00000035692/ ENSMUSG00000035042/ ENSMUSG00000055148/ ENSMUSG00000015133/ ENSMUSG00000070056/ ENSMUSG00000031750/ ENSMUSG00000068758/ ENSMUSG00000035186/ ENSMUSG00000038855/ ENSMUSG00000027995/ ENSMUSG00000031543/ ENSMUSG00000038301/ ENSMUSG00000014599/ ENSMUSG00000021250/ ENSMUSG00000024789/ ENSMUSG00000028382/ ENSMUSG00000021127/ ENSMUSG00000046186	
GO:0032943	mononuclear cell proliferation	29/815	309/21092	1.06×10^{-05}	0.0003	0.0003	ENSMUSG00000031897/ ENSMUSG00000115338/ ENSMUSG00000048806/ ENSMUSG00000027950/ ENSMUSG00000001123/ ENSMUSG00000067212/ ENSMUSG00000016206/ ENSMUSG00000070904/ ENSMUSG00000034218/ ENSMUSG00000000791/ ENSMUSG00000021795/ ENSMUSG00000032508/ ENSMUSG00000039217/ ENSMUSG00000031207/ ENSMUSG00000039285/ ENSMUSG00000066877/ ENSMUSG00000020115/ ENSMUSG00000042333/ ENSMUSG00000040329/ ENSMUSG00000029869/ ENSMUSG00000090958/ ENSMUSG00000016496/ ENSMUSG00000035042/ ENSMUSG00000035914/ ENSMUSG00000018899/ ENSMUSG00000031712/ ENSMUSG00000014599/ ENSMUSG00000034165/ ENSMUSG00000025746	29
GO:0045862	positive regulation of proteolysis	30/815	327/21092	1.17×10^{-05}	0.0004	0.0003	ENSMUSG00000026104/ ENSMUSG00000024066/ ENSMUSG00000064215/ ENSMUSG00000036986/ ENSMUSG00000022216/ ENSMUSG00000079197/ ENSMUSG00000021208/ ENSMUSG00000028793/ ENSMUSG00000016495/ ENSMUSG00000037075/ ENSMUSG00000049488/ ENSMUSG00000060450/ ENSMUSG00000037860/ ENSMUSG00000022346/ ENSMUSG00000028954/ ENSMUSG00000000915/ ENSMUSG00000004040/ ENSMUSG00000031948/ ENSMUSG00000038058/ ENSMUSG00000039304/ ENSMUSG00000032501/	30

							ENSMUSG00000073599/ ENSMUSG00000025887/ ENSMUSG00000000838/ ENSMUSG00000025888/ ENSMUSG00000024810/ ENSMUSG00000034485/ ENSMUSG00000033538/ ENSMUSG00000024789/ ENSMUSG00000028599	
GO:0032722	positive regulation of chemokine production	11/815	59/21092	1.38×10^{-05}	0.0004	0.0004	ENSMUSG00000024079/ ENSMUSG00000001123/ ENSMUSG00000031639/ ENSMUSG00000032508/ ENSMUSG00000039217/ ENSMUSG00000040329/ ENSMUSG00000035042/ ENSMUSG00000024810/ ENSMUSG00000027995/ ENSMUSG00000038418/ ENSMUSG00000025746	11
GO:0006956	complement activation	10/815	49/21092	1.47×10^{-05}	0.0005	0.0004	ENSMUSG00000024371/ ENSMUSG00000023224/ ENSMUSG00000055172/ ENSMUSG00000079343/ ENSMUSG00000026405/ ENSMUSG00000073418/ ENSMUSG00000015451/ ENSMUSG00000026365/ ENSMUSG00000090231/ ENSMUSG00000038521	10
GO:0007179	transforming growth factor beta receptor signaling pathway	19/815	162/21092	1.68×10^{-05}	0.0005	0.0004	ENSMUSG00000036986/ ENSMUSG00000027204/ ENSMUSG00000021360/ ENSMUSG00000038400/ ENSMUSG00000040152/ ENSMUSG00000024238/ ENSMUSG00000021796/ ENSMUSG00000052684/ ENSMUSG00000090958/ ENSMUSG00000029860/ ENSMUSG00000021065/ ENSMUSG00000021754/ ENSMUSG00000001870/ ENSMUSG00000092035/ ENSMUSG00000069255/ ENSMUSG00000021250/ ENSMUSG00000035385/ ENSMUSG00000046186/ ENSMUSG00000027660	19
GO:0050715	positive regulation of cytokine secretion	18/815	148/21092	1.69×10^{-05}	0.0005	0.0004	ENSMUSG00000026896/ ENSMUSG00000040296/ ENSMUSG00000001123/ ENSMUSG00000036908/ ENSMUSG00000072244/ ENSMUSG00000037447/ ENSMUSG00000037860/ ENSMUSG00000022575/ ENSMUSG00000022514/ ENSMUSG00000042333/ ENSMUSG00000031948/ ENSMUSG00000016496/ ENSMUSG00000025888/ ENSMUSG00000035914/ ENSMUSG00000002897/ ENSMUSG00000024810/ ENSMUSG00000033538/ ENSMUSG00000027995	18
GO:0032727	positive regulation of interferon−alpha production	7/815	23/21092	1.79×10^{-05}	0.0005	0.0005	ENSMUSG00000025498/ ENSMUSG00000026104/ ENSMUSG00000026896/ ENSMUSG00000040296/ ENSMUSG00000029826/ ENSMUSG00000031639/ ENSMUSG00000020115	7

GO:0070269	pyroptosis	7/815	23/21092	1.79×10^{-05}	0.0005	0.0005	ENSMUSG00000078942/ ENSMUSG00000078945/ ENSMUSG00000071203/ ENSMUSG00000037860/ ENSMUSG00000022575/ ENSMUSG00000025888/ ENSMUSG00000033538	7
GO:0002224	toll−like receptor signaling pathway	14/815	96/21092	1.87×10^{-05}	0.0006	0.0005	ENSMUSG00000025498/ ENSMUSG00000001123/ ENSMUSG00000036908/ ENSMUSG00000031639/ ENSMUSG00000045038/ ENSMUSG00000073643/ ENSMUSG00000032508/ ENSMUSG00000030921/ ENSMUSG00000070056/ ENSMUSG00000020641/ ENSMUSG00000025779/ ENSMUSG00000022126/ ENSMUSG00000018899/ ENSMUSG00000027995	14
GO:0032642	regulation of chemokine production	13/815	84/21092	1.93×10^{-05}	0.0006	0.0005	ENSMUSG00000024079/ ENSMUSG00000001123/ ENSMUSG00000031639/ ENSMUSG00000003032/ ENSMUSG00000032508/ ENSMUSG00000039217/ ENSMUSG00000040329/ ENSMUSG00000035042/ ENSMUSG00000024810/ ENSMUSG00000027995/ ENSMUSG00000022676/ ENSMUSG00000038418/ ENSMUSG00000025746	13
GO:0050830	defense response to Gram−positive bacterium	15/815	109/21092	1.94×10^{-05}	0.0006	0.0005	ENSMUSG00000067212/ ENSMUSG00000032508/ ENSMUSG00000022575/ ENSMUSG00000020115/ ENSMUSG00000042333/ ENSMUSG00000038058/ ENSMUSG00000028270/ ENSMUSG00000040253/ ENSMUSG00000105096/ ENSMUSG00000028268/ ENSMUSG00000104713/ ENSMUSG00000029298/ ENSMUSG00000040264/ ENSMUSG00000027995/ ENSMUSG00000062210	15
GO:0032648	regulation of interferon−beta production	10/815	51/21092	2.13×10^{-05}	0.0006	0.0005	ENSMUSG00000025498/ ENSMUSG00000026896/ ENSMUSG00000040296/ ENSMUSG00000026946/ ENSMUSG00000029826/ ENSMUSG00000020707/ ENSMUSG00000031639/ ENSMUSG00000020115/ ENSMUSG00000018899/ ENSMUSG00000027995	10
GO:0032732	positive regulation of interleukin−1 production	10/815	51/21092	2.13×10^{-05}	0.0006	0.0005	ENSMUSG00000001123/ ENSMUSG00000071203/ ENSMUSG00000037860/ ENSMUSG00000022575/ ENSMUSG00000038058/ ENSMUSG00000025888/ ENSMUSG00000033538/ ENSMUSG00000027995/ ENSMUSG00000038418/ ENSMUSG00000024789	10

GO:0010952	positive regulation of peptidase activity	19/815	165/21092	2.18×10^{-05}	0.0006	0.0005	ENSMUSG00000026104/ ENSMUSG00000024066/ ENSMUSG00000064215/ ENSMUSG00000036986/ ENSMUSG00000022216/ ENSMUSG00000079197/ ENSMUSG00000021208/ ENSMUSG00000037860/ ENSMUSG00000022346/ ENSMUSG00000000915/ ENSMUSG00000004040/ ENSMUSG00000031948/ ENSMUSG00000038058/ ENSMUSG00000039304/ ENSMUSG00000025887/ ENSMUSG00000025888/ ENSMUSG00000034485/ ENSMUSG00000033538/ ENSMUSG00000024789	19
GO:0042267	natural killer cell mediated cytotoxicity	11/815	62/21092	2.25×10^{-05}	0.0006	0.0006	ENSMUSG00000037321/ ENSMUSG00000024339/ ENSMUSG00000001123/ ENSMUSG00000067212/ ENSMUSG00000040987/ ENSMUSG00000016206/ ENSMUSG00000030157/ ENSMUSG00000028793/ ENSMUSG00000039217/ ENSMUSG00000045827/ ENSMUSG00000062300	11
GO:0051092	positive regulation of NF−kappaB transcription factor activity	16/815	124/21092	2.35×10^{-05}	0.0007	0.0006	ENSMUSG00000024079/ ENSMUSG00000000275/ ENSMUSG00000047098/ ENSMUSG00000031639/ ENSMUSG00000039853/ ENSMUSG00000000127/ ENSMUSG00000032508/ ENSMUSG00000039217/ ENSMUSG00000037860/ ENSMUSG00000022514/ ENSMUSG00000027466/ ENSMUSG00000038058/ ENSMUSG00000078923/ ENSMUSG00000041187/ ENSMUSG00000026637/ ENSMUSG00000027995	16
GO:0046651	lymphocyte proliferation	28/815	307/21092	2.52×10^{-05}	0.0007	0.0006	ENSMUSG00000031897/ ENSMUSG00000115338/ ENSMUSG00000048806/ ENSMUSG00000027950/ ENSMUSG00000001123/ ENSMUSG00000067212/ ENSMUSG00000016206/ ENSMUSG00000070904/ ENSMUSG00000034218/ ENSMUSG00000000791/ ENSMUSG00000021795/ ENSMUSG00000032508/ ENSMUSG00000039217/ ENSMUSG00000031207/ ENSMUSG00000039285/ ENSMUSG00000066877/ ENSMUSG00000020115/ ENSMUSG00000042333/ ENSMUSG00000040329/ ENSMUSG00000029869/ ENSMUSG00000090958/ ENSMUSG00000016496/ ENSMUSG00000035042/ ENSMUSG00000035914/ ENSMUSG00000018899/ ENSMUSG00000031712/ ENSMUSG00000034165/ ENSMUSG00000025746	28

GO:0006471	protein ADP−ribosylation	8/815	33/21092	2.82×10^{-05}	0.0008	0.0007	ENSMUSG00000034422/ ENSMUSG00000022906/ ENSMUSG00000063268/ ENSMUSG00000023249/ ENSMUSG00000021266/ ENSMUSG00000000708/ ENSMUSG00000022722/ ENSMUSG00000034842	8
GO:0032611	interleukin−1 beta production	12/815	75/21092	2.85×10^{-05}	0.0008	0.0007	ENSMUSG00000036986/ ENSMUSG00000071203/ ENSMUSG00000037860/ ENSMUSG00000022575/ ENSMUSG00000038058/ ENSMUSG00000025888/ ENSMUSG00000105504/ ENSMUSG00000033538/ ENSMUSG00000027995/ ENSMUSG00000038418/ ENSMUSG00000062210/ ENSMUSG00000024789	12
GO:0032675	regulation of interleukin−6 production	17/815	140/21092	2.94×10^{-05}	0.0008	0.0007	ENSMUSG00000026896/ ENSMUSG00000040296/ ENSMUSG00000001123/ ENSMUSG00000036908/ ENSMUSG00000031639/ ENSMUSG00000037447/ ENSMUSG00000032508/ ENSMUSG00000055447/ ENSMUSG00000022514/ ENSMUSG00000038058/ ENSMUSG00000055148/ ENSMUSG00000030921/ ENSMUSG00000034610/ ENSMUSG00000024810/ ENSMUSG00000026981/ ENSMUSG00000027995/ ENSMUSG00000025746	17
GO:0002228	natural killer cell mediated immunity	11/815	64/21092	3.06×10^{-05}	0.0008	0.0007	ENSMUSG00000037321/ ENSMUSG00000024339/ ENSMUSG00000001123/ ENSMUSG00000067212/ ENSMUSG00000040987/ ENSMUSG00000016206/ ENSMUSG00000030157/ ENSMUSG00000028793/ ENSMUSG00000039217/ ENSMUSG00000045827/ ENSMUSG00000062300	11
GO:0072376	protein activation cascade	11/815	64/21092	3.06×10^{-05}	0.0008	0.0007	ENSMUSG00000024371/ ENSMUSG00000023224/ ENSMUSG00000055172/ ENSMUSG00000079343/ ENSMUSG00000026405/ ENSMUSG00000073418/ ENSMUSG00000015451/ ENSMUSG00000030681/ ENSMUSG00000026365/ ENSMUSG00000090231/ ENSMUSG00000038521	11
GO:2000116	regulation of cysteine−type endopeptidase activity	23/815	230/21092	3.15×10^{-05}	0.0009	0.0008	ENSMUSG00000026104/ ENSMUSG00000096727/ ENSMUSG00000024066/ ENSMUSG00000064215/ ENSMUSG00000036986/ ENSMUSG00000078945/ ENSMUSG00000021208/ ENSMUSG00000003032/ ENSMUSG00000037860/ ENSMUSG00000022346/ ENSMUSG00000000915/ ENSMUSG00000040152/ ENSMUSG00000030654/ ENSMUSG00000038058/ ENSMUSG00000039304/ ENSMUSG00000025887/ ENSMUSG00000025888/ ENSMUSG00000004231/	23

							ENSMUSG00000034485/ ENSMUSG00000033538/ ENSMUSG00000028289/ ENSMUSG00000024789/ ENSMUSG00000025746	
GO:0032731	positive regulation of interleukin−1 beta production	9/815	43/21092	3.18×10^{-05}	0.0009	0.0008	ENSMUSG00000071203/ ENSMUSG00000037860/ ENSMUSG00000022575/ ENSMUSG00000038058/ ENSMUSG00000025888/ ENSMUSG00000033538/ ENSMUSG00000027995/ ENSMUSG00000038418/ ENSMUSG00000024789	9
GO:0032602	chemokine production	13/815	88/21092	3.21×10^{-05}	0.0009	0.0008	ENSMUSG00000024079/ ENSMUSG00000001123/ ENSMUSG00000031639/ ENSMUSG00000003032/ ENSMUSG00000032508/ ENSMUSG00000039217/ ENSMUSG00000040329/ ENSMUSG00000035042/ ENSMUSG00000024810/ ENSMUSG00000027995/ ENSMUSG00000022676/ ENSMUSG00000038418/ ENSMUSG00000025746	13
GO:0098792	xenophagy	5/815	11/21092	3.24×10^{-05}	0.0009	0.0008	ENSMUSG00000057554/ ENSMUSG00000026672/ ENSMUSG00000020115/ ENSMUSG00000038058/ ENSMUSG00000027995	5
GO:0002478	antigen processing and presentation of exogenous peptide antigen	7/815	25/21092	3.27×10^{-05}	0.0009	0.0008	ENSMUSG00000037321/ ENSMUSG00000024339/ ENSMUSG00000061232/ ENSMUSG00000024308/ ENSMUSG00000060802/ ENSMUSG00000036908/ ENSMUSG00000016206	7
GO:0046700	heterocycle catabolic process	36/815	450/21092	3.34×10^{-05}	0.0009	0.0008	ENSMUSG00000027639/ ENSMUSG00000024066/ ENSMUSG00000020464/ ENSMUSG00000115338/ ENSMUSG00000002227/ ENSMUSG00000040613/ ENSMUSG00000027580/ ENSMUSG00000027233/ ENSMUSG00000029826/ ENSMUSG00000034575/ ENSMUSG00000034218/ ENSMUSG00000039236/ ENSMUSG00000066800/ ENSMUSG00000023960/ ENSMUSG00000035248/ ENSMUSG00000020407/ ENSMUSG00000019872/ ENSMUSG00000032508/ ENSMUSG00000029759/ ENSMUSG00000022346/ ENSMUSG00000042772/ ENSMUSG00000032410/ ENSMUSG00000027433/ ENSMUSG00000037331/ ENSMUSG00000004040/ ENSMUSG00000058624/ ENSMUSG00000024472/ ENSMUSG00000032690/ ENSMUSG00000000838/ ENSMUSG00000034724/ ENSMUSG00000095687/ ENSMUSG00000023961/ ENSMUSG00000026986/ ENSMUSG00000034610/ ENSMUSG00000021127/ ENSMUSG00000028599	36

GO:0002702	positive regulation of production of molecular mediator of immune response	14/815	101/21092	3.36×10^{-05}	0.0009	0.0008	ENSMUSG00000040296/ ENSMUSG00000060802/ ENSMUSG00000067212/ ENSMUSG00000072244/ ENSMUSG00000037447/ ENSMUSG00000039217/ ENSMUSG00000042333/ ENSMUSG00000031948/ ENSMUSG00000020641/ ENSMUSG00000024810/ ENSMUSG00000027995/ ENSMUSG00000008734/ ENSMUSG00000030895/ ENSMUSG00000025746	14
GO:0002262	myeloid cell homeostasis	19/815	171/21092	3.60×10^{-05}	0.0010	0.0008	ENSMUSG00000026104/ ENSMUSG00000027951/ ENSMUSG00000060802/ ENSMUSG00000002602/ ENSMUSG00000032698/ ENSMUSG00000039217/ ENSMUSG00000004040/ ENSMUSG00000035692/ ENSMUSG00000055148/ ENSMUSG00000070056/ ENSMUSG00000027737/ ENSMUSG00000045502/ ENSMUSG00000038855/ ENSMUSG00000031543/ ENSMUSG00000024789/ ENSMUSG00000028382/ ENSMUSG00000035385/ ENSMUSG00000021127/ ENSMUSG00000025746	19
GO:0010950	positive regulation of endopeptidase activity	18/815	157/21092	3.78×10^{-05}	0.0010	0.0009	ENSMUSG00000026104/ ENSMUSG00000024066/ ENSMUSG00000064215/ ENSMUSG00000036986/ ENSMUSG00000022216/ ENSMUSG00000079197/ ENSMUSG00000021208/ ENSMUSG00000037860/ ENSMUSG00000022346/ ENSMUSG00000000915/ ENSMUSG00000004040/ ENSMUSG00000038058/ ENSMUSG00000039304/ ENSMUSG00000025887/ ENSMUSG00000025888/ ENSMUSG00000034485/ ENSMUSG00000033538/ ENSMUSG00000024789	18
GO:0045953	negative regulation of natural killer cell mediated cytotoxicity	6/815	18/21092	4.07×10^{-05}	0.0011	0.0009	ENSMUSG00000037321/ ENSMUSG00000024339/ ENSMUSG00000001123/ ENSMUSG00000067212/ ENSMUSG00000030157/ ENSMUSG00000045827	6
GO:0043122	regulation of I−kappaB kinase/NF−kappaB signaling	18/815	159/21092	4.47×10^{-05}	0.0012	0.0010	ENSMUSG00000026104/ ENSMUSG00000029826/ ENSMUSG00000000275/ ENSMUSG00000047098/ ENSMUSG00000031639/ ENSMUSG00000045038/ ENSMUSG00000026672/ ENSMUSG00000032508/ ENSMUSG00000055204/ ENSMUSG00000027466/ ENSMUSG00000038058/ ENSMUSG00000078923/ ENSMUSG00000025888/ ENSMUSG00000026637/ ENSMUSG00000035186/ ENSMUSG00000028028/ ENSMUSG00000026981/ ENSMUSG00000008734	18

GO:0030098	lymphocyte differentiation	32/815	388/21092	5.05×10^{-05}	0.0013	0.0011	ENSMUSG00000115338/ ENSMUSG00000060802/ ENSMUSG00000048806/ ENSMUSG00000001123/ ENSMUSG00000016206/ ENSMUSG00000070904/ ENSMUSG00000034218/ ENSMUSG00000057554/ ENSMUSG00000014773/ ENSMUSG00000002602/ ENSMUSG00000038037/ ENSMUSG00000039217/ ENSMUSG00000020918/ ENSMUSG00000028217/ ENSMUSG00000110206/ ENSMUSG00000031154/ ENSMUSG00000024238/ ENSMUSG00000004040/ ENSMUSG00000040329/ ENSMUSG00000032815/ ENSMUSG00000031304/ ENSMUSG00000021453/ ENSMUSG00000020641/ ENSMUSG00000037440/ ENSMUSG00000038855/ ENSMUSG00000018899/ ENSMUSG00000031712/ ENSMUSG00000039153/ ENSMUSG00000038418/ ENSMUSG00000031093/ ENSMUSG00000021127/ ENSMUSG00000025746	32
GO:0001781	neutrophil apoptotic process	5/815	12/21092	5.37×10^{-05}	0.0014	0.0012	ENSMUSG00000039217/ ENSMUSG00000027737/ ENSMUSG00000045502/ ENSMUSG00000038855/ ENSMUSG00000025746	5
GO:0051091	positive regulation of DNA−binding transcription factor activity	23/815	238/21092	5.38×10^{-05}	0.0014	0.0012	ENSMUSG00000040296/ ENSMUSG00000024079/ ENSMUSG00000030966/ ENSMUSG00000000275/ ENSMUSG00000047098/ ENSMUSG00000024457/ ENSMUSG00000031639/ ENSMUSG00000039853/ ENSMUSG00000000127/ ENSMUSG00000032508/ ENSMUSG00000039217/ ENSMUSG00000037860/ ENSMUSG00000022514/ ENSMUSG00000021067/ ENSMUSG00000027466/ ENSMUSG00000038058/ ENSMUSG00000078923/ ENSMUSG00000041187/ ENSMUSG00000026637/ ENSMUSG00000001627/ ENSMUSG00000027995/ ENSMUSG00000024789/ ENSMUSG00000025746	23
GO:0044270	cellular nitrogen compound catabolic process	35/815	443/21092	5.51×10^{-05}	0.0014	0.0012	ENSMUSG00000027639/ ENSMUSG00000024066/ ENSMUSG00000020464/ ENSMUSG00000115338/ ENSMUSG00000002227/ ENSMUSG00000040613/ ENSMUSG00000027580/ ENSMUSG00000027233/ ENSMUSG00000029826/ ENSMUSG00000034575/ ENSMUSG00000034218/ ENSMUSG00000039236/ ENSMUSG00000066800/ ENSMUSG00000023960/ ENSMUSG00000035248/ ENSMUSG00000020407/ ENSMUSG00000019872/ ENSMUSG00000032508/	35

							ENSMUSG00000022346/ ENSMUSG00000042772/ ENSMUSG00000032410/ ENSMUSG00000027433/ ENSMUSG00000037331/ ENSMUSG00000004040/ ENSMUSG00000058624/ ENSMUSG00000024472/ ENSMUSG00000032690/ ENSMUSG00000000838/ ENSMUSG00000034724/ ENSMUSG00000095687/ ENSMUSG00000023961/ ENSMUSG00000026986/ ENSMUSG00000034610/ ENSMUSG00000021127/ ENSMUSG00000028599	
GO:0002753	cytoplasmic pattern recognition receptor signaling pathway	8/815	36/21092	5.55 × 10⁻⁰⁵	0.0014	0.0012	ENSMUSG00000017830/ ENSMUSG00000069874/ ENSMUSG00000026896/ ENSMUSG00000040296/ ENSMUSG00000029826/ ENSMUSG00000037921/ ENSMUSG00000055204/ ENSMUSG00000028028	8
GO:0043281	regulation of cysteine−type endopeptidase activity involved in apoptotic process	21/815	207/21092	5.56 × 10⁻⁰⁵	0.0014	0.0012	ENSMUSG00000026104/ ENSMUSG00000024066/ ENSMUSG00000064215/ ENSMUSG00000036986/ ENSMUSG00000078945/ ENSMUSG00000021208/ ENSMUSG00000003032/ ENSMUSG00000022346/ ENSMUSG00000000915/ ENSMUSG00000040152/ ENSMUSG00000030654/ ENSMUSG00000038058/ ENSMUSG00000039304/ ENSMUSG00000025887/ ENSMUSG00000025888/ ENSMUSG00000004231/ ENSMUSG00000034485/ ENSMUSG00000033538/ ENSMUSG00000028289/ ENSMUSG00000024789/ ENSMUSG00000025746	21
GO:0006401	RNA catabolic process	23/815	239/21092	5.74 × 10⁻⁰⁵	0.0014	0.0012	ENSMUSG00000020464/ ENSMUSG00000002227/ ENSMUSG00000040613/ ENSMUSG00000027580/ ENSMUSG00000027233/ ENSMUSG00000029826/ ENSMUSG00000034575/ ENSMUSG00000034218/ ENSMUSG00000039236/ ENSMUSG00000066800/ ENSMUSG00000035248/ ENSMUSG00000032508/ ENSMUSG00000042772/ ENSMUSG00000032410/ ENSMUSG00000037331/ ENSMUSG00000024472/ ENSMUSG00000032690/ ENSMUSG00000000838/ ENSMUSG00000034724/ ENSMUSG00000095687/ ENSMUSG00000034610/ ENSMUSG00000021127/ ENSMUSG00000028599	23
GO:0002716	negative regulation of natural killer cell mediated immunity	6/815	19/21092	5.75 × 10⁻⁰⁵	0.0014	0.0012	ENSMUSG00000037321/ ENSMUSG00000024339/ ENSMUSG00000001123/ ENSMUSG00000067212/ ENSMUSG00000030157/ ENSMUSG00000045827	6

GO:0060338	regulation of type I interferon−mediated signaling pathway	6/815	19/21092	5.75×10^{-05}	0.0014	0.0012	ENSMUSG00000025498/ ENSMUSG00000027639/ ENSMUSG00000027951/ ENSMUSG00000072244/ ENSMUSG00000074151/ ENSMUSG00000027514	6
GO:0051090	regulation of DNA−binding transcription factor activity	32/815	391/21092	5.85×10^{-05}	0.0014	0.0013	ENSMUSG00000040296/ ENSMUSG00000024079/ ENSMUSG00000030966/ ENSMUSG00000063268/ ENSMUSG00000000275/ ENSMUSG00000047098/ ENSMUSG00000024457/ ENSMUSG00000031639/ ENSMUSG00000039853/ ENSMUSG00000000127/ ENSMUSG00000003032/ ENSMUSG00000032508/ ENSMUSG00000039217/ ENSMUSG00000037860/ ENSMUSG00000022514/ ENSMUSG00000003308/ ENSMUSG00000074151/ ENSMUSG00000031540/ ENSMUSG00000021067/ ENSMUSG00000027466/ ENSMUSG00000038058/ ENSMUSG00000078923/ ENSMUSG00000041187/ ENSMUSG00000032501/ ENSMUSG00000032815/ ENSMUSG00000034610/ ENSMUSG00000026637/ ENSMUSG00000022126/ ENSMUSG00000001627/ ENSMUSG00000027995/ ENSMUSG00000024789/ ENSMUSG00000025746	32
GO:0006919	activation of cysteine−type endopeptidase activity involved in apoptotic process	12/815	81/21092	6.23×10^{-05}	0.0015	0.0013	ENSMUSG00000026104/ ENSMUSG00000024066/ ENSMUSG00000064215/ ENSMUSG00000036986/ ENSMUSG00000021208/ ENSMUSG00000022346/ ENSMUSG00000000915/ ENSMUSG00000038058/ ENSMUSG00000025887/ ENSMUSG00000025888/ ENSMUSG00000033538/ ENSMUSG00000024789	12
GO:0019439	aromatic compound catabolic process	36/815	464/21092	6.29×10^{-05}	0.0015	0.0013	ENSMUSG00000027639/ ENSMUSG00000024066/ ENSMUSG00000020464/ ENSMUSG00000115338/ ENSMUSG00000002227/ ENSMUSG00000040613/ ENSMUSG00000027580/ ENSMUSG00000027233/ ENSMUSG00000029826/ ENSMUSG00000034575/ ENSMUSG00000034218/ ENSMUSG00000039236/ ENSMUSG00000066800/ ENSMUSG00000023960/ ENSMUSG00000035248/ ENSMUSG00000020407/ ENSMUSG00000019872/ ENSMUSG00000032508/ ENSMUSG00000029759/ ENSMUSG00000022346/ ENSMUSG00000042772/ ENSMUSG00000032410/ ENSMUSG00000027433/ ENSMUSG00000037331/ ENSMUSG00000004040/ ENSMUSG00000058624/ ENSMUSG00000024472/	36

							ENSMUSG00000032690/ ENSMUSG00000000838/ ENSMUSG00000034724/ ENSMUSG00000095687/ ENSMUSG00000023961/ ENSMUSG00000026986/ ENSMUSG00000034610/ ENSMUSG00000021127/ ENSMUSG00000028599	
GO:0032612	interleukin−1 production	13/815	94/21092	6.51×10^{-05}	0.0016	0.0014	ENSMUSG00000036986/ ENSMUSG00000001123/ ENSMUSG00000071203/ ENSMUSG00000037860/ ENSMUSG00000022575/ ENSMUSG00000038058/ ENSMUSG00000025888/ ENSMUSG00000105504/ ENSMUSG00000033538/ ENSMUSG00000027995/ ENSMUSG00000038418/ ENSMUSG00000062210/ ENSMUSG00000024789	13
GO:0045621	positive regulation of lymphocyte differentiation	14/815	108/21092	7.13×10^{-05}	0.0017	0.0015	ENSMUSG00000115338/ ENSMUSG00000001123/ ENSMUSG00000016206/ ENSMUSG00000002602/ ENSMUSG00000038037/ ENSMUSG00000039217/ ENSMUSG00000110206/ ENSMUSG00000040329/ ENSMUSG00000031304/ ENSMUSG00000037440/ ENSMUSG00000038855/ ENSMUSG00000018899/ ENSMUSG00000031712/ ENSMUSG00000025746	14
GO:0045619	regulation of lymphocyte differentiation	19/815	180/21092	7.28×10^{-05}	0.0017	0.0015	ENSMUSG00000115338/ ENSMUSG00000048806/ ENSMUSG00000001123/ ENSMUSG00000016206/ ENSMUSG00000002602/ ENSMUSG00000038037/ ENSMUSG00000039217/ ENSMUSG00000020918/ ENSMUSG00000110206/ ENSMUSG00000024238/ ENSMUSG00000040329/ ENSMUSG00000032815/ ENSMUSG00000031304/ ENSMUSG00000037440/ ENSMUSG00000038855/ ENSMUSG00000018899/ ENSMUSG00000031712/ ENSMUSG00000021127/ ENSMUSG00000025746	19
GO:0045589	regulation of regulatory T cell differentiation	7/815	28/21092	7.29×10^{-05}	0.0017	0.0015	ENSMUSG00000001123/ ENSMUSG00000016206/ ENSMUSG00000038037/ ENSMUSG00000020918/ ENSMUSG00000032815/ ENSMUSG00000031304/ ENSMUSG00000018899	7
GO:0032649	regulation of interferon−gamma production	14/815	109/21092	7.89×10^{-05}	0.0019	0.0016	ENSMUSG00000001123/ ENSMUSG00000016206/ ENSMUSG00000031639/ ENSMUSG00000000791/ ENSMUSG00000002602/ ENSMUSG00000060550/ ENSMUSG00000037447/ ENSMUSG00000039217/ ENSMUSG00000055447/	14

							ENSMUSG00000035692/ ENSMUSG00000016496/ ENSMUSG00000022378/ ENSMUSG00000035914/ ENSMUSG00000024810	
GO:0002367	cytokine production involved in immune response	13/815	96/21092	8.13 × 10^{-05}	0.0019	0.0017	ENSMUSG00000040296/ ENSMUSG00000060802/ ENSMUSG00000048806/ ENSMUSG00000031639/ ENSMUSG00000072244/ ENSMUSG00000037447/ ENSMUSG00000039217/ ENSMUSG00000042333/ ENSMUSG00000031948/ ENSMUSG00000020641/ ENSMUSG00000027995/ ENSMUSG00000008734/ ENSMUSG00000025746	13
GO:0001818	negative regulation of cytokine production	23/815	245/21092	8.40 × 10^{-05}	0.0020	0.0017	ENSMUSG00000017830/ ENSMUSG00000026946/ ENSMUSG00000036986/ ENSMUSG00000048806/ ENSMUSG00000001123/ ENSMUSG00000014773/ ENSMUSG00000002602/ ENSMUSG00000021795/ ENSMUSG00000003032/ ENSMUSG00000040152/ ENSMUSG00000031154/ ENSMUSG00000041736/ ENSMUSG00000090958/ ENSMUSG00000016496/ ENSMUSG00000055148/ ENSMUSG00000030921/ ENSMUSG00000079363/ ENSMUSG00000035914/ ENSMUSG00000024810/ ENSMUSG00000022126/ ENSMUSG00000027995/ ENSMUSG00000061665/ ENSMUSG00000025746	23
GO:0046631	alpha−beta T cell activation	17/815	153/21092	9.13 × 10^{-05}	0.0021	0.0019	ENSMUSG00000115338/ ENSMUSG00000001123/ ENSMUSG00000067212/ ENSMUSG00000016206/ ENSMUSG00000038037/ ENSMUSG00000039217/ ENSMUSG00000031154/ ENSMUSG00000042333/ ENSMUSG00000004040/ ENSMUSG00000031304/ ENSMUSG00000016496/ ENSMUSG00000021453/ ENSMUSG00000020641/ ENSMUSG00000038855/ ENSMUSG00000018899/ ENSMUSG00000031712/ ENSMUSG00000025746	17
GO:0006959	humoral immune response	20/815	199/21092	9.45 × 10^{-05}	0.0022	0.0019	ENSMUSG00000024371/ ENSMUSG00000048806/ ENSMUSG00000067212/ ENSMUSG00000070904/ ENSMUSG00000023224/ ENSMUSG00000021795/ ENSMUSG00000055172/ ENSMUSG00000079343/ ENSMUSG00000026405/ ENSMUSG00000073418/ ENSMUSG00000015451/ ENSMUSG00000034855/ ENSMUSG00000026365/ ENSMUSG00000060183/ ENSMUSG00000029417/	20

							ENSMUSG00000090231/ ENSMUSG00000022126/ ENSMUSG00000038521/ ENSMUSG00000030895/ ENSMUSG00000035385	
GO:0030217	T cell differentiation	24/815	264/21092	9.83×10^{-05}	0.0023	0.0020	ENSMUSG00000115338/ ENSMUSG00000060802/ ENSMUSG00000048806/ ENSMUSG00000001123/ ENSMUSG00000016206/ ENSMUSG00000038037/ ENSMUSG00000039217/ ENSMUSG00000020918/ ENSMUSG00000031154/ ENSMUSG00000024238/ ENSMUSG00000004040/ ENSMUSG00000040329/ ENSMUSG00000032815/ ENSMUSG00000031304/ ENSMUSG00000021453/ ENSMUSG00000020641/ ENSMUSG00000037440/ ENSMUSG00000038855/ ENSMUSG00000018899/ ENSMUSG00000031712/ ENSMUSG00000039153/ ENSMUSG00000038418/ ENSMUSG00000021127/ ENSMUSG00000025746	24
GO:1901361	organic cyclic compound catabolic process	37/815	494/21092	0.0001	0.0024	0.0021	ENSMUSG00000027639/ ENSMUSG00000024066/ ENSMUSG00000020464/ ENSMUSG00000115338/ ENSMUSG00000002227/ ENSMUSG00000040613/ ENSMUSG00000027580/ ENSMUSG00000027233/ ENSMUSG00000029826/ ENSMUSG00000034575/ ENSMUSG00000034218/ ENSMUSG00000039236/ ENSMUSG00000066800/ ENSMUSG00000023960/ ENSMUSG00000035248/ ENSMUSG00000020407/ ENSMUSG00000019872/ ENSMUSG00000032508/ ENSMUSG00000029759/ ENSMUSG00000022346/ ENSMUSG00000016194/ ENSMUSG00000042772/ ENSMUSG00000032410/ ENSMUSG00000027433/ ENSMUSG00000037331/ ENSMUSG00000004040/ ENSMUSG00000058624/ ENSMUSG00000024472/ ENSMUSG00000032690/ ENSMUSG00000000838/ ENSMUSG00000034724/ ENSMUSG00000095687/ ENSMUSG00000023961/ ENSMUSG00000026986/ ENSMUSG00000034610/ ENSMUSG00000021127/ ENSMUSG00000028599	37
GO:0001780	neutrophil homeostasis	6/815	21/21092	0.0001	0.0025	0.0021	ENSMUSG00000002602/ ENSMUSG00000039217/ ENSMUSG00000027737/ ENSMUSG00000045502/ ENSMUSG00000038855/ ENSMUSG00000025746	6
GO:0071560	cellular response to transforming growth factor beta stimulus	20/815	201/21092	0.0001	0.0025	0.0022	ENSMUSG00000036986/ ENSMUSG00000027204/ ENSMUSG00000021360/ ENSMUSG00000038400/ ENSMUSG00000040152/ ENSMUSG00000024238/	20

							ENSMUSG00000021796/ ENSMUSG00000052684/ ENSMUSG00000090958/ ENSMUSG00000029860/ ENSMUSG00000021065/ ENSMUSG00000021754/ ENSMUSG00000001870/ ENSMUSG00000092035/ ENSMUSG00000069255/ ENSMUSG00000021250/ ENSMUSG00000035385/ ENSMUSG00000021127/ ENSMUSG00000046186/ ENSMUSG00000027660	
GO:0031663	lipopolysaccharide−mediated signaling pathway	9/815	50/21092	0.0001	0.0025	0.0022	ENSMUSG00000026104/ ENSMUSG00000045038/ ENSMUSG00000032508/ ENSMUSG00000039217/ ENSMUSG00000032501/ ENSMUSG00000035042/ ENSMUSG00000025779/ ENSMUSG00000034610/ ENSMUSG00000022126	9
GO:0042269	regulation of natural killer cell mediated cytotoxicity	9/815	50/21092	0.0001	0.0025	0.0022	ENSMUSG00000037321/ ENSMUSG00000024339/ ENSMUSG00000001123/ ENSMUSG00000067212/ ENSMUSG00000040987/ ENSMUSG00000016206/ ENSMUSG00000030157/ ENSMUSG00000045827/ ENSMUSG00000062300	9
GO:0001774	microglial cell activation	7/815	30/21092	0.0001	0.0026	0.0023	ENSMUSG00000034218/ ENSMUSG00000031639/ ENSMUSG00000020572/ ENSMUSG00000052684/ ENSMUSG00000025888/ ENSMUSG00000024810/ ENSMUSG00000027995	7
GO:0002269	leukocyte activation involved in inflammatory response	7/815	30/21092	0.0001	0.0026	0.0023	ENSMUSG00000034218/ ENSMUSG00000031639/ ENSMUSG00000020572/ ENSMUSG00000052684/ ENSMUSG00000025888/ ENSMUSG00000024810/ ENSMUSG00000027995	7
GO:0034655	nucleobase−containing compound catabolic process	32/815	406/21092	0.0001	0.0026	0.0023	ENSMUSG00000027639/ ENSMUSG00000020464/ ENSMUSG00000115338/ ENSMUSG00000002227/ ENSMUSG00000040613/ ENSMUSG00000027580/ ENSMUSG00000027233/ ENSMUSG00000029826/ ENSMUSG00000034575/ ENSMUSG00000034218/ ENSMUSG00000039236/ ENSMUSG00000066800/ ENSMUSG00000023960/ ENSMUSG00000035248/ ENSMUSG00000020407/ ENSMUSG00000019872/ ENSMUSG00000032508/ ENSMUSG00000022346/ ENSMUSG00000042772/ ENSMUSG00000032410/ ENSMUSG00000027433/ ENSMUSG00000037331/ ENSMUSG00000004040/ ENSMUSG00000024472/ ENSMUSG00000032690/ ENSMUSG00000000838/ ENSMUSG00000034724/	32

							ENSMUSG00000095687/ ENSMUSG00000023961/ ENSMUSG00000034610/ ENSMUSG00000021127/ ENSMUSG00000028599	
GO:0007260	tyrosine phosphorylation of STAT protein	11/815	74/21092	0.0001	0.0027	0.0023	ENSMUSG00000034422/ ENSMUSG00000022906/ ENSMUSG00000038037/ ENSMUSG00000000127/ ENSMUSG00000039217/ ENSMUSG00000035042/ ENSMUSG00000018899/ ENSMUSG00000031712/ ENSMUSG00000030895/ ENSMUSG00000024789/ ENSMUSG00000025746	11
GO:0031348	negative regulation of defense response	20/815	203/21092	0.0001	0.0027	0.0024	ENSMUSG00000017830/ ENSMUSG00000034422/ ENSMUSG00000037321/ ENSMUSG00000024339/ ENSMUSG00000027639/ ENSMUSG00000042726/ ENSMUSG00000026946/ ENSMUSG00000027951/ ENSMUSG00000001123/ ENSMUSG00000067212/ ENSMUSG00000040987/ ENSMUSG00000030157/ ENSMUSG00000023224/ ENSMUSG00000074151/ ENSMUSG00000045827/ ENSMUSG00000070056/ ENSMUSG00000035914/ ENSMUSG00000022126/ ENSMUSG00000034485/ ENSMUSG00000028599	20
GO:0010508	positive regulation of autophagy	14/815	114/21092	0.0001	0.0028	0.0025	ENSMUSG00000069874/ ENSMUSG00000030966/ ENSMUSG00000026672/ ENSMUSG00000021036/ ENSMUSG00000004936/ ENSMUSG00000046879/ ENSMUSG00000037331/ ENSMUSG00000020115/ ENSMUSG00000025241/ ENSMUSG00000038058/ ENSMUSG00000037062/ ENSMUSG00000047496/ ENSMUSG00000028211/ ENSMUSG00000027995	14
GO:0002715	regulation of natural killer cell mediated immunity	9/815	51/21092	0.0001	0.0028	0.0025	ENSMUSG00000037321/ ENSMUSG00000024339/ ENSMUSG00000001123/ ENSMUSG00000067212/ ENSMUSG00000040987/ ENSMUSG00000016206/ ENSMUSG00000030157/ ENSMUSG00000045827/ ENSMUSG00000062300	9
GO:0002700	regulation of production of molecular mediator of immune response	16/815	143/21092	0.0001	0.0029	0.0025	ENSMUSG00000040296/ ENSMUSG00000060802/ ENSMUSG00000048806/ ENSMUSG00000067212/ ENSMUSG00000031639/ ENSMUSG00000072244/ ENSMUSG00000037447/ ENSMUSG00000039217/ ENSMUSG00000042333/ ENSMUSG00000031948/ ENSMUSG00000020641/ ENSMUSG00000024810/ ENSMUSG00000027995/ ENSMUSG00000008734/ ENSMUSG00000030895/ ENSMUSG00000025746	16

GO:0001911	negative regulation of leukocyte mediated cytotoxicity	6/815	22/21092	0.0001	0.0031	0.0027	ENSMUSG00000037321/ ENSMUSG00000024339/ ENSMUSG00000001123/ ENSMUSG00000067212/ ENSMUSG00000030157/ ENSMUSG00000045827	6
GO:0039528	cytoplasmic pattern recognition receptor signaling pathway in response to virus	6/815	22/21092	0.0001	0.0031	0.0027	ENSMUSG00000017830/ ENSMUSG00000026896/ ENSMUSG00000040296/ ENSMUSG00000029826/ ENSMUSG00000037921/ ENSMUSG00000055204	6
GO:0045066	regulatory T cell differentiation	7/815	31/21092	0.0001	0.0031	0.0027	ENSMUSG00000001123/ ENSMUSG00000016206/ ENSMUSG00000038037/ ENSMUSG00000020918/ ENSMUSG00000032815/ ENSMUSG00000031304/ ENSMUSG00000018899	7
GO:0006914	autophagy	30/815	375/21092	0.0001	0.0031	0.0027	ENSMUSG00000069874/ ENSMUSG00000030966/ ENSMUSG00000034218/ ENSMUSG00000057554/ ENSMUSG00000020572/ ENSMUSG00000026672/ ENSMUSG00000054676/ ENSMUSG00000056692/ ENSMUSG00000021036/ ENSMUSG00000016534/ ENSMUSG00000004936/ ENSMUSG00000046879/ ENSMUSG00000021814/ ENSMUSG00000038467/ ENSMUSG00000041736/ ENSMUSG00000037331/ ENSMUSG00000020115/ ENSMUSG00000025241/ ENSMUSG00000038058/ ENSMUSG00000035354/ ENSMUSG00000001750/ ENSMUSG00000042082/ ENSMUSG00000037062/ ENSMUSG00000054072/ ENSMUSG00000006930/ ENSMUSG00000047496/ ENSMUSG00000025888/ ENSMUSG00000030921/ ENSMUSG00000028211/ ENSMUSG00000027995	30
GO:0061919	process utilizing autophagic mechanism	30/815	375/21092	0.0001	0.0031	0.0027	ENSMUSG00000069874/ ENSMUSG00000030966/ ENSMUSG00000034218/ ENSMUSG00000057554/ ENSMUSG00000020572/ ENSMUSG00000026672/ ENSMUSG00000054676/ ENSMUSG00000056692/ ENSMUSG00000021036/ ENSMUSG00000016534/ ENSMUSG00000004936/ ENSMUSG00000046879/ ENSMUSG00000021814/ ENSMUSG00000038467/ ENSMUSG00000041736/ ENSMUSG00000037331/ ENSMUSG00000020115/ ENSMUSG00000025241/ ENSMUSG00000038058/ ENSMUSG00000035354/ ENSMUSG00000001750/ ENSMUSG00000042082/ ENSMUSG00000037062/ ENSMUSG00000054072/	30

							ENSMUSG00000006930/ ENSMUSG00000047496/ ENSMUSG00000025888/ ENSMUSG00000030921/ ENSMUSG00000028211/ ENSMUSG00000027995	
GO:0071559	response to transforming growth factor beta	20/815	206/21092	0.0002	0.0032	0.0028	ENSMUSG00000036986/ ENSMUSG00000027204/ ENSMUSG00000021360/ ENSMUSG00000038400/ ENSMUSG00000040152/ ENSMUSG00000024238/ ENSMUSG00000021796/ ENSMUSG00000052684/ ENSMUSG00000090958/ ENSMUSG00000029860/ ENSMUSG00000021065/ ENSMUSG00000021754/ ENSMUSG00000001870/ ENSMUSG00000092035/ ENSMUSG00000069255/ ENSMUSG00000021250/ ENSMUSG00000035385/ ENSMUSG00000021127/ ENSMUSG00000046186/ ENSMUSG00000027660	20
GO:0051701	interaction with host	14/815	116/21092	0.0002	0.0033	0.0029	ENSMUSG00000025492/ ENSMUSG00000030966/ ENSMUSG00000036986/ ENSMUSG00000000275/ ENSMUSG00000024457/ ENSMUSG00000002602/ ENSMUSG00000043279/ ENSMUSG00000022346/ ENSMUSG00000038467/ ENSMUSG00000045827/ ENSMUSG00000035354/ ENSMUSG00000030921/ ENSMUSG00000027995/ ENSMUSG00000062300	14
GO:0032651	regulation of interleukin−1 beta production	10/815	64/21092	0.0002	0.0034	0.0029	ENSMUSG00000036986/ ENSMUSG00000071203/ ENSMUSG00000037860/ ENSMUSG00000022575/ ENSMUSG00000038058/ ENSMUSG00000025888/ ENSMUSG00000033538/ ENSMUSG00000027995/ ENSMUSG00000038418/ ENSMUSG00000024789	10
GO:0070663	regulation of leukocyte proliferation	22/815	240/21092	0.0002	0.0035	0.0031	ENSMUSG00000115338/ ENSMUSG00000027950/ ENSMUSG00000001123/ ENSMUSG00000067212/ ENSMUSG00000016206/ ENSMUSG00000034218/ ENSMUSG00000000791/ ENSMUSG00000021795/ ENSMUSG00000032508/ ENSMUSG00000039217/ ENSMUSG00000110206/ ENSMUSG00000066877/ ENSMUSG00000042333/ ENSMUSG00000040329/ ENSMUSG00000090958/ ENSMUSG00000016496/ ENSMUSG00000035042/ ENSMUSG00000035914/ ENSMUSG00000018899/ ENSMUSG00000031712/ ENSMUSG00000014599/ ENSMUSG00000025746	22

GO:0042098	T cell proliferation	20/815	208/21092	0.0002	0.0036	0.0031	ENSMUSG00000031897/ ENSMUSG00000115338/ ENSMUSG00000001123/ ENSMUSG00000067212/ ENSMUSG00000016206/ ENSMUSG00000000791/ ENSMUSG00000021795/ ENSMUSG00000039217/ ENSMUSG00000031207/ ENSMUSG00000066877/ ENSMUSG00000042333/ ENSMUSG00000029869/ ENSMUSG00000090958/ ENSMUSG00000016496/ ENSMUSG00000035042/ ENSMUSG00000035914/ ENSMUSG00000018899/ ENSMUSG00000031712/ ENSMUSG00000034165/ ENSMUSG00000025746	20
GO:0016239	positive regulation of macroautophagy	10/815	65/21092	0.0002	0.0038	0.0033	ENSMUSG00000069874/ ENSMUSG00000026672/ ENSMUSG00000021036/ ENSMUSG00000046879/ ENSMUSG00000037331/ ENSMUSG00000020115/ ENSMUSG00000025241/ ENSMUSG00000038058/ ENSMUSG00000037062/ ENSMUSG00000027995	10
GO:0045655	regulation of monocyte differentiation	5/815	15/21092	0.0002	0.0038	0.0033	ENSMUSG00000022346/ ENSMUSG00000052684/ ENSMUSG00000031750/ ENSMUSG00000014599/ ENSMUSG00000021127	5
GO:0098581	detection of external biotic stimulus	6/815	23/21092	0.0002	0.0039	0.0034	ENSMUSG00000078942/ ENSMUSG00000078945/ ENSMUSG00000071203/ ENSMUSG00000038058/ ENSMUSG00000025779/ ENSMUSG00000027995	6
GO:0048872	homeostasis of number of cells	26/815	310/21092	0.0002	0.0039	0.0034	ENSMUSG00000026104/ ENSMUSG00000027951/ ENSMUSG00000060802/ ENSMUSG00000002602/ ENSMUSG00000019699/ ENSMUSG00000032698/ ENSMUSG00000020128/ ENSMUSG00000039217/ ENSMUSG00000110206/ ENSMUSG00000004040/ ENSMUSG00000040329/ ENSMUSG00000035692/ ENSMUSG00000055148/ ENSMUSG00000070056/ ENSMUSG00000027737/ ENSMUSG00000045502/ ENSMUSG00000038855/ ENSMUSG00000031543/ ENSMUSG00000014599/ ENSMUSG00000031093/ ENSMUSG00000024789/ ENSMUSG00000028382/ ENSMUSG00000035385/ ENSMUSG00000021127/ ENSMUSG00000025746/ ENSMUSG00000027660	26
GO:0050714	positive regulation of protein secretion	25/815	293/21092	0.0002	0.0039	0.0034	ENSMUSG00000026896/ ENSMUSG00000040296/ ENSMUSG00000001123/ ENSMUSG00000067212/ ENSMUSG00000036908/ ENSMUSG00000072244/ ENSMUSG00000045038/ ENSMUSG00000037447/ ENSMUSG00000037860/	25

							ENSMUSG00000022575/ ENSMUSG00000022514/ ENSMUSG00000042333/ ENSMUSG00000031948/ ENSMUSG00000051790/ ENSMUSG00000016496/ ENSMUSG00000025888/ ENSMUSG00000035914/ ENSMUSG00000034584/ ENSMUSG00000002897/ ENSMUSG00000024810/ ENSMUSG00000045502/ ENSMUSG00000033538/ ENSMUSG00000027995/ ENSMUSG00000024789/ ENSMUSG00000025746	
GO:2000377	regulation of reactive oxygen species metabolic process	19/815	194/21092	0.0002	0.0040	0.0035	ENSMUSG00000024066/ ENSMUSG00000016206/ ENSMUSG00000037580/ ENSMUSG00000003032/ ENSMUSG00000039217/ ENSMUSG00000029759/ ENSMUSG00000055447/ ENSMUSG00000040152/ ENSMUSG00000041736/ ENSMUSG00000004040/ ENSMUSG00000031266/ ENSMUSG00000032066/ ENSMUSG00000055148/ ENSMUSG00000030921/ ENSMUSG00000022126/ ENSMUSG00000004231/ ENSMUSG00000027995/ ENSMUSG00000024789/ ENSMUSG00000025746	19
GO:0045580	regulation of T cell differentiation	16/815	148/21092	0.0002	0.0041	0.0035	ENSMUSG00000115338/ ENSMUSG00000048806/ ENSMUSG00000001123/ ENSMUSG00000016206/ ENSMUSG00000038037/ ENSMUSG00000039217/ ENSMUSG00000020918/ ENSMUSG00000024238/ ENSMUSG00000040329/ ENSMUSG00000032815/ ENSMUSG00000031304/ ENSMUSG00000037440/ ENSMUSG00000038855/ ENSMUSG00000018899/ ENSMUSG00000031712/ ENSMUSG00000025746	16
GO:0046718	viral entry into host cell	9/815	54/21092	0.0002	0.0041	0.0036	ENSMUSG00000025492/ ENSMUSG00000030966/ ENSMUSG00000000275/ ENSMUSG00000024457/ ENSMUSG00000002602/ ENSMUSG00000043279/ ENSMUSG00000035354/ ENSMUSG00000030921/ ENSMUSG00000062300	9
GO:0097696	STAT cascade	18/815	179/21092	0.0002	0.0041	0.0036	ENSMUSG00000026104/ ENSMUSG00000034422/ ENSMUSG00000022906/ ENSMUSG00000048806/ ENSMUSG00000070904/ ENSMUSG00000064128/ ENSMUSG00000038037/ ENSMUSG00000000127/ ENSMUSG00000039217/ ENSMUSG00000001642/ ENSMUSG00000053141/ ENSMUSG00000004040/	18

							ENSMUSG00000035042/ ENSMUSG00000018899/ ENSMUSG00000031712/ ENSMUSG00000030895/ ENSMUSG00000024789/ ENSMUSG00000025746	
GO:0046596	regulation of viral entry into host cell	7/815	33/21092	0.0002	0.0044	0.0039	ENSMUSG00000025492/ ENSMUSG00000030966/ ENSMUSG00000000275/ ENSMUSG00000024457/ ENSMUSG00000043279/ ENSMUSG00000030921/ ENSMUSG00000062300	7
GO:0048661	positive regulation of smooth muscle cell proliferation	13/815	106/21092	0.0002	0.0045	0.0039	ENSMUSG00000026104/ ENSMUSG00000020572/ ENSMUSG00000032508/ ENSMUSG00000039217/ ENSMUSG00000022346/ ENSMUSG00000001642/ ENSMUSG00000040152/ ENSMUSG00000021796/ ENSMUSG00000052684/ ENSMUSG00000035042/ ENSMUSG00000038418/ ENSMUSG00000024789/ ENSMUSG00000025746	13
GO:0048660	regulation of smooth muscle cell proliferation	17/815	165/21092	0.0002	0.0046	0.0040	ENSMUSG00000026104/ ENSMUSG00000020572/ ENSMUSG00000003032/ ENSMUSG00000032508/ ENSMUSG00000039217/ ENSMUSG00000022346/ ENSMUSG00000001642/ ENSMUSG00000040152/ ENSMUSG00000021796/ ENSMUSG00000052684/ ENSMUSG00000032501/ ENSMUSG00000035042/ ENSMUSG00000022206/ ENSMUSG00000031712/ ENSMUSG00000038418/ ENSMUSG00000024789/ ENSMUSG00000025746	17
GO:0032944	regulation of mononuclear cell proliferation	21/815	230/21092	0.0002	0.0049	0.0042	ENSMUSG00000115338/ ENSMUSG00000027950/ ENSMUSG00000001123/ ENSMUSG00000067212/ ENSMUSG00000016206/ ENSMUSG00000034218/ ENSMUSG00000000791/ ENSMUSG00000021795/ ENSMUSG00000032508/ ENSMUSG00000039217/ ENSMUSG00000066877/ ENSMUSG00000042333/ ENSMUSG00000040329/ ENSMUSG00000090958/ ENSMUSG00000016496/ ENSMUSG00000035042/ ENSMUSG00000035914/ ENSMUSG00000018899/ ENSMUSG00000031712/ ENSMUSG00000014599/ ENSMUSG00000025746	21
GO:0002707	negative regulation of lymphocyte mediated immunity	8/815	44/21092	0.0002	0.0049	0.0042	ENSMUSG00000037321/ ENSMUSG00000024339/ ENSMUSG00000048806/ ENSMUSG00000001123/ ENSMUSG00000067212/ ENSMUSG00000030157/ ENSMUSG00000045827/ ENSMUSG00000069255	8

GO:0016045	detection of bacterium	5/815	16/21092	0.0003	0.0051	0.0044	ENSMUSG00000078942/ ENSMUSG00000078945/ ENSMUSG00000071203/ ENSMUSG00000038058/ ENSMUSG00000027995	5
GO:0098543	detection of other organism	5/815	16/21092	0.0003	0.0051	0.0044	ENSMUSG00000078942/ ENSMUSG00000078945/ ENSMUSG00000071203/ ENSMUSG00000038058/ ENSMUSG00000027995	5
GO:1902187	negative regulation of viral release from host cell	5/815	16/21092	0.0003	0.0051	0.0044	ENSMUSG00000030966/ ENSMUSG00000036986/ ENSMUSG00000000275/ ENSMUSG00000024457/ ENSMUSG00000043279	5
GO:0050727	regulation of inflammatory response	26/815	319/21092	0.0003	0.0058	0.0050	ENSMUSG00000030107/ ENSMUSG00000001123/ ENSMUSG00000034218/ ENSMUSG00000031639/ ENSMUSG00000056220/ ENSMUSG00000023224/ ENSMUSG00000032508/ ENSMUSG00000055447/ ENSMUSG00000031948/ ENSMUSG00000032815/ ENSMUSG00000035042/ ENSMUSG00000025888/ ENSMUSG00000070056/ ENSMUSG00000035914/ ENSMUSG00000026365/ ENSMUSG00000002897/ ENSMUSG00000024810/ ENSMUSG00000105504/ ENSMUSG00000022126/ ENSMUSG00000034485/ ENSMUSG00000027995/ ENSMUSG00000008734/ ENSMUSG00000024789/ ENSMUSG00000020826/ ENSMUSG00000028599/ ENSMUSG00000025746	26
GO:0032652	regulation of interleukin−1 production	11/815	82/21092	0.0003	0.0058	0.0051	ENSMUSG00000036986/ ENSMUSG00000001123/ ENSMUSG00000071203/ ENSMUSG00000037860/ ENSMUSG00000022575/ ENSMUSG00000038058/ ENSMUSG00000025888/ ENSMUSG00000033538/ ENSMUSG00000027995/ ENSMUSG00000038418/ ENSMUSG00000024789	11
GO:0006925	inflammatory cell apoptotic process	6/815	25/21092	0.0003	0.0058	0.0051	ENSMUSG00000039217/ ENSMUSG00000035042/ ENSMUSG00000027737/ ENSMUSG00000045502/ ENSMUSG00000038855/ ENSMUSG00000025746	6
GO:0009595	detection of biotic stimulus	6/815	25/21092	0.0003	0.0058	0.0051	ENSMUSG00000078942/ ENSMUSG00000078945/ ENSMUSG00000071203/ ENSMUSG00000038058/ ENSMUSG00000025779/ ENSMUSG00000027995	6
GO:0031342	negative regulation of cell killing	6/815	25/21092	0.0003	0.0058	0.0051	ENSMUSG00000037321/ ENSMUSG00000024339/ ENSMUSG00000001123/ ENSMUSG00000067212/ ENSMUSG00000030157/ ENSMUSG00000045827	6
GO:1903649	regulation of cytoplasmic transport	6/815	25/21092	0.0003	0.0058	0.0051	ENSMUSG00000031207/ ENSMUSG00000004936/ ENSMUSG00000037062/ ENSMUSG00000042766/ ENSMUSG00000020132/ ENSMUSG00000026433	6

GO:0030260	entry into host cell	9/815	57/21092	0.0003	0.0058	0.0051	ENSMUSG00000025492/ ENSMUSG00000030966/ ENSMUSG00000000275/ ENSMUSG00000024457/ ENSMUSG00000002602/ ENSMUSG00000043279/ ENSMUSG00000035354/ ENSMUSG00000030921/ ENSMUSG00000062300	9
GO:0044409	entry into host	9/815	57/21092	0.0003	0.0058	0.0051	ENSMUSG00000025492/ ENSMUSG00000030966/ ENSMUSG00000000275/ ENSMUSG00000024457/ ENSMUSG00000002602/ ENSMUSG00000043279/ ENSMUSG00000035354/ ENSMUSG00000030921/ ENSMUSG00000062300	9
GO:0051806	entry into cell of other organism involved in symbiotic interaction	9/815	57/21092	0.0003	0.0058	0.0051	ENSMUSG00000025492/ ENSMUSG00000030966/ ENSMUSG00000000275/ ENSMUSG00000024457/ ENSMUSG00000002602/ ENSMUSG00000043279/ ENSMUSG00000035354/ ENSMUSG00000030921/ ENSMUSG00000062300	9
GO:0051828	entry into other organism involved in symbiotic interaction	9/815	57/21092	0.0003	0.0058	0.0051	ENSMUSG00000025492/ ENSMUSG00000030966/ ENSMUSG00000000275/ ENSMUSG00000024457/ ENSMUSG00000002602/ ENSMUSG00000043279/ ENSMUSG00000035354/ ENSMUSG00000030921/ ENSMUSG00000062300	9
GO:0043280	positive regulation of cysteine−type endopeptidase activity involved in apoptotic process	14/815	124/21092	0.0003	0.0058	0.0051	ENSMUSG00000026104/ ENSMUSG00000024066/ ENSMUSG00000064215/ ENSMUSG00000036986/ ENSMUSG00000021208/ ENSMUSG00000022346/ ENSMUSG00000000915/ ENSMUSG00000038058/ ENSMUSG00000039304/ ENSMUSG00000025887/ ENSMUSG00000025888/ ENSMUSG00000034485/ ENSMUSG00000033538/ ENSMUSG00000024789	14
GO:0048659	smooth muscle cell proliferation	17/815	170/21092	0.0003	0.0061	0.0053	ENSMUSG00000026104/ ENSMUSG00000020572/ ENSMUSG00000003032/ ENSMUSG00000032508/ ENSMUSG00000039217/ ENSMUSG00000022346/ ENSMUSG00000001642/ ENSMUSG00000040152/ ENSMUSG00000021796/ ENSMUSG00000052684/ ENSMUSG00000032501/ ENSMUSG00000035042/ ENSMUSG00000022206/ ENSMUSG00000031712/ ENSMUSG00000038418/ ENSMUSG00000024789/ ENSMUSG00000025746	17
GO:0042509	regulation of tyrosine phosphorylation of STAT protein	10/815	70/21092	0.0003	0.0063	0.0055	ENSMUSG00000034422/ ENSMUSG00000022906/ ENSMUSG00000038037/ ENSMUSG00000039217/ ENSMUSG00000035042/ ENSMUSG00000018899/ ENSMUSG00000031712/ ENSMUSG00000030895/ ENSMUSG00000024789/ ENSMUSG00000025746	10

GO:0000209	protein polyubiquitination	18/815	187/21092	0.0004	0.0065	0.0056	ENSMUSG00000049502/ ENSMUSG00000030966/ ENSMUSG00000026946/ ENSMUSG00000063268/ ENSMUSG00000042350/ ENSMUSG00000047098/ ENSMUSG00000072244/ ENSMUSG00000028793/ ENSMUSG00000023307/ ENSMUSG00000043279/ ENSMUSG00000060450/ ENSMUSG00000020387/ ENSMUSG00000024807/ ENSMUSG00000027466/ ENSMUSG00000078923/ ENSMUSG00000041263/ ENSMUSG00000047496/ ENSMUSG00000021774	18
GO:0022408	negative regulation of cell−cell adhesion	18/815	187/21092	0.0004	0.0065	0.0056	ENSMUSG00000048806/ ENSMUSG00000001123/ ENSMUSG00000016206/ ENSMUSG00000021795/ ENSMUSG00000038037/ ENSMUSG00000003032/ ENSMUSG00000004936/ ENSMUSG00000017446/ ENSMUSG00000042333/ ENSMUSG00000090958/ ENSMUSG00000016496/ ENSMUSG00000035914/ ENSMUSG00000056153/ ENSMUSG00000018899/ ENSMUSG00000026981/ ENSMUSG00000022676/ ENSMUSG00000069255/ ENSMUSG00000024789	18
GO:0008334	histone mRNA metabolic process	5/815	17/21092	0.0004	0.0065	0.0057	ENSMUSG00000034575/ ENSMUSG00000034218/ ENSMUSG00000032410/ ENSMUSG00000041781/ ENSMUSG00000024472	5
GO:0002698	negative regulation of immune effector process	14/815	126/21092	0.0004	0.0067	0.0058	ENSMUSG00000017830/ ENSMUSG00000037321/ ENSMUSG00000024339/ ENSMUSG00000048806/ ENSMUSG00000001123/ ENSMUSG00000067212/ ENSMUSG00000040987/ ENSMUSG00000030157/ ENSMUSG00000023224/ ENSMUSG00000000127/ ENSMUSG00000045827/ ENSMUSG00000024810/ ENSMUSG00000018899/ ENSMUSG00000069255	14
GO:2001056	positive regulation of cysteine−type endopeptidase activity	15/815	141/21092	0.0004	0.0067	0.0059	ENSMUSG00000026104/ ENSMUSG00000024066/ ENSMUSG00000064215/ ENSMUSG00000036986/ ENSMUSG00000021208/ ENSMUSG00000037860/ ENSMUSG00000022346/ ENSMUSG00000000915/ ENSMUSG00000038058/ ENSMUSG00000039304/ ENSMUSG00000025887/ ENSMUSG00000025888/ ENSMUSG00000034485/ ENSMUSG00000033538/ ENSMUSG00000024789	15
GO:0033029	regulation of neutrophil apoptotic process	4/815	10/21092	0.0004	0.0069	0.0060	ENSMUSG00000039217/ ENSMUSG00000027737/ ENSMUSG00000045502/ ENSMUSG00000038855	4

GO:0006402	mRNA catabolic process	19/815	206/21092	0.0004	0.0076	0.0066	ENSMUSG00000020464/ ENSMUSG00000002227/ ENSMUSG00000040613/ ENSMUSG00000027580/ ENSMUSG00000027233/ ENSMUSG00000029826/ ENSMUSG00000034575/ ENSMUSG00000034218/ ENSMUSG00000066800/ ENSMUSG00000035248/ ENSMUSG00000032508/ ENSMUSG00000042772/ ENSMUSG00000032410/ ENSMUSG00000037331/ ENSMUSG00000024472/ ENSMUSG00000000838/ ENSMUSG00000034724/ ENSMUSG00000034610/ ENSMUSG00000021127	19
GO:0007259	JAK−STAT cascade	17/815	175/21092	0.0005	0.0082	0.0071	ENSMUSG00000026104/ ENSMUSG00000034422/ ENSMUSG00000022906/ ENSMUSG00000048806/ ENSMUSG00000070904/ ENSMUSG00000064128/ ENSMUSG00000038037/ ENSMUSG00000000127/ ENSMUSG00000039217/ ENSMUSG00000001642/ ENSMUSG00000004040/ ENSMUSG00000035042/ ENSMUSG00000018899/ ENSMUSG00000031712/ ENSMUSG00000030895/ ENSMUSG00000024789/ ENSMUSG00000025746	17
GO:0034121	regulation of toll−like receptor signaling pathway	9/815	60/21092	0.0005	0.0083	0.0072	ENSMUSG00000025498/ ENSMUSG00000031639/ ENSMUSG00000073643/ ENSMUSG00000030921/ ENSMUSG00000070056/ ENSMUSG00000020641/ ENSMUSG00000022126/ ENSMUSG00000018899/ ENSMUSG00000027995	9
GO:0044764	multi−organism cellular process	7/815	37/21092	0.0005	0.0083	0.0072	ENSMUSG00000017830/ ENSMUSG00000026896/ ENSMUSG00000040296/ ENSMUSG00000029826/ ENSMUSG00000037921/ ENSMUSG00000055204/ ENSMUSG00000062300	7
GO:0061900	glial cell activation	7/815	37/21092	0.0005	0.0083	0.0072	ENSMUSG00000034218/ ENSMUSG00000031639/ ENSMUSG00000020572/ ENSMUSG00000052684/ ENSMUSG00000025888/ ENSMUSG00000024810/ ENSMUSG00000027995	7
GO:0002726	positive regulation of T cell cytokine production	5/815	18/21092	0.0005	0.0084	0.0073	ENSMUSG00000060802/ ENSMUSG00000037447/ ENSMUSG00000039217/ ENSMUSG00000020641/ ENSMUSG00000025746	5
GO:0032823	regulation of natural killer cell differentiation	5/815	18/21092	0.0005	0.0084	0.0073	ENSMUSG00000001123/ ENSMUSG00000002602/ ENSMUSG00000110206/ ENSMUSG00000018899/ ENSMUSG00000031712	5

GO:0039529	RIG−I signaling pathway	5/815	18/21092	0.0005	0.0084	0.0073	ENSMUSG00000017830/ ENSMUSG00000040296/ ENSMUSG00000029826/ ENSMUSG00000037921/ ENSMUSG00000055204	5
GO:0002724	regulation of T cell cytokine production	6/815	27/21092	0.0005	0.0084	0.0073	ENSMUSG00000060802/ ENSMUSG00000048806/ ENSMUSG00000037447/ ENSMUSG00000039217/ ENSMUSG00000020641/ ENSMUSG00000025746	6
GO:2000778	positive regulation of interleukin−6 secretion	6/815	27/21092	0.0005	0.0084	0.0073	ENSMUSG00000026896/ ENSMUSG00000040296/ ENSMUSG00000001123/ ENSMUSG00000036908/ ENSMUSG00000037447/ ENSMUSG00000022514	6
GO:0070665	positive regulation of leukocyte proliferation	15/815	145/21092	0.0005	0.0087	0.0076	ENSMUSG00000115338/ ENSMUSG00000027950/ ENSMUSG00000067212/ ENSMUSG00000000791/ ENSMUSG00000032508/ ENSMUSG00000039217/ ENSMUSG00000110206/ ENSMUSG00000066877/ ENSMUSG00000040329/ ENSMUSG00000016496/ ENSMUSG00000035042/ ENSMUSG00000035914/ ENSMUSG00000031712/ ENSMUSG00000014599/ ENSMUSG00000025746	15
GO:0006809	nitric oxide biosynthetic process	10/815	74/21092	0.0005	0.0092	0.0080	ENSMUSG00000016206/ ENSMUSG00000003032/ ENSMUSG00000055447/ ENSMUSG00000041736/ ENSMUSG00000031266/ ENSMUSG00000055148/ ENSMUSG00000027995/ ENSMUSG00000024789/ ENSMUSG00000020826/ ENSMUSG00000025746	10
GO:0071347	cellular response to interleukin−1	10/815	74/21092	0.0005	0.0092	0.0080	ENSMUSG00000064090/ ENSMUSG00000035042/ ENSMUSG00000035373/ ENSMUSG00000022126/ ENSMUSG00000018899/ ENSMUSG00000026981/ ENSMUSG00000038418/ ENSMUSG00000035385/ ENSMUSG00000040026/ ENSMUSG00000025746	10
GO:0072604	interleukin−6 secretion	7/815	38/21092	0.0006	0.0095	0.0082	ENSMUSG00000026896/ ENSMUSG00000040296/ ENSMUSG00000001123/ ENSMUSG00000036908/ ENSMUSG00000037447/ ENSMUSG00000022514/ ENSMUSG00000020826	7
GO:0150076	neuroinflammatory response	7/815	38/21092	0.0006	0.0095	0.0082	ENSMUSG00000034218/ ENSMUSG00000031639/ ENSMUSG00000020572/ ENSMUSG00000052684/ ENSMUSG00000025888/ ENSMUSG00000024810/ ENSMUSG00000027995	7
GO:0042035	regulation of cytokine biosynthetic process	12/815	102/21092	0.0006	0.0097	0.0084	ENSMUSG00000026946/ ENSMUSG00000031639/ ENSMUSG00000003032/ ENSMUSG00000032508/ ENSMUSG00000040152/ ENSMUSG00000020115/	12

							ENSMUSG00000015340/ ENSMUSG00000035914/ ENSMUSG00000018899/ ENSMUSG00000027995/ ENSMUSG00000038418/ ENSMUSG00000025746	
GO:0018212	peptidyl−tyrosine modification	25/815	315/21092	0.0006	0.0097	0.0084	ENSMUSG00000034422/ ENSMUSG00000022906/ ENSMUSG00000027293/ ENSMUSG00000020357/ ENSMUSG00000000791/ ENSMUSG00000045038/ ENSMUSG00000038037/ ENSMUSG00000000127/ ENSMUSG00000039217/ ENSMUSG00000021365/ ENSMUSG00000041936/ ENSMUSG00000034118/ ENSMUSG00000042333/ ENSMUSG00000062991/ ENSMUSG00000035042/ ENSMUSG00000015133/ ENSMUSG00000030681/ ENSMUSG00000018899/ ENSMUSG00000069255/ ENSMUSG00000031712/ ENSMUSG00000028289/ ENSMUSG00000008734/ ENSMUSG00000030895/ ENSMUSG00000024789/ ENSMUSG00000025746	25
GO:0050670	regulation of lymphocyte proliferation	20/815	228/21092	0.0006	0.0097	0.0084	ENSMUSG00000115338/ ENSMUSG00000027950/ ENSMUSG00000001123/ ENSMUSG00000067212/ ENSMUSG00000016206/ ENSMUSG00000034218/ ENSMUSG00000000791/ ENSMUSG00000021795/ ENSMUSG00000032508/ ENSMUSG00000039217/ ENSMUSG00000066877/ ENSMUSG00000042333/ ENSMUSG00000040329/ ENSMUSG00000090958/ ENSMUSG00000016496/ ENSMUSG00000035042/ ENSMUSG00000035914/ ENSMUSG00000018899/ ENSMUSG00000031712/ ENSMUSG00000025746	20
GO:0060340	positive regulation of type I interferon−mediated signaling pathway	4/815	11/21092	0.0006	0.0099	0.0086	ENSMUSG00000025498/ ENSMUSG00000072244/ ENSMUSG00000074151/ ENSMUSG00000027514	4
GO:0030224	monocyte differentiation	6/815	28/21092	0.0006	0.0099	0.0086	ENSMUSG00000022346/ ENSMUSG00000052684/ ENSMUSG00000031750/ ENSMUSG00000068758/ ENSMUSG00000014599/ ENSMUSG00000021127	6
GO:1903131	mononuclear cell differentiation	6/815	28/21092	0.0006	0.0099	0.0086	ENSMUSG00000022346/ ENSMUSG00000052684/ ENSMUSG00000031750/ ENSMUSG00000068758/ ENSMUSG00000014599/ ENSMUSG00000021127	6

GO:0007178	transmembrane receptor protein serine/threonine kinase signaling pathway	27/815	352/21092	0.0006	0.0099	0.0086	ENSMUSG00000036986/ ENSMUSG00000001123/ ENSMUSG00000032333/ ENSMUSG00000027204/ ENSMUSG00000021360/ ENSMUSG00000032508/ ENSMUSG00000020387/ ENSMUSG00000038400/ ENSMUSG00000040152/ ENSMUSG00000024238/ ENSMUSG00000047407/ ENSMUSG00000021796/ ENSMUSG00000052684/ ENSMUSG00000090958/ ENSMUSG00000029860/ ENSMUSG00000021065/ ENSMUSG00000021754/ ENSMUSG00000001870/ ENSMUSG00000092035/ ENSMUSG00000038508/ ENSMUSG00000069255/ ENSMUSG00000039153/ ENSMUSG00000038418/ ENSMUSG00000021250/ ENSMUSG00000035385/ ENSMUSG00000046186/ ENSMUSG00000027660	27
GO:0002573	myeloid leukocyte differentiation	19/815	212/21092	0.0006	0.0099	0.0086	ENSMUSG00000048806/ ENSMUSG00000027204/ ENSMUSG00000030157/ ENSMUSG00000027562/ ENSMUSG00000022346/ ENSMUSG00000004508/ ENSMUSG00000052684/ ENSMUSG00000032501/ ENSMUSG00000035042/ ENSMUSG00000015133/ ENSMUSG00000031750/ ENSMUSG00000068758/ ENSMUSG00000035186/ ENSMUSG00000027995/ ENSMUSG00000038301/ ENSMUSG00000014599/ ENSMUSG00000021250/ ENSMUSG00000021127/ ENSMUSG00000046186	19
GO:0045637	regulation of myeloid cell differentiation	19/815	212/21092	0.0006	0.0099	0.0086	ENSMUSG00000026104/ ENSMUSG00000060802/ ENSMUSG00000048806/ ENSMUSG00000027204/ ENSMUSG00000030157/ ENSMUSG00000014773/ ENSMUSG00000027562/ ENSMUSG00000032698/ ENSMUSG00000022346/ ENSMUSG00000004040/ ENSMUSG00000052684/ ENSMUSG00000032501/ ENSMUSG00000035692/ ENSMUSG00000035042/ ENSMUSG00000031750/ ENSMUSG00000038855/ ENSMUSG00000014599/ ENSMUSG00000021250/ ENSMUSG00000021127	19
GO:0051770	positive regulation of nitric−oxide synthase biosynthetic process	5/815	19/21092	0.0006	0.0104	0.0090	ENSMUSG00000026104/ ENSMUSG00000020572/ ENSMUSG00000027995/ ENSMUSG00000024789/ ENSMUSG00000035385	5
GO:0070555	response to interleukin−1	11/815	90/21092	0.0007	0.0113	0.0098	ENSMUSG00000032508/ ENSMUSG00000064090/ ENSMUSG00000035042/ ENSMUSG00000035373/ ENSMUSG00000022126/ ENSMUSG00000018899/	11

							ENSMUSG00000026981/ ENSMUSG00000038418/ ENSMUSG00000035385/ ENSMUSG00000040026/ ENSMUSG00000025746	
GO:0046209	nitric oxide metabolic process	10/815	77/21092	0.0007	0.0121	0.0105	ENSMUSG00000016206/ ENSMUSG00000003032/ ENSMUSG00000055447/ ENSMUSG00000041736/ ENSMUSG00000031266/ ENSMUSG00000055148/ ENSMUSG00000027995/ ENSMUSG00000024789/ ENSMUSG00000020826/ ENSMUSG00000025746	10
GO:0046633	alpha−beta T cell proliferation	7/815	40/21092	0.0008	0.0125	0.0108	ENSMUSG00000001123/ ENSMUSG00000067212/ ENSMUSG00000039217/ ENSMUSG00000042333/ ENSMUSG00000016496/ ENSMUSG00000018899/ ENSMUSG00000031712	7
GO:0010506	regulation of autophagy	20/815	234/21092	0.0008	0.0129	0.0112	ENSMUSG00000069874/ ENSMUSG00000030966/ ENSMUSG00000034218/ ENSMUSG00000020572/ ENSMUSG00000026672/ ENSMUSG00000021036/ ENSMUSG00000004936/ ENSMUSG00000046879/ ENSMUSG00000038467/ ENSMUSG00000041736/ ENSMUSG00000037331/ ENSMUSG00000020115/ ENSMUSG00000025241/ ENSMUSG00000038058/ ENSMUSG00000037062/ ENSMUSG00000054072/ ENSMUSG00000047496/ ENSMUSG00000025888/ ENSMUSG00000028211/ ENSMUSG00000027995	20
GO:0033002	muscle cell proliferation	20/815	234/21092	0.0008	0.0129	0.0112	ENSMUSG00000026104/ ENSMUSG00000020572/ ENSMUSG00000003032/ ENSMUSG00000032508/ ENSMUSG00000039217/ ENSMUSG00000022346/ ENSMUSG00000001642/ ENSMUSG00000022306/ ENSMUSG00000040152/ ENSMUSG00000021067/ ENSMUSG00000021796/ ENSMUSG00000004040/ ENSMUSG00000052684/ ENSMUSG00000032501/ ENSMUSG00000035042/ ENSMUSG00000022206/ ENSMUSG00000031712/ ENSMUSG00000038418/ ENSMUSG00000024789/ ENSMUSG00000025746	20
GO:0050708	regulation of protein secretion	33/815	472/21092	0.0008	0.0130	0.0113	ENSMUSG00000026896/ ENSMUSG00000040296/ ENSMUSG00000036986/ ENSMUSG00000001123/ ENSMUSG00000067212/ ENSMUSG00000036908/ ENSMUSG00000072244/ ENSMUSG00000045038/ ENSMUSG00000038037/ ENSMUSG00000037447/ ENSMUSG00000037860/ ENSMUSG00000022575/	33

							ENSMUSG00000022514/ ENSMUSG00000031154/ ENSMUSG00000042333/ ENSMUSG00000031948/ ENSMUSG00000090958/ ENSMUSG00000051790/ ENSMUSG00000016496/ ENSMUSG00000035042/ ENSMUSG00000024743/ ENSMUSG00000025888/ ENSMUSG00000035914/ ENSMUSG00000020641/ ENSMUSG00000034584/ ENSMUSG00000002897/ ENSMUSG00000024810/ ENSMUSG00000045502/ ENSMUSG00000033538/ ENSMUSG00000027995/ ENSMUSG00000024789/ ENSMUSG00000020826/ ENSMUSG00000025746	
GO:1904951	positive regulation of establishment of protein localization	32/815	453/21092	0.0008	0.0130	0.0113	ENSMUSG00000026896/ ENSMUSG00000040296/ ENSMUSG00000001123/ ENSMUSG00000067212/ ENSMUSG00000036908/ ENSMUSG00000072244/ ENSMUSG00000064128/ ENSMUSG00000045038/ ENSMUSG00000037447/ ENSMUSG00000037860/ ENSMUSG00000022575/ ENSMUSG00000022514/ ENSMUSG00000001416/ ENSMUSG00000042333/ ENSMUSG00000031948/ ENSMUSG00000037062/ ENSMUSG00000051790/ ENSMUSG00000016496/ ENSMUSG00000025888/ ENSMUSG00000026433/ ENSMUSG00000035914/ ENSMUSG00000034584/ ENSMUSG00000002897/ ENSMUSG00000024810/ ENSMUSG00000045502/ ENSMUSG00000034485/ ENSMUSG00000033538/ ENSMUSG00000027995/ ENSMUSG00000024789/ ENSMUSG00000035385/ ENSMUSG00000025746/ ENSMUSG00000032328	32
GO:0010759	positive regulation of macrophage chemotaxis	5/815	20/21092	0.0008	0.0130	0.0113	ENSMUSG00000040152/ ENSMUSG00000031948/ ENSMUSG00000035042/ ENSMUSG00000014599/ ENSMUSG00000035385	5
GO:0032616	interleukin^{-13} production	5/815	20/21092	0.0008	0.0130	0.0113	ENSMUSG00000067212/ ENSMUSG00000039217/ ENSMUSG00000022514/ ENSMUSG00000002897/ ENSMUSG00000024810	5
GO:0002793	positive regulation of peptide secretion	25/815	323/21092	0.0008	0.0131	0.0114	ENSMUSG00000026896/ ENSMUSG00000040296/ ENSMUSG00000001123/ ENSMUSG00000067212/ ENSMUSG00000036908/ ENSMUSG00000072244/ ENSMUSG00000045038/ ENSMUSG00000037447/ ENSMUSG00000037860/ ENSMUSG00000022575/ ENSMUSG00000022514/ ENSMUSG00000042333/ ENSMUSG00000031948/ ENSMUSG00000051790/ ENSMUSG00000016496/	25

							ENSMUSG00000025888/ ENSMUSG00000035914/ ENSMUSG00000034584/ ENSMUSG00000002897/ ENSMUSG00000024810/ ENSMUSG00000045502/ ENSMUSG00000033538/ ENSMUSG00000027995/ ENSMUSG00000024789/ ENSMUSG00000025746	
GO:0042108	positive regulation of cytokine biosynthetic process	9/815	65/21092	0.0008	0.0133	0.0116	ENSMUSG00000031639/ ENSMUSG00000032508/ ENSMUSG00000040152/ ENSMUSG00000020115/ ENSMUSG00000015340/ ENSMUSG00000035914/ ENSMUSG00000018899/ ENSMUSG00000027995/ ENSMUSG00000038418	9
GO:0010826	negative regulation of centrosome duplication	4/815	12/21092	0.0009	0.0133	0.0116	ENSMUSG00000049488/ ENSMUSG00000020918/ ENSMUSG00000000708/ ENSMUSG00000072082	4
GO:0032825	positive regulation of natural killer cell differentiation	4/815	12/21092	0.0009	0.0133	0.0116	ENSMUSG00000002602/ ENSMUSG00000110206/ ENSMUSG00000018899/ ENSMUSG00000031712	4
GO:0042033	chemokine biosynthetic process	4/815	12/21092	0.0009	0.0133	0.0116	ENSMUSG00000031639/ ENSMUSG00000032508/ ENSMUSG00000038418/ ENSMUSG00000025746	4
GO:0045073	regulation of chemokine biosynthetic process	4/815	12/21092	0.0009	0.0133	0.0116	ENSMUSG00000031639/ ENSMUSG00000032508/ ENSMUSG00000038418/ ENSMUSG00000025746	4
GO:0071044	histone mRNA catabolic process	4/815	12/21092	0.0009	0.0133	0.0116	ENSMUSG00000034575/ ENSMUSG00000034218/ ENSMUSG00000032410/ ENSMUSG00000024472	4
GO:0006958	complement activation. classical pathway	6/815	30/21092	0.0009	0.0136	0.0118	ENSMUSG00000024371/ ENSMUSG00000023224/ ENSMUSG00000055172/ ENSMUSG00000079343/ ENSMUSG00000026405/ ENSMUSG00000038521	6
GO:0032660	regulation of interleukin−17 production	6/815	30/21092	0.0009	0.0136	0.0118	ENSMUSG00000037447/ ENSMUSG00000032508/ ENSMUSG00000039217/ ENSMUSG00000031154/ ENSMUSG00000027995/ ENSMUSG00000031712	6
GO:0045428	regulation of nitric oxide biosynthetic process	9/815	66/21092	0.0009	0.0146	0.0127	ENSMUSG00000016206/ ENSMUSG00000003032/ ENSMUSG00000055447/ ENSMUSG00000041736/ ENSMUSG00000031266/ ENSMUSG00000055148/ ENSMUSG00000027995/ ENSMUSG00000024789/ ENSMUSG00000025746	9
GO:0050730	regulation of peptidyl−tyrosine phosphorylation	21/815	255/21092	0.0010	0.0148	0.0129	ENSMUSG00000034422/ ENSMUSG00000022906/ ENSMUSG00000027293/ ENSMUSG00000045038/ ENSMUSG00000038037/ ENSMUSG00000039217/ ENSMUSG00000021365/ ENSMUSG00000041936/ ENSMUSG00000042333/	21

							ENSMUSG00000062991/ ENSMUSG00000035042/ ENSMUSG00000015133/ ENSMUSG00000030681/ ENSMUSG00000018899/ ENSMUSG00000069255/ ENSMUSG00000031712/ ENSMUSG00000028289/ ENSMUSG00000008734/ ENSMUSG00000030895/ ENSMUSG00000024789/ ENSMUSG00000025746	
GO:0032946	positive regulation of mononuclear cell proliferation	14/815	139/21092	0.0010	0.0152	0.0132	ENSMUSG00000115338/ ENSMUSG00000027950/ ENSMUSG00000067212/ ENSMUSG00000000791/ ENSMUSG00000032508/ ENSMUSG00000039217/ ENSMUSG00000066877/ ENSMUSG00000040329/ ENSMUSG00000016496/ ENSMUSG00000035042/ ENSMUSG00000035914/ ENSMUSG00000031712/ ENSMUSG00000014599/ ENSMUSG00000025746	14
GO:0045582	positive regulation of T cell differentiation	11/815	94/21092	0.0010	0.0152	0.0132	ENSMUSG00000115338/ ENSMUSG00000001123/ ENSMUSG00000016206/ ENSMUSG00000038037/ ENSMUSG00000039217/ ENSMUSG00000040329/ ENSMUSG00000031304/ ENSMUSG00000037440/ ENSMUSG00000038855/ ENSMUSG00000018899/ ENSMUSG00000025746	11
GO:0050729	positive regulation of inflammatory response	13/815	124/21092	0.0010	0.0157	0.0136	ENSMUSG00000031639/ ENSMUSG00000056220/ ENSMUSG00000032508/ ENSMUSG00000055447/ ENSMUSG00000031948/ ENSMUSG00000035042/ ENSMUSG00000002897/ ENSMUSG00000024810/ ENSMUSG00000105504/ ENSMUSG00000027995/ ENSMUSG00000008734/ ENSMUSG00000024789/ ENSMUSG00000025746	13
GO:0071359	cellular response to dsRNA	5/815	21/21092	0.0010	0.0157	0.0136	ENSMUSG00000026896/ ENSMUSG00000040296/ ENSMUSG00000029826/ ENSMUSG00000048806/ ENSMUSG00000031639	5
GO:0002704	negative regulation of leukocyte mediated immunity	8/815	54/21092	0.0010	0.0157	0.0136	ENSMUSG00000037321/ ENSMUSG00000024339/ ENSMUSG00000048806/ ENSMUSG00000001123/ ENSMUSG00000067212/ ENSMUSG00000030157/ ENSMUSG00000045827/ ENSMUSG00000069255	8
GO:0002440	production of molecular mediator of immune response	18/815	205/21092	0.0010	0.0158	0.0137	ENSMUSG00000027639/ ENSMUSG00000040296/ ENSMUSG00000060802/ ENSMUSG00000048806/ ENSMUSG00000067212/ ENSMUSG00000034218/ ENSMUSG00000031639/ ENSMUSG00000072244/ ENSMUSG00000037447/ ENSMUSG00000039217/ ENSMUSG00000042333/ ENSMUSG00000031948/	18

							ENSMUSG00000020641/ ENSMUSG00000024810/ ENSMUSG00000027995/ ENSMUSG00000008734/ ENSMUSG00000030895/ ENSMUSG00000025746	
GO:0002828	regulation of type 2 immune response	6/815	31/21092	0.0011	0.0158	0.0137	ENSMUSG00000048806/ ENSMUSG00000039217/ ENSMUSG00000020641/ ENSMUSG00000024810/ ENSMUSG00000018899/ ENSMUSG00000025746	6
GO:0032637	interleukin−8 production	9/815	67/21092	0.0011	0.0158	0.0137	ENSMUSG00000040296/ ENSMUSG00000031639/ ENSMUSG00000003032/ ENSMUSG00000032508/ ENSMUSG00000039217/ ENSMUSG00000038058/ ENSMUSG00000041187/ ENSMUSG00000027995/ ENSMUSG00000020826	9
GO:0045639	positive regulation of myeloid cell differentiation	11/815	95/21092	0.0011	0.0163	0.0142	ENSMUSG00000026104/ ENSMUSG00000027562/ ENSMUSG00000004040/ ENSMUSG00000052684/ ENSMUSG00000032501/ ENSMUSG00000035692/ ENSMUSG00000035042/ ENSMUSG00000031750/ ENSMUSG00000014599/ ENSMUSG00000021250/ ENSMUSG00000021127	11
GO:0018108	peptidyl−tyrosine phosphorylation	24/815	312/21092	0.0011	0.0168	0.0146	ENSMUSG00000034422/ ENSMUSG00000022906/ ENSMUSG00000027293/ ENSMUSG00000020357/ ENSMUSG00000000791/ ENSMUSG00000045038/ ENSMUSG00000038037/ ENSMUSG00000000127/ ENSMUSG00000039217/ ENSMUSG00000021365/ ENSMUSG00000041936/ ENSMUSG00000042333/ ENSMUSG00000062991/ ENSMUSG00000035042/ ENSMUSG00000015133/ ENSMUSG00000030681/ ENSMUSG00000018899/ ENSMUSG00000069255/ ENSMUSG00000031712/ ENSMUSG00000028289/ ENSMUSG00000008734/ ENSMUSG00000030895/ ENSMUSG00000024789/ ENSMUSG00000025746	24
GO:0042531	positive regulation of tyrosine phosphorylation of STAT protein	8/815	55/21092	0.0012	0.0174	0.0151	ENSMUSG00000034422/ ENSMUSG00000022906/ ENSMUSG00000039217/ ENSMUSG00000035042/ ENSMUSG00000031712/ ENSMUSG00000030895/ ENSMUSG00000024789/ ENSMUSG00000025746	8
GO:0097193	intrinsic apoptotic signaling pathway	23/815	295/21092	0.0012	0.0175	0.0152	ENSMUSG00000025647/ ENSMUSG00000115338/ ENSMUSG00000036986/ ENSMUSG00000034218/ ENSMUSG00000021208/ ENSMUSG00000022346/ ENSMUSG00000073489/ ENSMUSG00000022564/ ENSMUSG00000027006/ ENSMUSG00000004936/ ENSMUSG00000066877/ ENSMUSG00000024807/ ENSMUSG00000051413/ ENSMUSG00000037062/ ENSMUSG00000025887/	23

							ENSMUSG00000037440/ ENSMUSG00000034485/ ENSMUSG00000033538/ ENSMUSG00000030717/ ENSMUSG00000022676/ ENSMUSG00000024789/ ENSMUSG00000028599/ ENSMUSG00000027660	
GO:0035710	CD4−positive. alpha−beta T cell activation	11/815	96/21092	0.0012	0.0175	0.0152	ENSMUSG00000001123/ ENSMUSG00000038037/ ENSMUSG00000039217/ ENSMUSG00000031154/ ENSMUSG00000004040/ ENSMUSG00000031304/ ENSMUSG00000016496/ ENSMUSG00000021453/ ENSMUSG00000020641/ ENSMUSG00000018899/ ENSMUSG00000025746	11
GO:0002455	humoral immune response mediated by circulating immunoglobulin	7/815	43/21092	0.0012	0.0176	0.0153	ENSMUSG00000024371/ ENSMUSG00000023224/ ENSMUSG00000055172/ ENSMUSG00000079343/ ENSMUSG00000026405/ ENSMUSG00000038521/ ENSMUSG00000030895	7
GO:0042129	regulation of T cell proliferation	16/815	174/21092	0.0012	0.0177	0.0153	ENSMUSG00000115338/ ENSMUSG00000001123/ ENSMUSG00000067212/ ENSMUSG00000016206/ ENSMUSG00000000791/ ENSMUSG00000021795/ ENSMUSG00000039217/ ENSMUSG00000066877/ ENSMUSG00000042333/ ENSMUSG00000090958/ ENSMUSG00000016496/ ENSMUSG00000035042/ ENSMUSG00000035914/ ENSMUSG00000018899/ ENSMUSG00000031712/ ENSMUSG00000025746	16
GO:2001057	reactive nitrogen species metabolic process	10/815	82/21092	0.0012	0.0178	0.0154	ENSMUSG00000016206/ ENSMUSG00000003032/ ENSMUSG00000055447/ ENSMUSG00000041736/ ENSMUSG00000031266/ ENSMUSG00000055148/ ENSMUSG00000027995/ ENSMUSG00000024789/ ENSMUSG00000020826/ ENSMUSG00000025746	10
GO:0007050	cell cycle arrest	14/815	142/21092	0.0012	0.0179	0.0155	ENSMUSG00000020464/ ENSMUSG00000036986/ ENSMUSG00000034218/ ENSMUSG00000011114/ ENSMUSG00000029863/ ENSMUSG00000022346/ ENSMUSG00000045795/ ENSMUSG00000004936/ ENSMUSG00000040152/ ENSMUSG00000029207/ ENSMUSG00000028211/ ENSMUSG00000018899/ ENSMUSG00000035131/ ENSMUSG00000027660	14
GO:0002366	leukocyte activation involved in immune response	21/815	260/21092	0.0012	0.0179	0.0155	ENSMUSG00000048806/ ENSMUSG00000001123/ ENSMUSG00000016206/ ENSMUSG00000070904/ ENSMUSG00000057554/ ENSMUSG00000014773/	21

							ENSMUSG00000045038/ ENSMUSG00000000127/ ENSMUSG00000039217/ ENSMUSG00000028217/ ENSMUSG00000025178/ ENSMUSG00000031154/ ENSMUSG00000004508/ ENSMUSG00000004040/ ENSMUSG00000031948/ ENSMUSG00000021453/ ENSMUSG00000026797/ ENSMUSG00000024810/ ENSMUSG00000018899/ ENSMUSG00000031093/ ENSMUSG00000025746	
GO:1902186	regulation of viral release from host cell	6/815	32/21092	0.0013	0.0182	0.0158	ENSMUSG00000030966/ ENSMUSG00000036986/ ENSMUSG00000000275/ ENSMUSG00000024457/ ENSMUSG00000043279/ ENSMUSG00000038467	6
GO:0032897	negative regulation of viral transcription	5/815	22/21092	0.0013	0.0187	0.0162	ENSMUSG00000025492/ ENSMUSG00000030966/ ENSMUSG00000039853/ ENSMUSG00000052684/ ENSMUSG00000035042	5
GO:0035743	CD4−positive. alpha−beta T cell cytokine production	5/815	22/21092	0.0013	0.0187	0.0162	ENSMUSG00000048806/ ENSMUSG00000037447/ ENSMUSG00000039217/ ENSMUSG00000020641/ ENSMUSG00000025746	5
GO:1903409	reactive oxygen species biosynthetic process	12/815	112/21092	0.0013	0.0188	0.0164	ENSMUSG00000016206/ ENSMUSG00000003032/ ENSMUSG00000055447/ ENSMUSG00000041736/ ENSMUSG00000004040/ ENSMUSG00000031266/ ENSMUSG00000055148/ ENSMUSG00000015340/ ENSMUSG00000027995/ ENSMUSG00000024789/ ENSMUSG00000020826/ ENSMUSG00000025746	12
GO:0033674	positive regulation of kinase activity	32/815	467/21092	0.0013	0.0191	0.0166	ENSMUSG00000069874/ ENSMUSG00000012519/ ENSMUSG00000024079/ ENSMUSG00000002307/ ENSMUSG00000000184/ ENSMUSG00000061288/ ENSMUSG00000038037/ ENSMUSG00000022505/ ENSMUSG00000039217/ ENSMUSG00000021365/ ENSMUSG00000041936/ ENSMUSG00000004936/ ENSMUSG00000040152/ ENSMUSG00000045328/ ENSMUSG00000028266/ ENSMUSG00000031948/ ENSMUSG00000037062/ ENSMUSG00000062991/ ENSMUSG00000070871/ ENSMUSG00000021453/ ENSMUSG00000010538/ ENSMUSG00000021754/ ENSMUSG00000032740/ ENSMUSG00000026872/ ENSMUSG00000026981/ ENSMUSG00000038508/ ENSMUSG00000014599/ ENSMUSG00000038418/ ENSMUSG00000008734/ ENSMUSG00000024235/ ENSMUSG00000024789/ ENSMUSG00000034165	32

GO:0045860	positive regulation of protein kinase activity	30/815	430/21092	0.0014	0.0201	0.0175	ENSMUSG00000069874/ ENSMUSG00000012519/ ENSMUSG00000024079/ ENSMUSG00000002307/ ENSMUSG00000000184/ ENSMUSG00000061288/ ENSMUSG00000038037/ ENSMUSG00000022505/ ENSMUSG00000039217/ ENSMUSG00000021365/ ENSMUSG00000041936/ ENSMUSG00000004936/ ENSMUSG00000040152/ ENSMUSG00000045328/ ENSMUSG00000031948/ ENSMUSG00000062991/ ENSMUSG00000070871/ ENSMUSG00000021453/ ENSMUSG00000010538/ ENSMUSG00000021754/ ENSMUSG00000032740/ ENSMUSG00000026872/ ENSMUSG00000026981/ ENSMUSG00000038508/ ENSMUSG00000014599/ ENSMUSG00000038418/ ENSMUSG00000008734/ ENSMUSG00000024235/ ENSMUSG00000024789/ ENSMUSG00000034165	30
GO:0002263	cell activation involved in immune response	21/815	264/21092	0.0015	0.0211	0.0184	ENSMUSG00000048806/ ENSMUSG00000001123/ ENSMUSG00000016206/ ENSMUSG00000070904/ ENSMUSG00000057554/ ENSMUSG00000014773/ ENSMUSG00000045038/ ENSMUSG00000000127/ ENSMUSG00000039217/ ENSMUSG00000028217/ ENSMUSG00000025178/ ENSMUSG00000031154/ ENSMUSG00000004508/ ENSMUSG00000004040/ ENSMUSG00000031948/ ENSMUSG00000021453/ ENSMUSG00000026797/ ENSMUSG00000024810/ ENSMUSG00000018899/ ENSMUSG00000031093/ ENSMUSG00000025746	21
GO:0046427	positive regulation of JAK−STAT cascade	11/815	99/21092	0.0015	0.0217	0.0189	ENSMUSG00000034422/ ENSMUSG00000022906/ ENSMUSG00000064128/ ENSMUSG00000038037/ ENSMUSG00000039217/ ENSMUSG00000001642/ ENSMUSG00000035042/ ENSMUSG00000031712/ ENSMUSG00000030895/ ENSMUSG00000024789/ ENSMUSG00000025746	11
GO:1905523	positive regulation of macrophage migration	5/815	23/21092	0.0016	0.0225	0.0196	ENSMUSG00000040152/ ENSMUSG00000031948/ ENSMUSG00000035042/ ENSMUSG00000014599/ ENSMUSG00000035385	5
GO:0006857	oligopeptide transport	4/815	14/21092	0.0016	0.0225	0.0196	ENSMUSG00000027562/ ENSMUSG00000028217/ ENSMUSG00000025790/ ENSMUSG00000027737	4
GO:0032736	positive regulation of interleukin−13 production	4/815	14/21092	0.0016	0.0225	0.0196	ENSMUSG00000067212/ ENSMUSG00000022514/ ENSMUSG00000002897/ ENSMUSG00000024810	4

GO:0045591	positive regulation of regulatory T cell differentiation	4/815	14/21092	0.0016	0.0225	0.0196	ENSMUSG00000001123/ ENSMUSG00000016206/ ENSMUSG00000038037/ ENSMUSG00000031304	4
GO:0046606	negative regulation of centrosome cycle	4/815	14/21092	0.0016	0.0225	0.0196	ENSMUSG00000049488/ ENSMUSG00000020918/ ENSMUSG00000000708/ ENSMUSG00000072082	4
GO:0050862	positive regulation of T cell receptor signaling pathway	4/815	14/21092	0.0016	0.0225	0.0196	ENSMUSG00000029640/ ENSMUSG00000041187/ ENSMUSG00000026433/ ENSMUSG00000062300	4
GO:0070102	interleukin−6−mediated signaling pathway	4/815	14/21092	0.0016	0.0225	0.0196	ENSMUSG00000000127/ ENSMUSG00000004040/ ENSMUSG00000034610/ ENSMUSG00000025746	4
GO:0071360	cellular response to exogenous dsRNA	4/815	14/21092	0.0016	0.0225	0.0196	ENSMUSG00000026896/ ENSMUSG00000040296/ ENSMUSG00000029826/ ENSMUSG00000031639	4
GO:0019076	viral release from host cell	6/815	34/21092	0.0017	0.0238	0.0207	ENSMUSG00000030966/ ENSMUSG00000036986/ ENSMUSG00000000275/ ENSMUSG00000024457/ ENSMUSG00000043279/ ENSMUSG00000038467	6
GO:0035890	exit from host	6/815	34/21092	0.0017	0.0238	0.0207	ENSMUSG00000030966/ ENSMUSG00000036986/ ENSMUSG00000000275/ ENSMUSG00000024457/ ENSMUSG00000043279/ ENSMUSG00000038467	6
GO:0035891	exit from host cell	6/815	34/21092	0.0017	0.0238	0.0207	ENSMUSG00000030966/ ENSMUSG00000036986/ ENSMUSG00000000275/ ENSMUSG00000024457/ ENSMUSG00000043279/ ENSMUSG00000038467	6
GO:0052126	movement in host environment	6/815	34/21092	0.0017	0.0238	0.0207	ENSMUSG00000030966/ ENSMUSG00000036986/ ENSMUSG00000000275/ ENSMUSG00000024457/ ENSMUSG00000043279/ ENSMUSG00000038467	6
GO:0052192	movement in environment of other organism involved in symbiotic interaction	6/815	34/21092	0.0017	0.0238	0.0207	ENSMUSG00000030966/ ENSMUSG00000036986/ ENSMUSG00000000275/ ENSMUSG00000024457/ ENSMUSG00000043279/ ENSMUSG00000038467	6
GO:0002526	acute inflammatory response	12/815	116/21092	0.0018	0.0242	0.0210	ENSMUSG00000023224/ ENSMUSG00000045827/ ENSMUSG00000004040/ ENSMUSG00000039196/ ENSMUSG00000029869/ ENSMUSG00000035042/ ENSMUSG00000026365/ ENSMUSG00000037440/ ENSMUSG00000030717/ ENSMUSG00000026981/ ENSMUSG00000040026/ ENSMUSG00000025746	12
GO:0016241	regulation of macroautophagy	11/815	101/21092	0.0018	0.0245	0.0212	ENSMUSG00000069874/ ENSMUSG00000026672/ ENSMUSG00000021036/ ENSMUSG00000046879/ ENSMUSG00000038467/ ENSMUSG00000037331/	11

							ENSMUSG00000020115/ ENSMUSG00000025241/ ENSMUSG00000038058/ ENSMUSG00000037062/ ENSMUSG00000027995	
GO:2001235	positive regulation of apoptotic signaling pathway	16/815	181/21092	0.0018	0.0246	0.0213	ENSMUSG00000002307/ ENSMUSG00000036986/ ENSMUSG00000048806/ ENSMUSG00000029863/ ENSMUSG00000022346/ ENSMUSG00000000915/ ENSMUSG00000040152/ ENSMUSG00000066877/ ENSMUSG00000027466/ ENSMUSG00000051413/ ENSMUSG00000039304/ ENSMUSG00000037062/ ENSMUSG00000028211/ ENSMUSG00000026628/ ENSMUSG00000024789/ ENSMUSG00000027660	16
GO:0042136	neurotransmitter biosynthetic process	11/815	102/21092	0.0019	0.0262	0.0228	ENSMUSG00000016206/ ENSMUSG00000037580/ ENSMUSG00000003032/ ENSMUSG00000055447/ ENSMUSG00000041736/ ENSMUSG00000031266/ ENSMUSG00000055148/ ENSMUSG00000027995/ ENSMUSG00000024789/ ENSMUSG00000020826/ ENSMUSG00000025746	11
GO:0046634	regulation of alpha−beta T cell activation	11/815	102/21092	0.0019	0.0262	0.0228	ENSMUSG00000115338/ ENSMUSG00000001123/ ENSMUSG00000067212/ ENSMUSG00000038037/ ENSMUSG00000039217/ ENSMUSG00000042333/ ENSMUSG00000031304/ ENSMUSG00000016496/ ENSMUSG00000038855/ ENSMUSG00000018899/ ENSMUSG00000025746	11
GO:1903038	negative regulation of leukocyte cell−cell adhesion	13/815	133/21092	0.0020	0.0262	0.0228	ENSMUSG00000048806/ ENSMUSG00000001123/ ENSMUSG00000016206/ ENSMUSG00000021795/ ENSMUSG00000038037/ ENSMUSG00000003032/ ENSMUSG00000042333/ ENSMUSG00000090958/ ENSMUSG00000016496/ ENSMUSG00000035914/ ENSMUSG00000056153/ ENSMUSG00000018899/ ENSMUSG00000069255	13
GO:1904892	regulation of STAT cascade	15/815	166/21092	0.0020	0.0269	0.0233	ENSMUSG00000034422/ ENSMUSG00000022906/ ENSMUSG00000048806/ ENSMUSG00000070904/ ENSMUSG00000064128/ ENSMUSG00000038037/ ENSMUSG00000039217/ ENSMUSG00000001642/ ENSMUSG00000053141/ ENSMUSG00000035042/ ENSMUSG00000018899/ ENSMUSG00000031712/ ENSMUSG00000030895/ ENSMUSG00000024789/ ENSMUSG00000025746	15

GO:0050702	interleukin−1 beta secretion	7/815	47/21092	0.0020	0.0273	0.0238	ENSMUSG00000036986/ ENSMUSG00000037860/ ENSMUSG00000022575/ ENSMUSG00000025888/ ENSMUSG00000105504/ ENSMUSG00000033538/ ENSMUSG00000027995	7
GO:0002763	positive regulation of myeloid leukocyte differentiation	8/815	60/21092	0.0021	0.0277	0.0240	ENSMUSG00000027562/ ENSMUSG00000052684/ ENSMUSG00000032501/ ENSMUSG00000035042/ ENSMUSG00000031750/ ENSMUSG00000014599/ ENSMUSG00000021250/ ENSMUSG00000021127	8
GO:1904894	positive regulation of STAT cascade	11/815	103/21092	0.0021	0.0280	0.0243	ENSMUSG00000034422/ ENSMUSG00000022906/ ENSMUSG00000064128/ ENSMUSG00000038037/ ENSMUSG00000039217/ ENSMUSG00000001642/ ENSMUSG00000035042/ ENSMUSG00000031712/ ENSMUSG00000030895/ ENSMUSG00000024789/ ENSMUSG00000025746	11
GO:0032740	positive regulation of interleukin−17 production	4/815	15/21092	0.0021	0.0282	0.0245	ENSMUSG00000037447/ ENSMUSG00000032508/ ENSMUSG00000039217/ ENSMUSG00000031712	4
GO:0039535	regulation of RIG−I signaling pathway	4/815	15/21092	0.0021	0.0282	0.0245	ENSMUSG00000017830/ ENSMUSG00000029826/ ENSMUSG00000037921/ ENSMUSG00000055204	4
GO:0042095	interferon−gamma biosynthetic process	4/815	15/21092	0.0021	0.0282	0.0245	ENSMUSG00000031639/ ENSMUSG00000039217/ ENSMUSG00000021453/ ENSMUSG00000035914	4
GO:0042448	progesterone metabolic process	4/815	15/21092	0.0021	0.0282	0.0245	ENSMUSG00000033715/ ENSMUSG00000054757/ ENSMUSG00000027068/ ENSMUSG00000038418	4
GO:0090398	cellular senescence	8/815	61/21092	0.0023	0.0303	0.0263	ENSMUSG00000020464/ ENSMUSG00000036986/ ENSMUSG00000027641/ ENSMUSG00000020572/ ENSMUSG00000019699/ ENSMUSG00000004936/ ENSMUSG00000031540/ ENSMUSG00000021796	8
GO:0042088	T−helper 1 type immune response	7/815	48/21092	0.0023	0.0303	0.0263	ENSMUSG00000000791/ ENSMUSG00000037447/ ENSMUSG00000039217/ ENSMUSG00000070427/ ENSMUSG00000021453/ ENSMUSG00000024810/ ENSMUSG00000018899	7
GO:0051250	negative regulation of lymphocyte activation	14/815	152/21092	0.0023	0.0304	0.0264	ENSMUSG00000048806/ ENSMUSG00000001123/ ENSMUSG00000016206/ ENSMUSG00000034218/ ENSMUSG00000002602/ ENSMUSG00000021795/ ENSMUSG00000038037/ ENSMUSG00000042333/ ENSMUSG00000090958/	14

							ENSMUSG00000016496/ ENSMUSG00000035914/ ENSMUSG00000056153/ ENSMUSG00000018899/ ENSMUSG00000069255	
GO:0050868	negative regulation of T cell activation	12/815	120/21092	0.0024	0.0306	0.0266	ENSMUSG00000048806/ ENSMUSG00000001123/ ENSMUSG00000016206/ ENSMUSG00000021795/ ENSMUSG00000038037/ ENSMUSG00000042333/ ENSMUSG00000090958/ ENSMUSG00000016496/ ENSMUSG00000035914/ ENSMUSG00000056153/ ENSMUSG00000018899/ ENSMUSG00000069255	12
GO:0034123	positive regulation of toll−like receptor signaling pathway	5/815	25/21092	0.0024	0.0306	0.0266	ENSMUSG00000031639/ ENSMUSG00000073643/ ENSMUSG00000070056/ ENSMUSG00000020641/ ENSMUSG00000027995	5
GO:0051767	nitric−oxide synthase biosynthetic process	5/815	25/21092	0.0024	0.0306	0.0266	ENSMUSG00000026104/ ENSMUSG00000020572/ ENSMUSG00000027995/ ENSMUSG00000024789/ ENSMUSG00000035385	5
GO:0051769	regulation of nitric−oxide synthase biosynthetic process	5/815	25/21092	0.0024	0.0306	0.0266	ENSMUSG00000026104/ ENSMUSG00000020572/ ENSMUSG00000027995/ ENSMUSG00000024789/ ENSMUSG00000035385	5
GO:1903532	positive regulation of secretion by cell	31/815	465/21092	0.0024	0.0308	0.0268	ENSMUSG00000026896/ ENSMUSG00000040296/ ENSMUSG00000027950/ ENSMUSG00000001123/ ENSMUSG00000067212/ ENSMUSG00000036908/ ENSMUSG00000072244/ ENSMUSG00000045038/ ENSMUSG00000037447/ ENSMUSG00000037860/ ENSMUSG00000022575/ ENSMUSG00000022514/ ENSMUSG00000017446/ ENSMUSG00000004508/ ENSMUSG00000042333/ ENSMUSG00000031948/ ENSMUSG00000051790/ ENSMUSG00000016496/ ENSMUSG00000024743/ ENSMUSG00000025888/ ENSMUSG00000035914/ ENSMUSG00000026797/ ENSMUSG00000034584/ ENSMUSG00000002897/ ENSMUSG00000024810/ ENSMUSG00000045502/ ENSMUSG00000030659/ ENSMUSG00000033538/ ENSMUSG00000027995/ ENSMUSG00000024789/ ENSMUSG00000025746	31
GO:0043254	regulation of protein complex assembly	30/815	446/21092	0.0024	0.0314	0.0273	ENSMUSG00000069874/ ENSMUSG00000024079/ ENSMUSG00000027233/ ENSMUSG00000040483/ ENSMUSG00000034218/ ENSMUSG00000045038/ ENSMUSG00000057058/ ENSMUSG00000002233/ ENSMUSG00000000127/ ENSMUSG00000037860/ ENSMUSG00000000915/ ENSMUSG00000031207/	30

							ENSMUSG00000021676/ ENSMUSG00000015733/ ENSMUSG00000028217/ ENSMUSG00000021266/ ENSMUSG00000016534/ ENSMUSG00000038400/ ENSMUSG00000045795/ ENSMUSG00000066877/ ENSMUSG00000058624/ ENSMUSG00000028266/ ENSMUSG00000052684/ ENSMUSG00000037062/ ENSMUSG00000034724/ ENSMUSG00000030921/ ENSMUSG00000021754/ ENSMUSG00000026797/ ENSMUSG00000105504/ ENSMUSG00000027995	
GO:0032874	positive regulation of stress−activated MAPK cascade	15/815	170/21092	0.0025	0.0323	0.0280	ENSMUSG00000012519/ ENSMUSG00000024079/ ENSMUSG00000024066/ ENSMUSG00000031639/ ENSMUSG00000020357/ ENSMUSG00000061288/ ENSMUSG00000032508/ ENSMUSG00000031948/ ENSMUSG00000038058/ ENSMUSG00000021453/ ENSMUSG00000070056/ ENSMUSG00000021754/ ENSMUSG00000026872/ ENSMUSG00000026981/ ENSMUSG00000069255	15
GO:0034101	erythrocyte homeostasis	13/815	137/21092	0.0025	0.0323	0.0280	ENSMUSG00000026104/ ENSMUSG00000027951/ ENSMUSG00000060802/ ENSMUSG00000002602/ ENSMUSG00000032698/ ENSMUSG00000004040/ ENSMUSG00000035692/ ENSMUSG00000055148/ ENSMUSG00000070056/ ENSMUSG00000031543/ ENSMUSG00000024789/ ENSMUSG00000028382/ ENSMUSG00000021127	13
GO:0050671	positive regulation of lymphocyte proliferation	13/815	137/21092	0.0025	0.0323	0.0280	ENSMUSG00000115338/ ENSMUSG00000027950/ ENSMUSG00000067212/ ENSMUSG00000000791/ ENSMUSG00000032508/ ENSMUSG00000039217/ ENSMUSG00000066877/ ENSMUSG00000040329/ ENSMUSG00000016496/ ENSMUSG00000035042/ ENSMUSG00000035914/ ENSMUSG00000031712/ ENSMUSG00000025746	13
GO:0060249	anatomical structure homeostasis	27/815	389/21092	0.0026	0.0325	0.0282	ENSMUSG00000036986/ ENSMUSG00000034218/ ENSMUSG00000037855/ ENSMUSG00000056220/ ENSMUSG00000019699/ ENSMUSG00000021795/ ENSMUSG00000002204/ ENSMUSG00000027562/ ENSMUSG00000021360/ ENSMUSG00000027323/ ENSMUSG00000020128/ ENSMUSG00000023249/ ENSMUSG00000022346/ ENSMUSG00000001642/ ENSMUSG00000042772/ ENSMUSG00000032410/ ENSMUSG00000016534/ ENSMUSG00000110206/	27

							ENSMUSG00000038467/ ENSMUSG00000001416/ ENSMUSG00000040329/ ENSMUSG00000024472/ ENSMUSG00000015133/ ENSMUSG00000038301/ ENSMUSG00000024330/ ENSMUSG00000014599/ ENSMUSG00000025746	
GO:0046638	positive regulation of alpha−beta T cell differentiation	7/815	49/21092	0.0026	0.0331	0.0287	ENSMUSG00000115338/ ENSMUSG00000038037/ ENSMUSG00000039217/ ENSMUSG00000031304/ ENSMUSG00000038855/ ENSMUSG00000018899/ ENSMUSG00000025746	7
GO:0030316	osteoclast differentiation	11/815	106/21092	0.0026	0.0333	0.0289	ENSMUSG00000048806/ ENSMUSG00000027204/ ENSMUSG00000030157/ ENSMUSG00000027562/ ENSMUSG00000004508/ ENSMUSG00000035042/ ENSMUSG00000015133/ ENSMUSG00000038301/ ENSMUSG00000014599/ ENSMUSG00000021250/ ENSMUSG00000046186	11
GO:2000379	positive regulation of reactive oxygen species metabolic process	11/815	106/21092	0.0026	0.0333	0.0289	ENSMUSG00000024066/ ENSMUSG00000016206/ ENSMUSG00000003032/ ENSMUSG00000039217/ ENSMUSG00000040152/ ENSMUSG00000041736/ ENSMUSG00000055148/ ENSMUSG00000022126/ ENSMUSG00000027995/ ENSMUSG00000024789/ ENSMUSG00000025746	11
GO:0070304	positive regulation of stress−activated protein kinase signaling cascade	15/815	171/21092	0.0027	0.0336	0.0292	ENSMUSG00000012519/ ENSMUSG00000024079/ ENSMUSG00000024066/ ENSMUSG00000031639/ ENSMUSG00000020357/ ENSMUSG00000061288/ ENSMUSG00000032508/ ENSMUSG00000031948/ ENSMUSG00000038058/ ENSMUSG00000021453/ ENSMUSG00000070056/ ENSMUSG00000021754/ ENSMUSG00000026872/ ENSMUSG00000026981/ ENSMUSG00000069255	15
GO:0002369	T cell cytokine production	6/815	37/21092	0.0027	0.0338	0.0293	ENSMUSG00000060802/ ENSMUSG00000048806/ ENSMUSG00000037447/ ENSMUSG00000039217/ ENSMUSG00000020641/ ENSMUSG00000025746	6
GO:0032620	interleukin−17 production	6/815	37/21092	0.0027	0.0338	0.0293	ENSMUSG00000037447/ ENSMUSG00000032508/ ENSMUSG00000039217/ ENSMUSG00000031154/ ENSMUSG00000027995/ ENSMUSG00000031712	6
GO:0033028	myeloid cell apoptotic process	6/815	37/21092	0.0027	0.0338	0.0293	ENSMUSG00000039217/ ENSMUSG00000035042/ ENSMUSG00000027737/ ENSMUSG00000045502/ ENSMUSG00000038855/ ENSMUSG00000025746	6
GO:0042092	type 2 immune response	6/815	37/21092	0.0027	0.0338	0.0293	ENSMUSG00000048806/ ENSMUSG00000039217/ ENSMUSG00000020641/ ENSMUSG00000024810/ ENSMUSG00000018899/ ENSMUSG00000025746	6

GO:0046640	regulation of alpha−beta T cell proliferation	6/815	37/21092	0.0027	0.0338	0.0293	ENSMUSG00000001123/ ENSMUSG00000067212/ ENSMUSG00000039217/ ENSMUSG00000042333/ ENSMUSG00000016496/ ENSMUSG00000018899	6
GO:0002830	positive regulation of type 2 immune response	4/815	16/21092	0.0028	0.0341	0.0296	ENSMUSG00000039217/ ENSMUSG00000020641/ ENSMUSG00000024810/ ENSMUSG00000025746	4
GO:0032230	positive regulation of synaptic transmission. GABAergic	4/815	16/21092	0.0028	0.0341	0.0296	ENSMUSG00000045038/ ENSMUSG00000027562/ ENSMUSG00000051790/ ENSMUSG00000006930	4
GO:0050755	chemokine metabolic process	4/815	16/21092	0.0028	0.0341	0.0296	ENSMUSG00000031639/ ENSMUSG00000032508/ ENSMUSG00000038418/ ENSMUSG00000025746	4
GO:0031334	positive regulation of protein complex assembly	19/815	242/21092	0.0028	0.0346	0.0300	ENSMUSG00000034218/ ENSMUSG00000045038/ ENSMUSG00000057058/ ENSMUSG00000002233/ ENSMUSG00000000127/ ENSMUSG00000037860/ ENSMUSG00000000915/ ENSMUSG00000031207/ ENSMUSG00000021676/ ENSMUSG00000028217/ ENSMUSG00000021266/ ENSMUSG00000045795/ ENSMUSG00000066877/ ENSMUSG00000058624/ ENSMUSG00000052684/ ENSMUSG00000037062/ ENSMUSG00000034724/ ENSMUSG00000021754/ ENSMUSG00000105504	19
GO:0002761	regulation of myeloid leukocyte differentiation	12/815	123/21092	0.0029	0.0356	0.0309	ENSMUSG00000048806/ ENSMUSG00000027204/ ENSMUSG00000030157/ ENSMUSG00000027562/ ENSMUSG00000022346/ ENSMUSG00000052684/ ENSMUSG00000032501/ ENSMUSG00000035042/ ENSMUSG00000031750/ ENSMUSG00000014599/ ENSMUSG00000021250/ ENSMUSG00000021127	12
GO:0072593	reactive oxygen species metabolic process	21/815	280/21092	0.0030	0.0368	0.0320	ENSMUSG00000024066/ ENSMUSG00000016206/ ENSMUSG00000037580/ ENSMUSG00000003032/ ENSMUSG00000039217/ ENSMUSG00000029759/ ENSMUSG00000055447/ ENSMUSG00000040152/ ENSMUSG00000041736/ ENSMUSG00000004040/ ENSMUSG00000031266/ ENSMUSG00000032066/ ENSMUSG00000055148/ ENSMUSG00000030921/ ENSMUSG00000015340/ ENSMUSG00000022126/ ENSMUSG00000004231/ ENSMUSG00000027995/ ENSMUSG00000024789/ ENSMUSG00000020826/ ENSMUSG00000025746	21

GO:0034612	response to tumor necrosis factor	15/815	174/21092	0.0032	0.0384	0.0334	ENSMUSG00000026104/ ENSMUSG00000037580/ ENSMUSG00000037860/ ENSMUSG00000031948/ ENSMUSG00000035042/ ENSMUSG00000025888/ ENSMUSG00000035373/ ENSMUSG00000022126/ ENSMUSG00000035186/ ENSMUSG00000018899/ ENSMUSG00000033538/ ENSMUSG00000024789/ ENSMUSG00000035385/ ENSMUSG00000021127/ ENSMUSG00000025746	15
GO:0051222	positive regulation of protein transport	29/815	435/21092	0.0033	0.0395	0.0343	ENSMUSG00000026896/ ENSMUSG00000040296/ ENSMUSG00000001123/ ENSMUSG00000067212/ ENSMUSG00000036908/ ENSMUSG00000072244/ ENSMUSG00000045038/ ENSMUSG00000037447/ ENSMUSG00000037860/ ENSMUSG00000022575/ ENSMUSG00000022514/ ENSMUSG00000042333/ ENSMUSG00000031948/ ENSMUSG00000051790/ ENSMUSG00000016496/ ENSMUSG00000025888/ ENSMUSG00000026433/ ENSMUSG00000035914/ ENSMUSG00000034584/ ENSMUSG00000002897/ ENSMUSG00000024810/ ENSMUSG00000045502/ ENSMUSG00000034485/ ENSMUSG00000033538/ ENSMUSG00000027995/ ENSMUSG00000024789/ ENSMUSG00000035385/ ENSMUSG00000025746/ ENSMUSG00000032328	29
GO:0060761	negative regulation of response to cytokine stimulus	7/815	51/21092	0.0033	0.0399	0.0347	ENSMUSG00000034422/ ENSMUSG00000027639/ ENSMUSG00000027951/ ENSMUSG00000003032/ ENSMUSG00000074151/ ENSMUSG00000026981/ ENSMUSG00000025746	7
GO:0036037	CD8−positive. alpha−beta T cell activation	5/815	27/21092	0.0034	0.0408	0.0355	ENSMUSG00000067212/ ENSMUSG00000038037/ ENSMUSG00000031154/ ENSMUSG00000016496/ ENSMUSG00000018899	5
GO:0016236	macroautophagy	16/815	193/21092	0.0035	0.0416	0.0362	ENSMUSG00000069874/ ENSMUSG00000057554/ ENSMUSG00000026672/ ENSMUSG00000056692/ ENSMUSG00000021036/ ENSMUSG00000016534/ ENSMUSG00000046879/ ENSMUSG00000038467/ ENSMUSG00000037331/ ENSMUSG00000020115/ ENSMUSG00000025241/ ENSMUSG00000038058/ ENSMUSG00000001750/ ENSMUSG00000037062/ ENSMUSG00000028211/ ENSMUSG00000027995	16

GO:0060416	response to growth hormone	4/815	17/21092	0.0035	0.0421	0.0365	ENSMUSG00000004040/ ENSMUSG00000018899/ ENSMUSG00000038508/ ENSMUSG00000024789	4
GO:1904469	positive regulation of tumor necrosis factor secretion	4/815	17/21092	0.0035	0.0421	0.0365	ENSMUSG00000026896/ ENSMUSG00000040296/ ENSMUSG00000037447/ ENSMUSG00000027995	4
GO:2001185	regulation of CD8−positive. alpha−beta T cell activation	4/815	17/21092	0.0035	0.0421	0.0365	ENSMUSG00000067212/ ENSMUSG00000038037/ ENSMUSG00000016496/ ENSMUSG00000018899	4
GO:0046632	alpha−beta T cell differentiation	11/815	110/21092	0.0035	0.0421	0.0366	ENSMUSG00000115338/ ENSMUSG00000038037/ ENSMUSG00000039217/ ENSMUSG00000031154/ ENSMUSG00000004040/ ENSMUSG00000031304/ ENSMUSG00000021453/ ENSMUSG00000020641/ ENSMUSG00000038855/ ENSMUSG00000018899/ ENSMUSG00000025746	11
GO:0030218	erythrocyte differentiation	12/815	126/21092	0.0035	0.0422	0.0366	ENSMUSG00000026104/ ENSMUSG00000027951/ ENSMUSG00000060802/ ENSMUSG00000032698/ ENSMUSG00000004040/ ENSMUSG00000035692/ ENSMUSG00000055148/ ENSMUSG00000070056/ ENSMUSG00000031543/ ENSMUSG00000024789/ ENSMUSG00000028382/ ENSMUSG00000021127	12
GO:0050856	regulation of T cell receptor signaling pathway	6/815	39/21092	0.0036	0.0425	0.0369	ENSMUSG00000036461/ ENSMUSG00000029640/ ENSMUSG00000041187/ ENSMUSG00000026433/ ENSMUSG00000069255/ ENSMUSG00000062300	6
GO:0043367	CD4−positive. alpha−beta T cell differentiation	9/815	80/21092	0.0037	0.0434	0.0377	ENSMUSG00000038037/ ENSMUSG00000039217/ ENSMUSG00000031154/ ENSMUSG00000004040/ ENSMUSG00000031304/ ENSMUSG00000021453/ ENSMUSG00000020641/ ENSMUSG00000018899/ ENSMUSG00000025746	9
GO:0032655	regulation of interleukin−12 production	7/815	52/21092	0.0037	0.0435	0.0378	ENSMUSG00000036908/ ENSMUSG00000016206/ ENSMUSG00000031639/ ENSMUSG00000055447/ ENSMUSG00000040152/ ENSMUSG00000018899/ ENSMUSG00000027995	7
GO:0002695	negative regulation of leukocyte activation	15/815	177/21092	0.0037	0.0437	0.0380	ENSMUSG00000048806/ ENSMUSG00000001123/ ENSMUSG00000016206/ ENSMUSG00000034218/ ENSMUSG00000002602/ ENSMUSG00000021795/ ENSMUSG00000038037/ ENSMUSG00000000127/ ENSMUSG00000042333/ ENSMUSG00000090958/ ENSMUSG00000016496/ ENSMUSG00000035914/ ENSMUSG00000056153/ ENSMUSG00000018899/ ENSMUSG00000069255	15

GO:0046425	regulation of JAK−STAT cascade	14/815	161/21092	0.0039	0.0464	0.0403	ENSMUSG00000034422/ ENSMUSG00000022906/ ENSMUSG00000048806/ ENSMUSG00000070904/ ENSMUSG00000064128/ ENSMUSG00000038037/ ENSMUSG00000039217/ ENSMUSG00000001642/ ENSMUSG00000035042/ ENSMUSG00000018899/ ENSMUSG00000031712/ ENSMUSG00000030895/ ENSMUSG00000024789/ ENSMUSG00000025746	14
GO:1903426	regulation of reactive oxygen species biosynthetic process	10/815	96/21092	0.0040	0.0464	0.0403	ENSMUSG00000016206/ ENSMUSG00000003032/ ENSMUSG00000055447/ ENSMUSG00000041736/ ENSMUSG00000004040/ ENSMUSG00000031266/ ENSMUSG00000055148/ ENSMUSG00000027995/ ENSMUSG00000024789/ ENSMUSG00000025746	10
GO:0032816	positive regulation of natural killer cell activation	5/815	28/21092	0.0040	0.0466	0.0405	ENSMUSG00000002602/ ENSMUSG00000039217/ ENSMUSG00000110206/ ENSMUSG00000018899/ ENSMUSG00000031712	5
GO:0035690	cellular response to drug	25/815	363/21092	0.0040	0.0466	0.0405	ENSMUSG00000002307/ ENSMUSG00000048806/ ENSMUSG00000027950/ ENSMUSG00000002602/ ENSMUSG00000045038/ ENSMUSG00000022587/ ENSMUSG00000027323/ ENSMUSG00000037860/ ENSMUSG00000022346/ ENSMUSG00000001642/ ENSMUSG00000019907/ ENSMUSG00000040152/ ENSMUSG00000037331/ ENSMUSG00000028367/ ENSMUSG00000038058/ ENSMUSG00000000838/ ENSMUSG00000028211/ ENSMUSG00000032177/ ENSMUSG00000035373/ ENSMUSG00000022126/ ENSMUSG00000004231/ ENSMUSG00000038418/ ENSMUSG00000024789/ ENSMUSG00000020826/ ENSMUSG00000025746	25
GO:0050732	negative regulation of peptidyl−tyrosine phosphorylation	7/815	53/21092	0.0041	0.0478	0.0415	ENSMUSG00000034422/ ENSMUSG00000038037/ ENSMUSG00000039217/ ENSMUSG00000015133/ ENSMUSG00000030681/ ENSMUSG00000018899/ ENSMUSG00000069255	7
GO:0050866	negative regulation of cell activation	16/815	197/21092	0.0042	0.0490	0.0426	ENSMUSG00000048806/ ENSMUSG00000001123/ ENSMUSG00000016206/ ENSMUSG00000034218/ ENSMUSG00000002602/ ENSMUSG00000021795/ ENSMUSG00000038037/ ENSMUSG00000000127/ ENSMUSG00000017446/ ENSMUSG00000042333/ ENSMUSG00000090958/ ENSMUSG00000016496/	16

							ENSMUSG00000035914/ ENSMUSG00000056153/ ENSMUSG00000018899/ ENSMUSG00000069255	
GO:0008630	intrinsic apoptotic signaling pathway in response to DNA damage	11/815	113/21092	0.0043	0.0498	0.0433	ENSMUSG00000025647/ ENSMUSG00000115338/ ENSMUSG00000036986/ ENSMUSG00000034218/ ENSMUSG00000022346/ ENSMUSG00000073489/ ENSMUSG00000034485/ ENSMUSG00000030717/ ENSMUSG00000022676/ ENSMUSG00000028599/ ENSMUSG00000027660	11
GO:0002577	regulation of antigen processing and presentation	4/815	18/21092	0.0044	0.0498	0.0433	ENSMUSG00000024339/ ENSMUSG00000038213/ ENSMUSG00000040152/ ENSMUSG00000038058	4
GO:0016137	glycoside metabolic process	4/815	18/21092	0.0044	0.0498	0.0433	ENSMUSG00000001642/ ENSMUSG00000031266/ ENSMUSG00000033715/ ENSMUSG00000054757	4
GO:0032656	regulation of interleukin−13 production	4/815	18/21092	0.0044	0.0498	0.0433	ENSMUSG00000067212/ ENSMUSG00000022514/ ENSMUSG00000002897/ ENSMUSG00000024810	4
GO:0032674	regulation of interleukin−5 production	4/815	18/21092	0.0044	0.0498	0.0433	ENSMUSG00000022514/ ENSMUSG00000002897/ ENSMUSG00000024810/ ENSMUSG00000005364	4
GO:0034138	toll−like receptor 3 signaling pathway	4/815	18/21092	0.0044	0.0498	0.0433	ENSMUSG00000001123/ ENSMUSG00000036908/ ENSMUSG00000031639/ ENSMUSG00000073643	4
GO:0039531	regulation of viral−induced cytoplasmic pattern recognition receptor signaling pathway	4/815	18/21092	0.0044	0.0498	0.0433	ENSMUSG00000017830/ ENSMUSG00000029826/ ENSMUSG00000037921/ ENSMUSG00000055204	4
GO:0043555	regulation of translation in response to stress	4/815	18/21092	0.0044	0.0498	0.0433	ENSMUSG00000024079/ ENSMUSG00000036986/ ENSMUSG00000066877/ ENSMUSG00000040435	4
GO:2000641	regulation of early endosome to late endosome transport	4/815	18/21092	0.0044	0.0498	0.0433	ENSMUSG00000031207/ ENSMUSG00000004936/ ENSMUSG00000037062/ ENSMUSG00000020132	4
**DENV−infected and APAP−treated cells**								
**GO terms significantly enriched in gene cluster 2**								
**ID**	**Description**	**GeneRatio**	**BgRatio**	**pvalue**	**p.adjust**	**qvalue**	**geneID**	**Count**

GO:0070059	intrinsic apoptotic signaling pathway in response to endoplasmic reticulum stress	3/18	63/21087	2.01×10^{-05}	0.007	0.004	ENSMUSG00000002083/ ENSMUSG00000025408/ ENSMUSG00000059552	3
GO:2001244	positive regulation of intrinsic apoptotic signaling pathway	3/18	70/21087	2.76×10^{-05}	0.007	0.004	ENSMUSG00000002083/ ENSMUSG00000025408/ ENSMUSG00000059552	3
GO:1990440	positive regulation of transcription from RNA polymerase II promoter in response to endoplasmic reticulum stress	2/18	11/21087	3.77×10^{-05}	0.007	0.004	ENSMUSG00000025408/ ENSMUSG00000059552	2
GO:0043525	positive regulation of neuron apoptotic process	3/18	80/21087	4.12×10^{-05}	0.007	0.004	ENSMUSG00000002083/ ENSMUSG00000025408/ ENSMUSG00000059552	3
GO:0006983	ER overload response	2/18	13/21087	5.34×10^{-05}	0.007	0.004	ENSMUSG00000025408/ ENSMUSG00000059552	2
GO:0072655	establishment of protein localization to mitochondrion	3/18	92/21087	6.25×10^{-05}	0.007	0.004	ENSMUSG00000002083/ ENSMUSG00000025408/ ENSMUSG00000041736	3
GO:0070234	positive regulation of T cell apoptotic process	2/18	15/21087	7.18×10^{-05}	0.007	0.004	ENSMUSG00000002083/ ENSMUSG00000059552	2
GO:0070585	protein localization to mitochondrion	3/18	98/21087	7.55×10^{-05}	0.007	0.004	ENSMUSG00000002083/ ENSMUSG00000025408/ ENSMUSG00000041736	3
GO:0070243	regulation of thymocyte apoptotic process	2/18	17/21087	9.29×10^{-05}	0.008	0.004	ENSMUSG00000002083/ ENSMUSG00000059552	2
GO:0051235	maintenance of location	4/18	313/21087	0.0001	0.009	0.005	ENSMUSG00000002083/ ENSMUSG00000025408/ ENSMUSG00000025509/ ENSMUSG00000041736	4
GO:1901216	positive regulation of neuron death	3/18	121/21087	0.0001	0.009	0.005	ENSMUSG00000002083/ ENSMUSG00000025408/ ENSMUSG00000059552	3
GO:0070230	positive regulation of lymphocyte apoptotic process	2/18	21/21087	0.0001	0.009	0.005	ENSMUSG00000002083/ ENSMUSG00000059552	2
GO:0070242	thymocyte apoptotic process	2/18	22/21087	0.0002	0.009	0.005	ENSMUSG00000002083/ ENSMUSG00000059552	2
GO:0032461	positive regulation of protein oligomerization	2/18	24/21087	0.0002	0.009	0.005	ENSMUSG00000002083/ ENSMUSG00000059552	2
GO:0010821	regulation of mitochondrion organization	3/18	138/21087	0.0002	0.009	0.005	ENSMUSG00000002083/ ENSMUSG00000059552/ ENSMUSG00000041736	3
GO:0009299	mRNA transcription	2/18	26/21087	0.0002	0.009	0.005	ENSMUSG00000025408/ ENSMUSG00000059552	2

GO:0036003	positive regulation of transcription from RNA polymerase II promoter in response to stress	2/18	27/21087	0.0002	0.009	0.005	ENSMUSG00000025408/ ENSMUSG00000059552	2
GO:0008340	determination of adult lifespan	2/18	28/21087	0.0003	0.009	0.005	ENSMUSG00000002083/ ENSMUSG00000059552	2
GO:0090200	positive regulation of release of cytochrome c from mitochondria	2/18	28/21087	0.0003	0.009	0.005	ENSMUSG00000002083/ ENSMUSG00000059552	2
GO:1903579	negative regulation of ATP metabolic process	2/18	28/21087	0.0003	0.009	0.005	ENSMUSG00000059552/ ENSMUSG00000041736	2
GO:0060969	negative regulation of gene silencing	2/18	31/21087	0.0003	0.011	0.006	ENSMUSG00000052565/ ENSMUSG00000059552	2
GO:0072594	establishment of protein localization to organelle	4/18	413/21087	0.0004	0.011	0.006	ENSMUSG00000002083/ ENSMUSG00000025408/ ENSMUSG00000059552/ ENSMUSG00000041736	4
GO:1903146	regulation of autophagy of mitochondrion	2/18	33/21087	0.0004	0.011	0.006	ENSMUSG00000059552/ ENSMUSG00000041736	2
GO:2000108	positive regulation of leukocyte apoptotic process	2/18	33/21087	0.0004	0.011	0.006	ENSMUSG00000002083/ ENSMUSG00000059552	2
GO:0006984	ER−nucleus signaling pathway	2/18	34/21087	0.0004	0.011	0.006	ENSMUSG00000025408/ ENSMUSG00000059552	2
GO:2001242	regulation of intrinsic apoptotic signaling pathway	3/18	173/21087	0.0004	0.011	0.006	ENSMUSG00000002083/ ENSMUSG00000025408/ ENSMUSG00000059552	3
GO:0035794	positive regulation of mitochondrial membrane permeability	2/18	37/21087	0.0005	0.012	0.006	ENSMUSG00000002083/ ENSMUSG00000059552	2
GO:1901998	toxin transport	2/18	37/21087	0.0005	0.012	0.006	ENSMUSG00000002083/ ENSMUSG00000027936	2
GO:2001235	positive regulation of apoptotic signaling pathway	3/18	186/21087	0.0005	0.012	0.006	ENSMUSG00000002083/ ENSMUSG00000025408/ ENSMUSG00000059552	3
GO:1905710	positive regulation of membrane permeability	2/18	39/21087	0.0005	0.012	0.006	ENSMUSG00000002083/ ENSMUSG00000059552	2
GO:1902108	regulation of mitochondrial membrane permeability involved in apoptotic process	2/18	40/21087	0.0005	0.013	0.007	ENSMUSG00000002083/ ENSMUSG00000059552	2
GO:0032459	regulation of protein oligomerization	2/18	42/21087	0.0006	0.013	0.007	ENSMUSG00000002083/ ENSMUSG00000059552	2

GO:0042771	intrinsic apoptotic signaling pathway in response to DNA damage by p53 class mediator	2/18	43/21087	0.0006	0.013	0.007	ENSMUSG00000002083/ ENSMUSG00000059552	2
GO:0070232	regulation of T cell apoptotic process	2/18	43/21087	0.0006	0.013	0.007	ENSMUSG00000002083/ ENSMUSG00000059552	2
GO:0010259	multicellular organism aging	2/18	47/21087	0.0007	0.015	0.008	ENSMUSG00000002083/ ENSMUSG00000059552	2
GO:0051651	maintenance of location in cell	3/18	216/21087	0.0008	0.016	0.008	ENSMUSG00000002083/ ENSMUSG00000025408/ ENSMUSG00000041736	3
GO:0090199	regulation of release of cytochrome c from mitochondria	2/18	49/21087	0.0008	0.016	0.008	ENSMUSG00000002083/ ENSMUSG00000059552	2
GO:0043618	regulation of transcription from RNA polymerase II promoter in response to stress	2/18	51/21087	0.0009	0.016	0.009	ENSMUSG00000025408/ ENSMUSG00000059552	2
GO:0070265	necrotic cell death	2/18	51/21087	0.0009	0.016	0.009	ENSMUSG00000059552/ ENSMUSG00000041736	2
GO:0010823	negative regulation of mitochondrion organization	2/18	55/21087	0.0010	0.018	0.009	ENSMUSG00000059552/ ENSMUSG00000041736	2
GO:0046902	regulation of mitochondrial membrane permeability	2/18	55/21087	0.0010	0.018	0.009	ENSMUSG00000002083/ ENSMUSG00000059552	2
GO:0043620	regulation of DNA−templated transcription in response to stress	2/18	56/21087	0.0010	0.018	0.009	ENSMUSG00000025408/ ENSMUSG00000059552	2
GO:0034976	response to endoplasmic reticulum stress	3/18	240/21087	0.0010	0.018	0.009	ENSMUSG00000002083/ ENSMUSG00000025408/ ENSMUSG00000059552	3
GO:0070231	T cell apoptotic process	2/18	58/21087	0.0011	0.018	0.010	ENSMUSG00000002083/ ENSMUSG00000059552	2
GO:0010506	regulation of autophagy	3/18	253/21087	0.0012	0.020	0.011	ENSMUSG00000025408/ ENSMUSG00000059552/ ENSMUSG00000041736	3
GO:0001836	release of cytochrome c from mitochondria	2/18	62/21087	0.0013	0.020	0.011	ENSMUSG00000002083/ ENSMUSG00000059552	2
GO:0071479	cellular response to ionizing radiation	2/18	65/21087	0.0014	0.020	0.011	ENSMUSG00000002083/ ENSMUSG00000059552	2
GO:0043523	regulation of neuron apoptotic process	3/18	266/21087	0.0014	0.020	0.011	ENSMUSG00000002083/ ENSMUSG00000025408/ ENSMUSG00000059552	3

GO:0000422	autophagy of mitochondrion	2/18	66/21087	0.0014	0.020	0.011	ENSMUSG00000059552/ ENSMUSG00000041736	2
GO:0061726	mitochondrion disassembly	2/18	66/21087	0.0014	0.020	0.011	ENSMUSG00000059552/ ENSMUSG00000041736	2
GO:0090559	regulation of membrane permeability	2/18	67/21087	0.0015	0.020	0.011	ENSMUSG00000002083/ ENSMUSG00000059552	2
GO:0097194	execution phase of apoptosis	2/18	67/21087	0.0015	0.020	0.011	ENSMUSG00000002083/ ENSMUSG00000059552	2
GO:2000378	negative regulation of reactive oxygen species metabolic process	2/18	67/21087	0.0015	0.020	0.011	ENSMUSG00000059552/ ENSMUSG00000041736	2
GO:0010507	negative regulation of autophagy	2/18	75/21087	0.0018	0.024	0.013	ENSMUSG00000059552/ ENSMUSG00000041736	2
GO:0010822	positive regulation of mitochondrion organization	2/18	75/21087	0.0018	0.024	0.013	ENSMUSG00000002083/ ENSMUSG00000059552	2
GO:0070228	regulation of lymphocyte apoptotic process	2/18	76/21087	0.0019	0.024	0.013	ENSMUSG00000002083/ ENSMUSG00000059552	2
GO:0097193	intrinsic apoptotic signaling pathway	3/18	295/21087	0.0019	0.024	0.013	ENSMUSG00000002083/ ENSMUSG00000025408/ ENSMUSG00000059552	3
GO:0072332	intrinsic apoptotic signaling pathway by p53 class mediator	2/18	77/21087	0.0019	0.024	0.013	ENSMUSG00000002083/ ENSMUSG00000059552	2
GO:0051402	neuron apoptotic process	3/18	298/21087	0.0019	0.024	0.013	ENSMUSG00000002083/ ENSMUSG00000025408/ ENSMUSG00000059552	3
GO:0060968	regulation of gene silencing	2/18	80/21087	0.0021	0.026	0.014	ENSMUSG00000052565/ ENSMUSG00000059552	2
GO:1903008	organelle disassembly	2/18	90/21087	0.0026	0.032	0.017	ENSMUSG00000059552/ ENSMUSG00000041736	2
GO:1903578	regulation of ATP metabolic process	2/18	92/21087	0.0028	0.033	0.017	ENSMUSG00000059552/ ENSMUSG00000041736	2
GO:0070227	lymphocyte apoptotic process	2/18	98/21087	0.0031	0.036	0.019	ENSMUSG00000002083/ ENSMUSG00000059552	2
GO:0007006	mitochondrial membrane organization	2/18	100/21087	0.0032	0.037	0.020	ENSMUSG00000002083/ ENSMUSG00000059552	2
GO:0051289	protein homotetramerization	2/18	104/21087	0.0035	0.039	0.021	ENSMUSG00000027936/ ENSMUSG00000059552	2
GO:1905477	positive regulation of protein localization to membrane	2/18	104/21087	0.0035	0.039	0.021	ENSMUSG00000002083/ ENSMUSG00000073982	2
GO:1901214	regulation of neuron death	3/18	370/21087	0.0036	0.039	0.021	ENSMUSG00000002083/ ENSMUSG00000025408/ ENSMUSG00000059552	3

GO:2000106	regulation of leukocyte apoptotic process	2/18	108/21087	0.0038	0.041	0.022	ENSMUSG00000002083/ ENSMUSG00000059552	2
GO:2000379	positive regulation of reactive oxygen species metabolic process	2/18	110/21087	0.0039	0.042	0.022	ENSMUSG00000059552/ ENSMUSG00000041736	2
GO:0051260	protein homooligomerization	3/18	385/21087	0.0040	0.042	0.022	ENSMUSG00000002083/ ENSMUSG00000027936/ ENSMUSG00000059552	3
GO:0008630	intrinsic apoptotic signaling pathway in response to DNA damage	2/18	114/21087	0.0042	0.042	0.022	ENSMUSG00000002083/ ENSMUSG00000059552	2
GO:0008637	apoptotic mitochondrial changes	2/18	114/21087	0.0042	0.042	0.022	ENSMUSG00000002083/ ENSMUSG00000059552	2
GO:0051209	release of sequestered calcium ion into cytosol	2/18	115/21087	0.0043	0.042	0.022	ENSMUSG00000002083/ ENSMUSG00000025408	2
GO:0006914	autophagy	3/18	395/21087	0.0043	0.042	0.022	ENSMUSG00000025408/ ENSMUSG00000059552/ ENSMUSG00000041736	3
GO:0061919	process utilizing autophagic mechanism	3/18	395/21087	0.0043	0.042	0.022	ENSMUSG00000025408/ ENSMUSG00000059552/ ENSMUSG00000041736	3
GO:0051283	negative regulation of sequestering of calcium ion	2/18	117/21087	0.0044	0.043	0.023	ENSMUSG00000002083/ ENSMUSG00000025408	2
GO:0051282	regulation of sequestering of calcium ion	2/18	119/21087	0.0046	0.043	0.023	ENSMUSG00000002083/ ENSMUSG00000025408	2
GO:0010639	negative regulation of organelle organization	3/18	404/21087	0.0046	0.043	0.023	ENSMUSG00000052565/ ENSMUSG00000059552/ ENSMUSG00000041736	3
GO:0051208	sequestering of calcium ion	2/18	120/21087	0.0046	0.043	0.023	ENSMUSG00000002083/ ENSMUSG00000025408	2
GO:0070997	neuron death	3/18	411/21087	0.0048	0.044	0.023	ENSMUSG00000002083/ ENSMUSG00000025408/ ENSMUSG00000059552	3
GO:0051053	negative regulation of DNA metabolic process	2/18	123/21087	0.0049	0.044	0.023	ENSMUSG00000052565/ ENSMUSG00000059552	2
GO:2001233	regulation of apoptotic signaling pathway	3/18	420/21087	0.0051	0.046	0.024	ENSMUSG00000002083/ ENSMUSG00000025408/ ENSMUSG00000059552	3
GO:0010212	response to ionizing radiation	2/18	127/21087	0.0052	0.046	0.024	ENSMUSG00000002083/ ENSMUSG00000059552	2

GO:0006816	calcium ion transport	3/18	427/21087	0.0054	0.046	0.025	ENSMUSG00000002083/ ENSMUSG00000025408/ ENSMUSG00000041736	3
GO:0016570	histone modification	3/18	427/21087	0.0054	0.046	0.025	ENSMUSG00000027936/ ENSMUSG00000052565/ ENSMUSG00000059552	3
GO:0071887	leukocyte apoptotic process	2/18	132/21087	0.0056	0.048	0.025	ENSMUSG00000002083/ ENSMUSG00000059552	2
GO:0097553	calcium ion transmembrane import into cytosol	2/18	136/21087	0.0059	0.050	0.026	ENSMUSG00000002083/ ENSMUSG00000025408	2
GO:0016569	covalent chromatin modification	3/18	444/21087	0.0060	0.050	0.026	ENSMUSG00000027936/ ENSMUSG00000052565/ ENSMUSG00000059552	3
GO:0072331	signal transduction by p53 class mediator	2/18	137/21087	0.0060	0.050	0.026	ENSMUSG00000002083/ ENSMUSG00000059552	2
**GO terms significantly enriched in gene cluster 4**								
**ID**	**Description**	**GeneRatio**	**BgRatio**	**pvalue**	**p.adjust**	**qvalue**	**geneID**	**Count**
GO:0006977	DNA damage response. signal transduction by p53 class mediator resulting in cell cycle arrest	2/17	11/21087	3.35×10^{-05}	0.002	0.002	ENSMUSG00000027510/ ENSMUSG00000060938	2
GO:0002181	cytoplasmic translation	3/17	82/21087	3.71×10^{-05}	0.002	0.002	ENSMUSG00000031490/ ENSMUSG00000060938/ ENSMUSG00000057863	3
GO:0072431	signal transduction involved in mitotic G1 DNA damage checkpoint	2/17	12/21087	4.02×10^{-05}	0.002	0.002	ENSMUSG00000027510/ ENSMUSG00000060938	2
GO:1902400	intracellular signal transduction involved in G1 DNA damage checkpoint	2/17	12/21087	4.02×10^{-05}	0.002	0.002	ENSMUSG00000027510/ ENSMUSG00000060938	2
GO:0072413	signal transduction involved in mitotic cell cycle checkpoint	2/17	15/21087	6.38×10^{-05}	0.002	0.002	ENSMUSG00000027510/ ENSMUSG00000060938	2
GO:1902402	signal transduction involved in mitotic DNA damage checkpoint	2/17	15/21087	6.38×10^{-05}	0.002	0.002	ENSMUSG00000027510/ ENSMUSG00000060938	2
GO:1902403	signal transduction involved in mitotic DNA integrity checkpoint	2/17	15/21087	6.38×10^{-05}	0.002	0.002	ENSMUSG00000027510/ ENSMUSG00000060938	2

GO:0006978	DNA damage response. signal transduction by p53 class mediator resulting in transcription of p21 class mediator	2/17	16/21087	7.29×10^{-05}	0.002	0.002	ENSMUSG00000027510/ ENSMUSG00000060938	2
GO:0042772	DNA damage response. signal transduction resulting in transcription	2/17	17/21087	8.26×10^{-05}	0.002	0.002	ENSMUSG00000027510/ ENSMUSG00000060938	2
GO:0031571	mitotic G1 DNA damage checkpoint	2/17	19/21087	0.0001	0.003	0.002	ENSMUSG00000027510/ ENSMUSG00000060938	2
GO:0044783	G1 DNA damage checkpoint	2/17	20/21087	0.0001	0.003	0.002	ENSMUSG00000027510/ ENSMUSG00000060938	2
GO:0044819	mitotic G1/S transition checkpoint	2/17	20/21087	0.0001	0.003	0.002	ENSMUSG00000027510/ ENSMUSG00000060938	2
GO:0044772	mitotic cell cycle phase transition	4/17	363/21087	0.0002	0.004	0.002	ENSMUSG00000031490/ ENSMUSG00000025226/ ENSMUSG00000027510/ ENSMUSG00000060938	4
GO:0072395	signal transduction involved in cell cycle checkpoint	2/17	29/21087	0.0002	0.004	0.003	ENSMUSG00000027510/ ENSMUSG00000060938	2
GO:0072401	signal transduction involved in DNA integrity checkpoint	2/17	29/21087	0.0002	0.004	0.003	ENSMUSG00000027510/ ENSMUSG00000060938	2
GO:0072422	signal transduction involved in DNA damage checkpoint	2/17	29/21087	0.0002	0.004	0.003	ENSMUSG00000027510/ ENSMUSG00000060938	2
GO:0044770	cell cycle phase transition	4/17	402/21087	0.0003	0.004	0.003	ENSMUSG00000031490/ ENSMUSG00000025226/ ENSMUSG00000027510/ ENSMUSG00000060938	4
GO:0000082	G1/S transition of mitotic cell cycle	3/17	179/21087	0.0004	0.006	0.004	ENSMUSG00000031490/ ENSMUSG00000027510/ ENSMUSG00000060938	3
GO:0071158	positive regulation of cell cycle arrest	2/17	38/21087	0.0004	0.006	0.004	ENSMUSG00000027510/ ENSMUSG00000060938	2
GO:0044843	cell cycle G1/S phase transition	3/17	194/21087	0.0005	0.006	0.004	ENSMUSG00000031490/ ENSMUSG00000027510/ ENSMUSG00000060938	3
GO:0044773	mitotic DNA damage checkpoint	2/17	57/21087	0.0010	0.012	0.008	ENSMUSG00000027510/ ENSMUSG00000060938	2
GO:0071156	regulation of cell cycle arrest	2/17	61/21087	0.0011	0.013	0.009	ENSMUSG00000027510/ ENSMUSG00000060938	2
GO:2000134	negative regulation of G1/S transition of mitotic cell cycle	2/17	64/21087	0.0012	0.013	0.009	ENSMUSG00000027510/ ENSMUSG00000060938	2

GO:0030330	DNA damage response. signal transduction by p53 class mediator	2/17	65/21087	0.0012	0.013	0.009	ENSMUSG00000027510/ ENSMUSG00000060938	2
GO:0044774	mitotic DNA integrity checkpoint	2/17	65/21087	0.0012	0.013	0.009	ENSMUSG00000027510/ ENSMUSG00000060938	2
GO:1902807	negative regulation of cell cycle G1/S phase transition	2/17	69/21087	0.0014	0.015	0.010	ENSMUSG00000027510/ ENSMUSG00000060938	2
GO:0042770	signal transduction in response to DNA damage	2/17	92/21087	0.0025	0.025	0.017	ENSMUSG00000027510/ ENSMUSG00000060938	2
GO:0045787	positive regulation of cell cycle	3/17	353/21087	0.0027	0.026	0.018	ENSMUSG00000031490/ ENSMUSG00000027510/ ENSMUSG00000060938	3
GO:0000077	DNA damage checkpoint	2/17	102/21087	0.0030	0.028	0.019	ENSMUSG00000027510/ ENSMUSG00000060938	2
GO:0031570	DNA integrity checkpoint	2/17	112/21087	0.0036	0.033	0.022	ENSMUSG00000027510/ ENSMUSG00000060938	2
GO:2000045	regulation of G1/S transition of mitotic cell cycle	2/17	119/21087	0.0041	0.036	0.024	ENSMUSG00000027510/ ENSMUSG00000060938	2
GO:0007093	mitotic cell cycle checkpoint	2/17	130/21087	0.0048	0.041	0.028	ENSMUSG00000027510/ ENSMUSG00000060938	2
GO:1902806	regulation of cell cycle G1/S phase transition	2/17	134/21087	0.0051	0.042	0.029	ENSMUSG00000027510/ ENSMUSG00000060938	2
GO:0072331	signal transduction by p53 class mediator	2/17	137/21087	0.0053	0.043	0.029	ENSMUSG00000027510/ ENSMUSG00000060938	2
GO:0007050	cell cycle arrest	2/17	143/21087	0.0058	0.044	0.030	ENSMUSG00000027510/ ENSMUSG00000060938	2
GO:1901991	negative regulation of mitotic cell cycle phase transition	2/17	144/21087	0.0059	0.044	0.030	ENSMUSG00000027510/ ENSMUSG00000060938	2
**GO terms significantly enriched in gene cluster 5**								
**ID**	**Description**	**GeneRatio**	**BgRatio**	**pvalue**	**p.adjust**	**qvalue**	**geneID**	**Count**
GO:0000281	mitotic cytokinesis	3/20	68/21087	3.52×10^{-05}	0.016	0.011	ENSMUSG00000041147/ ENSMUSG00000024989/ ENSMUSG00000037725	3
GO:0061640	cytoskeleton−dependent cytokinesis	3/20	93/21087	8.97×10^{-05}	0.020	0.014	ENSMUSG00000041147/ ENSMUSG00000024989/ ENSMUSG00000037725	3

GO:0007059	chromosome segregation	4/20	318/21087	0.0002	0.030	0.021	ENSMUSG00000041147/ ENSMUSG00000028820/ ENSMUSG00000028312/ ENSMUSG00000005233	4
GO:0000910	cytokinesis	3/20	158/21087	0.0004	0.047	0.033	ENSMUSG00000041147/ ENSMUSG00000024989/ ENSMUSG00000037725	3

GeneRatio represents the ratio between the number of DEGs and the number of total genes associated with a GO term in the Mus musculus genome; BgRatio indicates the ratio between the number of background genes annotated to GO terms and the total background genes; Count represents the amount of DEGs associated with each GO term.
